# Architecture-Guided Sequential Editing of Distinct Disulfides in Unprotected Peptides

**DOI:** 10.1021/acs.orglett.6c03451

**Published:** 2026-09-29

**Authors:** Samrat Kundu, Adrija Majumdar, Monika Raj

**Affiliations:** Department of Chemistry, 1371 Emory University, Atlanta, Georgia 30322, United States

## Abstract

Disulfide modification typically relies on global reduction, preventing the selective manipulation of individual disulfide bonds within complex peptides. Here, we show that aromatic, ethyl, and *tert*-butyl disulfides embedded in fully unprotected peptides exhibit distinct intrinsic reactivities that can be selectively decoded by using matched activation modes. This architecture-guided hierarchy enables sequential chemoselective functionalization without the use of protecting groups. Aromatic disulfides further undergo an orthogonal phosphite-mediated transformation, expanding the repertoire of programmable disulfide editing.

## Graphical abstract

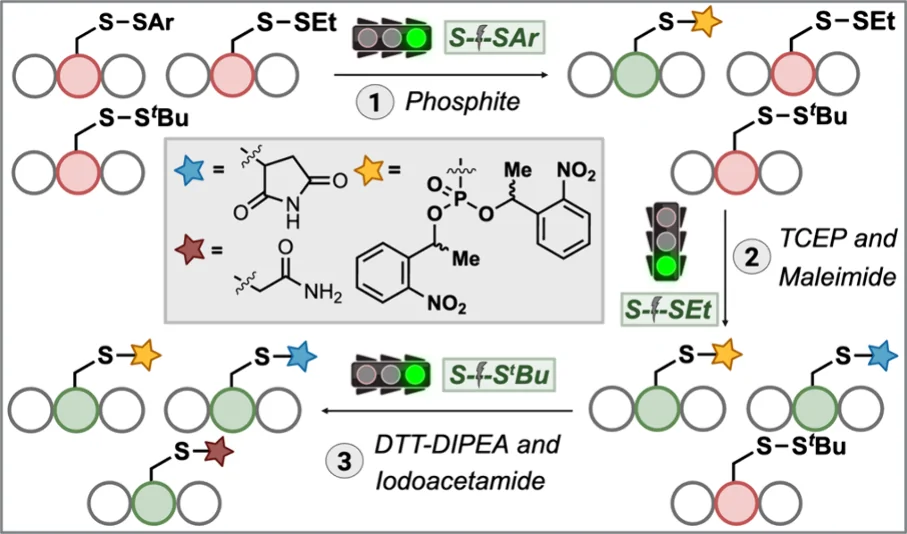

Disulfide bonds are among the most versatile functional groups in peptide chemistry owing to their reversible redox chemistry and susceptibility to thiol–disulfide exchange, which support protein folding, structural stability, biological activity, and chemical modification.^1,2^ Conventional disulfide functionalization, however, generally treats all disulfides as chemically equivalent by reducing them globally to free thiols prior to derivatization.^3^ Although powerful, this strategy erases the chemical identity of individual disulfides and precludes selective modification of specific S–S linkages within complex peptide architectures.

Selective manipulation of individual cysteine residues has traditionally relied on orthogonal nondisulfide protecting groups introduced during peptide synthesis.^4^ Sequential deprotection using acids, bases, or other orthogonal conditions provides a well-established strategy for site-selective cysteine functionalization (Figure 1A).^5^ These approaches are highly effective when orthogonal nondisulfide protecting groups are incorporated during peptide synthesis. Here, we provide a complementary strategy for the late-stage diversification of fully unprotected peptides by directly discriminating among pre-existing disulfide architectures based on their intrinsic reactivity.

**Figure 1:**
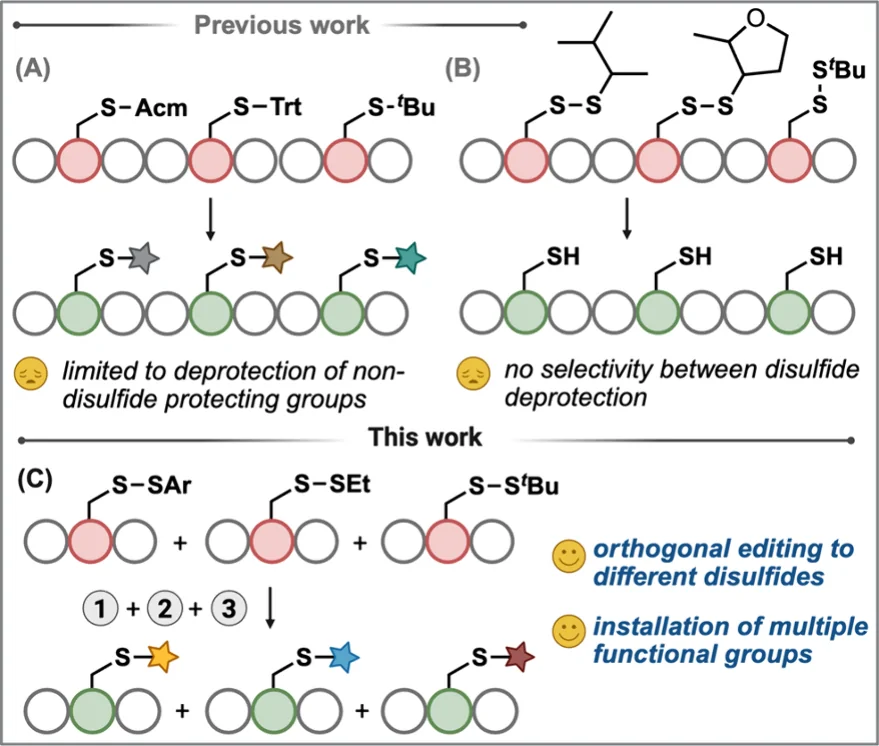
(A) Previous methods utilize orthogonal non-disulfide protecting groups for selective modification of cysteine on peptides. (B) Previous methods unable to modify different disulfides selectively under reducing conditions. (C) This work exploits intrinsic differences among disulfide architectures to selectively cleave and functionalize individual S–S bonds in fully unprotected peptides.

We envisioned an alternative strategy in which the disulfide bond itself serves as the source of the selectivity. Because disulfides differ in steric and electronic properties, we hypothesized that distinct disulfide architectures would possess characteristic activation profiles that could be selectively addressed using appropriately matched reaction conditions.^6^ Rather than viewing disulfides as interchangeable functional groups, this approach exploits their intrinsic chemical identity to enable predictable and programmable editing. Although previous studies have shown that different disulfide classes can display distinct reactivity, selective cleavage of one disulfide architecture in the presence of another remains underdeveloped (Figure 1B).^5d^

Here we demonstrate that aromatic (S–SAr), ethyl (S–SEt), and *tert*-butyl (S–S^*t*^Bu) disulfides embedded within fully unprotected peptides constitute three orthogonal classes of chemical reactivity (Figure 1C). Matched activation modes selectively address each architecture while preserving the remaining disulfides, enabling the sequential installation of multiple functional groups without global reduction.^7^ We further identified a reduction-free phosphite-mediated transformation unique to aromatic disulfides that provides an additional orthogonal editing pathway. Together, these studies establish disulfide architecture as a programmable determinant of chemoselective peptide modification and provide a general framework for the sequential editing of multiple disulfides in fully unprotected peptides.

## Architecture-Dependent Reactivity Establishes a Programmable Disulfide Hierarchy

We first examined whether structurally distinct disulfides display predictable differences in their reactivity toward commonly used reducing agents. Model amino acid derivatives containing aromatic (S–SAr^1^) **1a**, ethyl (S–SEt) **1b**, or *tert*-butyl (S–S^*t*^Bu) **1c** disulfides were evaluated using DTT, TCEP, and phosphine-based reagents (Table 1). A screen of reductants revealed that DTT selectively reduced the aromatic disulfide S–SAr while leaving both S–SEt and S–S^*t*^Bu unchanged (Table 1, entry 2 vs entries 7 and 12, Table S1). Greater S–S bond polarization and stabilization of the departing aryl thiolate likely underlie the increased activation of the aryl disulfide, thereby lowering the barrier to exchange. Under near-neutral conditions, the low concentration of reactive DTT thiolate may further enhance discrimination by favoring reaction with the more activated aryl disulfide over the less reactive aliphatic disulfides.

**Table 1:**
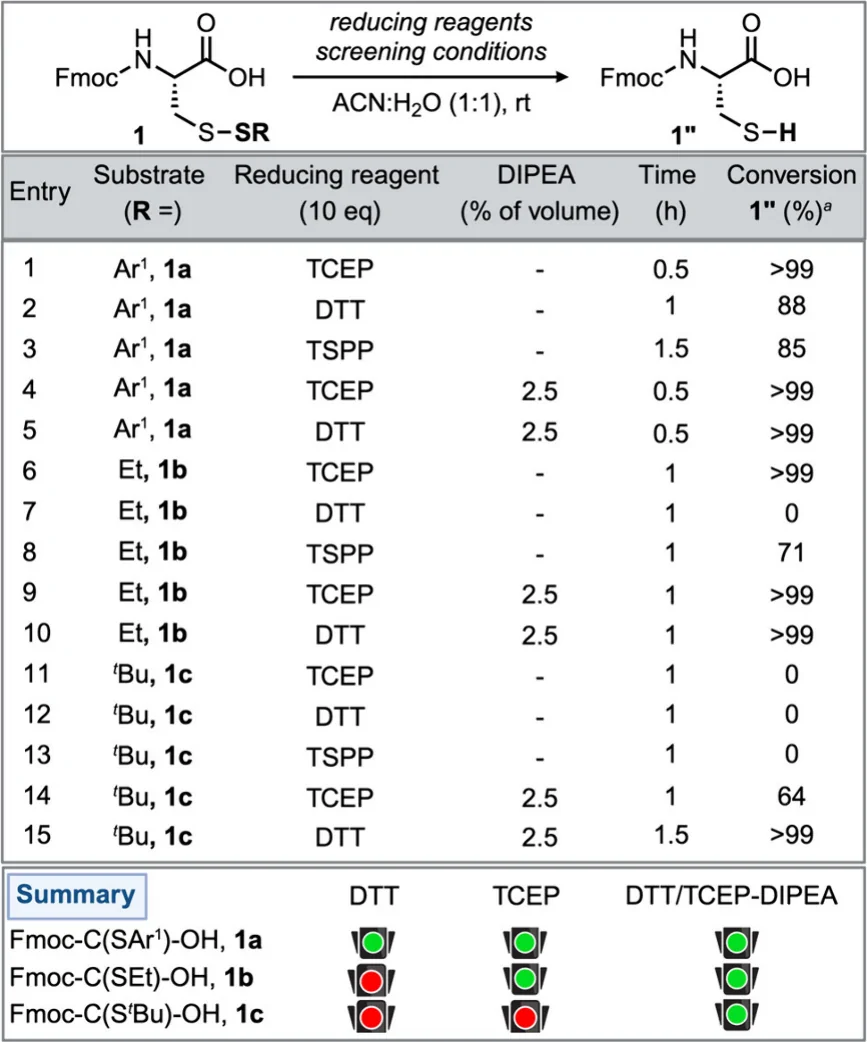
Establishing an Operational Reactivity Hierarchy for Selective Cleavage of Three Disulfide Classes^a^

a S–SAr is selectively cleaved by DTT, S–SEt by TCEP, and S–S^*t*^Bu only under base-assisted DTT/DIPEA or TCEP/DIPEA conditions. DTT = dithiothreitol, TCEP = tris­(2-carboxyethyl)­phosphine, TSPP = tris­(3-sulfonatophenyl)­phosphine hydrate, Ar^1^ = (4-NO_2_)­(3-CO_2_Me)­C_6_H_3_.

In contrast, TCEP selectively cleaved the S–SEt disulfide without detectable cleavage of the more sterically hindered S–S^*t*^Bu analogue (Table 1, entry 6 vs entry 11, Table S2). This selectivity likely reflects TCEP’s direct nucleophilic phosphorus attack, which favors the less sterically hindered S–SEt linkage without requiring thiolate formation. The S–S^*t*^Bu linkage was the most resistant architecture, and its activation required the addition of base and proceeded efficiently only under DIPEA-assisted DTT or TCEP conditions (Table 1, entries 14 and 15, Table S3).^5d^ DIPEA likely increases the concentration of reactive DTT thiolate, enabling attack on the sterically shielded S–S^t^Bu linkage.

## Sequential Chemoselective Editing of Paired Disulfides

We next examined whether the reactivity hierarchy remained predictive when two disulfide architectures were present simultaneously. Fully unprotected peptides containing S–SAr/S–S^*t*^Bu or S–SEt/S–S^*t*^Bu combinations, together with a mixture of S–SAr- and S–SEt-containing peptides, were subjected to the corresponding activation conditions (Figure 2, Figures S2–S5).

**Figure 2:**
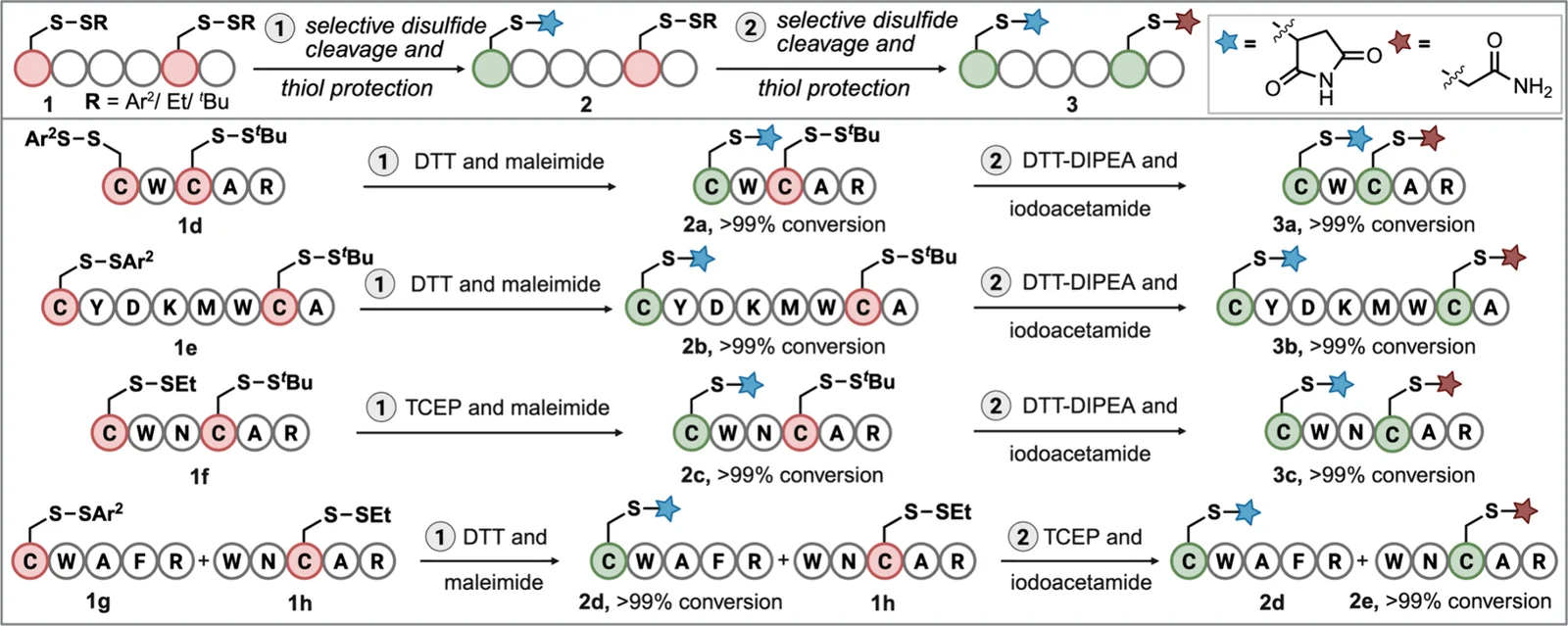
Orthogonal cleavage and modification of paired disulfides in fully unprotected peptides. Pairwise peptide substrates demonstrate that the activation hierarchy is preserved in the presence of competing disulfide linkages and native amino-acid side chains. Reaction conditions: **1** (1 equiv), DTT or TCEP (10 equiv), DIPEA (if required, 2.5% of volume) followed by thiol protecting group (IAA/maleimide, 10 equiv). Ar^2^ = (4-NO_2_)­(3-CO_2_H)­C_6_H_3_.

Treatment of peptides **1d** and **1e** bearing S–SAr and S–S^*t*^Bu linkages, with DTT selectively cleaved the aromatic disulfide while preserving S–S^*t*^Bu. Trapping of the newly generated thiol with maleimide afforded the corresponding monofunctionalized products (**2a**, **2b**) in near-quantitative conversion (Figure 2, Figures S2 and S3). Subsequent treatment with DTT/DIPEA activated the remaining S–S^t^Bu linkage, enabling installation of a second functional group with iodoacetamide (IAA) to furnish **3a** and **3b** (Figure 2, Figures S2 and S3). Chemoselectivity was also maintained in **1e**, which contains Lys, Tyr, Met, Trp, and Asp residues in addition to S–SAr and S–S^*t*^Bu linkages. We have further established the sequence diversity, position tolerance, and Trp independence of our methodology under the optimized conditions by incorporating His, Ser, and Glu residues, introducing a disulfide bond at the C-terminus, and eliminating aromatic residues, respectively, in different peptide sequences. Importantly, no detectable modifications of these native side chains were observed (Figure S3). In peptide **1f**, containing S–SEt and S–S^*t*^Bu linkages, TCEP selectively cleaved S–SEt while leaving the more hindered S–S^*t*^Bu linkage intact. After trapping the newly generated thiol with maleimide to afford **2c**, the remaining S–S^*t*^Bu linkage was activated under base-assisted conditions.

DTT/DIPEA conditions and functionalized with IAA to provide **3c** in near-quantitative conversion (Figure 2, Figure S4). The distinction between S–SAr and S–SEt was further evaluated using a mixture of peptides **1g** and **1h**, each containing one of the architectures. DTT selectively activated the S–SAr-containing peptide, followed by functionalization of free thiols with maleimide (**2d**). Subsequent treatment with TCEP selectively cleaved the remaining S–SEt linkage, followed by sequential functionalization with IAA to afford **2e** under orthogonal conditions (Figure 2, Table S4, and Figure S5). These experiments establish our hypothesis of selectively addressing each disulfide architecture by a matched activation mode while preserving the other for subsequent transformations.

## Reduction-Free Editing of Aromatic Disulfides

The distinctive reactivity of aromatic disulfides prompted us to investigate whether S–SAr linkages could undergo direct conversion without prior formation of a free thiol.^8^ Screening phosphite-based reagents revealed that aryl phosphite P­(OAr_3_)_3_ converted S–SAr-containing peptides to the corresponding phosphite-modified product **4a**. Because this nitrobenzylphosphite reagent is not commercially available, it was synthesized as described in the Supporting Information and handled under conditions that minimize exposure to air due to its air sensitivity (Figure S7). In contrast, alkyl phosphite P­(OEt)_3_ showed poor reactivity (entries 1 and 2, Table 2, Figures S8 and S9). Initial substrates displayed moderate conversion, whereas incorporation of electron-withdrawing substituents into the aryl disulfide **1g** afforded the corresponding product, **4c**, in near-quantitative conversion within 4 h (Table 2, entry 3, Figure S9). This enhancement is consistent with increased polarization of the S–S bond and stabilization of the departing aryl thiolate, which may facilitate phosphorus-mediated substitution. Although the detailed mechanism remains to be established, these observations support a pathway distinct from conventional reduction, followed by thiol functionalization.

**Table 2:**
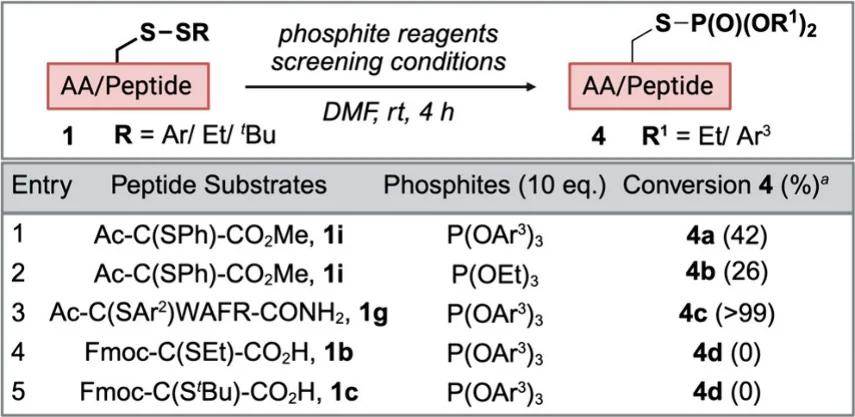
Establishing Reduction Free Selective Modification of Aryl Disulfides with Phosphite Reagents

a Reaction conditions: 1 (1 equiv), phosphites (10 equiv). Ar^2^ = (4-NO_2_)­(3-CO_2_H)­C_6_H_3_, Ar^3^ = 1-ethyl-2-nitrobenzene.

Importantly, the aliphatic disulfide S–SEt and S–S^*t*^Bu linkages remained intact under the phosphite-mediated conditions (Table 2, entries 4 and 5, Figure S9). Aromatic disulfides can therefore be addressed through either selective reductive activation or a complementary reduction-free pathway, providing two distinct editing modes while preserving the competing aliphatic disulfides.

## Reduction-Free Sequential Editing of Paired Disulfides

We next examined whether phosphite-mediated S–SAr modification could serve as the first step in sequential editing of paired disulfide architectures (Figure 3A, Figures S10–S13). In a peptide **1e** containing S–SAr and S–S^*t*^Bu linkages, the optimized phosphite selectively converted the aromatic disulfide S–SAr to the phosphorus-modified product **4e**, while leaving S–S^*t*^Bu intact. Subsequent DTT/DIPEA-mediated cleavage of S–S^*t*^Bu, followed by maleimide trapping, afforded the doubly modified peptide **5e** in >99% conversion (Figure 3A, Figure S10). In addition, the sequence has been further evaluated in this peptide, when potentially reactive residues Tyr, Asp, Lys, Met, and Trp were chemically untouched throughout the process and no detectable modification of the native amino acid side chains was observed, demonstrating that this method remains chemoselective in a densely functionalized, fully unprotected peptide environment. Orthogonality between S–SAr and S–SEt was demonstrated using a mixture of peptides **1g** and **1h**. Phosphite selectively modified the S–SAr-containing peptide **1g** to **4c**, whereas the S–SEt-containing peptide **1h** remained unchanged. Subsequent TCEP reduction and thiol trapping with maleimide afforded **2f**-functionalized S–SEt (Figure 3A, Figure S11). These results establish phosphite-mediated S–SAr conversion as a reduction-free entry point for sequential editing while preserving aliphatic disulfides for later activation.

**Figure 3:**
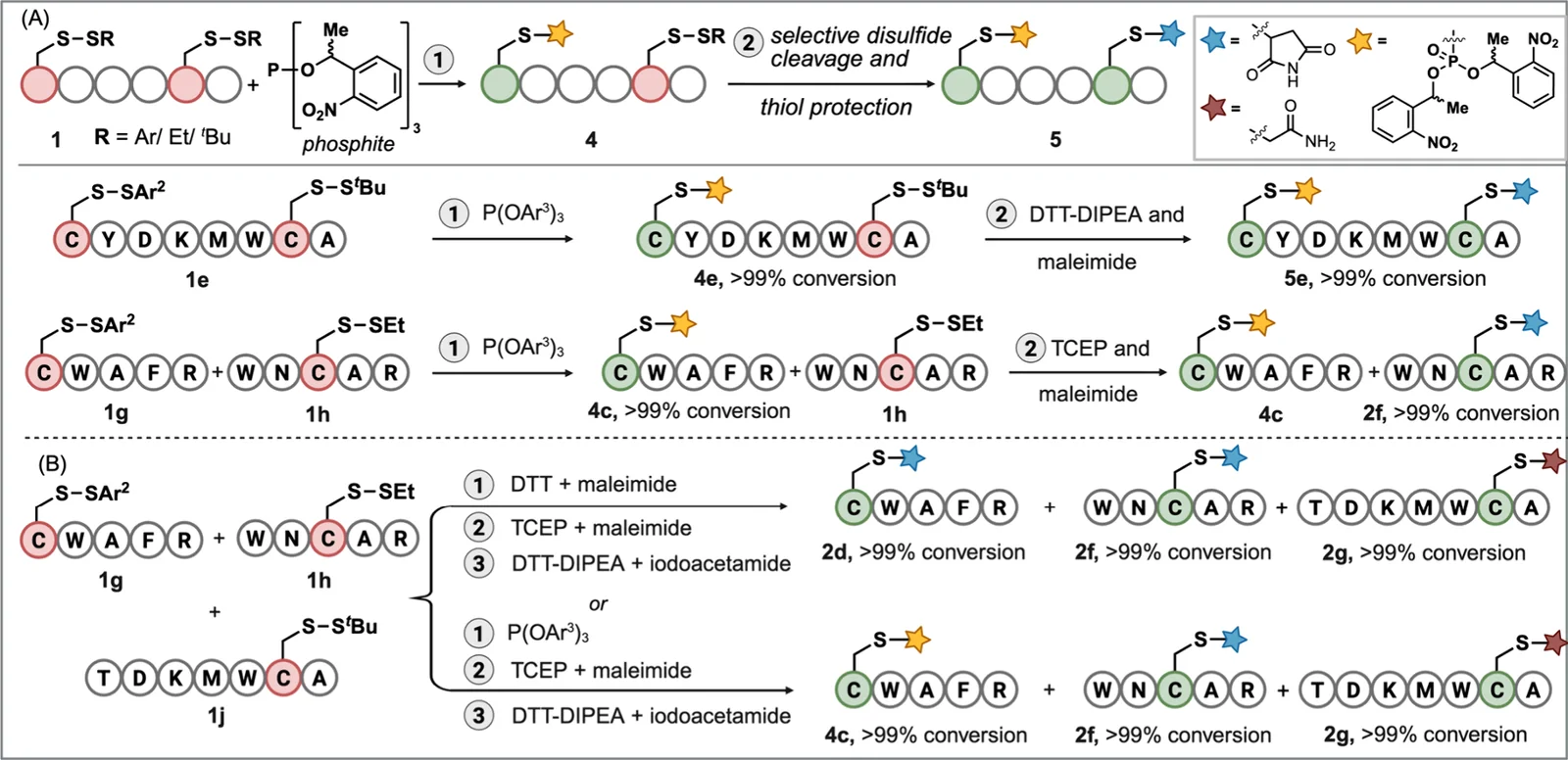
(A) Direct phosphorus-mediated modification of aromatic disulfides. Aromatic S–SAr linkages undergo direct conversion with phosphorus-based reagents without requiring a discrete reduction step or global thiol exposure. (B) Establishing pairwise orthogonality and aromatic-disulfide direct conversion on peptide containing all three disulfide architectures. Reaction Conditions: **1** (1 equiv), phosphites (10 equiv), DTT or TCEP (10 equiv), DIPEA (if required, 2.5% of volume) followed by thiol protecting group (IAA/maleimide, 10 equiv). Ar^3^ = 1-ethyl-2-nitrobenzene.

## Sequential Editing of Three Disulfide Architectures in Fully Unprotected Peptides

Having established selective activation of individual and paired disulfides, we next evaluated whether all three architectures could be addressed sequentially within a single reaction sequence. A mixture of peptides containing S–SAr^2^ (**1g**), S–SEt (**1h**), and S–S^*t*^Bu (**1j**) linkages was subjected to the matched activation conditions in the order defined by the reactivity hierarchy (Figure 3B, Figures S12 and S13).

Following the operational hierarchy, peptides were mixed and first treated with DTT to selectively cleave S–SAr^2^ in **1g**, which was functionalized with maleimide to afford **2d**, while **1h** and **1j** remained unchanged. Subsequent treatment with TCEP selectively activated S–SEt in **1h**, enabling installation of a second functional group on reaction with maleimide and forming **2f** without affecting S–S^*t*^Bu in **1j**. Finally, exposure to DTT/DIPEA cleaved the remaining S–S^*t*^Bu linkage, which was functionalized with IAA and furnished **2g** (Figure 3B, Figure S12). Each step proceeded with high conversion and preserved the disulfide architectures designated for later activations.

The sequence could also be initiated through direct phosphorus-mediated editing of aromatic disulfide. Treatment of the three-component mixture with P­(OAr^3^)_3_ selectively converted the S–SAr^2^-containing peptide **1g** to phosphite product **4c** (Figure 3B, Figure S13). The remaining disulfides containing peptides (**1h**–**1j**) were then addressed sequentially using TCEP and base-assisted DTT followed by selective functionalization, respectively, and afforded **2f** and **2g** in high conversion (Figure 3B, Figure S13). This alternative sequence demonstrates that reductive and reduction-free activation modes can be combined within the same workflow to expand the order and type of transformations available for disulfide editing.

We developed an architecture-guided strategy for sequential disulfide editing in fully unprotected peptides. Aromatic, ethyl, and *tert*-butyl disulfides display distinct activation profiles that permit their selective transformation using DTT, TCEP, and base-assisted reducing conditions, respectively. This selectivity arises from the interplay of the disulfide architecture, reductant mechanism, and base activation rather than from external protecting groups or global thiol exposure. Aromatic disulfides also undergo direct phosphite-mediated modification without prior reduction, thereby providing a complementary reduction-free editing pathway. Together, these results establish intrinsic disulfide architecture as a programmable source of chemoselectivity and expand disulfide chemistry from a global reduction strategy to a set of selectively addressable transformations for the stepwise diversification of unprotected peptide scaffolds.

## Supplementary Material



## Data Availability

The data underlying this study are available in the published article and its Supporting Information.

## Notes

General experimental procedures and characterization details, including HPLC, HRMS, and ^1^H and ^13^C NMR spectra of all reported compounds (PDF)
