# Supplementary material for: Architecture-Guided Sequential Editing of Distinct Disulfides in Unprotected Peptides
Source: Org Lett. 2026 Sep 29;28(40):12594–8. doi: 10.1021/acs.orglett.6c03451 (PMC13660385; doi:10.1021/acs.orglett.6c03451)

Supporting Information

Architecture-Guided Sequential Editing of Distinct Disulfides in Unprotected Peptides

Samrat Kundu,[#] Adrija Majumdar,[#] Monika Raj*

Department of Chemistry, Emory University, Atlanta, Georgia 30322, United States

E-mail: *monika.raj@emory.edu*

Table of Contents

I. General...5

II. Materials...5

III. Purification...5

IV. Instrumentation and Sample Analysis...5

V. Fmoc-Solid Phase Peptide Synthesis...6

VII. Synthesis of *N*-(((9*H*-fluoren-9-yl)methoxy)carbonyl)-*S*-(ethylthio)-*L*-cysteine 1b.......................................8

VIII. Table S1. Screening of Reducing Reagents for Disulfide Reduction on Compound *N*-(((9*H*-fluoren-9-yl)methoxy)carbonyl)-*S*-((3-(methoxycarbonyl)-4-nitrophenyl)thio)-*L*-cysteine 1a. 9

IX. Table S2. Screening of Reducing Reagents for Disulfide Reduction on Compound *N*-(((9*H*-fluoren-9-yl)methoxy)carbonyl)-*S*-(ethylthio)-*L*-cysteine 1b. 13

X. Table S3. Screening of Reducing Reagents for Disulfide Reduction on Compound *N*-(((9*H*-fluoren-9-yl)methoxy)carbonyl)-*S*-(*tert*-butylthio)-*L*-cysteine 1c. 15

XI.1. Supplementary Figure 1. Synthesis of Aryl Disulfide in Cys-Containing Peptide Sequence............19

XI.1.1. Synthesis of Peptide Ac-C(SAr^2)WC(S^{t}Bu)AR-CONH$_2$ **1d**:......................................19

XI.1.2. Synthesis of Peptide Ac-C(SAr^2)YDKMWC(S^{t}Bu)A-CONH$_2$ **1e**: 20

XI.1.3. Synthesis of Peptide Ac-C(SAr^2)WAFR-CONH$_2$ **1g**. 21

XI.2. Synthesis of Ethyl Disulfide in Cys-Containing Peptide Sequence......................................22

XI.2.1. Synthesis of Peptide Ac-C(SEt)WNC(S^{t}Bu)AR-CONH$_2$ **1f**:......................................22

XI.2.2. Synthesis of Peptide Ac-WNC(SEt)AR-CONH$_2$ **1h**:......................................23

XII.a. Supplementary Figure 2. Orthogonal Cleavage and Modification of Aryl and tert-Butyl containing Disulfides in Fully Unprotected Peptide **1d**.......................................24

XII.a.1. Orthogonal Cleavage of Aryl Containing Disulfide on Peptide **1d**.......................................24

XII.a.2. Orthogonal Modification of Free Cys on Unprotected Peptide **1d''**. 26

XII.a.3. Orthogonal Cleavage of *tert*-Butyl Containing Disulfide on Peptide **2a**. 27

XII.a.4. Orthogonal Modification of Free Cys on Unprotected Peptide **2a''**. 28

XII.b. Supplementary Figure 3. Orthogonal Cleavage and Modification of Aryl and *tert*-Butyl Containing Disulfides in Fully Unprotected Peptide **1e**. 30

XII.b.1. Orthogonal Cleavage of Aryl Containing Disulfide on Peptide **1e**. 30

XII.b.2. Orthogonal Modification of Free Cys on Unprotected Peptide **1e''**. 31

XII.b.3. Orthogonal Cleavage of *tert*-Butyl Containing Disulfide on Peptide **2b**. 32

XII.b.4. Orthogonal Modification of Free Cys on Unprotected Peptide **2b''**. 33

XII.b.5. Orthogonal Cleavage and Modification of Disulfide in Chemo-selective, Position-independent, Non-aromatic Residue Containing Peptide 35

XII.c. Supplementary Figure 4. Orthogonal Cleavage and Modification of Ethyl and *tert*-Butyl Containing Disulfides in Fully Unprotected Peptide **1f** 38

XII.c.1. Orthogonal Cleavage of Ethyl Containing Disulfide on Peptide **1f**. 38

XII.c.2. Orthogonal Modification of Free Cys on Unprotected Peptide **1f''**. 39

XII.c.3. Orthogonal Cleavage of *tert*-Butyl Containing Disulfide on Peptide **2c** 40

XII.c.4. Orthogonal Modification of Free Cys on Unprotected Peptide **2c''** 42

XII.d. Table S4: Screening of Reaction Conditions for Orthogonal Cleavage of Aryl-Containing Disulfide on 1g from Unprotected Peptide Mixture of **1g** and **1h**. 43

XII.d. Supplementary Figure 5. Orthogonal Cleavage and Modification of Aryl and Ethyl Containing Disulfides in Fully Unprotected Peptide mixture of **1g** and **1h**. 49

XII.d.1. Orthogonal Cleavage of Aryl Containing Disulfide on 1g from the Mixture of Peptides **1g** and **1h**. 49

XII.d.2. Orthogonal Modification of Free Cys on **1g''** from the Mixture **1g''** and **1h**. 51

XII.d.3. Orthogonal Cleavage of Ethyl Containing Disulfide on **1h** in the Mixture of Peptides **1h** and **2d**. 53

XII.d.4. Orthogonal Modification of Free Cys in Peptide **1h''** in the Mixture of **1h''** and **2d**. 55

XIII. Supplementary Figure 6. Synthesis of Methyl *N*-acetyl-*S*-(phenylthio)-*L*-cysteinate **1i**. 57

XIV. Supplementary Figure 7. Synthesis of tris(1-(2-Nitrophenyl)ethyl)phosphite **S10**.[4] 58

XV. Supplementary Figure 8. Reaction of Aryl-Phosphite **S10** on Methyl *N*-acetyl-*S*-(phenylthio)-*L*-cysteinate **1i**. 59

XV.1. Large Scale Synthesis of Methyl N-Acetyl-S-(bis(1-(2-nitrophenyl)ethoxy)phosphoryl)-L-cysteinate **4a**. 60

XVI. Supplementary Figure 9. Reaction of alkyl-phosphite S11 on Methyl *N*-acetyl-*S*-(phenylthio)-*L*-cysteinate **1i**. 61

XVI.1. Large Scale Synthesis of methyl *N*-acetyl-*S*-(diethoxyphosphoryl)cysteinate **4b**. 62

XVII. Reaction of Aryl-Phosphite **S10** on Aryl-Disulfide Contain Peptide **1g**. 63

XVIII. Reaction of Aryl-Phosphite **S10** on Fmoc-C(SEt)-OH **1b**. 64

XIX. Reaction of Aryl-Phosphite **S10** on Fmoc-C(S^tBu)-OH **1c**. 65

XX.a. Supplementary Figure 10. Phosphite Mediated Orthogonal Cleavage and Modification of Aryl and *tert*-Butyl Containing Disulfides on Fully Unprotected Peptide **1e**. 66

XX.a.1. Orthogonal Phosphite-Modification of Aryl Disulfide on Peptide **1e**. 66

XX.a.2. Orthogonal Cleavage of *tert*-Butyl Containing Disulfide on Compound **4e**. 68

XX.a.3. Orthogonal Modification of Free Cys on Unprotected Peptide **4e"**. 68

XX.b. Supplementary Figure 11. Phosphite Mediated Orthogonal Cleavage and Modification of Aryl and Ethyl Containing Disulfides on Fully Unprotected Peptide mixture of **1g** and **1h**. 70

XX.b.1. Orthogonal Phosphite-Modification of Aryl Disulfide on Peptide **1g** in the Mixture of Peptides **1g** and **1h.** 70

XX.b.2. Orthogonal Cleavage of Ethyl Containing Disulfide on Compound **1h** in the Mixture of Peptides **4c** and **1h**. 72

XX.b.3. Orthogonal Modification of Free Cys on **1h"** in the Mixture Peptides of **4c** and **1h"**. 75

XXI.a. Supplementary Figure 12. Orthogonal Cleavage and Modification of Aryl, Ethyl and *tert*-Butyl Containing Disulfides in Fully Unprotected Peptide mixture of **1g**, **1h** and **1j**. 77

XXI.a.1. Orthogonal Cleavage of Aryl Containing Disulfide on **1g** from the Mixture of Peptides **1g**, **1h** and **1j**. 77

XXI.a.2. Orthogonal Modification of Free Cys on **1g**" from the Mixture **1g"**,**1h** and **1j**. 79

XXI.a.3. Orthogonal Cleavage of Ethyl Containing Disulfide on **1h** in the Mixture of Peptides **1h**, **1j** and **2d**. 81

XXI.a.4. Orthogonal Modification of Free Cys in Peptide **1h"** in the Mixture of **1h"**, **1j** and **2d**. 83

XXI.a.5. Orthogonal Cleavage of *tert*-Butyl Containing Disulfide on **1j** in the Mixture of Peptides **2f**, **1j** and **2d**. 86

XXI.a.6. Orthogonal Modification of Free Cys in Peptide **1j"** in the Mixture of **1j"**, **2f** and **2d**. 88

XXI.b.1 Supplementary Figure 13. Phosphite Mediated Orthogonal Cleavage and Modification of Aryl, Ethyl and *tert*-Butyl Containing Disulfides on Fully Unprotected Peptide mixture of **1g**, **1h** and **1j**. 90

XXI.b.2. Orthogonal Cleavage of Ethyl-Containing Disulfide on **1h** from the Mixture **4c**, **1h** and **1j**.. 92

XXI.b.3. Orthogonal Modification of Free Cys on **1h"** from the Mixture **4c**, **1h"** and **1j**. 93

XXI.b.4. Orthogonal Cleavage of *tert*-Butyl Containing Disulfide on **1j** in the Mixture of Peptides **2f**, **1j** and **4c**. 95

XXI.b.5. Orthogonal Modification of free Cys in Peptide **1j"** in the Mixture of **1j"**, **2f** and **4c**. 95

XXII. References 97

I. General. All commercial materials (Sigma-Aldrich, Fluka and Novabiochem) were used without further purification. All solvents were reagent or HPLC (Fisher) grade. All reactions were performed under air in glass vials or round bottom flasks. Conversions refer to chromatographically pure compounds; % conversions were obtained by comparing HPLC peak areas of products and starting materials. HPLC and HRMS were used to monitor reaction progress, and products were characterized using HRMS and NMR.

II. Materials. Fmoc-amino acids, Rink amide resin, 3[bis(dimethylamino)methyliumyl]-3H-benzotriazol-1-oxide hexafluorophosphate (HBTU), and N,N-Diisopropylethylamine (DIPEA) were obtained from CreoSalus (Louisville, Kentucky). Piperidine and trifluoroacetic acid (TFA), were obtained from Alfa Aesar (Ward Hill, Massachusetts). Potassium carbonate (K_2CO_3), N,Ndimethylformamide (DMF), dichloromethane (DCM), methanol (MeOH) and acetonitrile (ACN) were obtained from VWR (100 Matsonford Road Radnor, Pennsylvania). L-Cysteine methyl ester hydrochloride, manganese(IV) oxide were obtained from Sigma Aldrich (3050 Spruce Street, St. Louis, Missouri).Tris(2-carboxyethyl)phosphine hydrochloride (TCEP) was obtained from Combi-Blocks (7949 Silverton Ave #915, San Diego, California). Dithiothreitol (DTT) and formic acid (FA) was obtained from Oakwood Chemical (730 Columbiaa Hwy N. Estill, South Carolina). *N*-(((9*H*-fluoren-9-yl)methoxy)carbonyl)-*S*-(*tert*-butylthio)-*L*-cysteine (**1c**) was purchased from Chem Scene (1 Deer Park Dr, Suite F, Monmouth Junction, NJ 08852).

III. Purification. HPLC: Purification of peptides was performed using preparatory high performance liquid chromatography (HPLC) on an ACCQ Prep HP 150 equipped with a 10 x 150 mm RediSep Prep C18Aq, 100A, 5 µm column. Separations involved a mobile phase of 0.1% FA in water (solvent A) and 0.1 % FA in acetonitrile (solvent B) or mobile phase of water (solvent A) and acetonitrile (solvent B). The eluent was monitored by absorbance at 220 nm.

HPLC Method: C18, Water (A)/ACN (B); gradient: 10 to 65% **B** (0.1 % FA) in 35 min; 65 to 90% **B** (0.1 % FA) in 35.1 to 45 min, flow rate = 4.7 mL/min, I = 220 nm.

IV. Instrumentation and Sample Analysis. NMR. ^{1}H and ^{13}C spectra were acquired at 25 °C in $CDCl_3$, DMSO-d_6 and CD_3CN using a Bruker 400 MHz spectrometer. All ^{1}H NMR chemical shifts (δ) were referenced relative to the residual $CDCl_3$ peak at 7.26 ppm, DMSO-d_6 peak at 2.50 ppm and CD_3CN peak at 1.94 ppm. ^{13}C NMR chemical shifts were referenced to $CDCl_3$ peak at 77.16 ppm, DMSO-d_6 at 39.52 ppm and CD_3CN peak at 1.32 and 118.26 ppm. ^{13}C NMR spectra were proton decoupled. ^{31}P NMR spectra were recorded in CD_3CN solvent without proton decoupled. NMR spectral data are reported as chemical shift (multiplicity, coupling constants (*J*), integration). Multiplicity is reported as follows: singlet (s), doublet (d), doublet of doublets (dd), doublet of triplets (td), triplet (t), quintet (q), septet (sep) and multiplet (m). Coupling constant (*J*) in hertz (Hz).

Analytical HPLC. Analytical HPLC was performed on an Agilent 1100 series HPLC equipped with a 4.6 x 150 mm RediSep Prep C18Aq, 100A, 5 µm column. The reaction was monitored by analytical reverse phase HPLC using a gradient of water versus acetonitrile in linear gradients with a constant flow rate of 1 mL min^{-1}. Separations involved a mobile phase of 0.1% formic acid in water (solvent A) and 0.1% formic acid in acetonitrile (solvent B) or mobile phase of water (solvent A) and acetonitrile (solvent B). The eluent was monitored with a detection wavelength of 220 nm.

Analytical HPLC Method: C18, Water (A)/ACN (B); gradient: 5 to 85% B (0.1% formic acid in ACN) in 20 min, flow rate = 1.0 mL/min, I = 220 nm.

HRMS. High resolution MS data were acquired on a ThermoFisherLTQ Orbitrap Velos mass spectrometer with a heated electrospray source. For direct infusion experiments, the solution was infused at a rate of 300 µl min-1 and the positive ion spray voltage was set to 3.0 kV. The instrument parameters were as follows: scan range = 200-2000 m/z; capillary temp = 320 °C, RF lens = 60%. Spectra were taken at 60,000 resolutions at m/z 200 using Tune software and analyzed with Thermo's Freestyle software.

V. Fmoc-Solid Phase Peptide Synthesis. All starting peptides were manually synthesized on Rink Amide using standard protocols.[1] Resin (291 mg, 0.2 mmol, 1 eq) was swelled in 5 mL DCM for 1 hour. For the initial Fmoc deprotection, the swelling solution was replaced with 5 mL 20% piperidine in DMF and the resin was placed on a wrist action shaker for 20 minutes at room temperature. After Fmoc deprotection, the resin was washed with DMF, MeOH, and DCM (3 x 5 mL each). Subsequent amino acid couplings were performed using Fmoc-protected amino acid (1 mmol, 5 eq), HBTU (1 mmol, 5 eq), and DIPEA (1 mmol, 5 eq) in 2.1 mL DMF, shaking for 30 minutes at room temperature. Iterative deprotection and coupling steps were performed until the full sequence was achieved. Fmoc deprotection was reduced to 15 minutes for these subsequent steps. Peptides were cleaved from the resin using 4 mL of a cocktail consisting of 98:2.5:2.5 trifluoroacetic acid : water : triethylsilane (TES) for 2 h. The resin was removed by filtration, and the resulting solution was concentrated. Peptides were precipitated and centrifugated with cold diethyl ether (3 x 10 mL) to obtain the crude product. Crude peptides were dissolved in ACN:H_2O and purified by **Preparatory HPLC Method**: C18, Water (A)/ACN (B); gradient: 10 to 65% **B** (0.1 % FA) in 35 min; 65 to 90% **B** (0.1 % FA) in 35.1 to 45 min, flow rate = 4.7 mL/min, I = 220 nm.

VI. Synthesis of N-(((9H-fluoren-9-yl)methoxy)carbonyl)-S-((3-(methoxycarbonyl)-4-nitrophenyl)thio)-L-cysteine 1a.

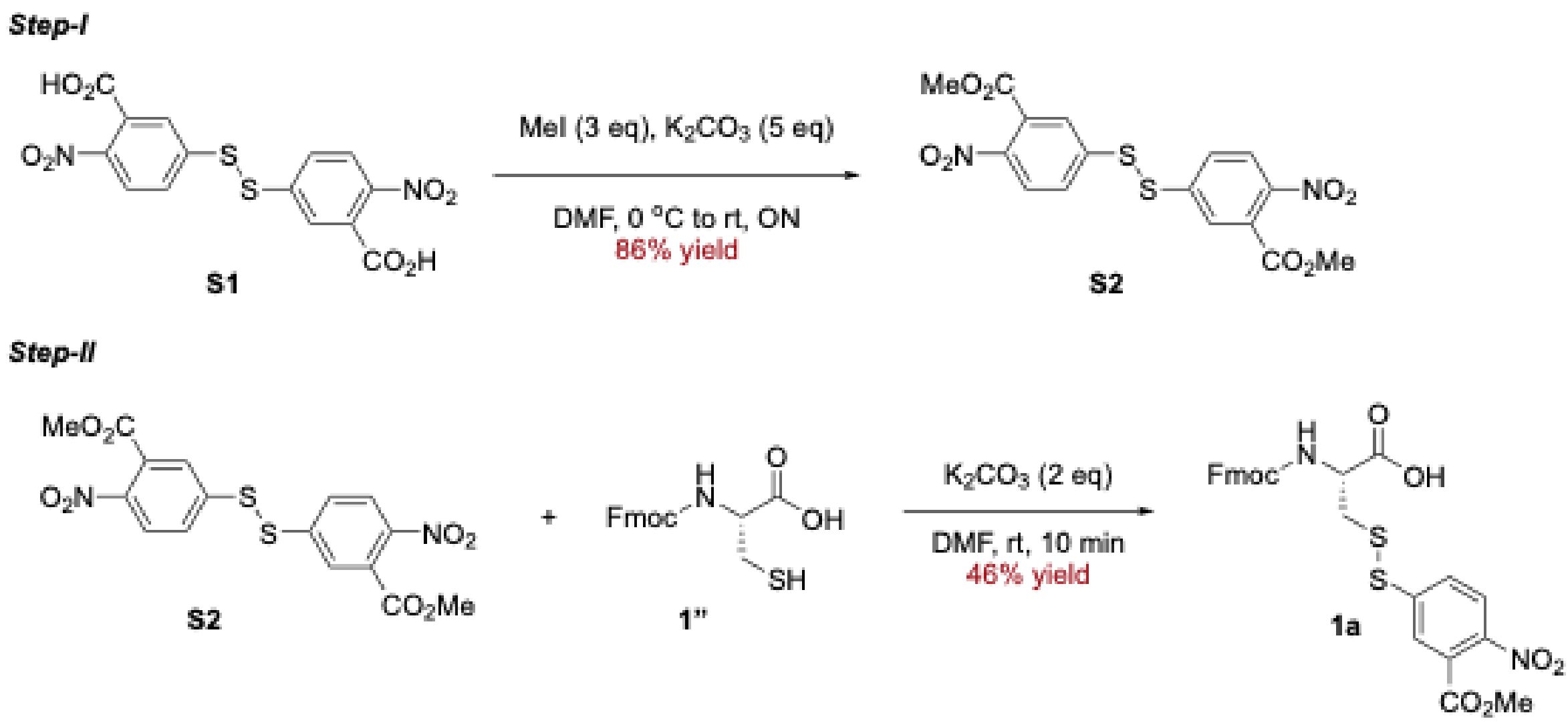

Step I: *Synthesis of dimethyl 5,5'-disulfanediylbis(2-nitrobenzoate) S2:*[1]

In a 100 mL round bottom flask 4,4'-disulfanediylbis(2-nitrobenzoic acid) **S1** (5.29 g, 13.34 mmol, 1 eq) was taken and dissolved in *N,N*-dimethylformamide (27 mL) while stirring at room temperature. Then, the solution was cooled down to 0 ºC and potassium carbonate (K_2CO_3, 9.2 g, 66.5 mmol, 5 eq) was added sequentially and left the solution stirred for 15 min at the same temperature. After that, methyl iodide (MeI, 2.5 mL, 40 mmol, 3 eq) was added dropwise at 0 ºC, gradually increased the temperature to room temperature and left the solution stirred for overnight at the same temperature. After complete consumption of **S1**, monitored by TLC, diluted the reaction mixture with ethyl acetate, washed the organic layer with saturated $NaHCO_3$ (30 ml x 3), water (30 mL) followed by brine solution (30 mL). The accumulated organic layer was evaporated to dryness under vacuum, and the crude product was isolated through silica gel column chromatography using 30% ethyl acetate-hexane as eluent to afford the desired product dimethyl 4,4'-disulfanediylbis(2-nitrobenzoate) **S2** (4.8 g, yellow solid, 86% yield). R_f = (0.7 in ethyl acetate). **^{1}H NMR (400 MHz, $CDCl_3$)** δ (ppm):7.93 (d, *J* = 8.6 Hz, 2H), 7.75 (d, *J* = 2.2 Hz, 2H), 7.67 (dd, *J* = 8.6, 2.2 Hz, 2H), 3.93 (s, 6H).

Step II: *Synthesis of N-(((9H-fluoren-9-yl)methoxy)carbonyl)-S-((3-(methoxycarbonyl)-4-nitrophenyl)thio)-L-cysteine 1a*

In a 25 ml round bottom flask, (((9*H*-fluoren-9-yl)methoxy)carbonyl)-*L*-cysteine **1"** (206 mg, 0.6 mmol, 1 eq) was dissolved in 1.5 mL of DMF while stirring. In another 25 ml round bottom flask, dimethyl 4,4'-disulfanediylbis(2-nitrobenzoate) **S2** (424 mg, 1 mmol, 1.7 eq), potassium carbonate (K_2CO_3, 276 mg, 2 mmol, 3.3 eq) were dissolved in 5 ml DMF. After that, to the second solution, solution of **1"** in DMF was added dropwise over the period of 5 mins at room temperature and left the reaction mixture stirred for 5 mins more at the same temperature. After complete consumption of **1"**, monitored by TLC, diluted the reaction mixture with ethyl acetate (40 ml), washed the organic layer with saturated $NaHCO_3$ (15 mL), followed by brine solution (20 mL). The accumulated organic layer was evaporated to dryness under vacuum, and the crude product was isolated through silica gel column chromatography using 80% ethyl

acetate-hexane to 10% methanol-dichloromethane as eluent to afford the desired product *N*-(((9*H*-fluoren-9-yl)methoxy)carbonyl)-*S*-((4-(methoxycarbonyl)-3-nitrophenyl)thio)-*L*-cysteine **1a** (153 mg, pale yellow solid, 46% yield). R_f = (0.2 in 10% methanol-dichloromethane). **^{1}H NMR (400 MHz, DMSO-*d$_6$*)** δ (ppm): 8.02 (d, *J* = 9.3 Hz, 1H), 7.91 – 7.87 (m, 4H), 7.71 (d, *J* = 10.0 Hz, 2H), 7.54 (d, *J* = 8.3 Hz, 1H), 7.40 (t, *J* = 7.5 Hz, 2H), 7.32 (t, *J* = 7.5 Hz, 2H), 4.27 – 4.21 (m, 3H), 4.16 – 4.08 (m, 2H), 3.83 (s, 3H), 3.28 (dd, *J* = 13.6, 4.2 Hz, 1H), 3.12 (dd, *J* = 13.6, 9.4 Hz, 1H). **^{13}C {^{1}H} NMR (101 MHz, DMSO-*d$_6$*)** δ (ppm): 165.1, 156.0, 145.2, 143.9, 140.9, 140.8, 128.5, 128.0, 127.80, 127.76, 127.2, 125.9, 125.39, 125.36, 120.3, 65.8, 55.0, 54.1, 53.5, 46.7. **HRMS (ESI)** m/z: for $C_{26}H_{22}N_2NaO_8S_2$ $[M + Na]^+$ Calcd for 577.0710, Found 577.0708.

VII. Synthesis of *N*-(((9*H*-fluoren-9-yl)methoxy)carbonyl)-*S*-(ethylthio)-*L*-cysteine 1b.

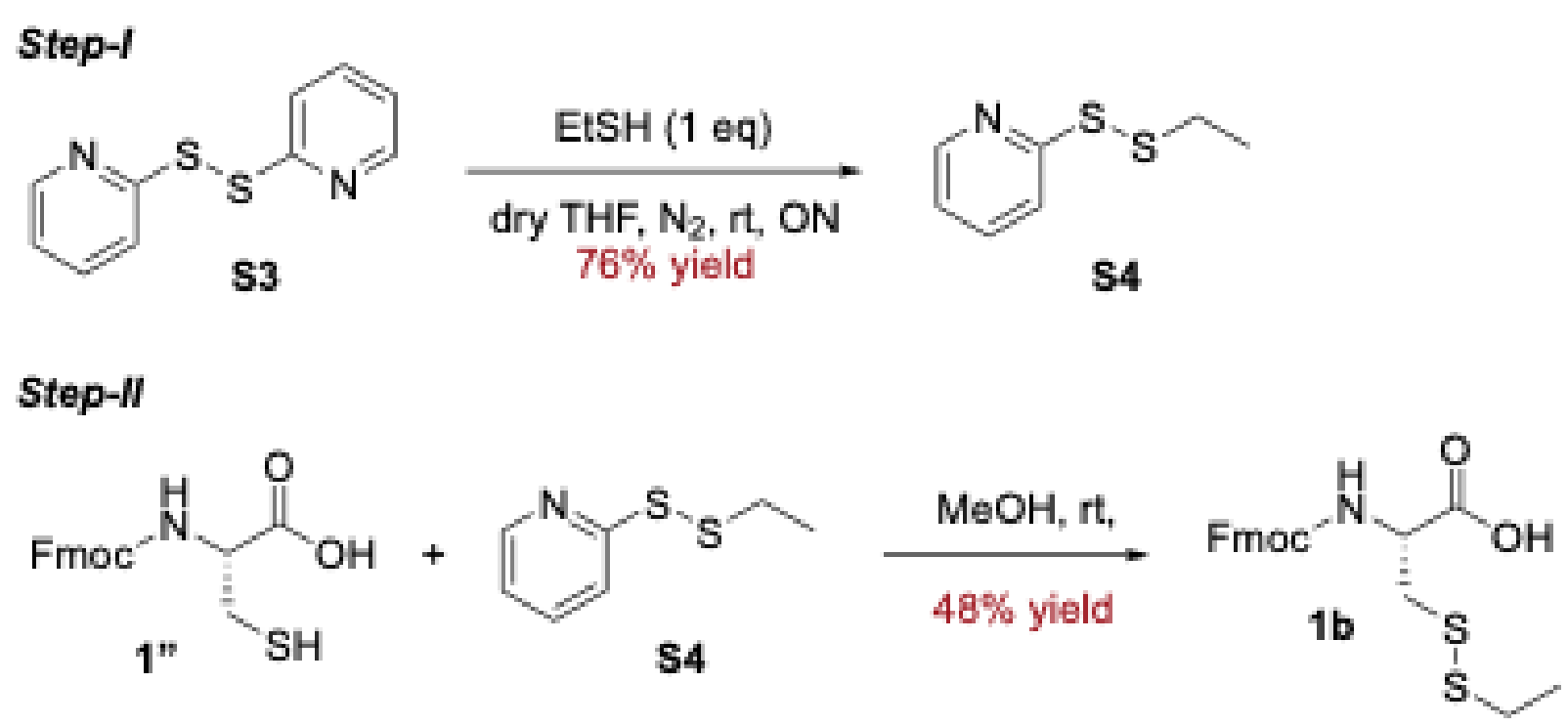

The compound **1b** was synthesized according to the modified literature procedure.

Step-I: *Synthesis of 2-(ethyldisulfaneyl)pyridine:*[2]
In a 100 mL two neck round bottom flask, 1,2-di(pyridin-2-yl)disulfane **S3** (5.4 g, 25 mmol, 1 eq) was added and the flask was evacuated followed by backfilled with nitrogen three times. Anhydrous tetrahydrofuran (THF, 23 mL) was added while stirring under the positive pressure of nitrogen. Then, ethanethiol (EtSH, 1.8 mL, 25 mmol, 1 eq) was added dropwise under N_2 and left the reaction stirred for overnight at room temperature. After completion, the solvent was evaporated and the crude product was purified through silica gel column chromatography using 7% ethyl acetate-hexane as eluent to afford the desired product 2-(ethyldisulfaneyl)pyridine **S4** (3.2 g, yellow liquid, 76% yield). R_f = 0.3 (in 20% ethyl acetate-hexane). **^{1}H NMR (400 MHz, DMSO-*d$_6$*)** δ (ppm): 8.44 – 8.42 (m, 1H), 7.71 (dt, *J* = 8.1, 1.0 Hz, 1H), 7.64 – 7.59 (m, 1H), 7.07 – 7.04 (m, 1H), 2.80 (q, *J* = 7.3 Hz, 2H), 1.31 (t, *J* = 7.3 Hz, 3H).

Step-II: *Synthesis of N-(((9H-fluoren-9-yl)methoxy)carbonyl)-S-(ethylthio)-L-cysteine:*[2]
In a 50 mL round bottom flask, (((9*H*-fluoren-9-yl)methoxy)carbonyl)-*L*-cysteine **1"** (343 mg, 1 mmol, 1 eq) and 2-(ethyldisulfaneyl)pyridine **S4** (340 mg, 2 mmol, 2 eq) were taken and dissolved in methanol (12 mL) while stirring and left the solution stirred for 4h. After complete consumption of **1"**, monitored by TLC, solvent was evaporated and the crude product was purified through silica gel column chromatography using 1% methanol-dichloromethane as eluent to afford *N*-(((9*H*-fluoren-9-yl)methoxy)carbonyl)-*S*-(ethylthio)-*L*-cysteine **1b** (193 mg, pale white solid, 48% yield). R_f = 0.2 (in 10% methanol-dichloromethane). **^{1}H NMR (400 MHz, DMSO-*d$_6$*)** δ (ppm): 12.95 (s, 1H), 7.89 (d, *J* = 7.5 Hz, 2H), 7.80 (d, *J* = 8.4 Hz, 1H), 7.72 (d, *J* = 7.5 Hz, 2H), 7.42 (t, *J* = 7.0 Hz, 2H), 7.33 (t, *J* = 7.5 Hz, 2H), 4.36 (m, 4H), 3.12 (dd, *J* = 13.6, 4.1 Hz, 1H), 2.92 (dd, *J* = 13.6, 10.2 Hz, 1H), 2.71 (q, *J* = 7.3 Hz, 2H), 1.22 (t, *J* = 7.3

Hz, 3H). **^{13}C {^{1}H} NMR (101 MHz, DMSO-*d$_6$*)** δ (ppm): 172.2, 156.0, 143.8, 140.7, 127.6, 127.1, 125.2, 120.1, 65.7, 53.1, 46.6, 31.5, 14.3.

VIII. Table S1. Screening of Reducing Reagents for Disulfide Reduction on Compound *N*-(((9*H*-fluoren-9-yl)methoxy)carbonyl)-*S*-((3-(methoxycarbonyl)-4-nitrophenyl)thio)-*L*-cysteine 1a.

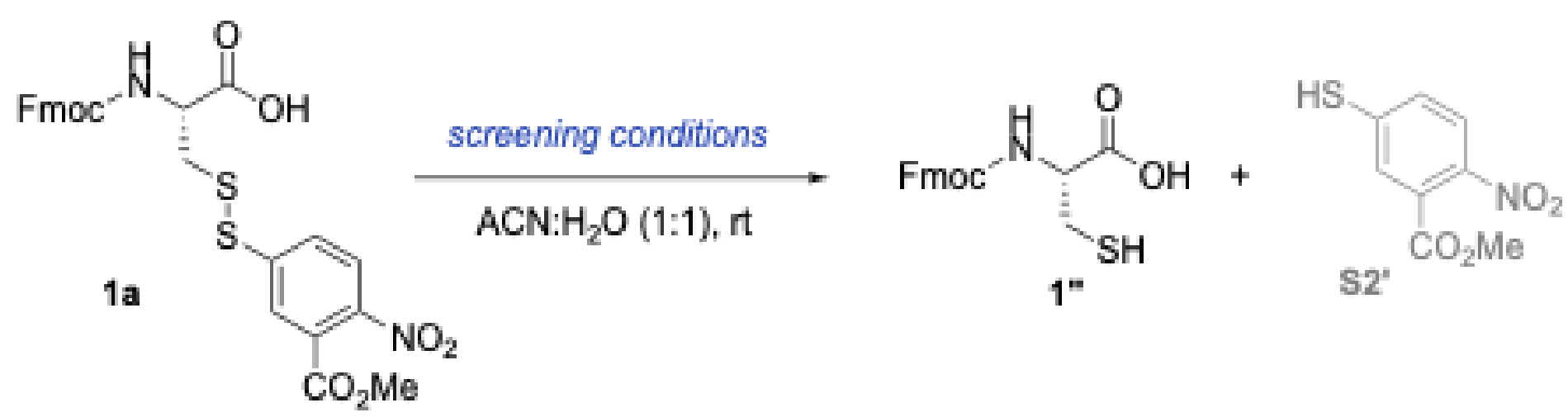

Entry	Reducing reagent (eq)	Base (% of volume)	Time (h)	Conversion **1''** (%)
1	TCEP (10)	-	0.5	100
2	DTT (10)	-	0.5	89
3	TSPP (10)	-	0.5	50
4	TCEP (10)	DIPEA (2.5)	0.5	100
5	DTT (10)	DIPEA (2.5)	0.5	100
6	TSPP (10)	-	1	60
7	TSPP (10)	-	1.5	85
8	DTT (10)	-	1	88
9	DTT (10)	-	1.5	76
10[a]	TCEP (10)	-	0.5	100
11[a]	DTT (10)	-	0.5	80

a = solvent used DMF:H_2O (1:1) and the solution concentration is 4.63 mM

The amino acid *N-(((9H-fluoren-9-yl)methoxy)carbonyl)-S-((4-(methoxycarbonyl)-3-nitrophenyl)thio)-L-cysteine* **1a** (2.54 mg, 0.0046 mmol, 1 eq), reducing reagents (0.046 mmol, 10 eq), DIPEA (37 µL, 2.5% of solvent volume, if required) were taken to a 1 dram vial equipped with a magnetic stirrer and dissolved in solvent mixture (ACN:H_2O = 1:1, 1 mL) for screening under the conditions listed in **Table 1**. The resulting crude mixture was analyzed via **Analytical HPLC Method** (C18, Water (A)/ACN (B); gradient: 5 to 85% B (0.1% formic acid in ACN) in 20 min, flow rate = 1.0 mL/min, I = 220 nm) to determine the conversion. Under these conditions, the starting amino acid disulfide **1a** and the product (((9*H*-fluoren-9-yl)methoxy)carbonyl)-*L*-cysteine **1''** exhibited retention times of 19.46 min and 16.31 min, respectively.

Analytical HPLC traces of reaction mixtures

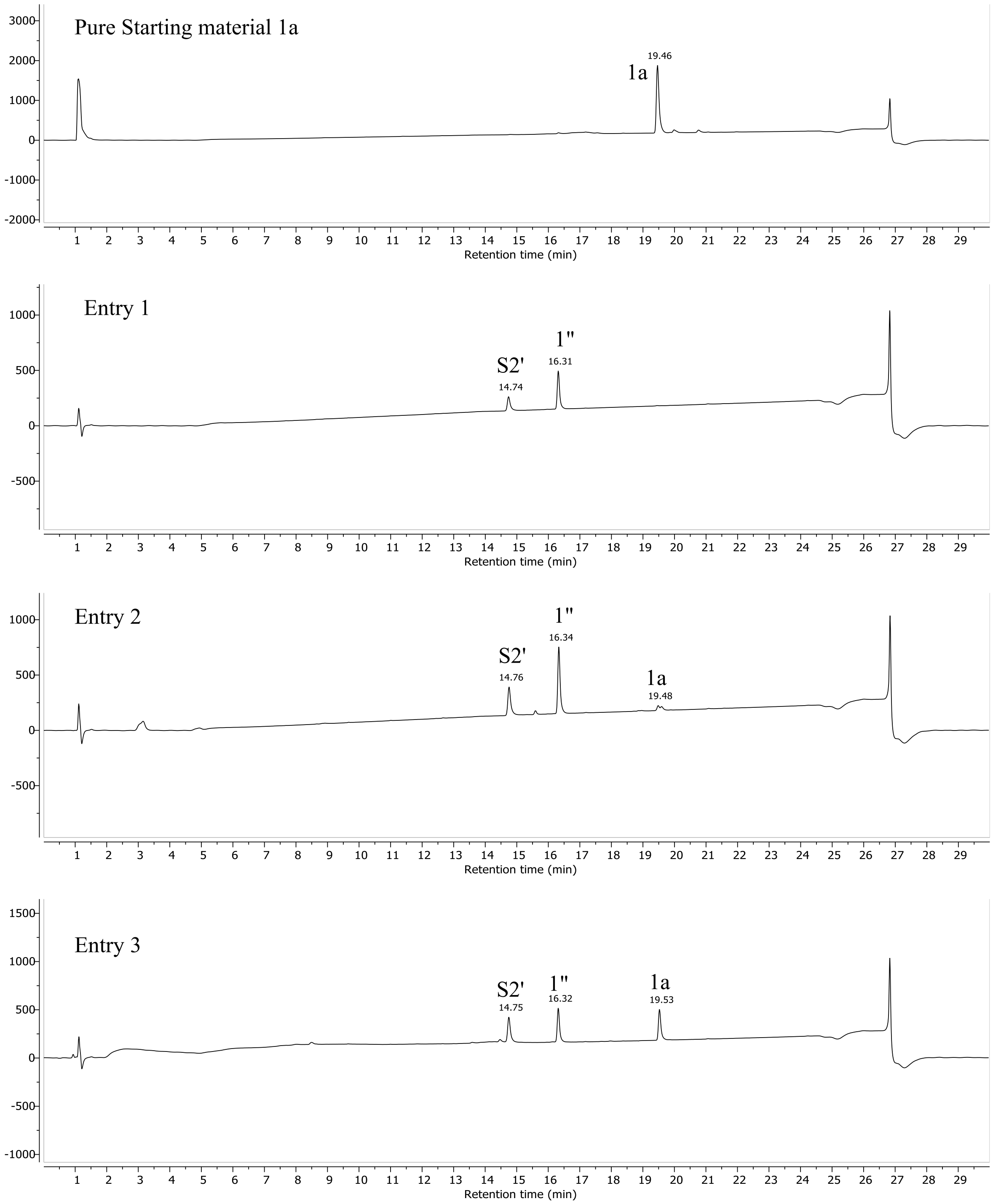

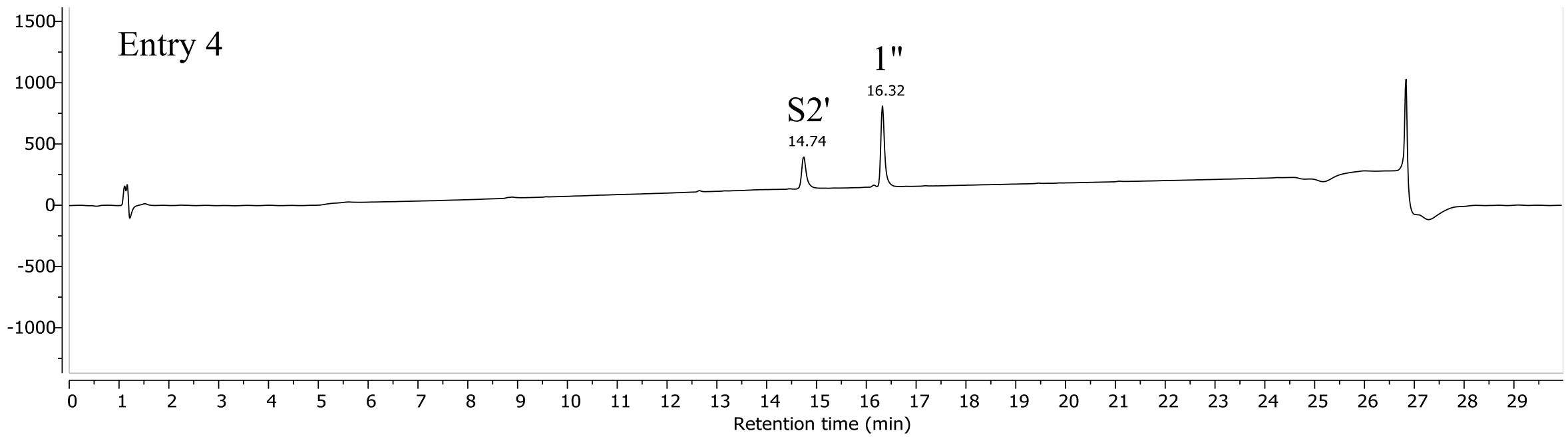
Entry 4
S2'
14.74
1"
16.32
Retention time (min)

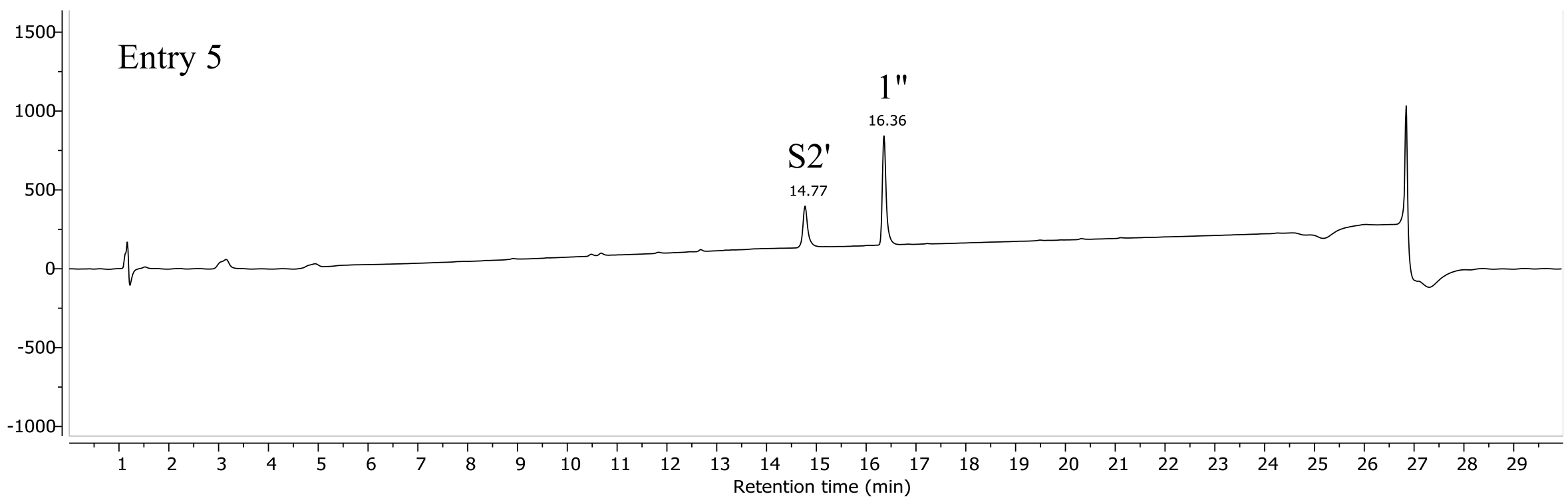
Entry 5
S2'
14.77
1"
16.36
Retention time (min)

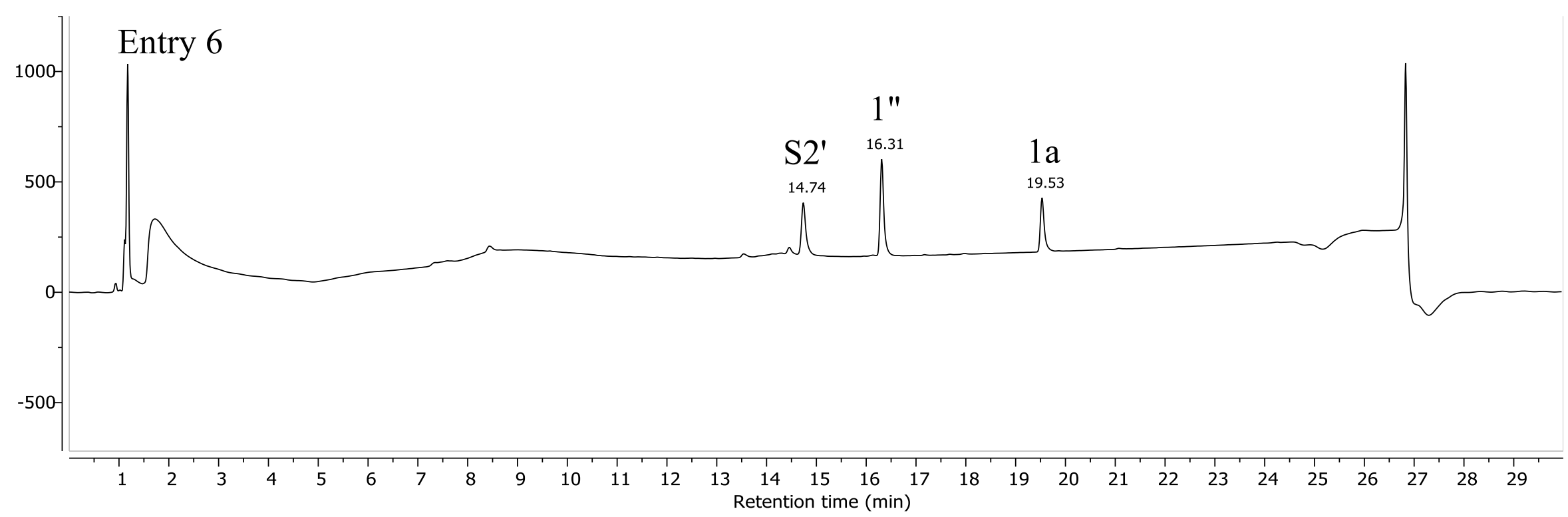
Entry 6
S2'
14.74
1"
16.31
1a
19.53
Retention time (min)

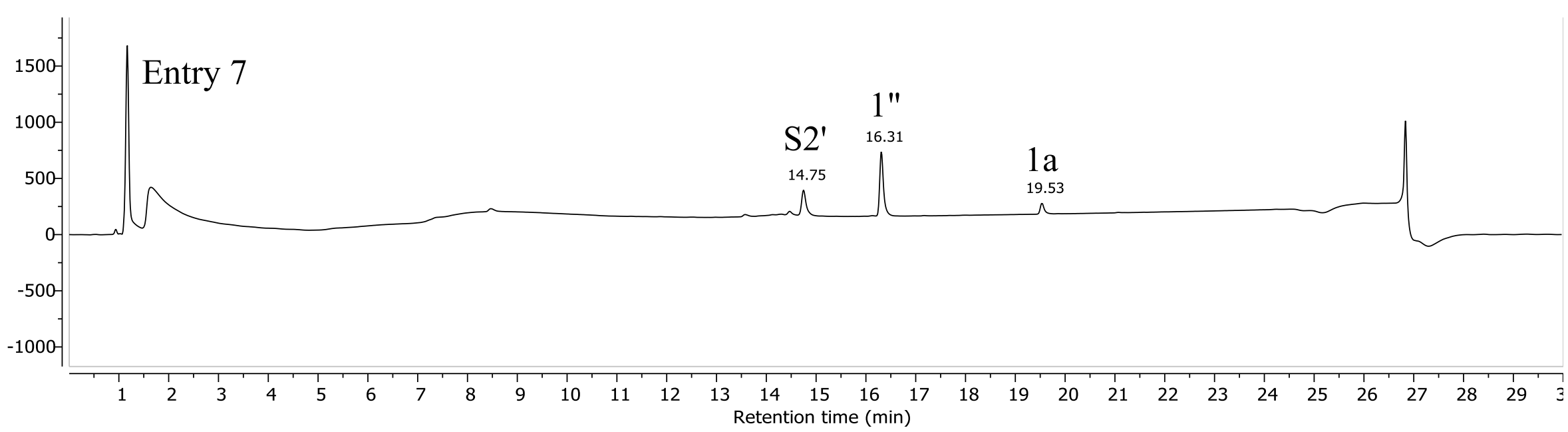
Entry 7
S2'
14.75
1"
16.31
1a
19.53
Retention time (min)

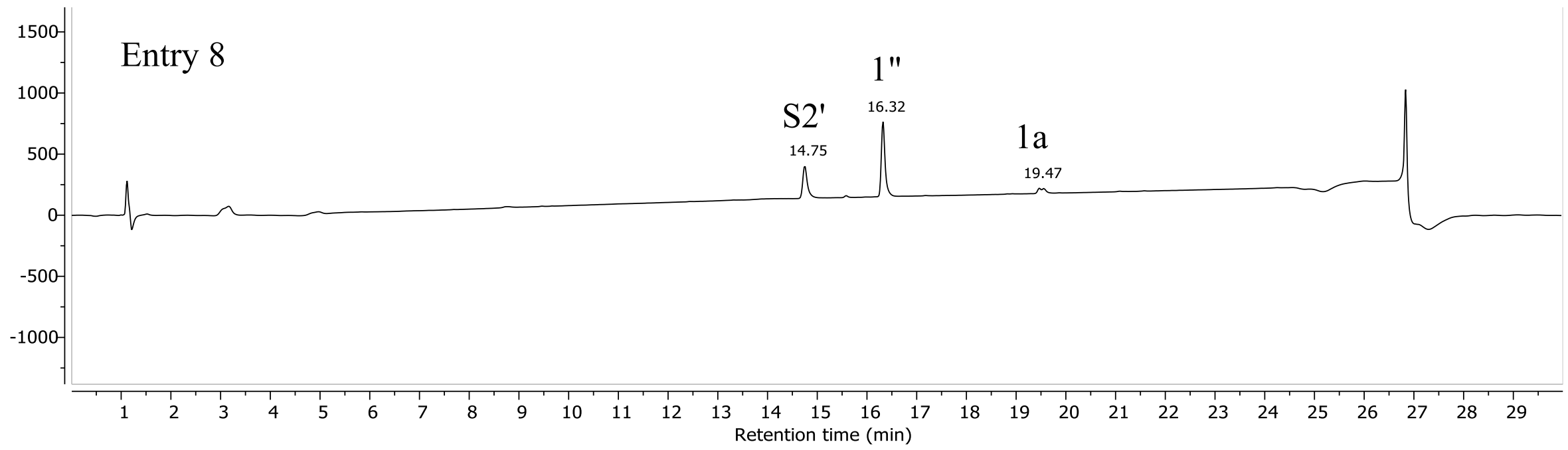
Entry 8
S2'
14.75
1"
16.32
1a
19.47
Retention time (min)

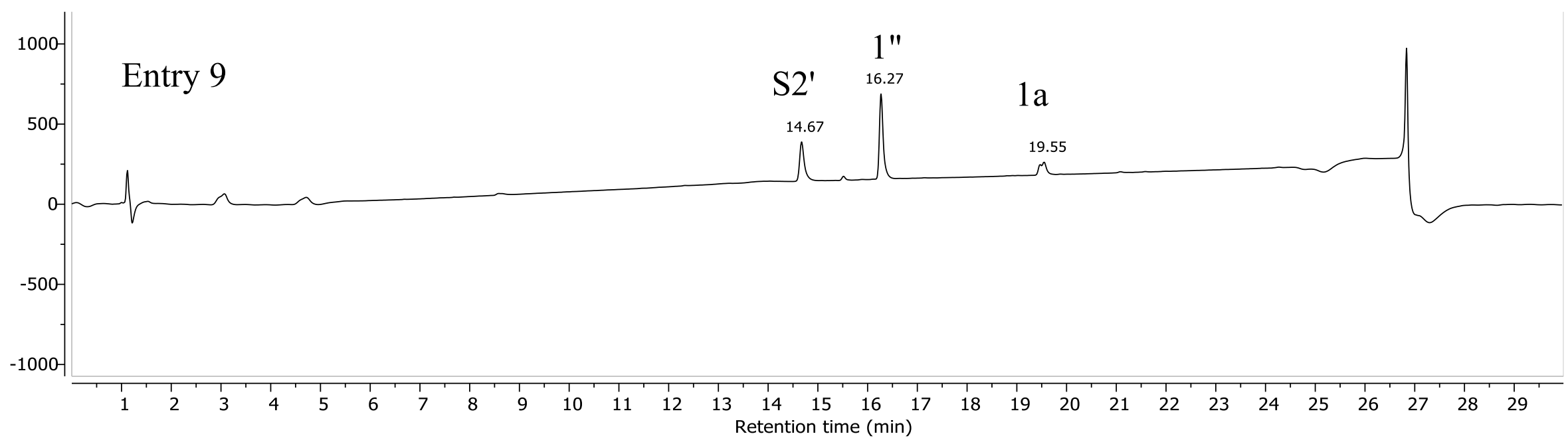
Entry 9
S2'
14.67
1"
16.27
1a
19.55
Retention time (min)

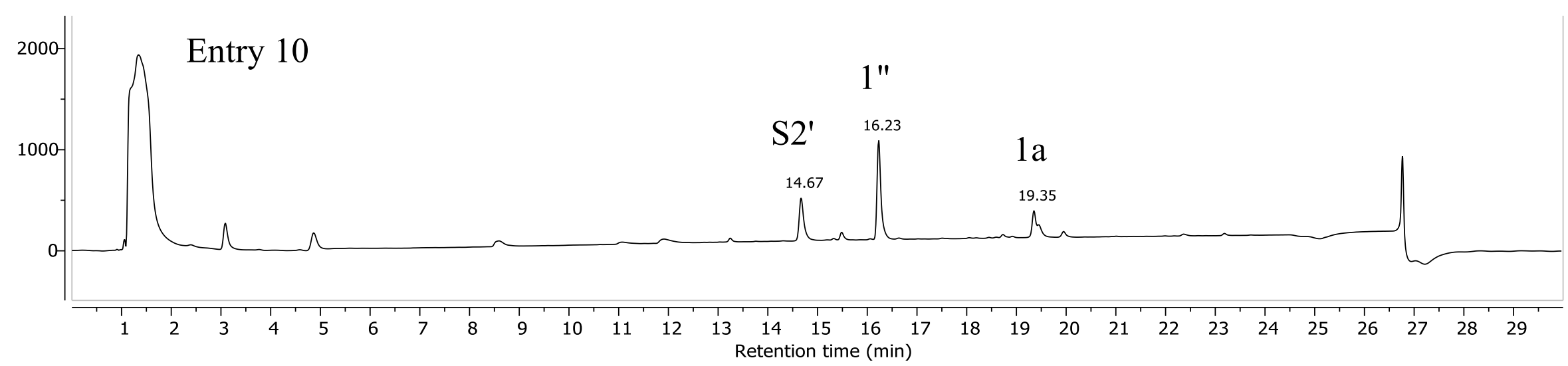
Entry 10
S2'
14.67
1"
16.23
1a
19.35
Retention time (min)

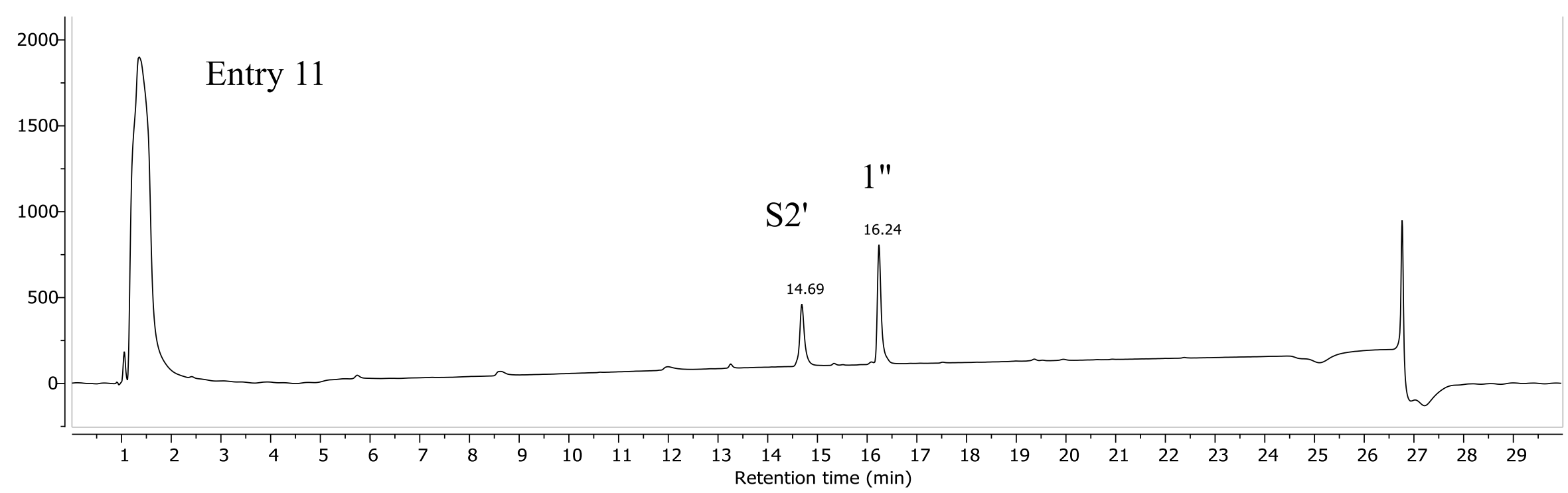
Entry 11
S2'
14.69
1"
16.24
Retention time (min)

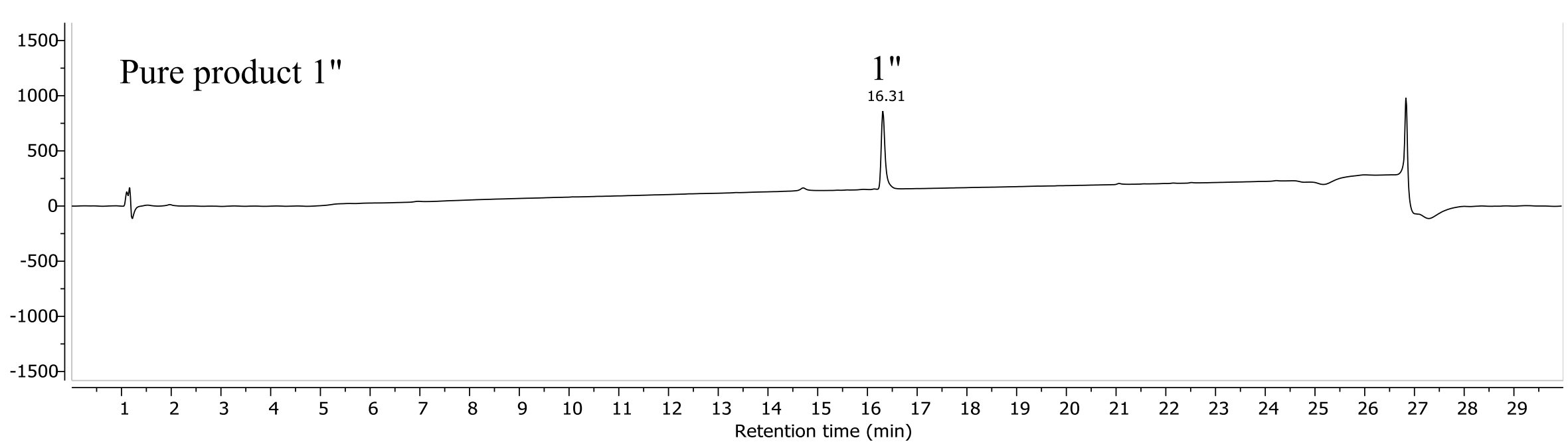
Pure product 1"
1"
16.31
Retention time (min)

IX. Table S2. Screening of Reducing Reagents for Disulfide Reduction on Compound *N*-(((9*H*-fluoren-9-yl)methoxy)carbonyl)-*S*-(ethylthio)-*L*-cysteine 1b.

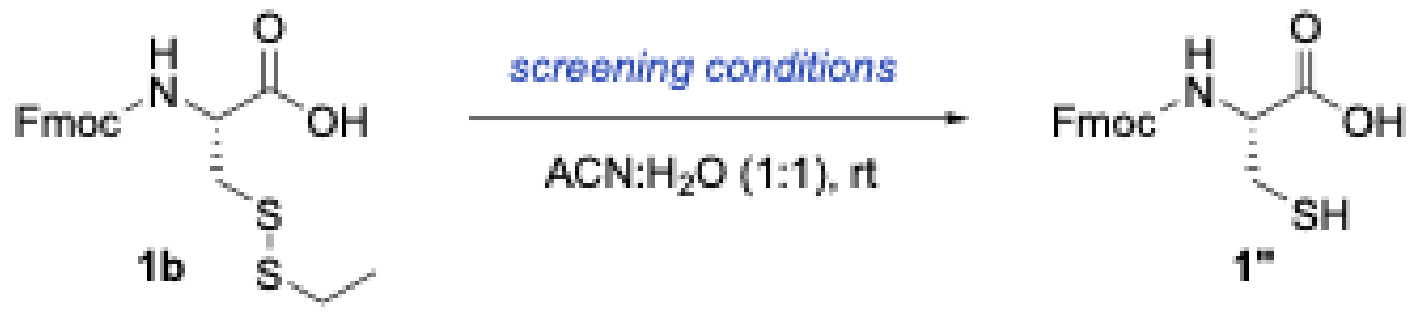

Entry	Reducing reagent (eq)	Base (% of volume)	Time (h)	Conversion **1"** (%)
1	TCEP (10)	-	1	100
2	DTT (10)	-	1	0
3	TSPP (10)	-	1	71
4	TCEP (10)	DIPEA (2.5)	1	100
5	DTT (10)	DIPEA (2.5)	1	100
6[a]	TCEP (10)	-	1	100

a = solvent used DMF:H_2O (1:1) and the solution concentration is 4.63 mM

The amino acid *N-(((9H-fluoren-9-yl)methoxy)carbonyl)-S-(ethylthio)-L-cysteine* **1b** (1.85 mg, 0.0046 mmol, 1 eq), reducing reagents (0.046 mmol, 10 eq), DIPEA (37 µL, 2.5% of solvent volume, if required) were taken to a 1 dram vial equipped with a magnetic stirrer and dissolved in solvent mixture (ACN:H_2O = 1:1, 1 mL) for screening under the conditions listed in **Table 2**. The resulting crude mixture was analyzed via **Analytical HPLC Method** (C18, Water (A)/ACN (B); gradient: 5 to 85% B (0.1% formic acid in ACN) in 20 min, flow rate = 1.0 mL/min, I = 220 nm) to determine the conversion. Under these conditions, the starting amino acid disulfide **1b** and the product (((9*H*-fluoren-9-yl)methoxy)carbonyl)-*L*-cysteine **1"** exhibited retention times of 18.5 min and 16.31 min, respectively.

Analytical HPLC traces of reaction mixtures

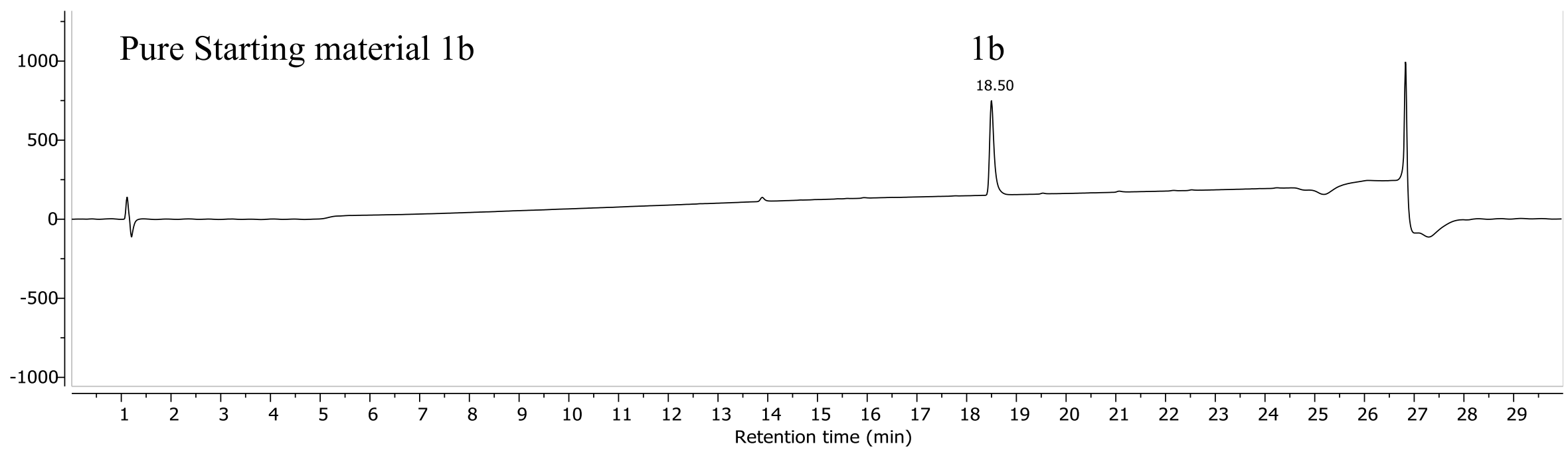

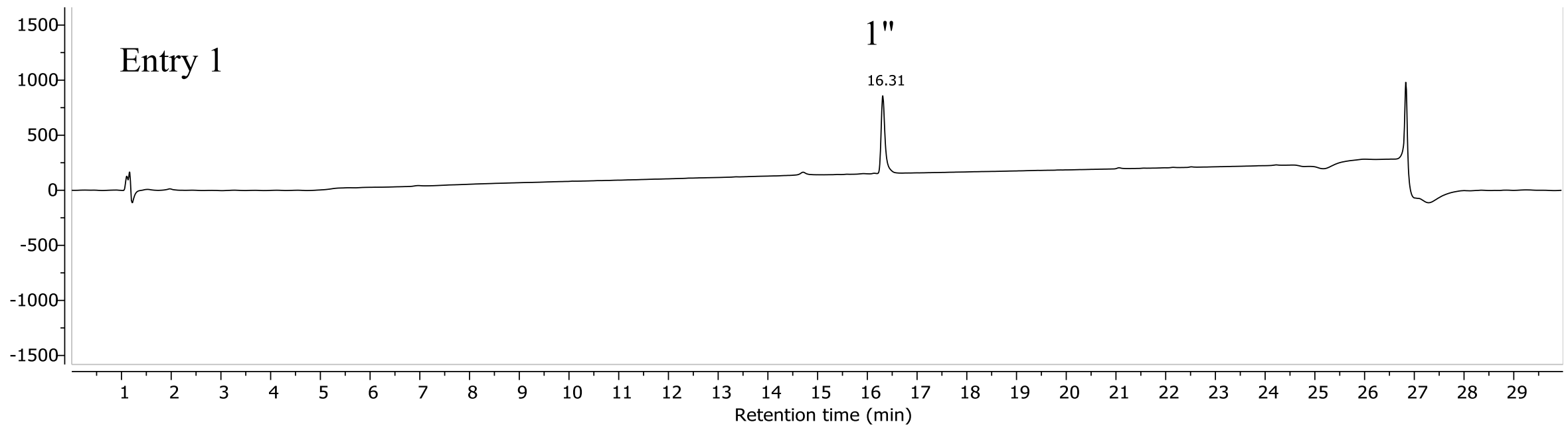
Entry 1
1"
16.31
Retention time (min)

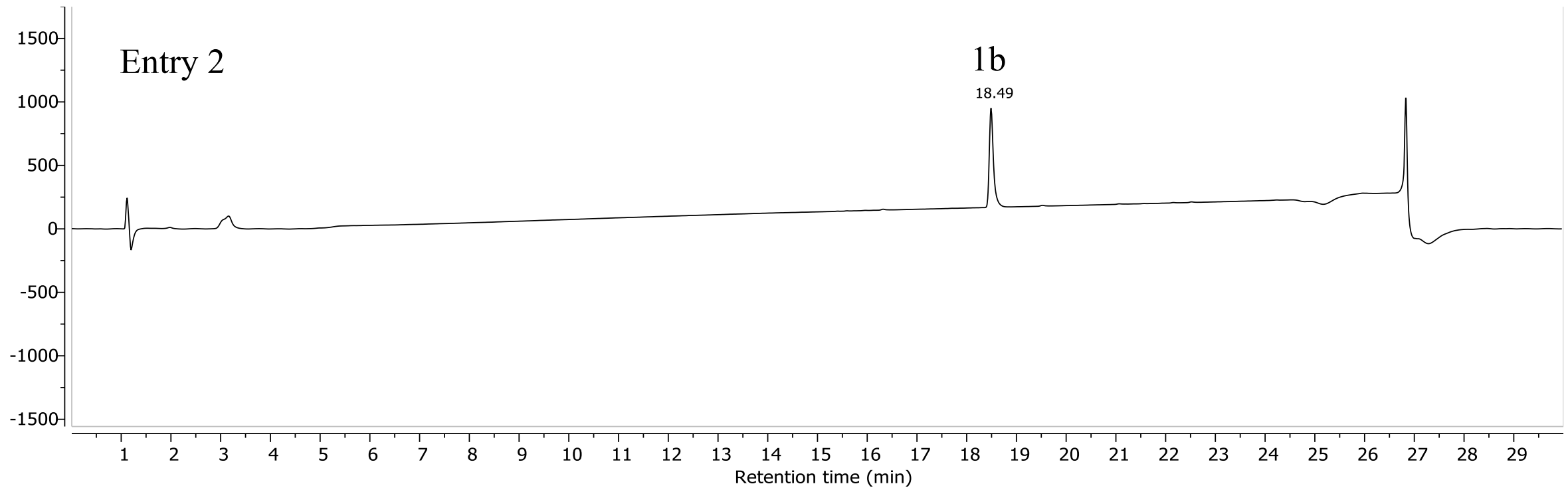
Entry 2
1b
18.49
Retention time (min)

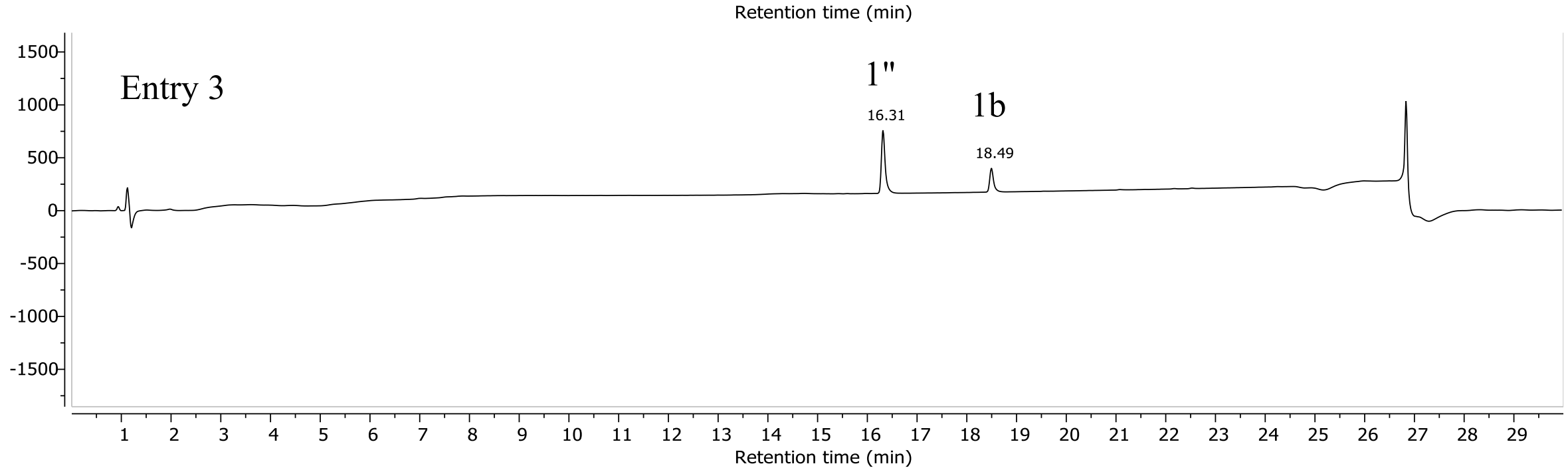
Entry 3
1"
16.31
1b
18.49
Retention time (min)

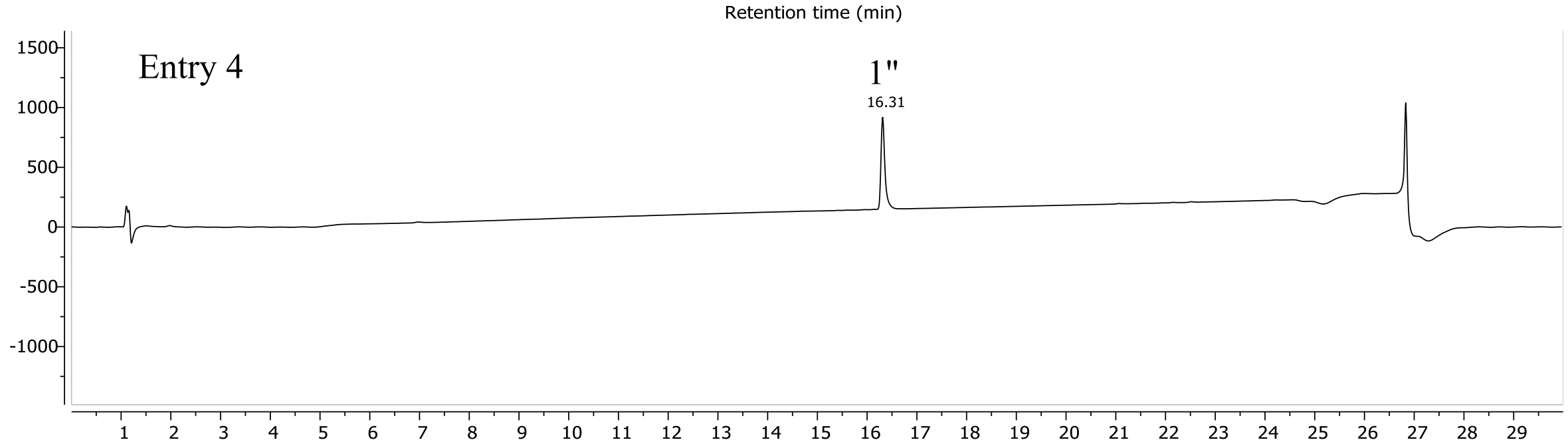
Entry 4
1"
16.31
Retention time (min)

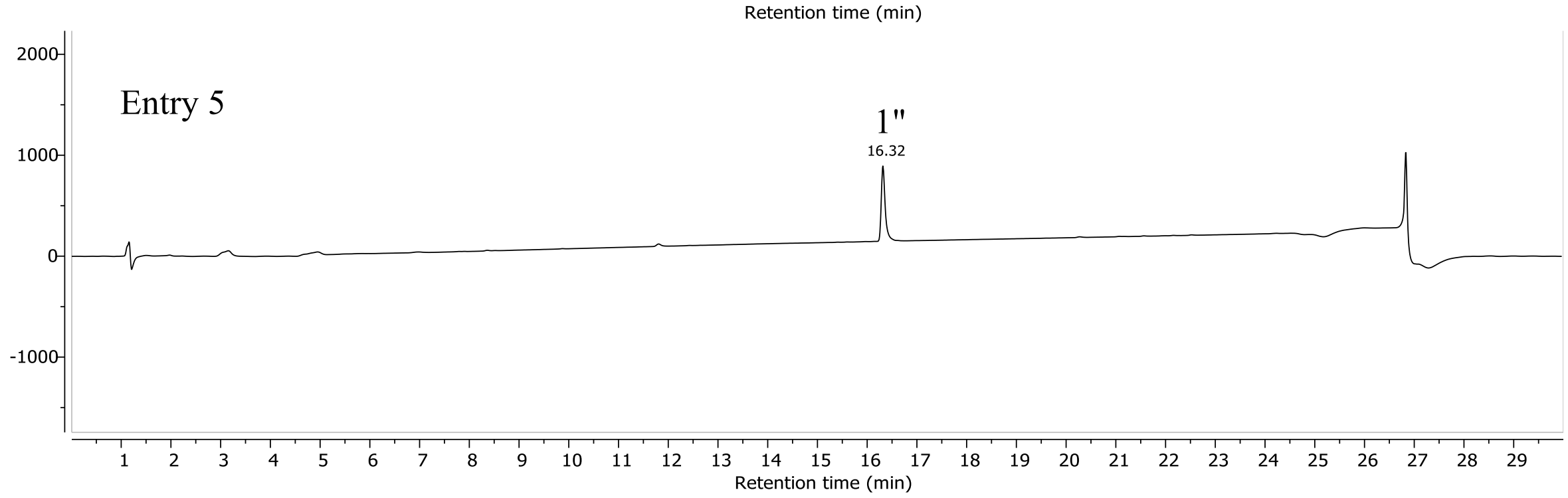
Entry 5
1"
16.32
Retention time (min)

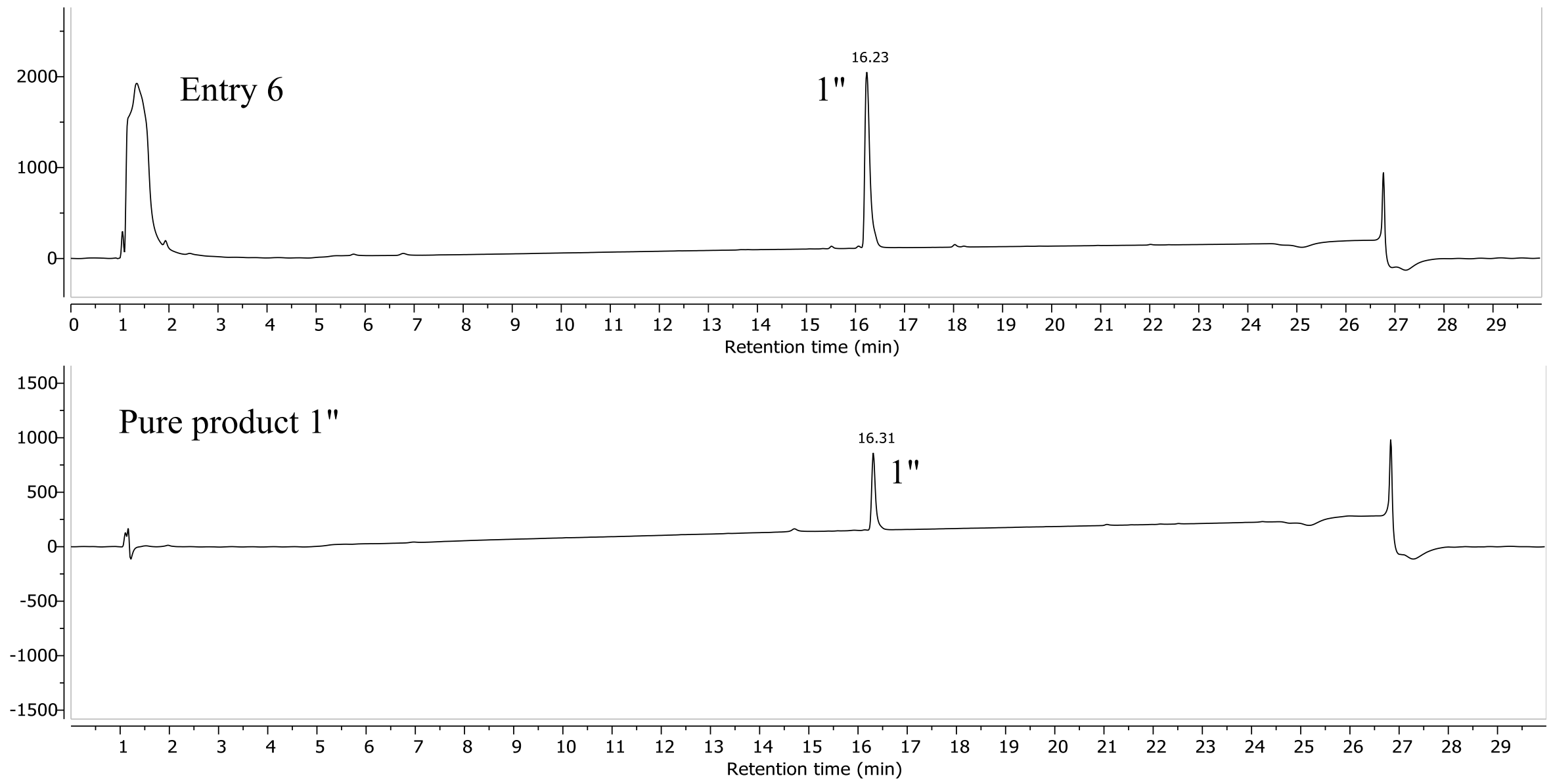

X. Table S3. Screening of Reducing Reagents for Disulfide Reduction on Compound *N*-(((9*H*-fluoren-9-yl)methoxy)carbonyl)-*S*-(*tert*-butylthio)-*L*-cysteine 1c.

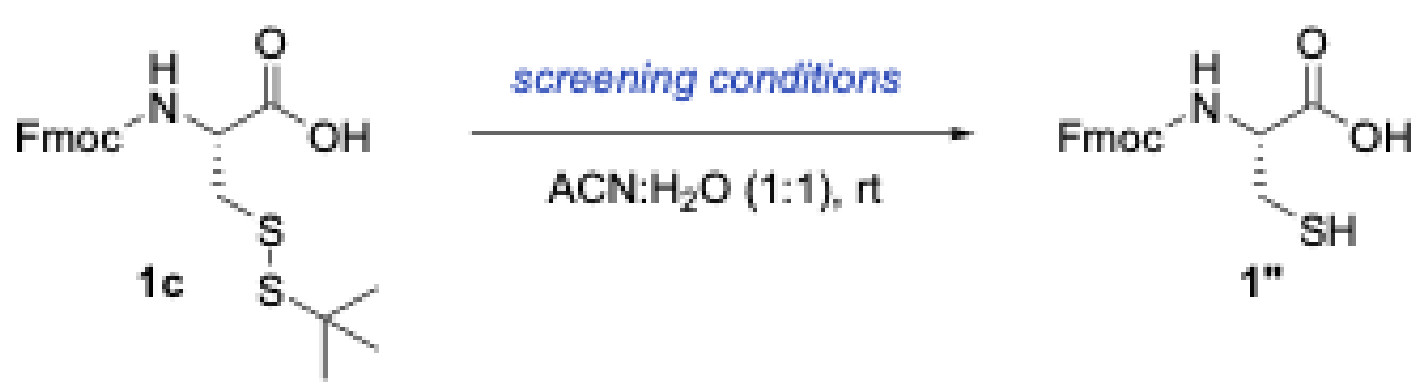

Entry	Reducing reagent (eq)	Base (% of volume)	Time (h)	Conversion **1"** (%)
1	TCEP (10)	-	1	0
2	DTT (10)	-	1	0
3	TSPP (10)	-	1	0
4	TCEP (10)	DIPEA (2.5)	1	64
5	DTT (10)	DIPEA (2.5)	1	86
6	DTT (10)	DIPEA (2.5)	1.5	100
7[a]	TCEP (10)	DIPEA (2.5)	1	74
8[a]	DTT (10)	DIPEA (2.5)	1.5	100
9[a]	DTT (10)	-		0
10[b]	DTT (10)	DIPEA (2.5)	1.5	100
11[c]	DTT (10)	DIPEA (2.5)	1.5	84

a = solvent used DMF:H_2O (1:1) and the solution concentration is 4.63 mM; *b* = solvent used ACN:H_2O (1:1) and concentration is 2.32 mM; *c* = solvent used ACN:H_2O (1:1) and concentration is 1.16 mM

The amino acid *N*-(((9*H*-fluoren-9-yl)methoxy)carbonyl)-*S*-(*tert*-butylthio)-*L*-cysteine **1c** (2 mg, 0.0046 mmol, 1 eq), reducing reagents (0.046 mmol, 10 eq), DIPEA (37 µL, 2.5% of solvent volume, if required) were taken to a 1 dram vial equipped with a magnetic stirrer and dissolved in solvent mixture (ACN:H_2O = 1:1, 1 mL) for screening under the conditions listed in **Table 3**. The resulting crude mixture was analyzed via **Analytical HPLC Method** (C18, Water (A)/ACN (B); gradient: 5 to 85% B (0.1% formic acid in ACN) in 20 min, flow rate = 1.0 mL/min, I = 220 nm) to determine the conversion. Under these conditions, the starting amino acid disulfide **1c** and the product (((9*H*-fluoren-9-yl)methoxy)carbonyl)-*L*-cysteine **1"** exhibited retention times of 19.86 min and 16.31 min, respectively.

<u>*Analytical HPLC traces of reaction mixtures*</u>

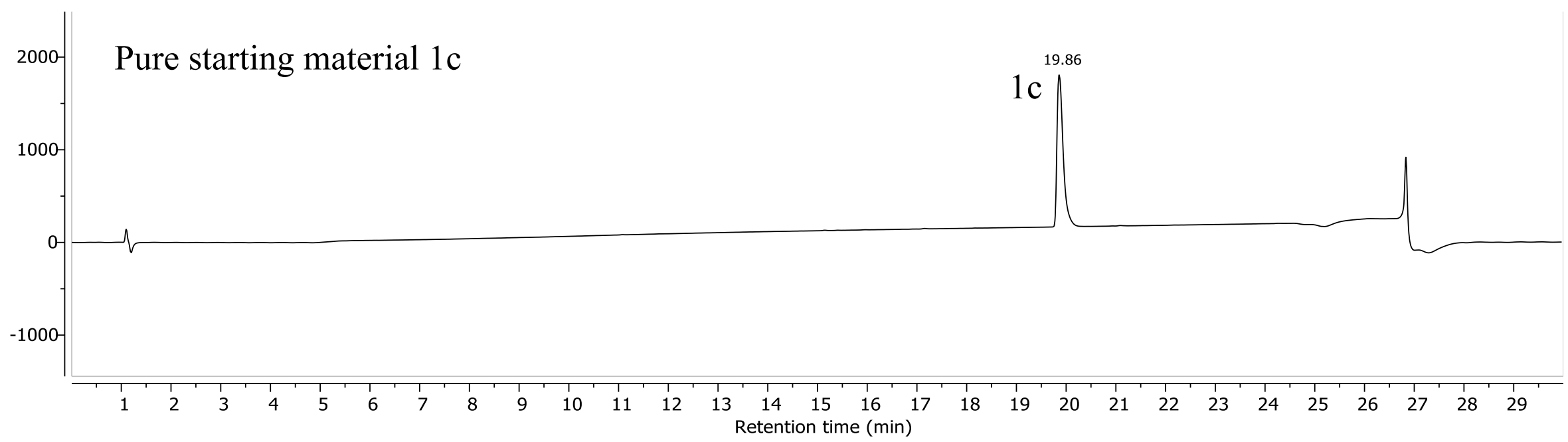

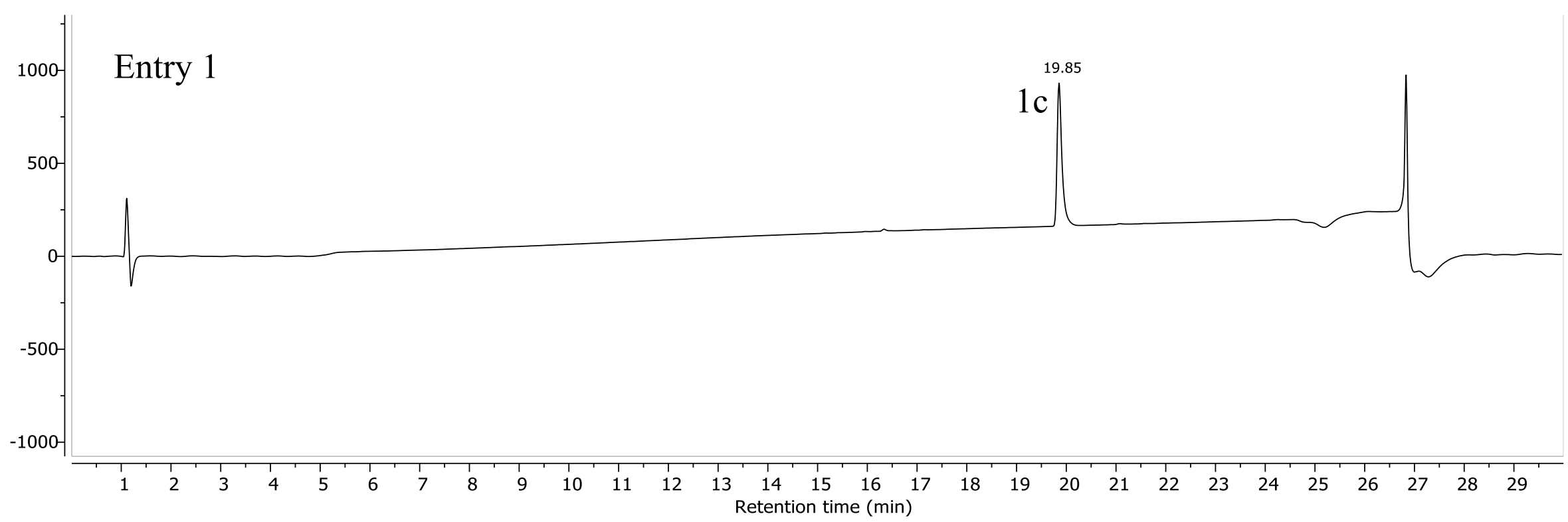

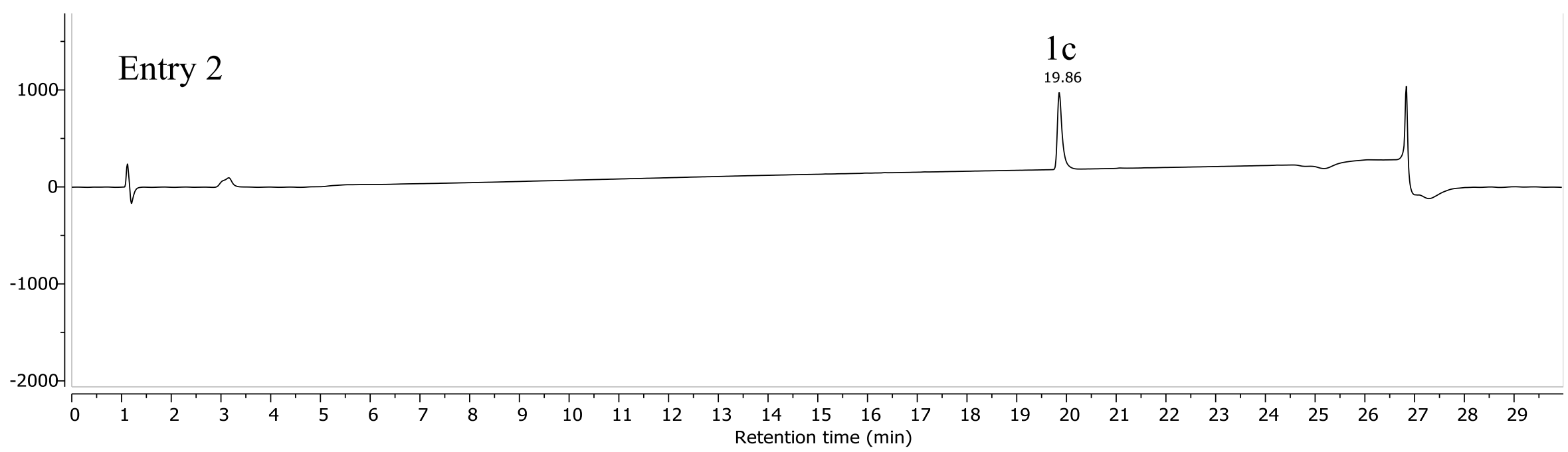

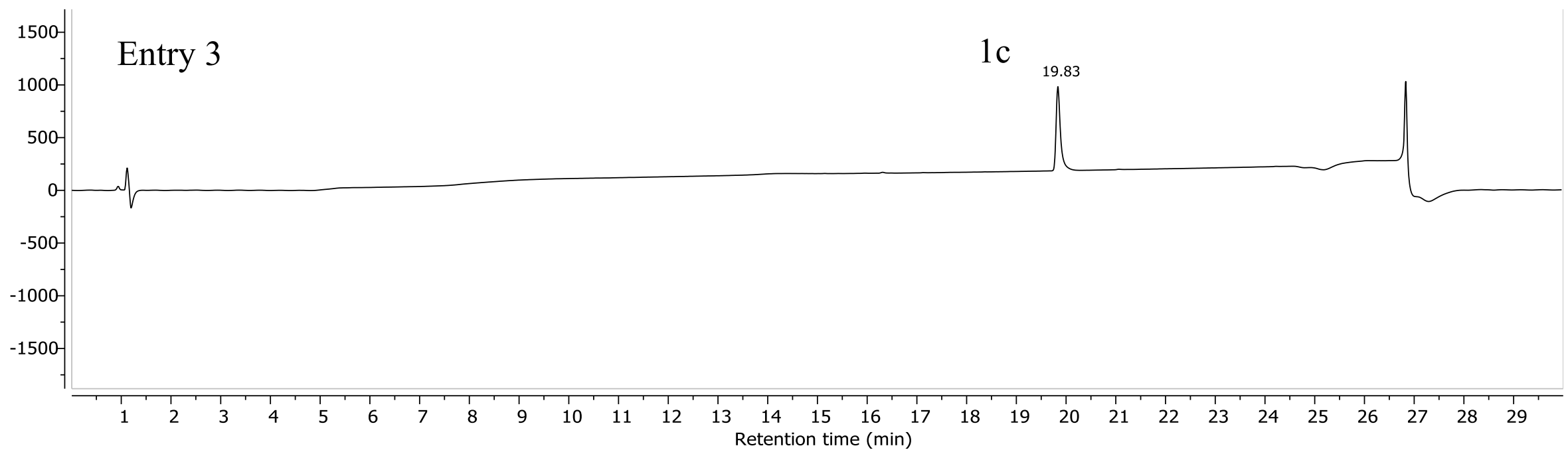
Entry 3
1c
19.83
Retention time (min)

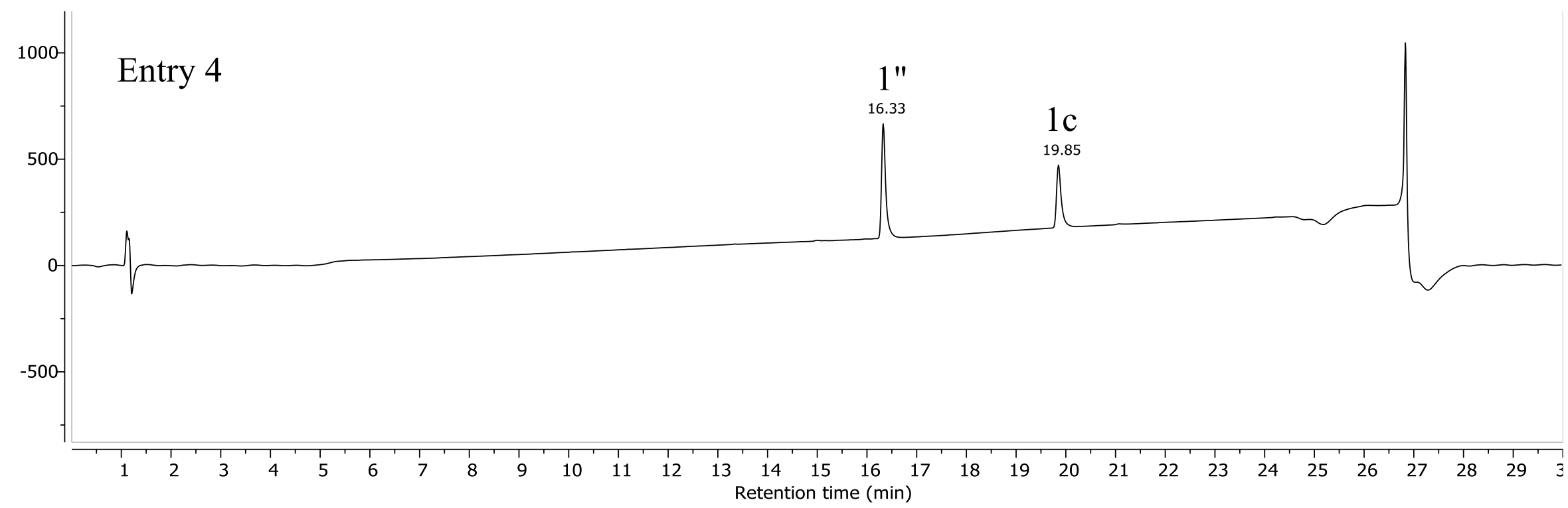
Entry 4
1"
16.33
1c
19.85
Retention time (min)

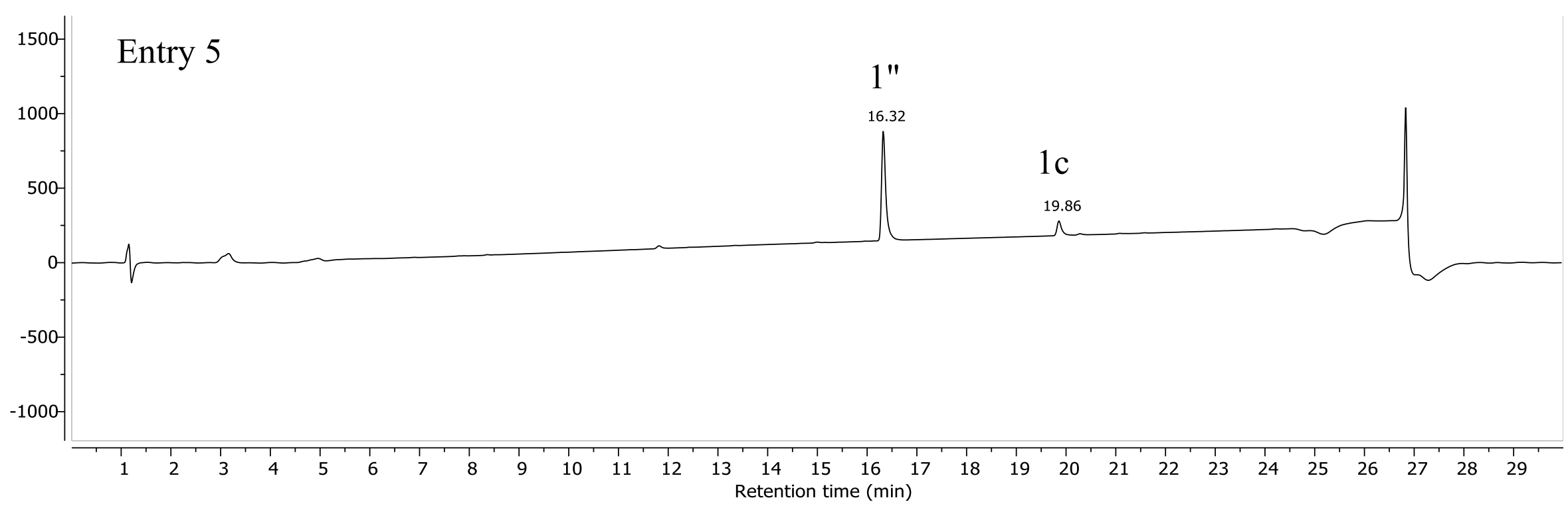
Entry 5
1"
16.32
1c
19.86
Retention time (min)

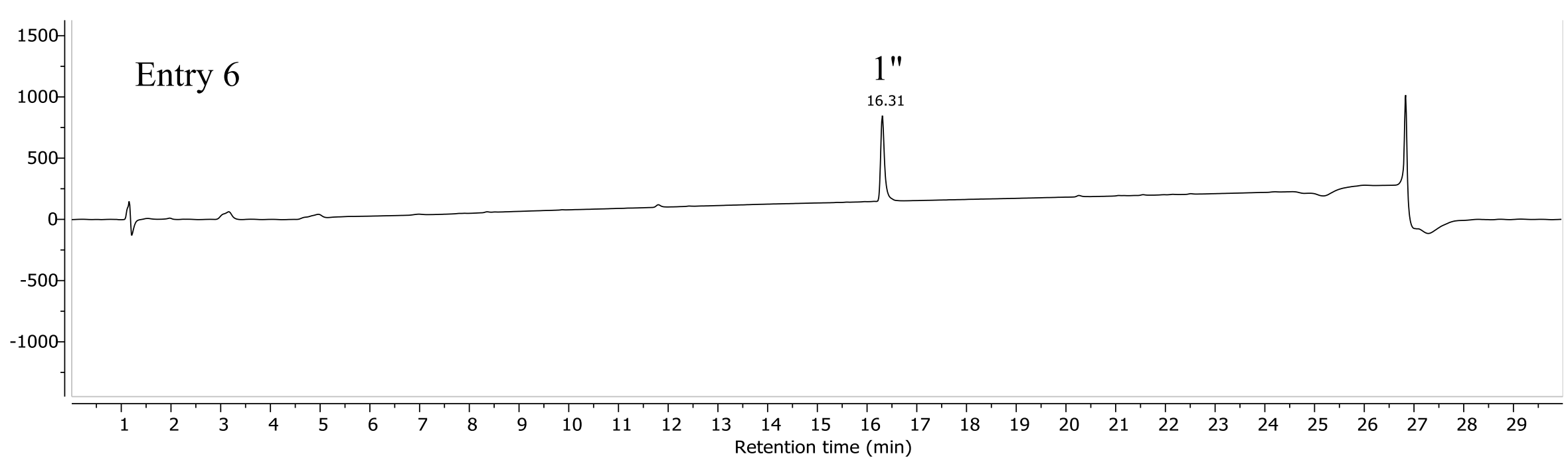
Entry 6
1"
16.31
Retention time (min)

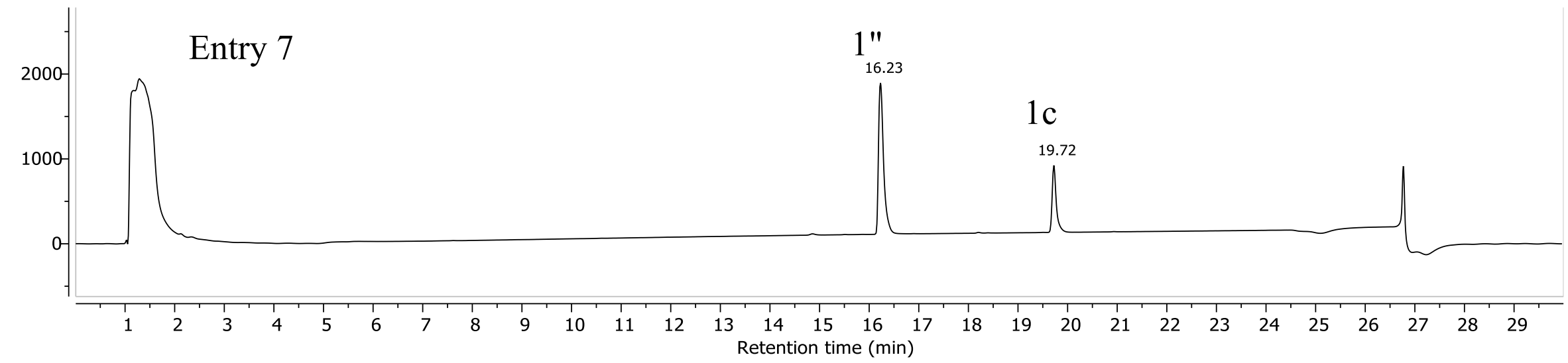
Entry 7
1"
16.23
1c
19.72
Retention time (min)

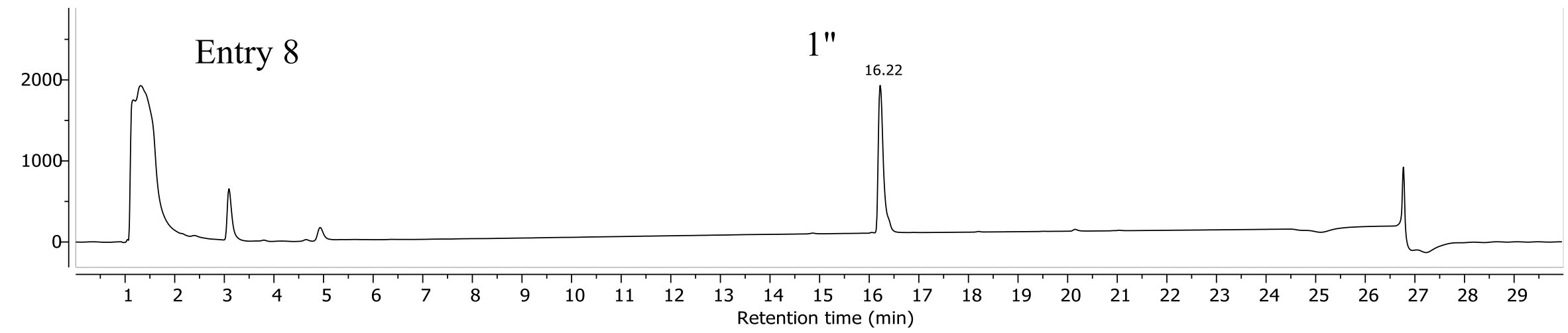
Entry 8
1"
16.22
Retention time (min)

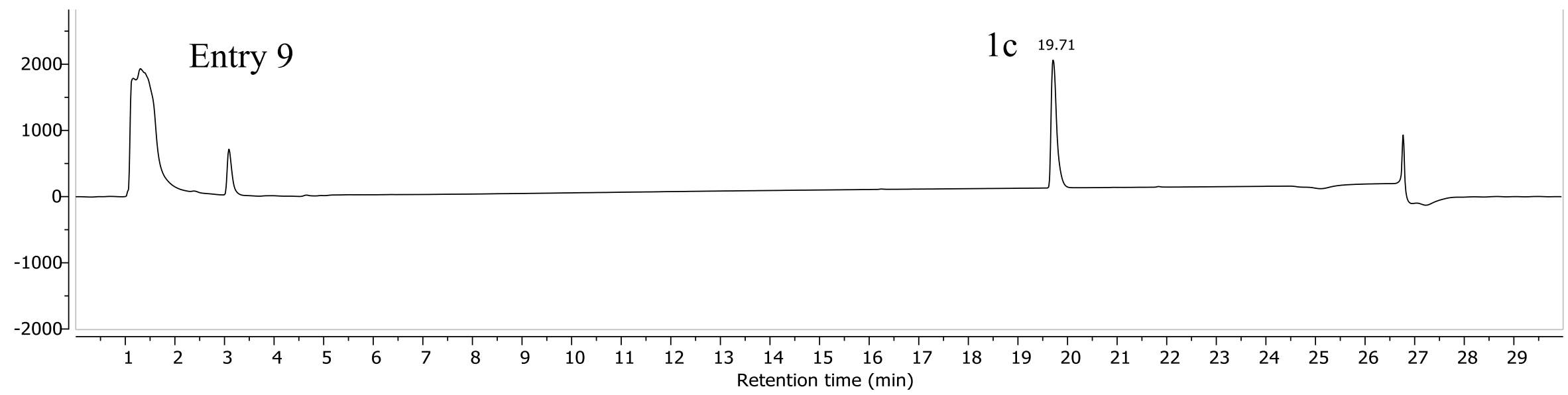
Entry 9
1c
19.71
Retention time (min)

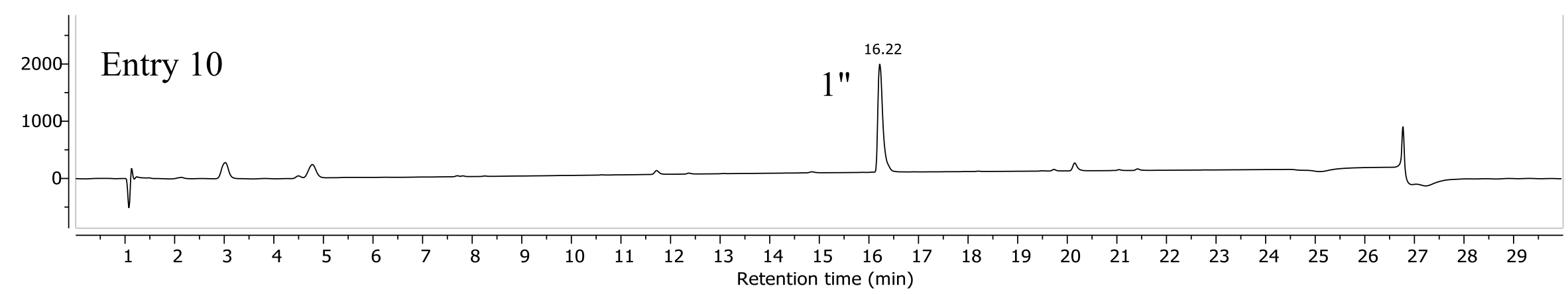
Entry 10
16.22
1"
Retention time (min)

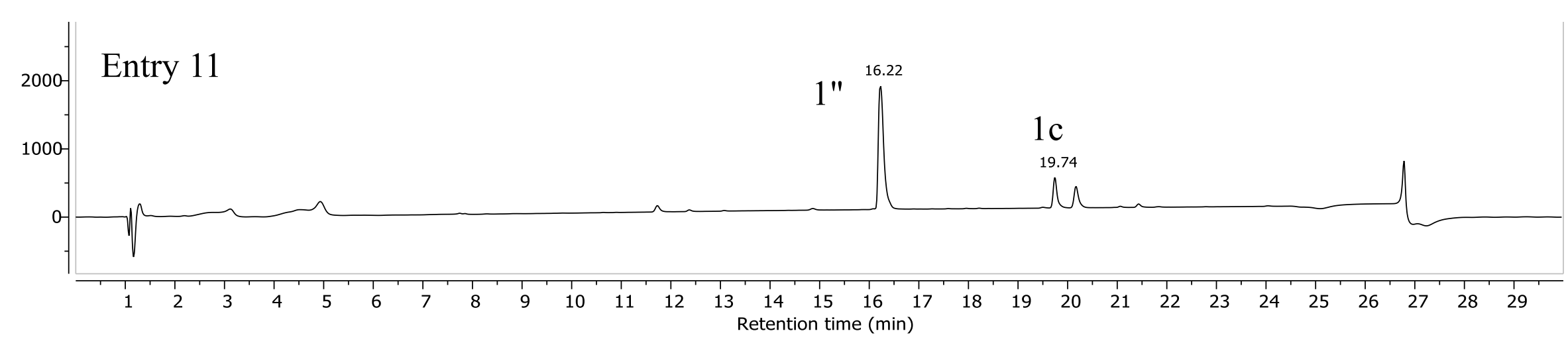
Entry 11
16.22
1"
1c
19.74
Retention time (min)

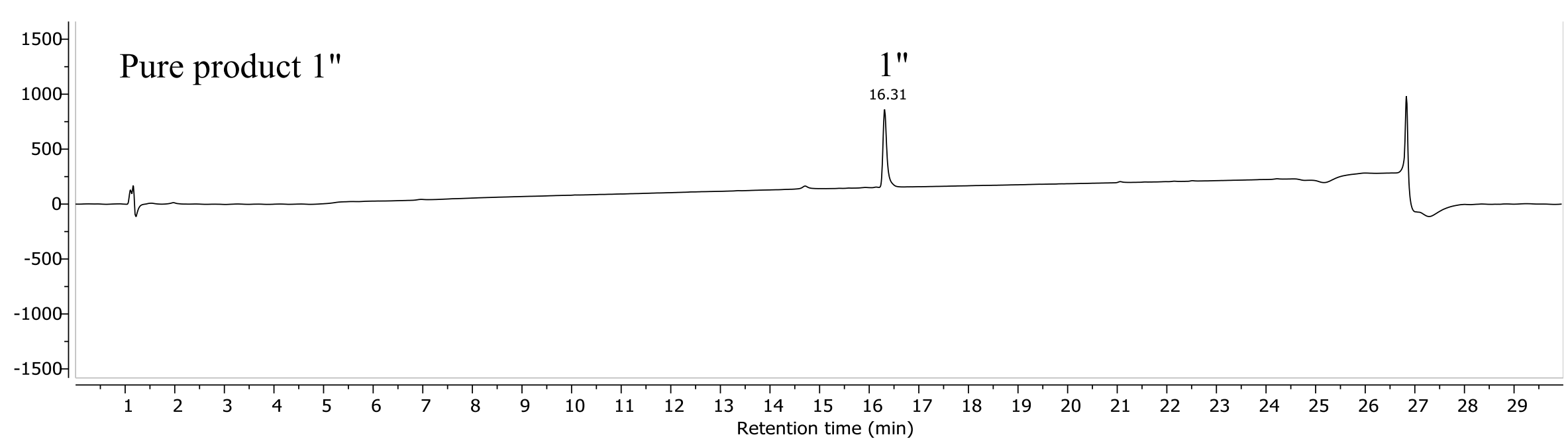
Pure product 1"
1"
16.31
Retention time (min)

XI.1. Supplementary Figure 1. Synthesis of Aryl Disulfide in Cys-Containing Peptide Sequence.

XI.1.1. Synthesis of Peptide Ac-C(SAr^2)WC(S^tBu)AR-$CONH_2$ **1d**:

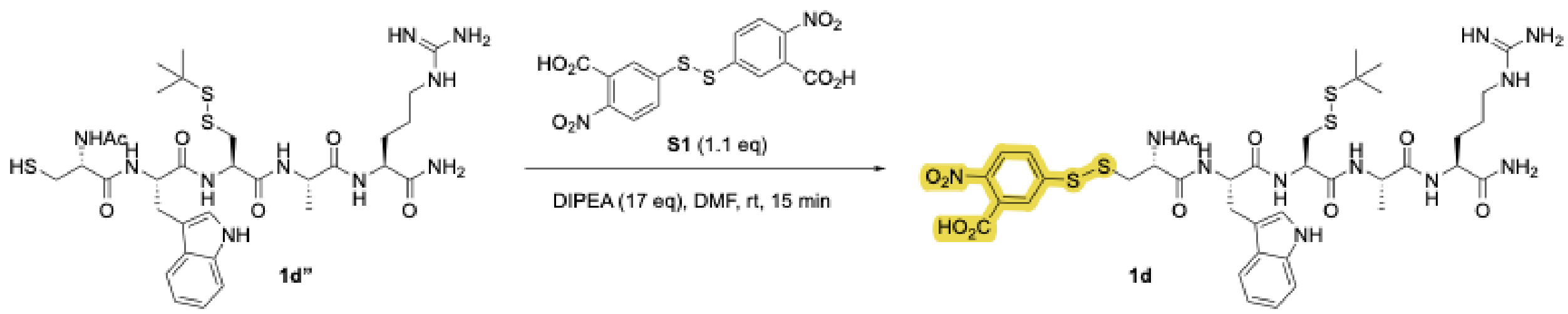

In a 1 dram vial equipped with a magnetic stirrer was added the purified peptide Ac-CWC(S^tBu)AR-$CONH_2$ **1d"** (3.1 mg, 0.0041 mmol, 1 eq), *N,N*-diisopropylethylamine (DIPEA, 12 µL, 0.69 mmol, 17 eq) and *N,N*-dimethylformamide (DMF, 700 µL), while stirring. In a separate 1 dram vial, 4,4'-disulfanediylbis-2-nitrobenzoic acid **S1** (1.8 mg, 0.0045 mmol, 1.1 eq) was taken and dissolved in DMF (300 µL) and the solution was added dropwise to the peptide solution and resulting mixture was allowed to stir 15 min at room temperature. After that, conversion of the aryl-disulfide containing peptide **1d** was determined via analytical HPLC using 0.1% formic acid (FA) in both mobile phases A and B (pale white solid, 3.9 mg, >99% conversion).

Peptide Ac-C(SAr^2)WC(S^tBu)AR-$CONH_2$ 1d: **HRMS (ESI)** m/z: $C_{39}H_{54}N_{11}O_{10}S_4$ $[M+H]^+$ Calcd for 964.2932, Found 964.2968. **Analytical HPLC Method** (C18, Water (A)/ACN (B); gradient: 5 to 85% B (0.1% formic acid in ACN) in 20 min, flow rate = 1.0 mL/min, I = 220 nm): retention time of 13.07 min.

Analytical HPLC trace of purified peptide Ac-C(SAr^2)WC(S^tBu)AR-$CONH_2$ ***1d***

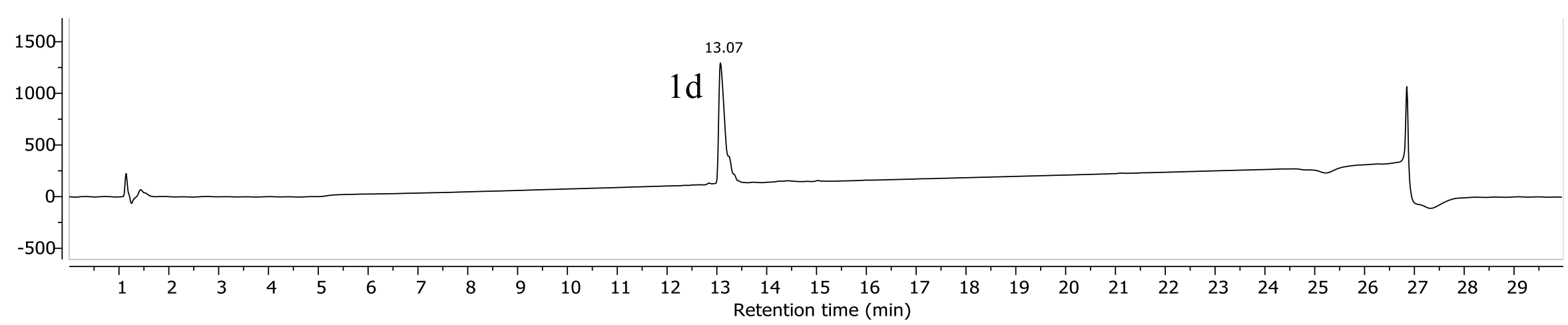

*HRMS spectrum of purified peptide Ac-C(SAr^2)WC(S^{t}Bu)AR-CONH$_2$ **1d***

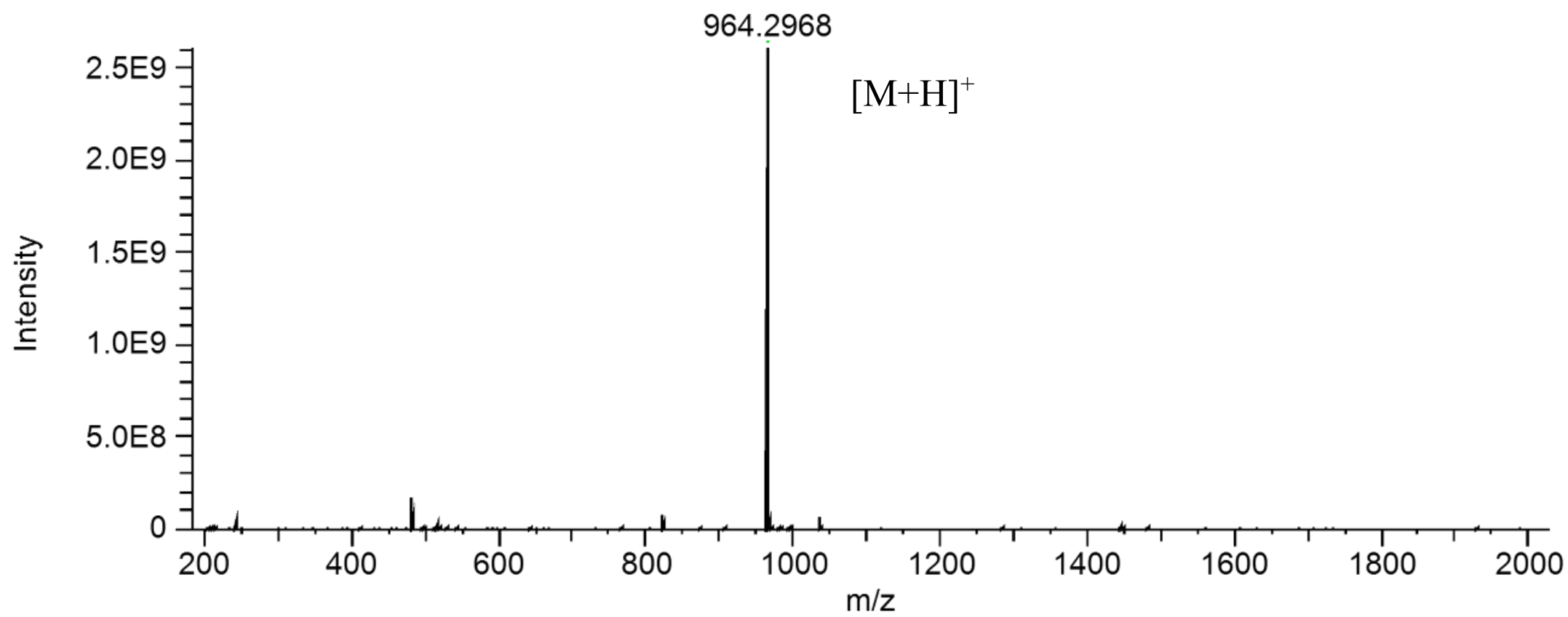

XI.1.2. Synthesis of Peptide Ac-C(SAr^2)YDKMWC(S^{t}Bu)A-CONH$_2$ 1e:

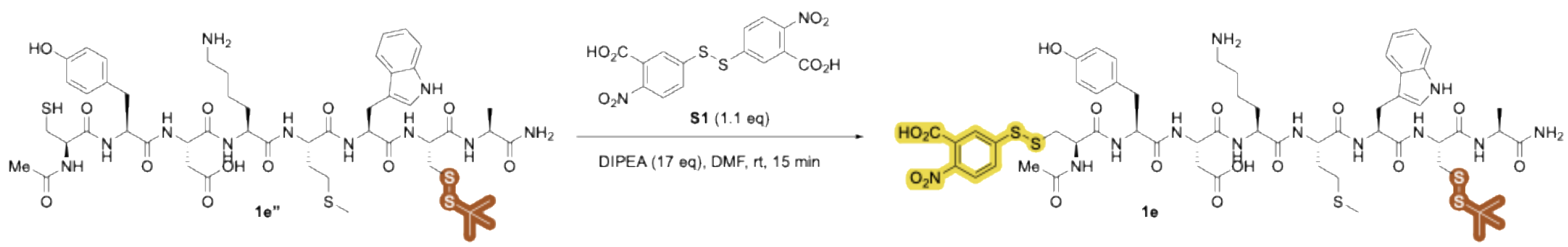

In a 1 dram vial equipped with a magnetic stirrer was added the purified peptide Ac-CYDKMWC(S^{t}Bu)A-CONH$_2$ **1e''** (4.7 mg, 0.0041 mmol, 1 eq), *N,N*-diisopropylethylamine (DIPEA, 12 µL, 0.69 mmol, 17 eq) and *N,N*-dimethylformamide (DMF, 700 µL), while stirring. In a separate 1 dram vial, 4,4'-disulfanediylbis-2-nitrobenzoic acid **S1** (1.8 mg, 0.0045 mmol, 1.1 eq) was taken and dissolved in DMF (300 µL) and the solution was added dropwise to the peptide solution and resulting mixture was allowed to stir 15 min at room temperature. After that, conversion of the aryl-disulfide containing peptide **1e** was determined via analytical HPLC using 0.1% formic acid (FA) in both mobile phases A and B (pale white solid, 5.5 mg, >99% conversion).

Peptide Ac-C(SAr)YDKMWC(S^{t}Bu)A-CONH$_2$ 1e: **HRMS (ESI)** m/z: $C_{57}H_{77}N_{12}O_{16}S_5$ [M+H]$^+$ Calcd for 1345.4179, Found 1345.4224; [M+2H]$^{+2}$ Calcd for 673.2053, Found 673.2151. **Analytical HPLC Method** (C18, Water (A)/ACN (B); gradient: 5 to 85% B (0.1% formic acid in ACN) in 20 min, flow rate = 1.0 mL/min, I = 220 nm)**:** retention time of 13.31 min.

*Analytical HPLC trace of purified peptide Ac-C(SAr)YDKMWC(S^{t}Bu)A-CONH$_2$ **1e***

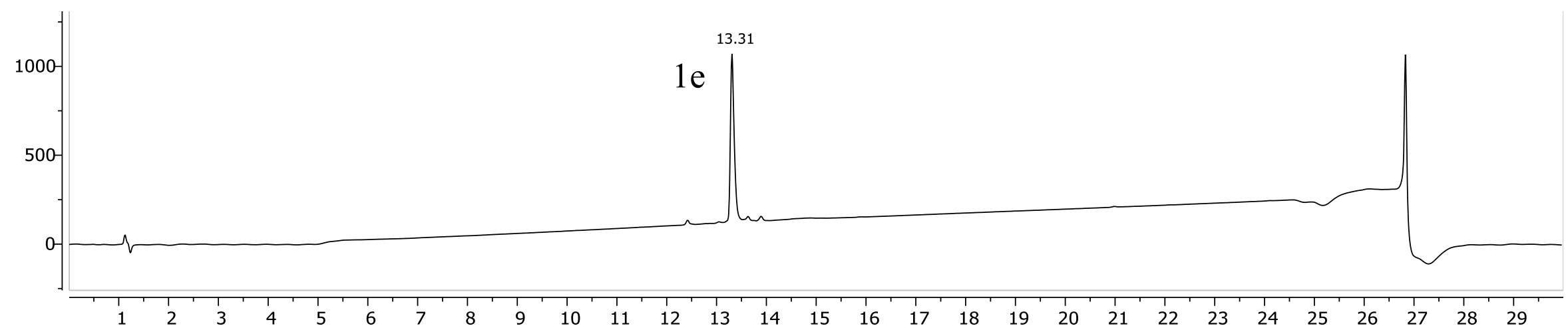

HRMS spectrum of purified peptide Ac-C(SAr)YDKMWC(S^{t}Bu)A-CONH$_2$ ***1e***

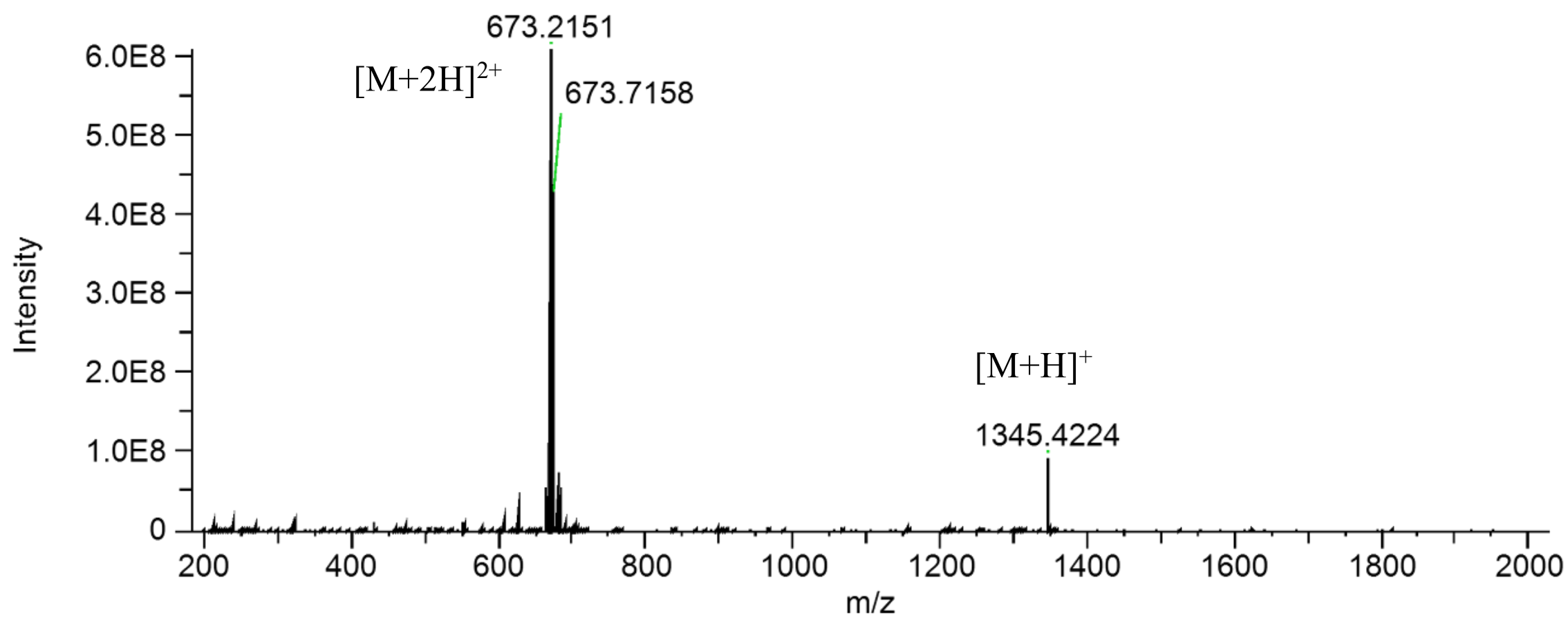

XI.1.3. Synthesis of Peptide Ac-C(SAr^2)WAFR-CONH$_2$ **1g**.

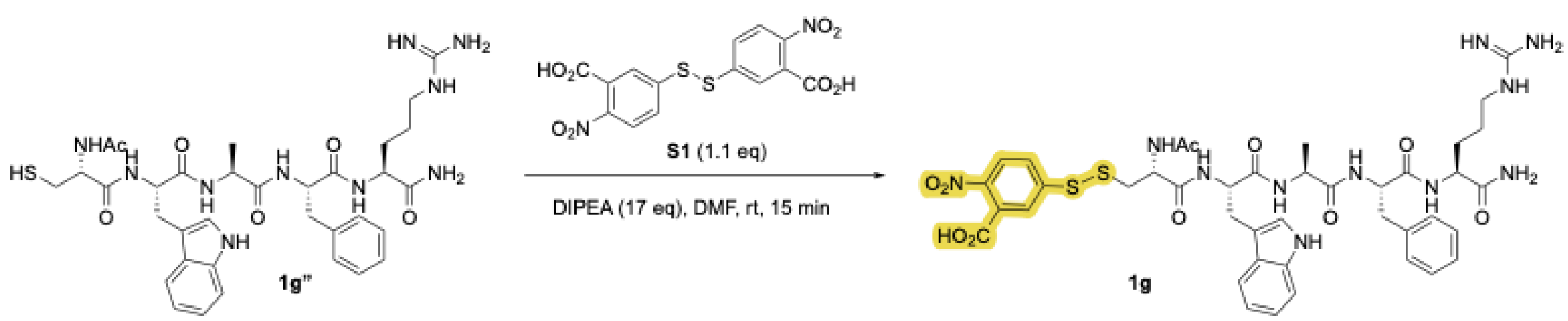

In a 1 dram vial equipped with a magnetic stirrer was added the purified peptide Ac-CWAFR-CONH$_2$ **1g''** (3 mg, 0.0041 mmol, 1 eq), *N,N*-diisopropylethylamine (DIPEA, 12 µL, 0.69 mmol, 17 eq) and *N,N*-dimethylformamide (DMF, 700 µL), while stirring. In a separate 1 dram vial, 4,4'-disulfanediylbis-2-nitrobenzoic acid **S1** (1.8 mg, 0.0045 mmol, 1.1 eq) was taken and dissolved in DMF (300 µL) and the solution was added dropwise to the peptide solution and resulting mixture was allowed to stir 15 min at room temperature. After that, conversion of the aryl-disulfide containing peptide **1g** was determined via analytical HPLC using 0.1% formic acid (FA) in both mobile phases A and B (pale white solid, 3.7 mg, >99% conversion).

Peptide Ac-C(SAr^2)WAFR-CONH$_2$ 1g: **HRMS (ESI)** m/z: $C_{41}H_{50}N_{11}O_{10}S_2$ [M+H]$^+$ Calcd for 920.3178, Found 920.3179. **Analytical HPLC Method** (C18, Water (A)/ACN (B); gradient: 5 to 85% B (0.1% formic acid in ACN) in 20 min, flow rate = 1.0 mL/min, I = 220 nm)**:** retention time of 11.92 min.

*Analytical HPLC trace of purified peptide Ac-C(SAr)WAFR-CONH$_2$ **1g***

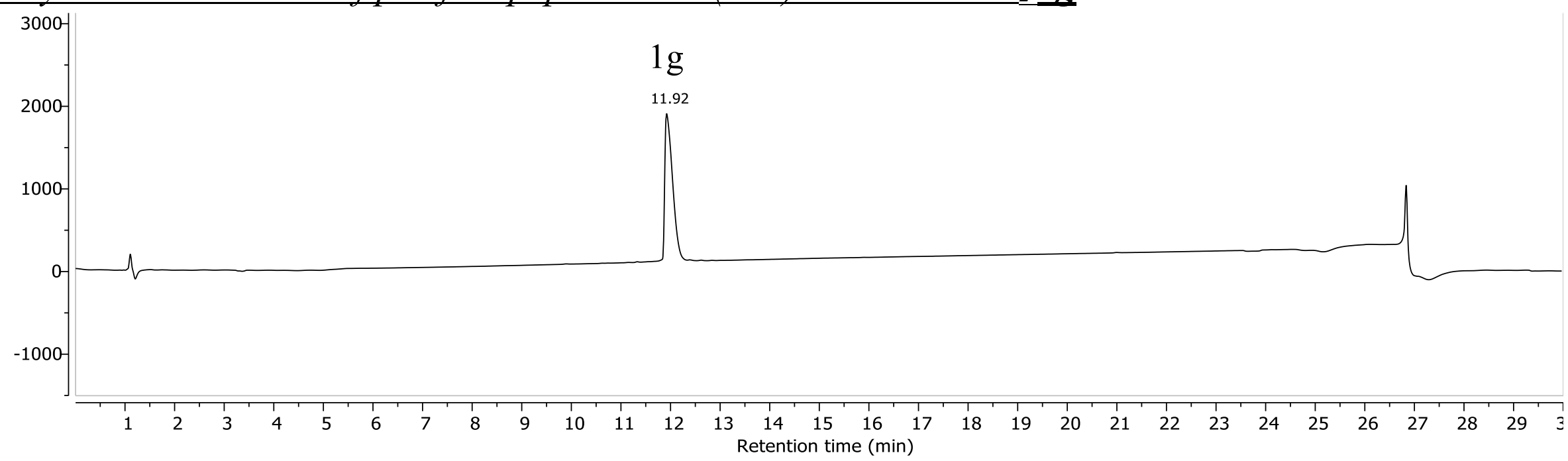

*HRMS spectrum of purified peptide Ac-C(SAr)WAFR-CONH$_2$ **1g***

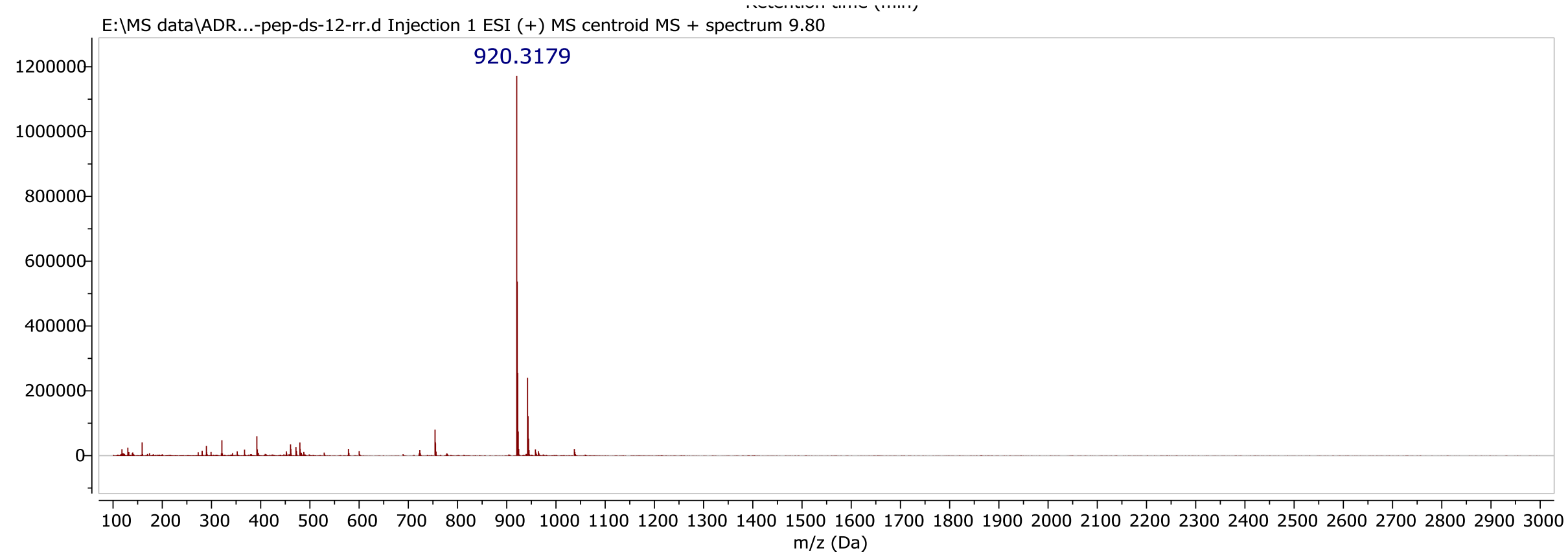

XI.2. Synthesis of Ethyl Disulfide in Cys-Containing Peptide Sequence

XI.2.1. Synthesis of Peptide Ac-C(SEt)WNC(S^{t}Bu)AR-CONH$_2$ **1f**:

Normal amino acids were coupled according to the general procedure **V**. Ethyl disulfide residue was introduced at N-terminal by coupling with Fmoc-C(SEt)-CO$_2$H **1b** (403 mg, 1 mmol, 5 eq) on resin bounded peptide (558 mg, 0.2 mmol, 1 eq) with DIPEA (0.17 mL, 1 mmol, 5 eq) and HBTU (380 mg, 1 mmol, 5 eq) in 2.1 mL DMF solution). After the reaction, peptide was cleaved from resin by using 5 mL of cleavage cocktail mixture (TFA:TIPS:H$_2$O = 95:2.5:2.5). The resin was removed by filtration, and the resulting solution was concentrated. Peptide was precipitated and centrifugated with cold diethyl ether (3 x 10 mL) to obtain the crude product. Crude peptide was dissolved in ACN:H$_2$O and purified by **Preparatory HPLC Method** (C18, Water (A)/ACN (B); gradient: 10 to 65% **B** (0.1 % FA) in 35 min; 65 to 90% **B** (0.1 % FA) in 35.1 to 45 min, flow rate = 4.7 mL/min, I = 220 nm) to obtain the desired product **1f** (white solid, 10 mg) in ~6% conversion.

Peptide Ac-C(SEt)WNC(S^{t}Bu)AR-CONH$_2$ 1f: **HRMS (ESI)** m/z: $C_{38}H_{61}N_{12}O_8S_4$ $[M+H]^+$ Calcd for 941.3613, Found 941.3645. **Analytical HPLC Method** (C18, Water (A)/ACN (B); gradient: 5 to 85% B (0.1% formic acid in ACN) in 20 min, flow rate = 1.0 mL/min, I = 220 nm)**:** retention time of 12.75 min.

Analytical HPLC trace of purified peptide Ac-C(SEt)WNC(S^{t}Bu)AR-CONH$_2$ ***1f***

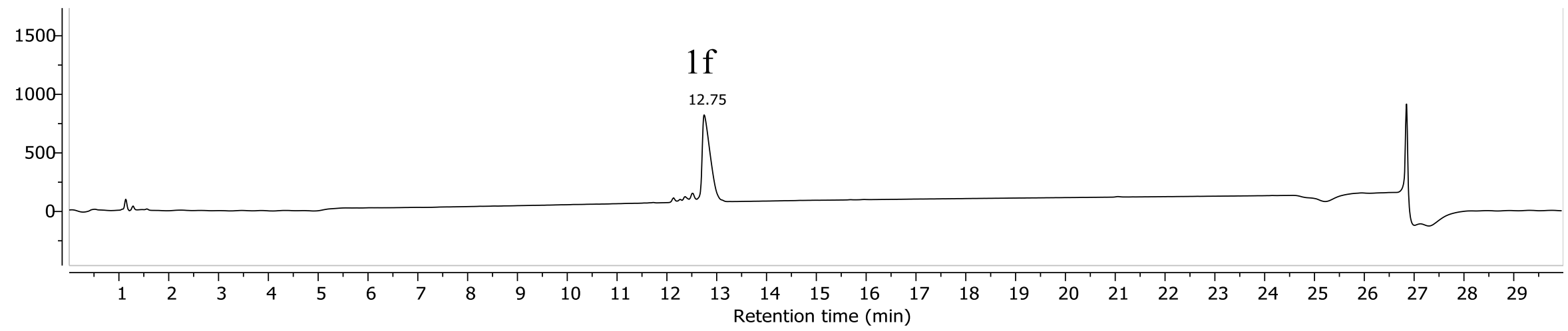

HRMS spectrum of purified peptide Ac-C(SEt)WNC(S^{t}Bu)AR-CONH$_2$ ***1f***

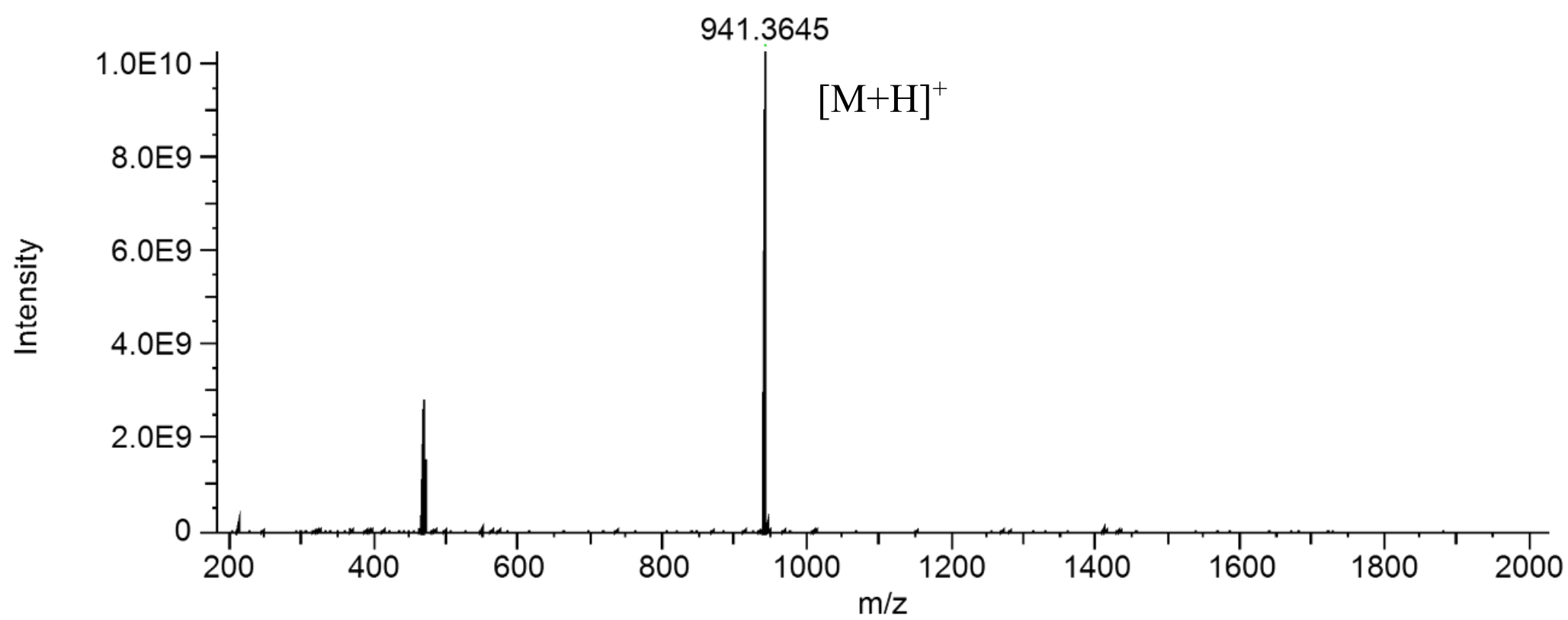

XI.2.2. Synthesis of Peptide Ac-WNC(SEt)AR-CONH$_2$ **1h**:

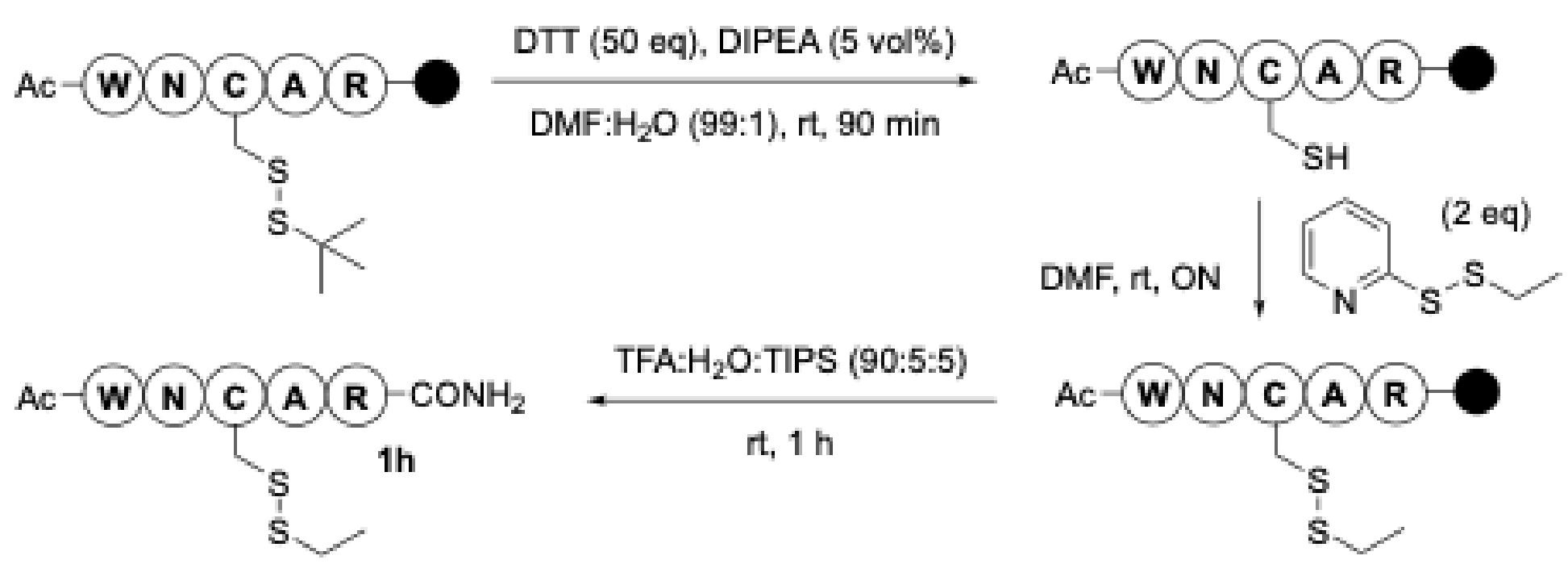

Normal amino acids were coupled according to the modified general procedure **V**. Ethyl disulfide residue was introduced on the free Cys side chain on resin in the peptide sequence. At first, on resin, *tert*-butyl protection on Cys containing peptide (301 mg, 0.1 mmol, 1 eq) was cleaved by using dithiothreitol (DTT, 705 g, 5 mmol, 50 eq), in 5 mL DMF:H_2O (99:1) and N,N-diisopropyletylamine (DIPEA, 250 uL, 5% of total volume) while stirring at room temperature for 1.5 h. After completion, the resin was washed with DMF (3 x 5 mL) to remove the excess reagents. After that, the resin was dissolved in DMF and solution of 2-(ethyldisulfaneyl)pyridine **S4** (34 mg, 0.2 mmol, 2 eq in 4 mL of DMF) was added and mixed on a wrist action shaker for overnight at room temperature. Finally, peptide was cleaved from the resin using 5 mL cocktail consisting of 90:5:5 trifluoroacetic acid : water : triethylsilane (TES) for 1 h. The resin was removed by filtration, and the resulting solution was concentrated. Peptide was precipitated and centrifugated with cold diethyl ether (3 x 10 mL) to obtain the crude product. Crude peptide was dissolved in ACN:H_2O and

purified by **Preparatory HPLC Method** (C18, Water (A)/ACN (B); gradient: 10 to 65% **B** (0.1 % FA) in 35 min; 65 to 90% **B** (0.1 % FA) in 35.1 to 45 min, flow rate = 4.7 mL/min, I = 220 nm) to obtain the desired pure peptide **1h** (pale white solid, 11 mg) in 13% conversion.

Peptide Ac-WNC(SEt)AR-CONH$_2$ 1h: HRMS (ESI) m/z: $C_{31}H_{48}N_{11}O_7S_2$ $[M+H]^+$ Calcd for 750.3174, Found 750.3156. **Analytical HPLC Method** (C18, Water (A)/ACN (B); gradient: 5 to 85% B (0.1% formic acid in ACN) in 20 min, flow rate = 1.0 mL/min, I = 220 nm)**:** retention time of 11.65 min.

Analytical HPLC trace of purified peptide Ac-WNC(SEt)AR-CONH$_2$ ***1h***

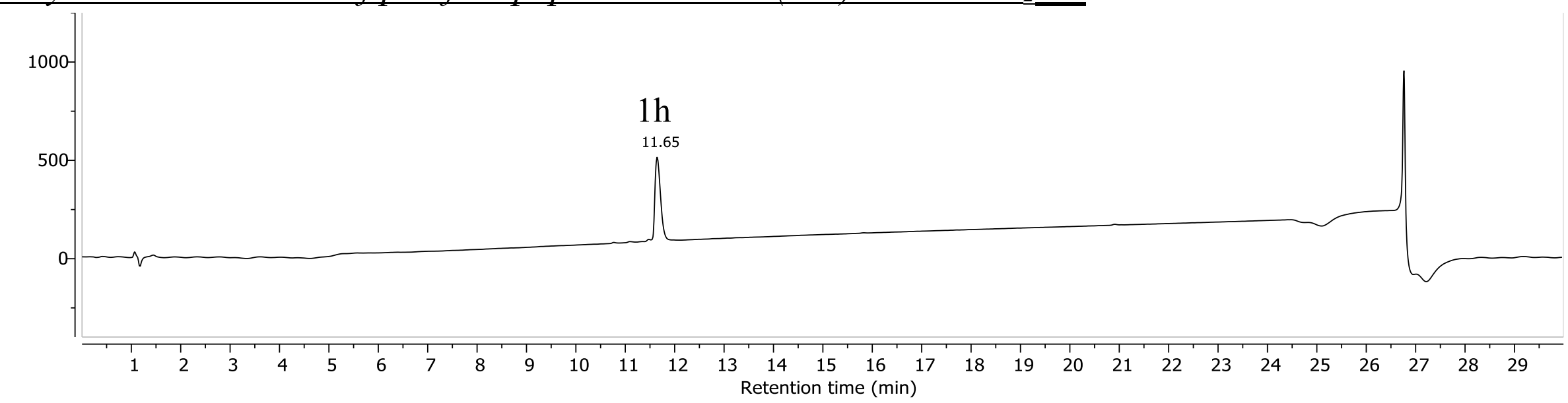

HRMS spectrum of purified peptide Ac-WNC(SEt)AR-CONH$_2$ ***1h***

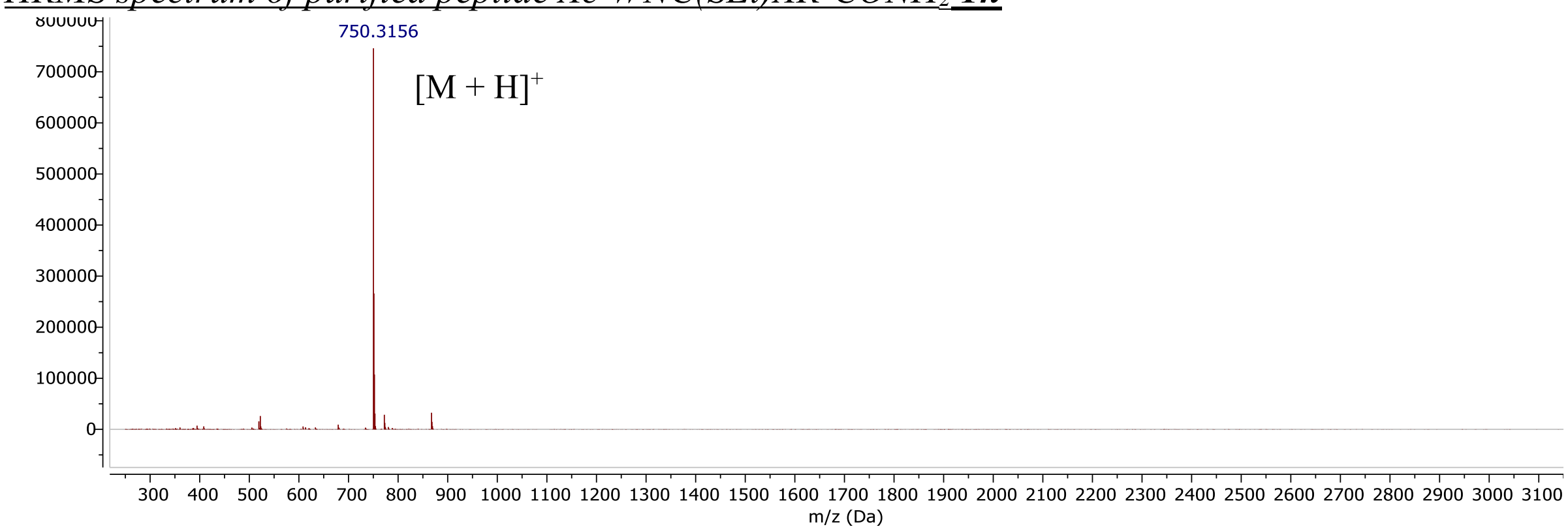

XII.a. Supplementary Figure 2. Orthogonal Cleavage and Modification of Aryl and tert-Butyl containing Disulfides in Fully Unprotected Peptide **1d**.

XII.a.1. Orthogonal Cleavage of Aryl Containing Disulfide on Peptide **1d**.

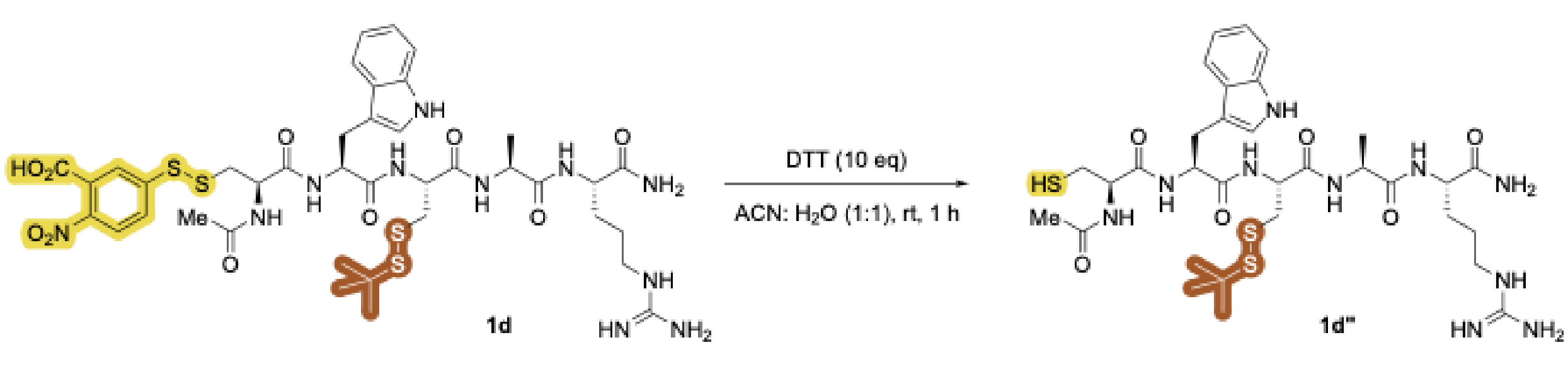

In a 1 dram vial equipped with a magnetic stirrer was added the purified peptide Ac-C(SAr^2)WC(S^{t}Bu)AR-CONH$_2$ **1d** (4 mg, 0.0041 mmol, 1 eq) and it was dissolved in ACN (500 µL), and H_2O (200 µL) while stirring. In a separate 1 dram vial, dithiothreitol (DTT) (6.3 mg, 0.041 mmol, 10 eq) was taken and dissolved in H_2O (300 µL) and the solution was added dropwise to the peptide solution and resulting mixture was

allowed to stir 1 h at room temperature. After that, the selective reduced thiol product Ac-CWC(S^{t}Bu)AR-CONH$_2$ **1d''** (pale white solid, 3 mg, >99% conversion) was determined via analytical HPLC using 0.1% formic acid (FA) in both mobile phases A and B. Following analysis, product was subsequently purified using this same method.

Peptide Ac-CWC(S^{t}Bu)AR-CONH$_2$ 1d'': **HRMS (ESI)** m/z: $C_{32}H_{51}N_{10}O_6S_3$ [M+H]$^+$ Calcd for 767.3150, Found 767.3129. **Analytical HPLC Method** (C18, Water (A)/ACN (B); gradient: 5 to 85% B (0.1% formic acid in ACN) in 20 min, flow rate = 1.0 mL/min, I = 220 nm)**:** retention time of 11.95 min.

*Analytical HPLC trace of the crude reaction mixture converting Ac-C(SAr)WC(S^{t}Bu)AR-CONH$_2$ **1d** to Ac-CWC(S^{t}Bu)AR-CONH$_2$ **1d''***

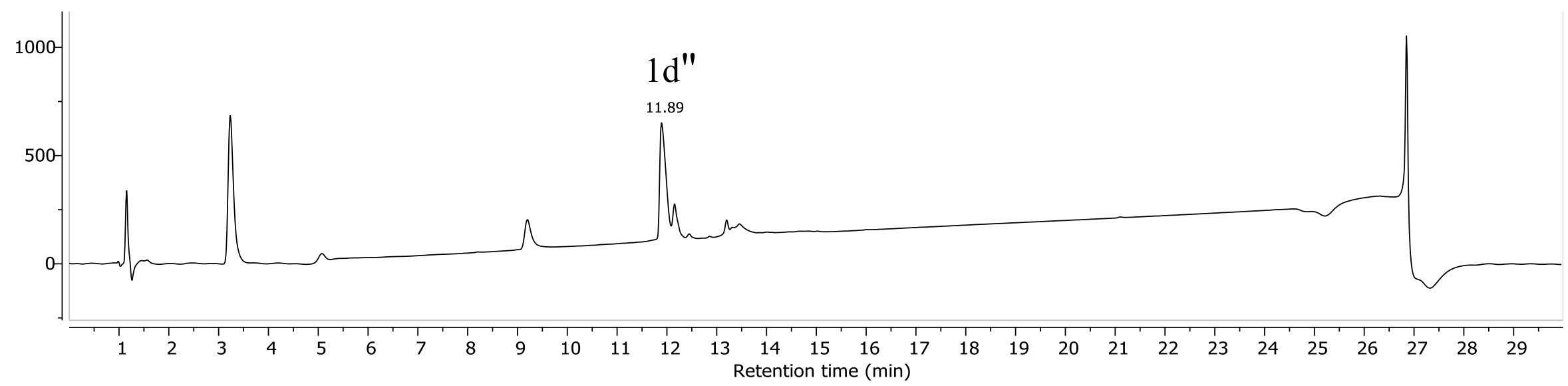

*Analytical HPLC trace of purified peptide Ac-CWC(S^{t}Bu)AR-CONH$_2$ **1d''***

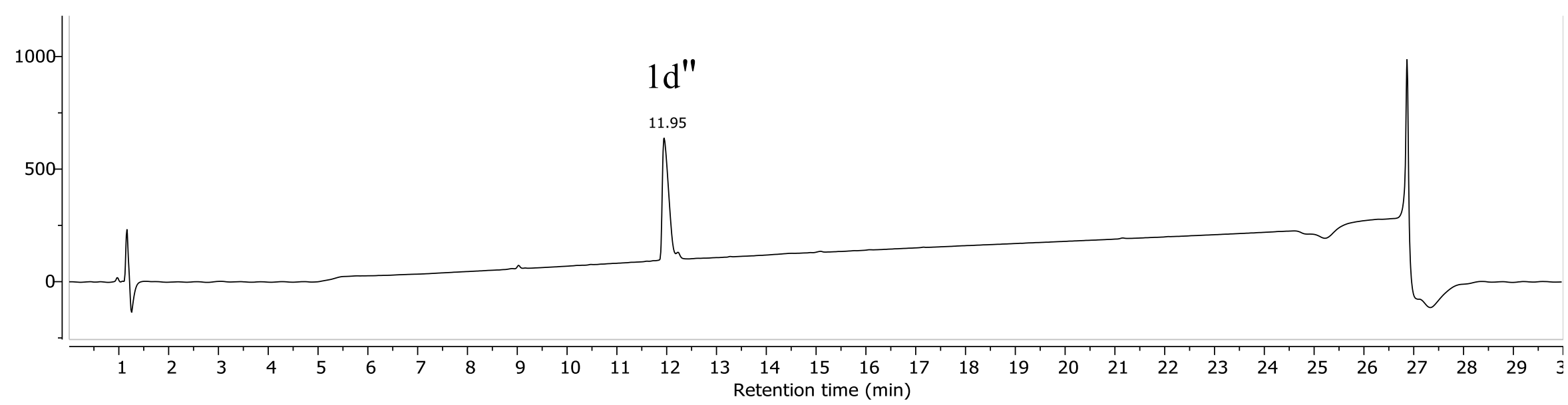

*HRMS spectrum of purified peptide Ac-CWC(S^{t}Bu)AR-CONH$_2$ **1d''***

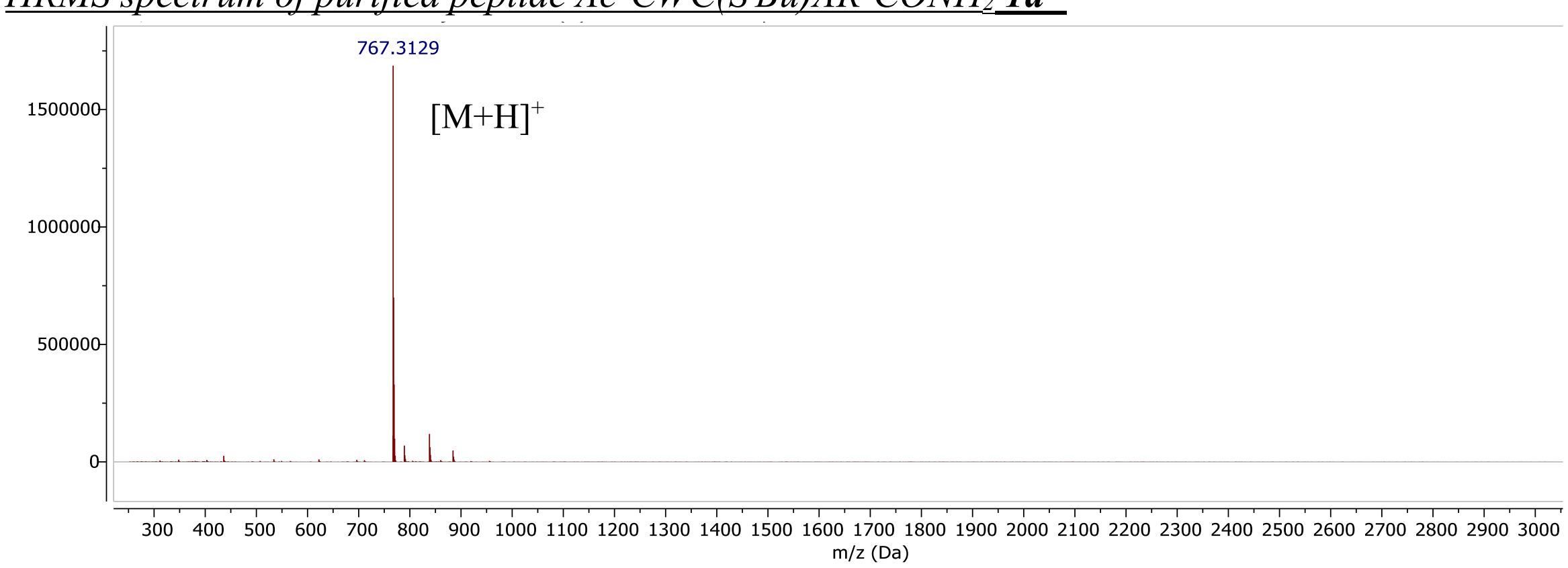

XII.a.2. Orthogonal Modification of Free Cys on Unprotected Peptide **1d''**.

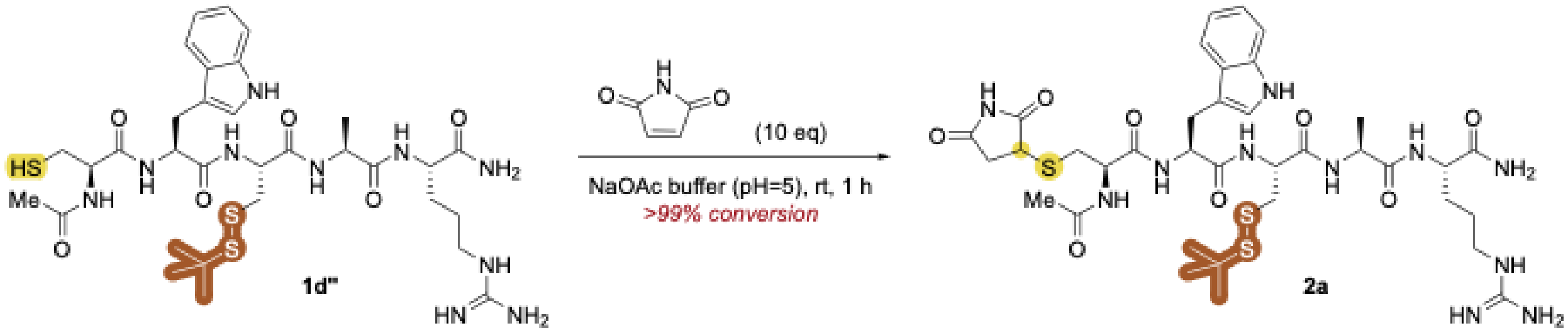

In a 1 dram vial equipped with a magnetic stirrer was added the purified peptide Ac-CWC(S^{t}Bu)AR-CONH$_2$ **1d''** (3 mg, 0.0039 mmol, 1 eq) and it was dissolved in sodium acetate buffer (NaOAc buffer pH=5) (300 µL) while stirring. In a separate 1 dram vial, maleimide (3.8 mg, 0.039 mmol, 10 eq) was taken and dissolved in NaOAc buffer (200 µL) and the solution was added dropwise to the peptide solution and resulting mixture was allowed to stir 1 h at room temperature. After that, the selective maleimide adduct peptide **2a** (pale white solid, 3.3 mg, >99% conversion) was determined via analytical HPLC using 0.1% formic acid (FA) in both mobile phases A and B. Following analysis, product was subsequently purified using this same method.

Peptide Ac-C(maleimide)WC(S^{t}Bu)AR-CONH$_2$ 2a: **HRMS (ESI)** m/z: $C_{36}H_{54}N_{11}O_8S_3$ $[M+H]^+$ Calcd for 864.3313, Found 864.3306. **Analytical HPLC Method** (C18, Water (A)/ACN (B); gradient: 5 to 85% B (0.1% formic acid in ACN) in 20 min, flow rate = 1.0 mL/min, I = 220 nm)**:** retention time of 11.38 min.

*Analytical HPLC trace of the crude reaction mixture converting Ac-CWC(S^{t}Bu)AR-CONH$_2$ **1d''** to Ac-C(maleimide)WC(S^{t}Bu)AR-CONH$_2$ **2a***

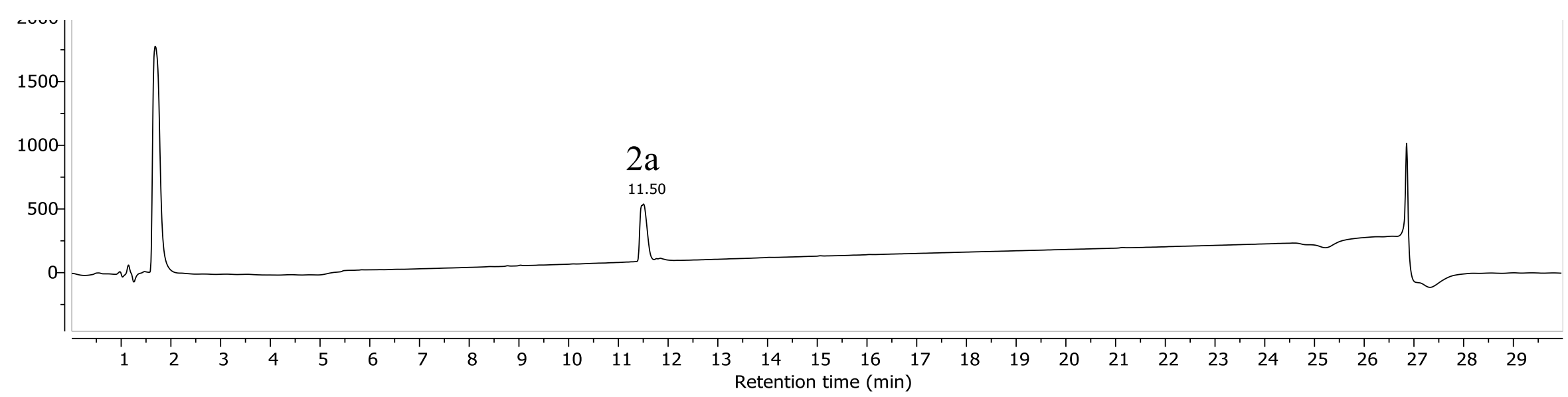

*Analytical HPLC trace of purified peptide Ac-C(maleimide)WC(S^{t}Bu)AR-CONH$_2$ **2a***

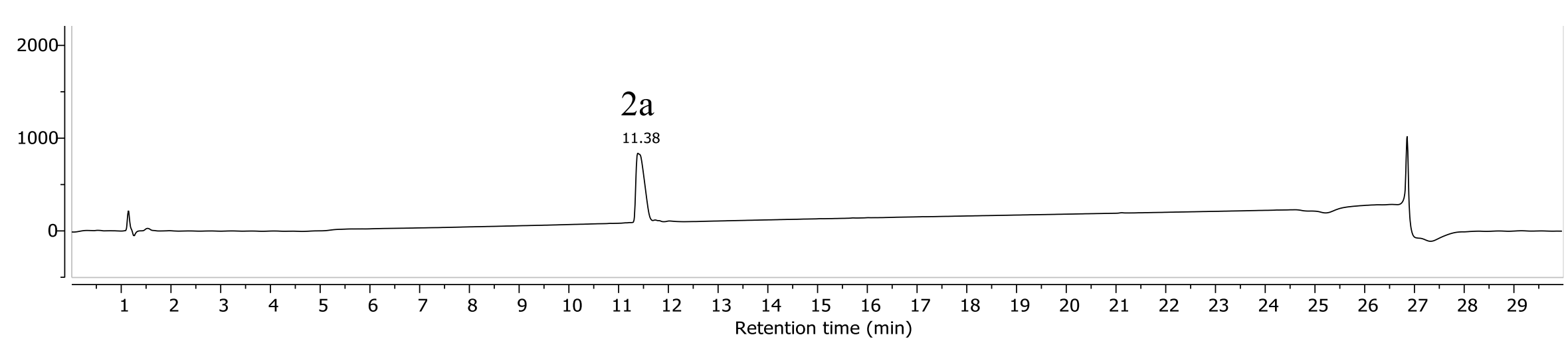

HRMS spectrum of purified peptide Ac-C(maleimide)WC(S^{t}Bu)AR-CONH$_2$ ***2a***

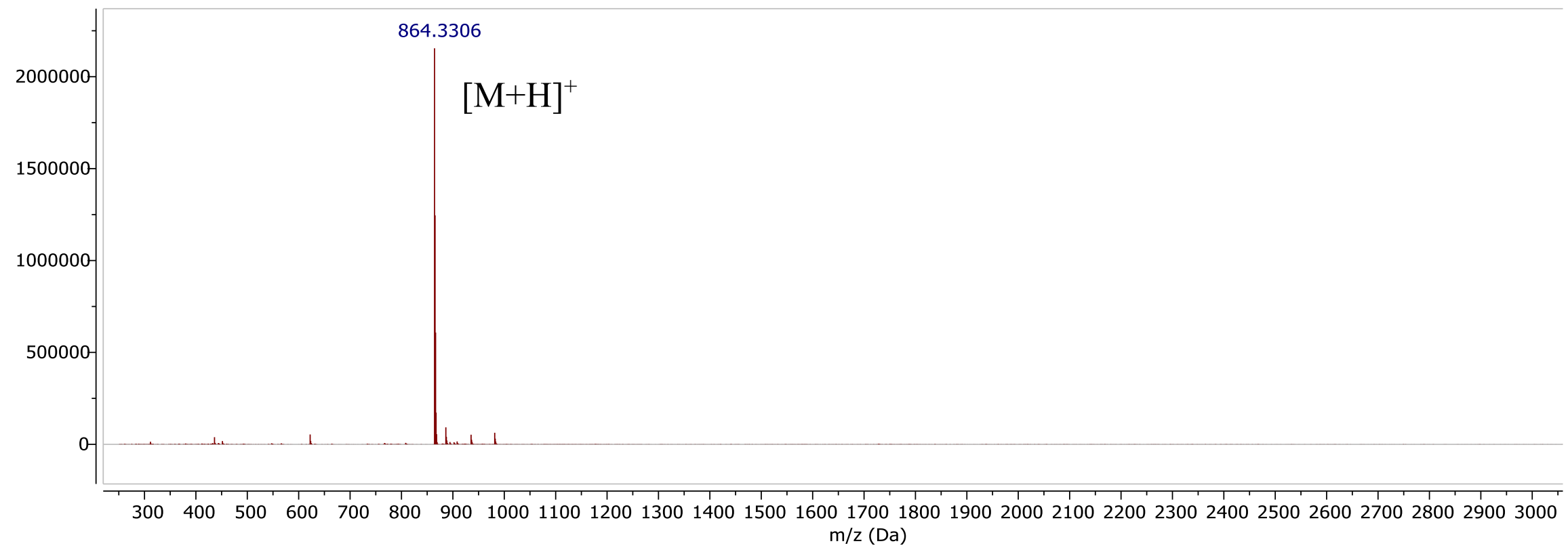

XII.a.3. Orthogonal Cleavage of *tert*-Butyl Containing Disulfide on Peptide **2a**.

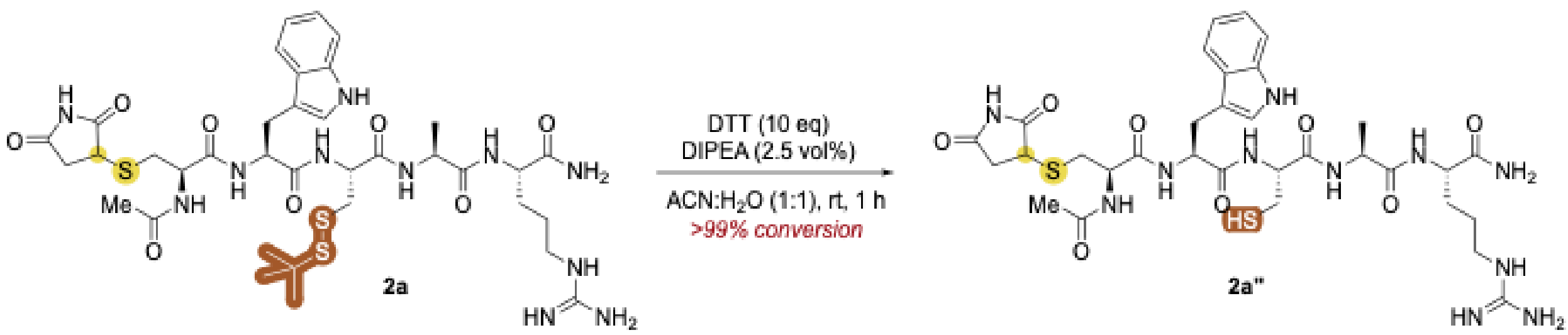

In a 1 dram vial equipped with a magnetic stirrer was added the purified peptide Ac-C(maleimide)WC(S^{t}Bu)AR-CONH$_2$ **2a** (3 mg, 0.0034 mmol, 1 eq) and dissolved in ACN (500 µL), and H_2O (200 µL) while stirring. In a separate 1 dram vial, dithiothreitol (DTT) (5.3 mg, 0.034 mmol, 10 eq) was taken and dissolved in H_2O (300 µL) and the solution was added dropwise to the peptide solution followed by addition of *N,N*-diisopropylethylamine (DIPEA, 25 µL) at once and resulting mixture was allowed to stir 1 h at room temperature. After that, the selective reduced thiol product **2a''** (pale white solid, 2.6 mg, >99% conversion) was determined via analytical HPLC using 0.1% formic acid (FA) in both mobile phases A and B. Following analysis, product was subsequently purified using this same method.

Peptide Ac-C(maleimide)WCAR-CONH$_2$ 2a'': **HRMS (ESI)** m/z: $C_{32}H_{46}N_{11}O_8S_2$ $[M+H]^+$ Calcd for 776.2967, Found 776.2993. **Analytical HPLC Method** (C18, Water (A)/ACN (B); gradient: 5 to 85% B (0.1% formic acid in ACN) in 20 min, flow rate = 1.0 mL/min, I = 220 nm)**:** retention time of 8.86 min.

Analytical HPLC trace of the crude reaction mixture converting Ac-C(maleimide)WC(S^{t}Bu)AR-CONH$_2$ ***2a*** *to Ac-C(maleimide)WCAR-CONH$_2$* ***2a''***

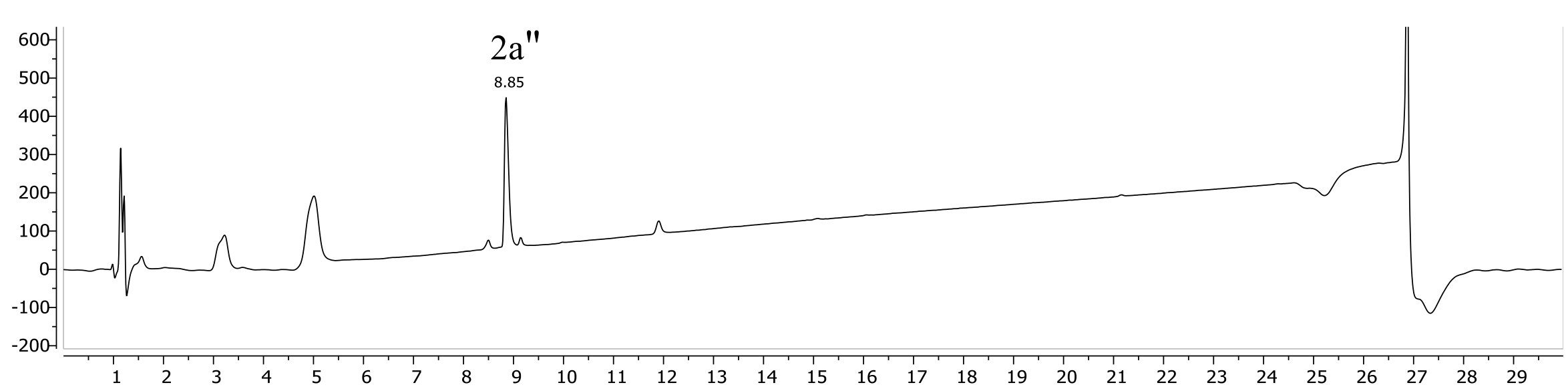

Analytical HPLC trace of purified peptide Ac-C(maleimide)WCAR-CONH₂ ***2a''***

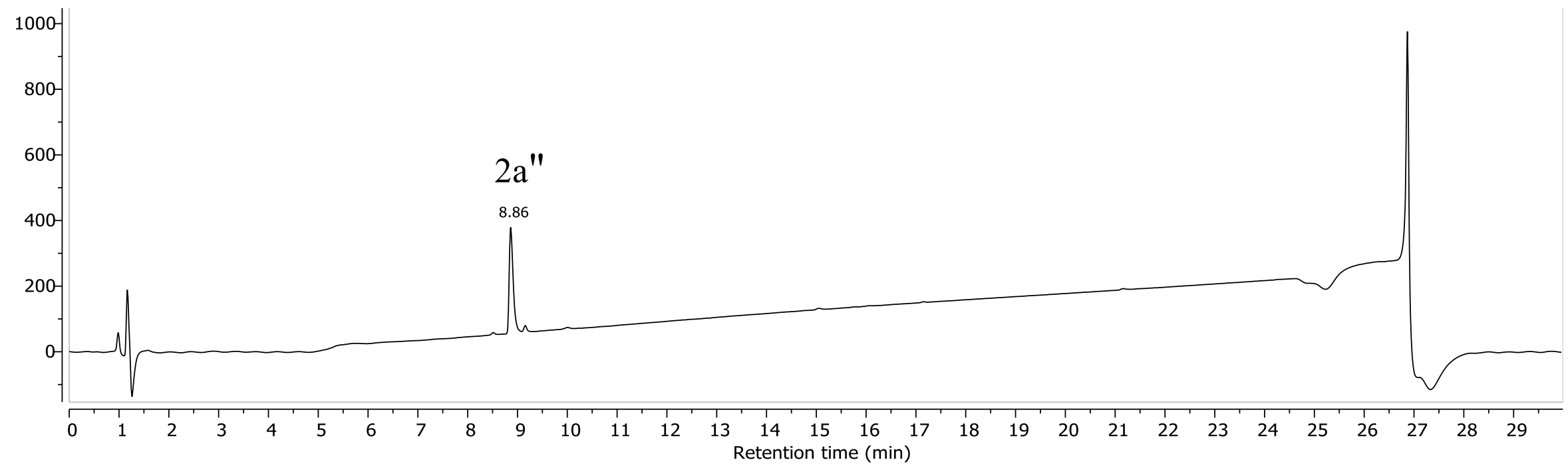

HRMS spectrum of purified peptide Ac-C(maleimide)WCAR-CONH₂ ***2a''***

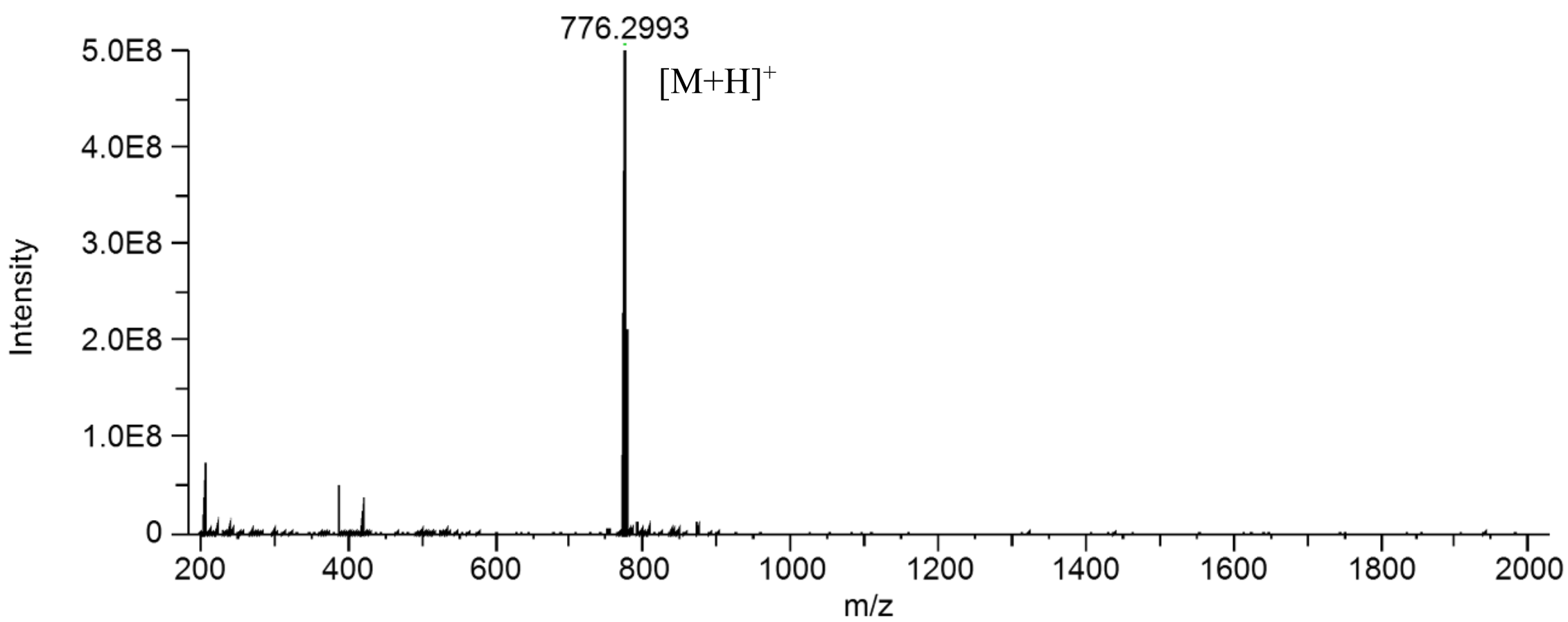

XII.a.4. Orthogonal Modification of Free Cys on Unprotected Peptide **2a''**.

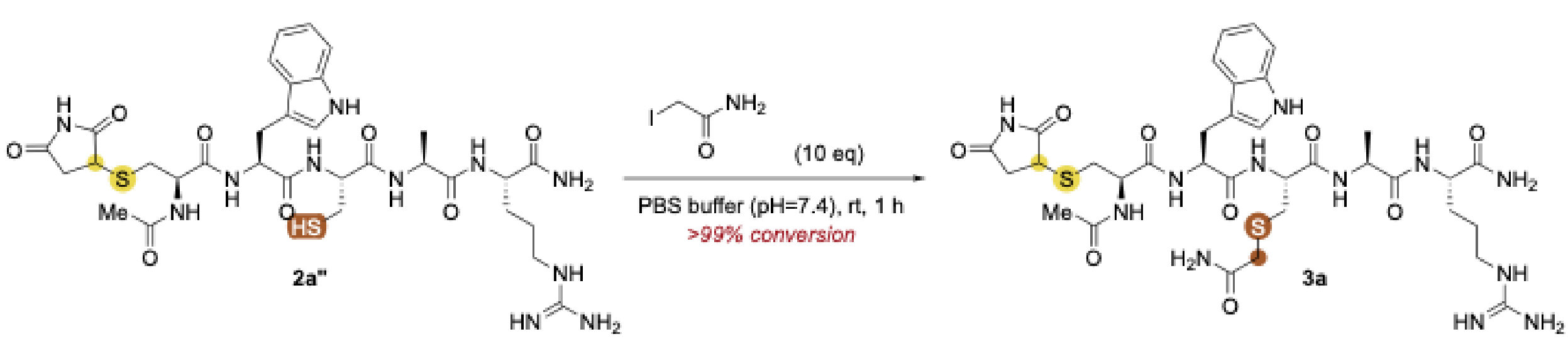

In a 1 dram vial equipped with a magnetic stirrer was added the purified peptide Ac-C(maleimide)WCAR-CONH₂ **2a''** (2 mg, 0.0025 mmol, 1 eq) and it was dissolved in PBS buffer (pH=7.4) (300 µL) while stirring. In a separate 1 dram vial, iodoacetamide (4.6 mg, 0.025 mmol, 10 eq) was taken and dissolved in PBS buffer (200 µL) and the solution was added dropwise to the peptide solution and resulting mixture was allowed to stir 1 h at room temperature. After that, the selective iodoacetamide adduct peptide **3a** (pale white solid, 2 mg, >99% conversion) was determined via analytical HPLC using 0.1% formic acid (FA) in both mobile phases A and B. Following analysis, product was subsequently purified using this same method.

Peptide Ac-C(maleimide)WC(iodoacetamide)AR-CONH₂ 3a: **HRMS (ESI)** m/z: $C_{34}H_{49}N_{12}O_9S_2$ $[M+H]^+$ Calcd for 833.3181, Found 833.3207. **Analytical HPLC Method** (C18, Water (A)/ACN (B);

gradient: 5 to 85% B (0.1% formic acid in ACN) in 20 min, flow rate = 1.0 mL/min, I = 220 nm)**:** retention time of 8.29 min.

*Analytical HPLC trace of the crude reaction mixture converting Ac-C(maleimide)WCAR-$CONH_2$ **2a"** to Ac-C(maleimide)WC(iodoacetamide)AR-$CONH_2$ **3a***

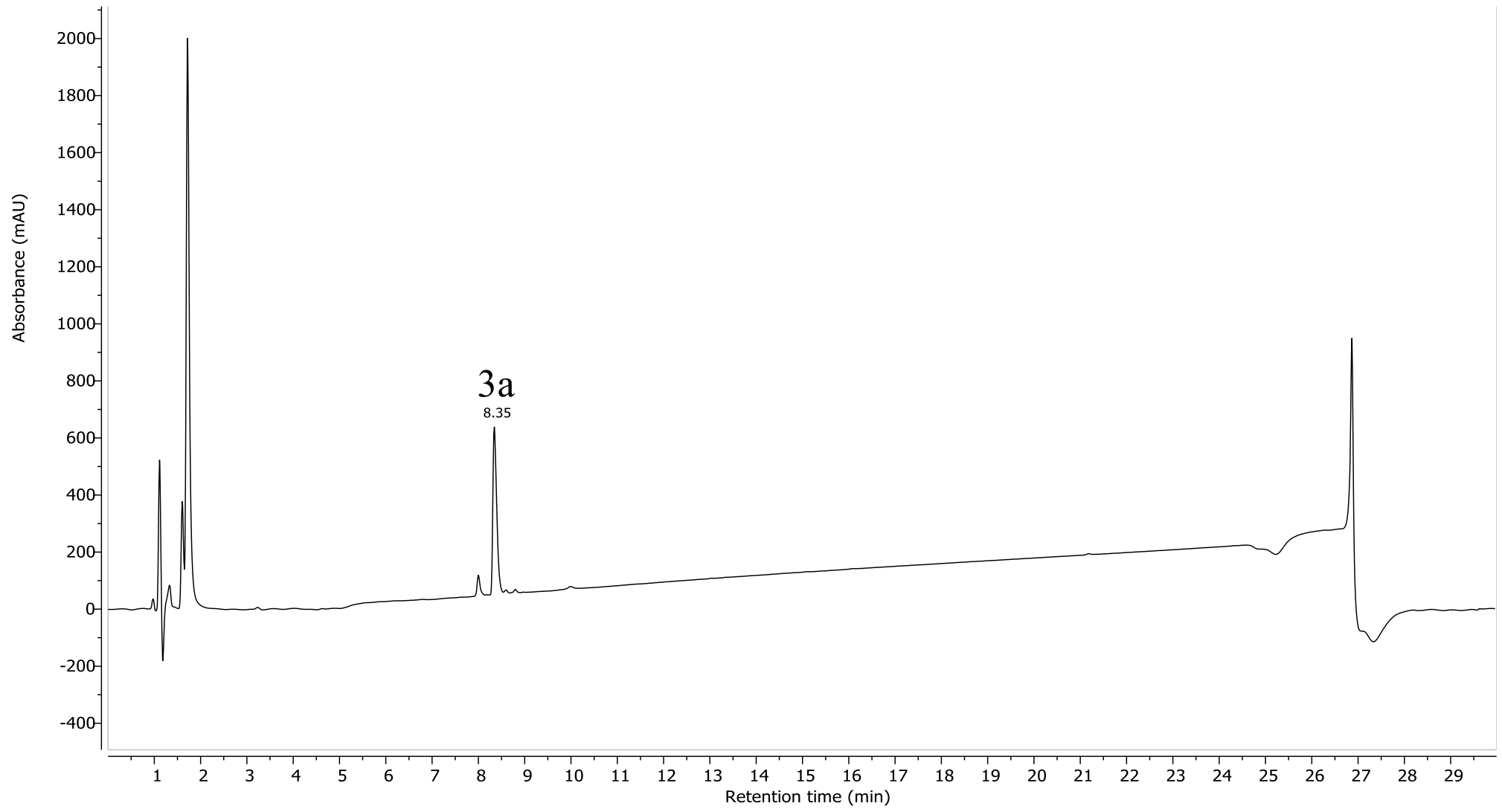

*Analytical HPLC trace of purified peptide Ac-C(maleimide)WC(iodoacetamide)AR-$CONH_2$ **3a***

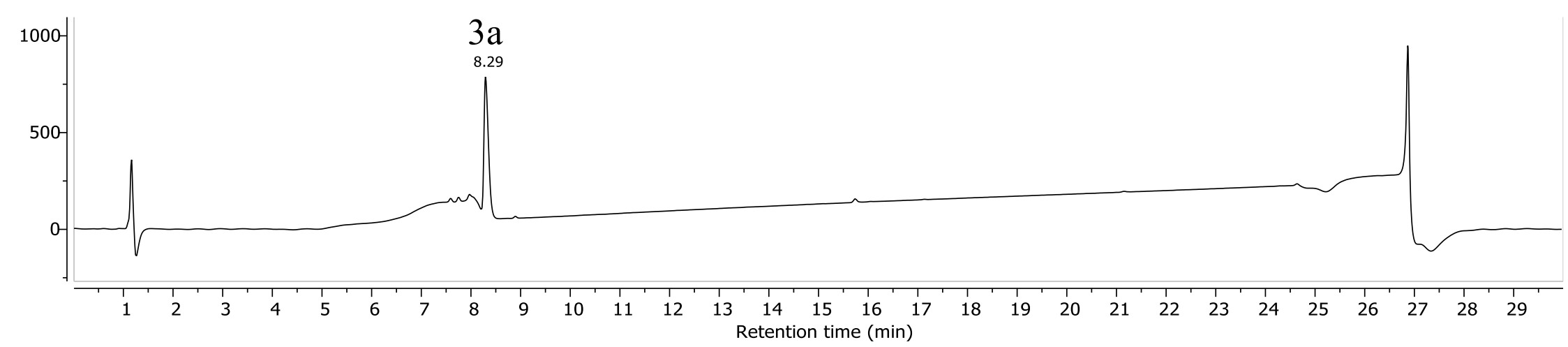

*HRMS spectrum of purified peptide Ac-C(maleimide)WC(iodoacetamide)AR-$CONH_2$ **3a***

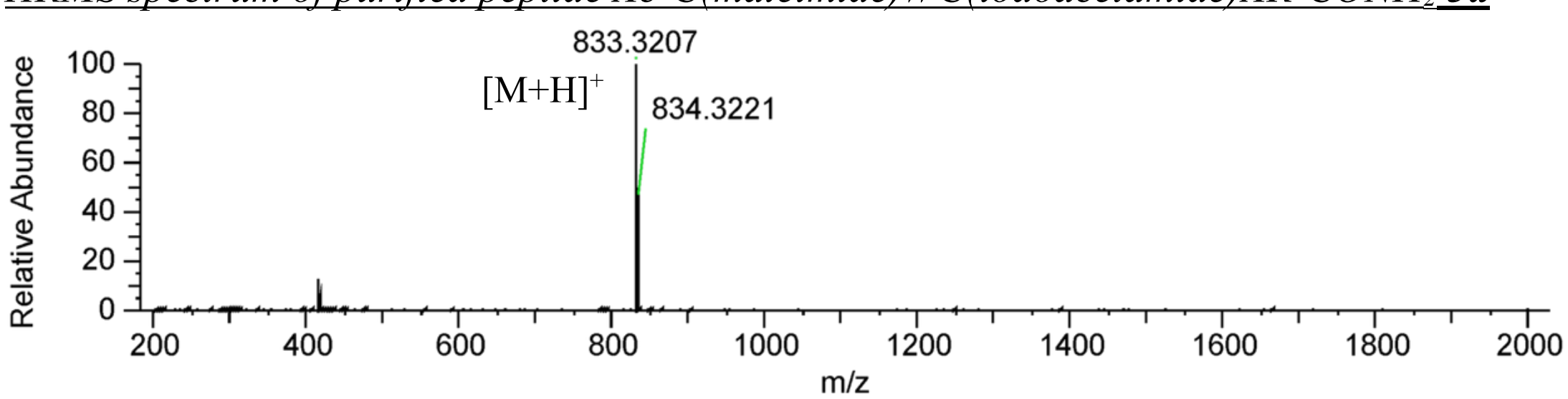

XII.b. Supplementary Figure 3. Orthogonal Cleavage and Modification of Aryl and *tert*-Butyl Containing Disulfides in Fully Unprotected Peptide **1e**.

XII.b.1. Orthogonal Cleavage of Aryl Containing Disulfide on Peptide **1e**.

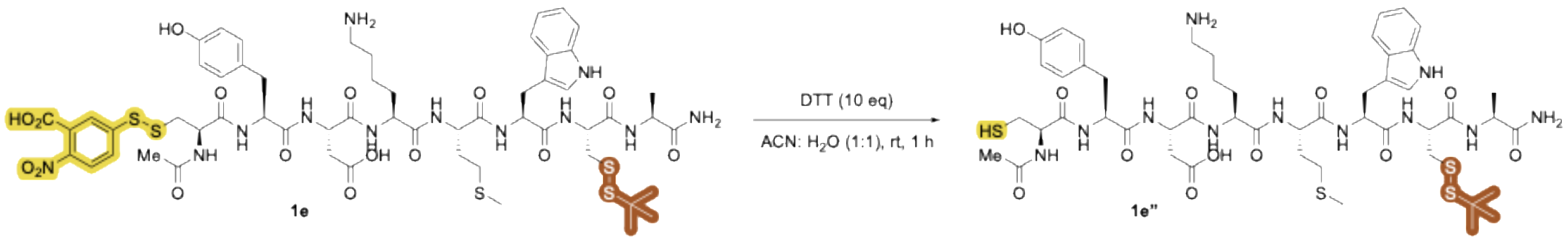

In a 1 dram vial equipped with a magnetic stirrer was added the purified peptide Ac-C(SAr)YDKMWC(S^{t}Bu)A-CONH$_2$ **1e** (4 mg, 0.0029 mmol, 1 eq) and it was dissolved in ACN (500 µL), and H_2O (200 µL) while stirring. In a separate 1 dram vial, dithiothreitol (DTT) (4.5 mg, 0.029 mmol, 10 eq) was taken and dissolved in H_2O (300 µL) and the solution was added dropwise to the peptide solution and resulting mixture was allowed to stir 1 h at room temperature. After that, the selective reduced thiol product Ac-CYDKMWC(S^{t}Bu)A-CONH$_2$ **1e"** (pale white solid, 3.3 mg, >99% conversion) was determined via analytical HPLC using 0.1% formic acid (FA) in both mobile phases A and B. Following analysis, product was subsequently purified using this same method.

Peptide Ac-CYDKMWC(S^{t}Bu)A-CONH$_2$ 1e": **HRMS (ESI)** m/z: $C_{50}H_{74}N_{11}O_{12}S_4$ $[M+H]^+$ Calcd for 1148.4396, Found 1148.4362; $[M+Na]^+$ Calcd for 1170.4215, Found 1170.4171. **Analytical HPLC Method** (C18, Water (A)/ACN (B); gradient: 5 to 85% B (0.1% formic acid in ACN) in 20 min, flow rate = 1.0 mL/min, I = 220 nm)**:** retention time of 12.28 min.

*Analytical HPLC trace of the crude reaction mixture converting Ac-C(SAr)YDKMWC(S^{t}Bu)A-CONH$_2$ **1e** to Ac-CYDKMWC(S^{t}Bu)A-CONH$_2$ **1e"***

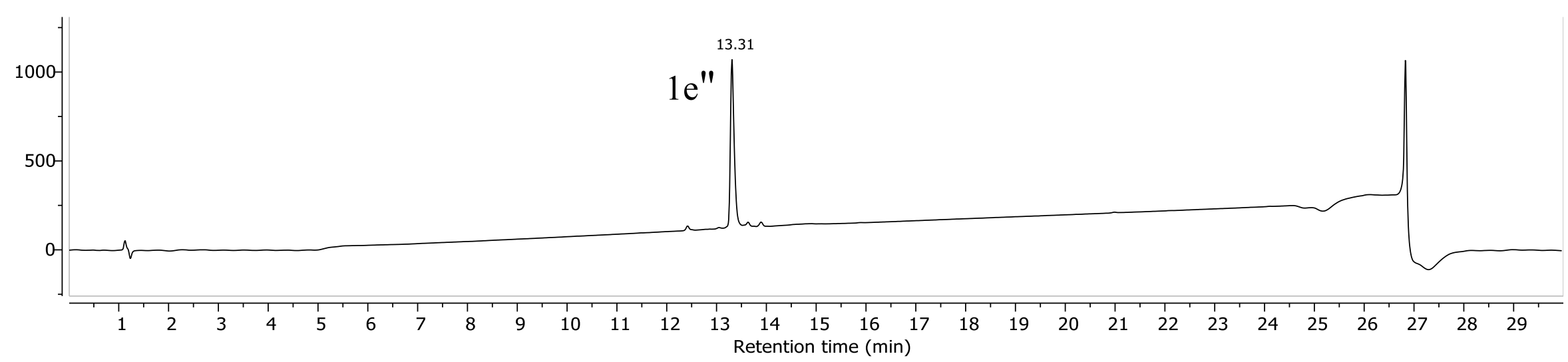

*Analytical HPLC trace of purified peptide Ac-CYDKMWC(S^{t}Bu)A-CONH$_2$ **1e"***

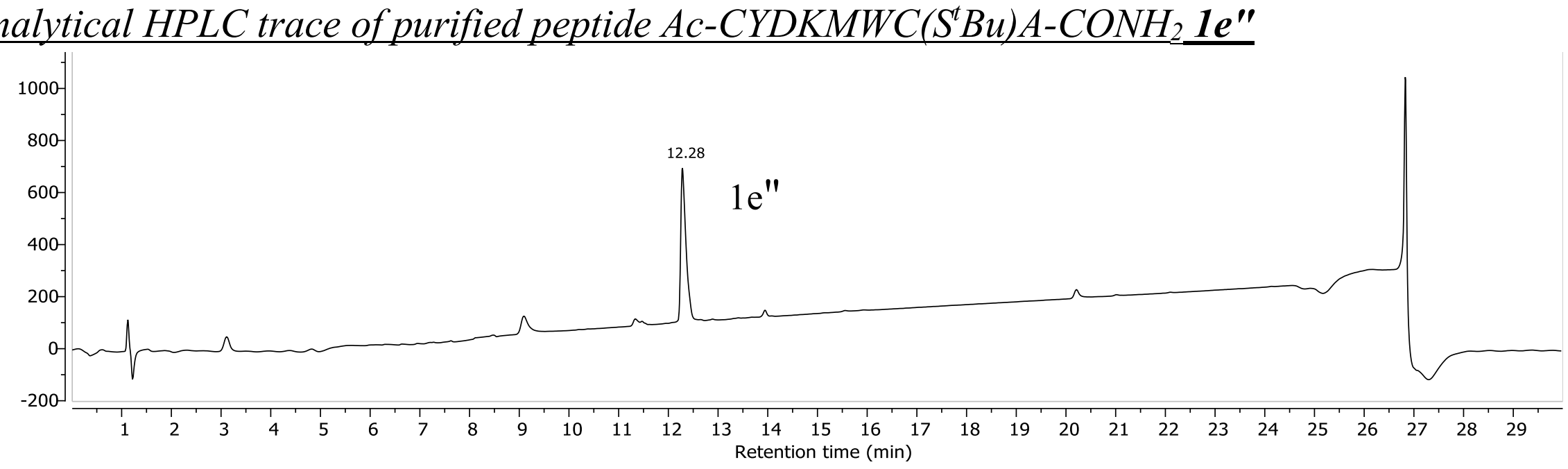

*HRMS spectrum of purified peptide Ac-CYDKMWC(S^{t}Bu)A-CONH$_2$ **1e"***

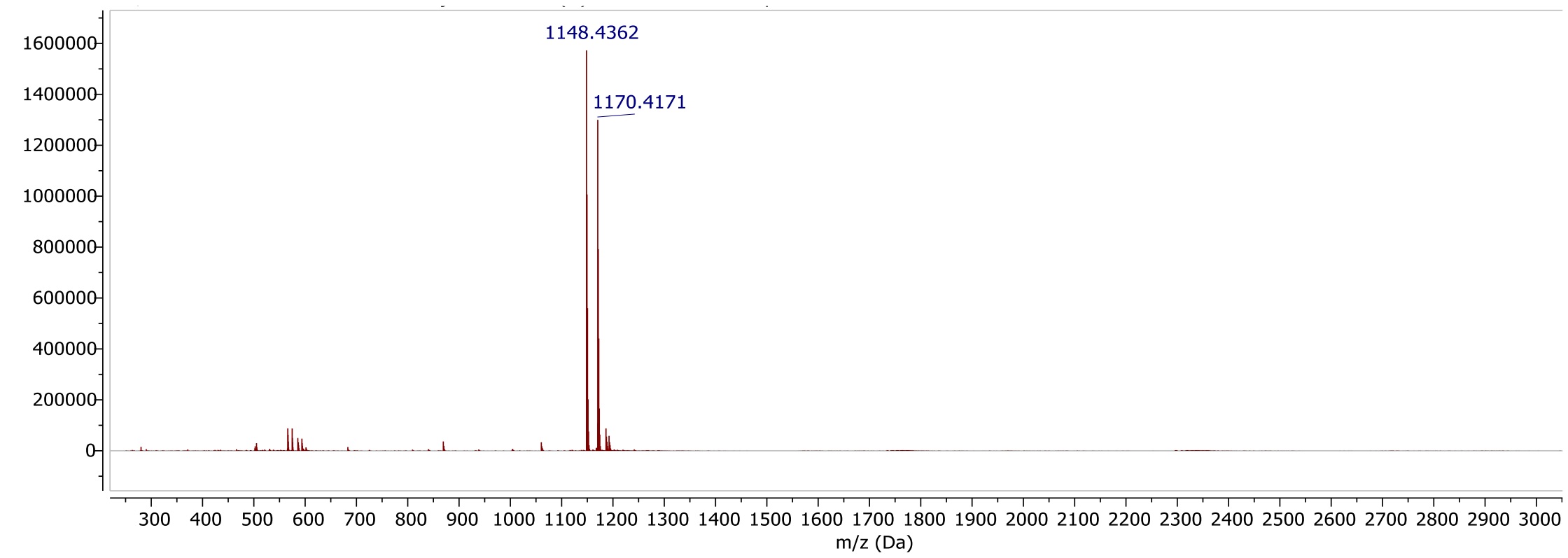

XII.b.2. Orthogonal Modification of Free Cys on Unprotected Peptide **1e"**.

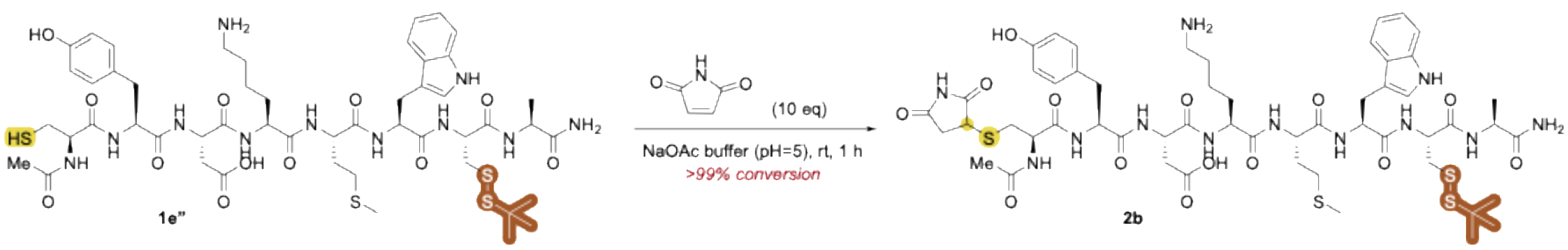

In a 1 dram vial equipped with a magnetic stirrer was added the purified peptide Ac-CYDKMWC(S^{t}Bu)A-CONH$_2$ **1e"** (3 mg, 0.0026 mmol, 1 eq) and it was dissolved in sodium acetate buffer (NaOAc buffer pH=5) (300 µL) while stirring. In a separate 1 dram vial, maleimide (2.5 mg, 0.026 mmol, 10 eq) was taken and dissolved in NaOAc buffer (200 µL) and the solution was added dropwise to the peptide solution and resulting mixture was allowed to stir 1 h at room temperature. After that, the selective maleimide adduct peptide **2b** (pale white solid, 3.2 mg, >99% conversion) was determined via analytical HPLC using 0.1% formic acid (FA) in both mobile phases A and B. Following analysis, product was subsequently purified using this same method.

Peptide Ac-C(maleimide)YDKMWC(S^{t}Bu)A-CONH$_2$ 2b: **HRMS (ESI)** m/z: $C_{54}H_{77}N_{12}O_{14}S_4$ $[M+H]^+$ Calcd for 1245.4560, Found 1245.4563. **Analytical HPLC Method** (C18, Water (A)/ACN (B); gradient: 5 to 85% B (0.1% formic acid in ACN) in 20 min, flow rate = 1.0 mL/min, I = 220 nm)**:** retention time of 11.80 min.

*Analytical HPLC trace of the crude reaction mixture converting Ac-CYDKMWC(S^{t}Bu)A-CONH$_2$ **1e"** to Ac-C(maleimide)YDKMWC(S^{t}Bu)A-CONH$_2$ **2b***

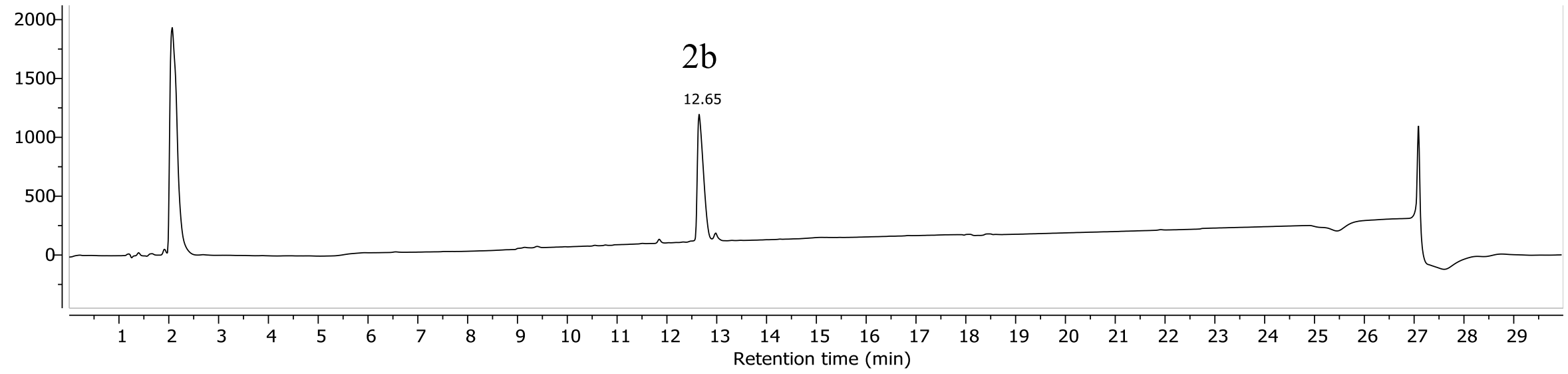

Analytical HPLC trace of purified peptide Ac-C(maleimide)YDKMWC(S^{t}Bu)A-CONH$_2$ ***2b***

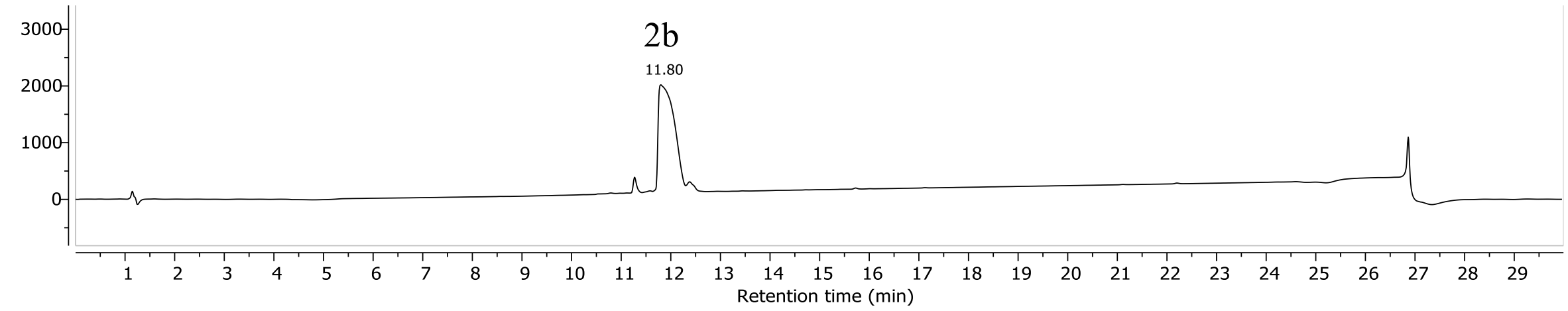

HRMS spectrum of purified peptide Ac-C(maleimide)YDKMWC(S^{t}Bu)A-CONH$_2$ ***2b***

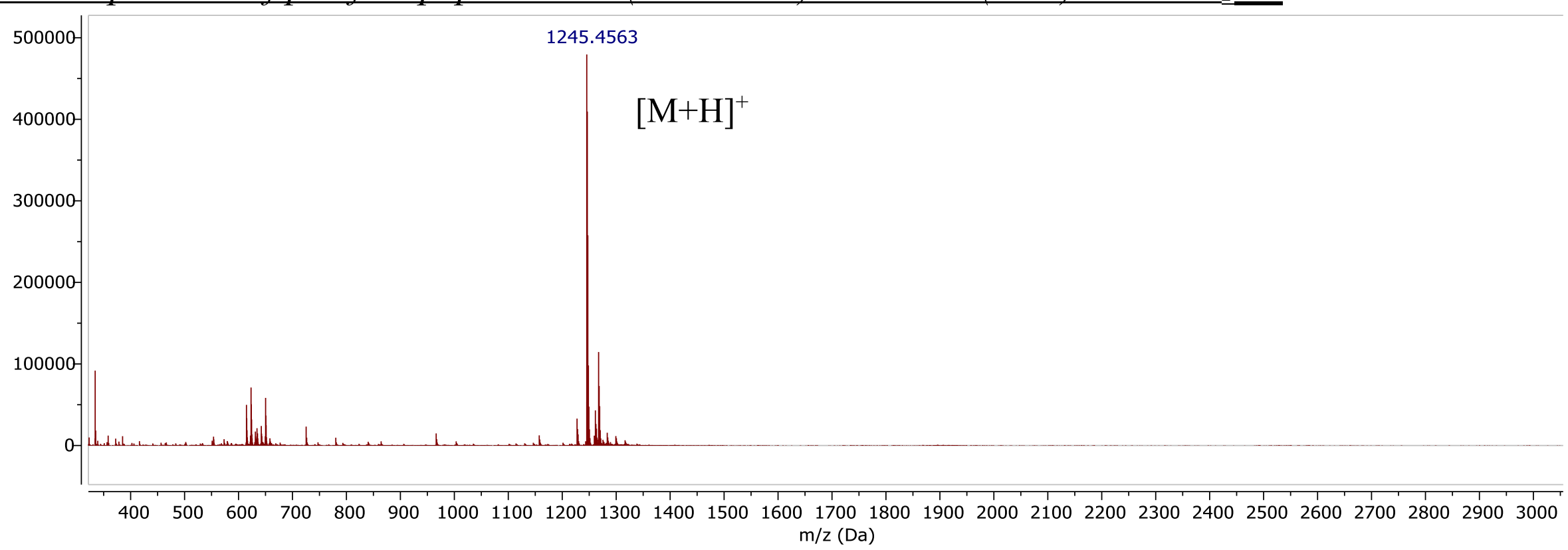

XII.b.3. Orthogonal Cleavage of *tert*-Butyl Containing Disulfide on Peptide **2b**.

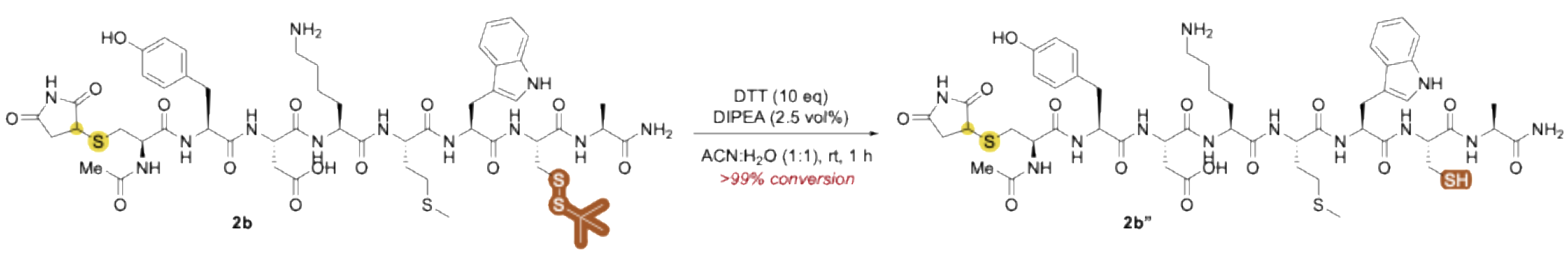

In a 1 dram vial equipped with a magnetic stirrer was added the purified peptide Ac-C(maleimide)YDKMWC(S^{t}Bu)A-CONH$_2$ **2b** (3 mg, 0.0024 mmol, 1 eq) and dissolved in ACN (500 µL), and H_2O (200 µL) while stirring. In a separate 1 dram vial, dithiothreitol (DTT) (3.7 mg, 0.024 mmol, 10 eq) was taken and dissolved in H_2O (300 µL) and the solution was added dropwise to the peptide solution followed by addition of *N,N*-diisopropylethylamine (DIPEA, 25 µL) at once and resulting mixture was allowed to stir 1 h at room temperature. After that, the selective reduced thiol product **2b"** (pale white solid, 2.7 mg, >99% conversion) was determined via analytical HPLC using 0.1% formic acid (FA) in both mobile phases A and B. Following analysis, product was subsequently purified using this same method.

Peptide Ac-C(maleimide)YDKMWCA-CONH$_2$ 2b": **HRMS (ESI)** m/z: $C_{50}H_{69}N_{12}O_{14}S_3$ $[M+H]^+$ Calcd for 1157.4213, Found 1157.4246; $[M + 2H]^{+2}$ Calcd for 579. 2070, Found 579.2158. **Analytical HPLC Method** (C18, Water (A)/ACN (B); gradient: 5 to 85% B (0.1% formic acid in ACN) in 20 min, flow rate = 1.0 mL/min, I = 220 nm)**:** retention time of 10.12 min.

*Analytical HPLC trace of the crude reaction mixture converting Ac-C(maleimide)YDKMWC(S^{t}Bu)A-CONH$_2$ **2b** to Ac-C(maleimide)YDKMWCA-CONH$_2$ **2b''***

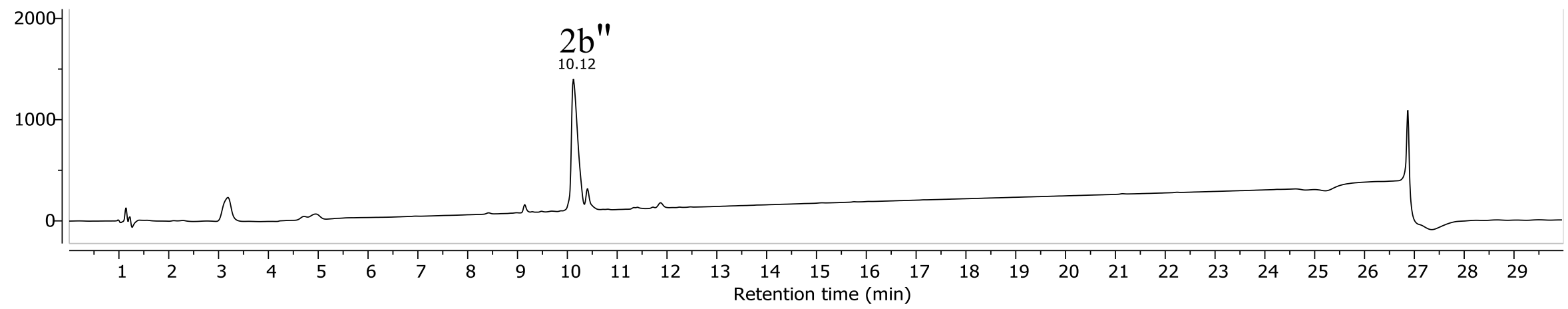

*Analytical HPLC trace of purified peptide Ac-C(maleimide)YDKMWCA-CONH$_2$ **2b''***

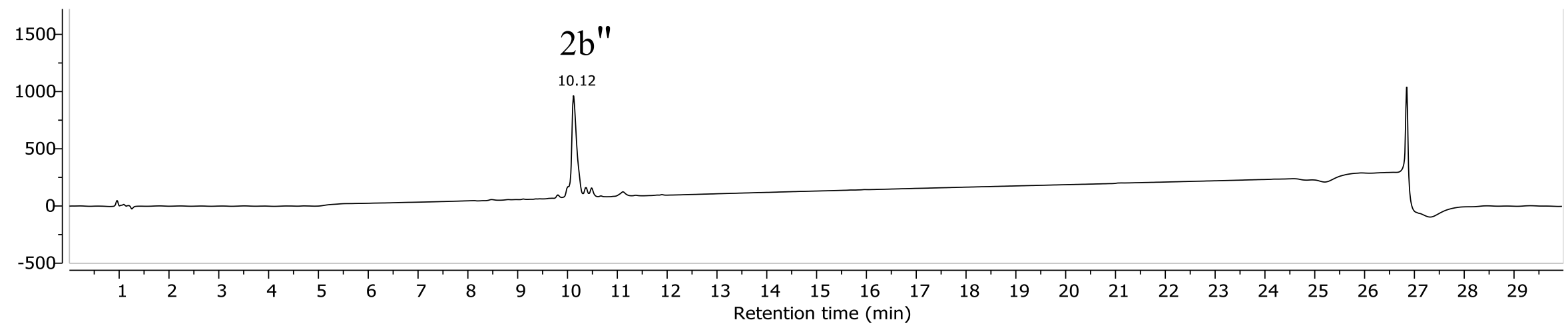

*HRMS spectrum of purified peptide Ac-C(maleimide)YDKMWCA-CONH$_2$ **2b''***

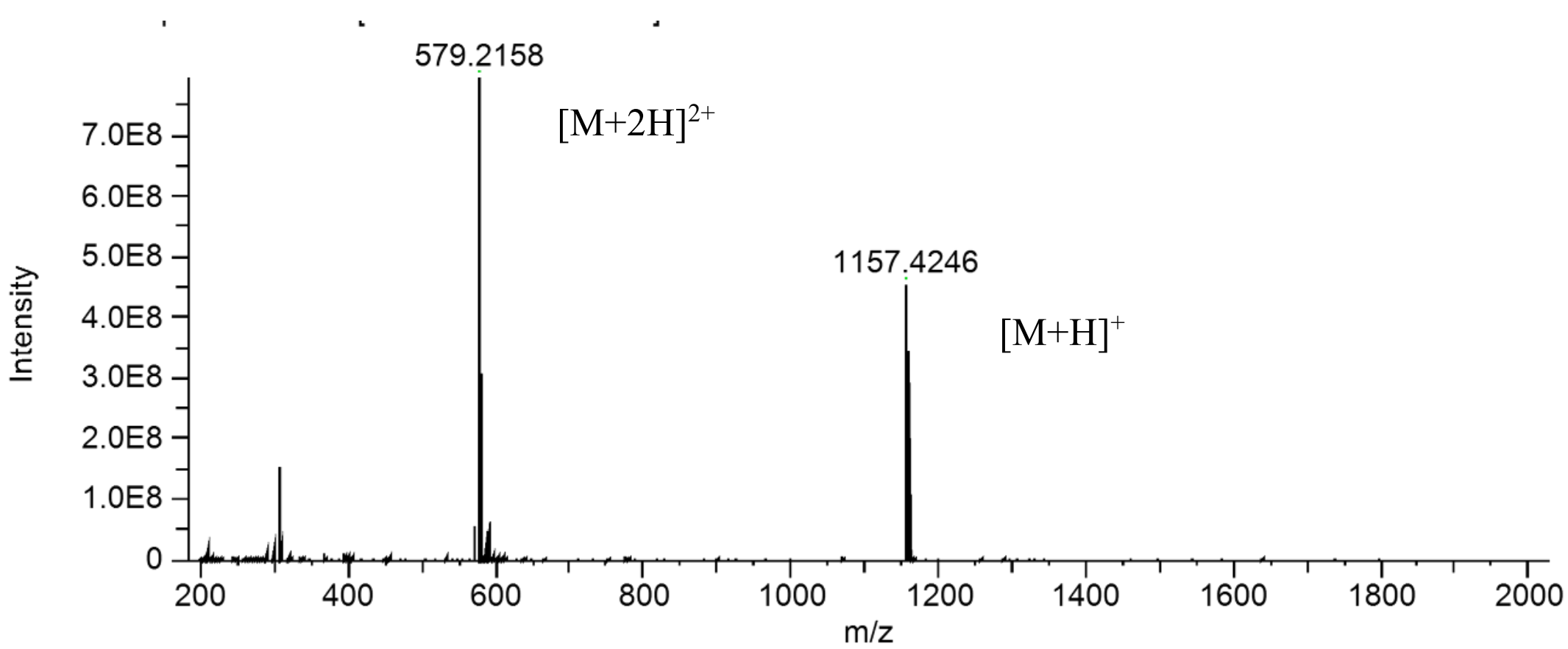

XII.b.4. Orthogonal Modification of Free Cys on Unprotected Peptide **2b''**.

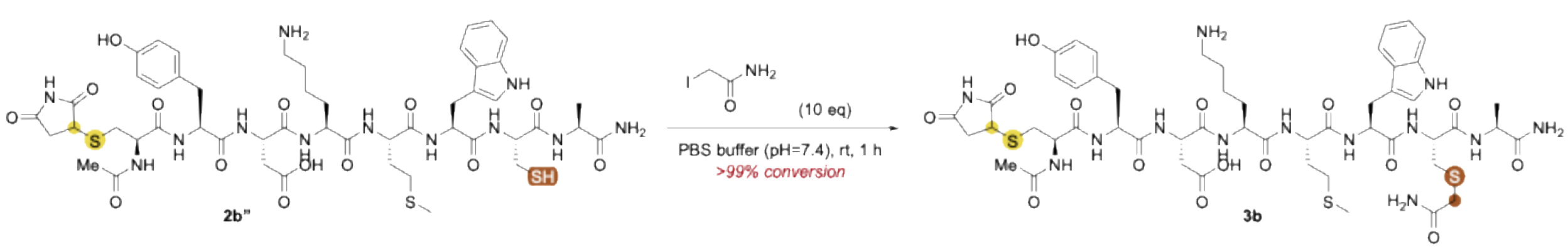

In a 1 dram vial equipped with a magnetic stirrer was added the purified peptide Ac-C(maleimide)YDKMWCA-CONH$_2$ **2b''** (2 mg, 0.0017 mmol, 1 eq) and it was dissolved in PBS buffer (pH=7.4) (300 µL) while stirring. In a separate 1 dram vial, iodoacetamide (3.2 mg, 0.017 mmol, 10 eq) was taken and dissolved in PBS buffer (200 µL) and the solution was added dropwise to the peptide solution and resulting mixture was allowed to stir 1 h at room temperature. After that, the selective iodoacetamide adduct peptide **3b** (pale white solid, 2 mg, >99% conversion) was determined via analytical HPLC using

0.1% formic acid (FA) in both mobile phases A and B. Following analysis, product was subsequently purified using this same method.

Peptide Ac-C(maleimide)YDKMWC(iodoacetamide)A-CONH₂ 3b: **HRMS (ESI)** m/z: $C_{52}H_{72}N_{13}O_{15}S_3$ $[M+H]^+$ Calcd for 1214.4427, Found 1214.4424. **Analytical HPLC Method** (C18, Water (A)/ACN (B); gradient: 5 to 85% B (0.1% formic acid in ACN) in 20 min, flow rate = 1.0 mL/min, I = 220 nm)**:** retention time of 9.36 min.

*Analytical HPLC trace of the crude reaction mixture converting Ac-C(maleimide)YDKMWCA-CONH$_2$ **2b''** to Ac-C(maleimide)YDKMWC(iodoacetamide)A-CONH$_2$ **3b***

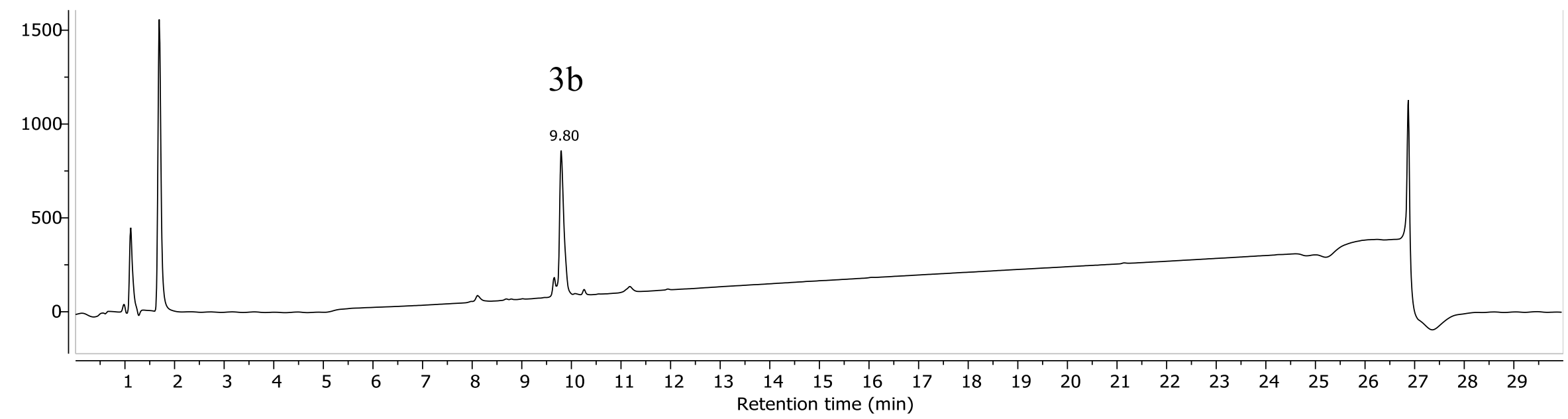

*Analytical HPLC trace of purified peptide Ac-C(maleimide)YDKMWC(iodoacetamide)A-CONH$_2$ **3b***

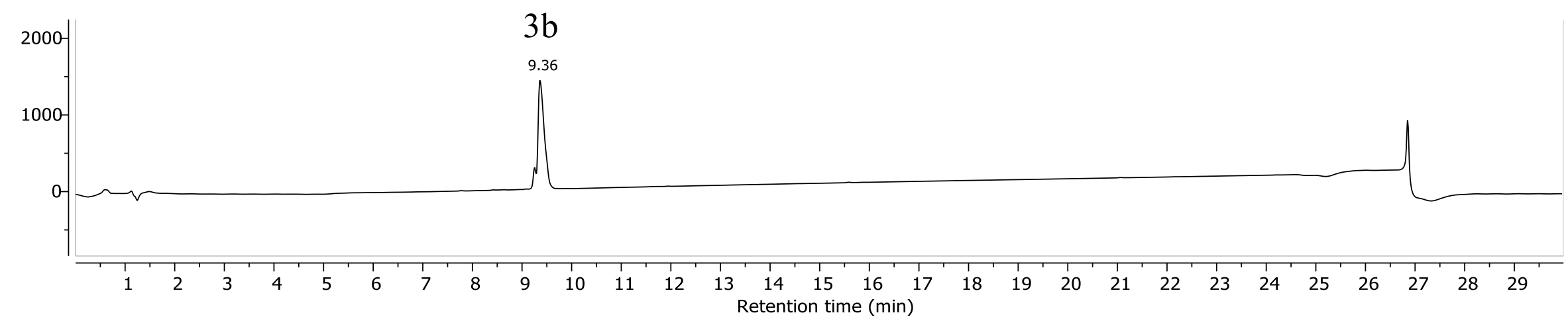

*HRMS spectrum of purified peptide Ac-C(maleimide)YDKMWC(iodoacetamide)A-CONH$_2$ **3b***

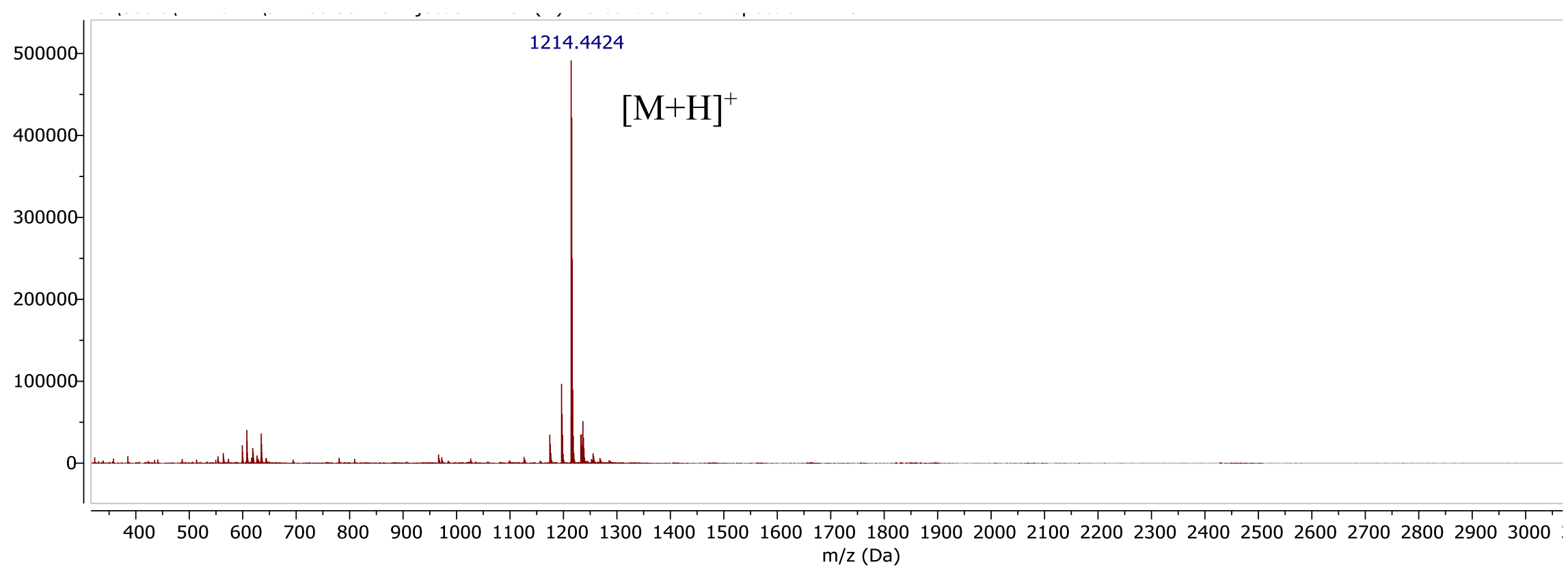

XII.b.5. Orthogonal Cleavage and Modification of Disulfide in Chemo-selective, Position-independent, Non-aromatic Residue Containing Peptide

Synthesis of Aryl-disulfide Containing Peptide:

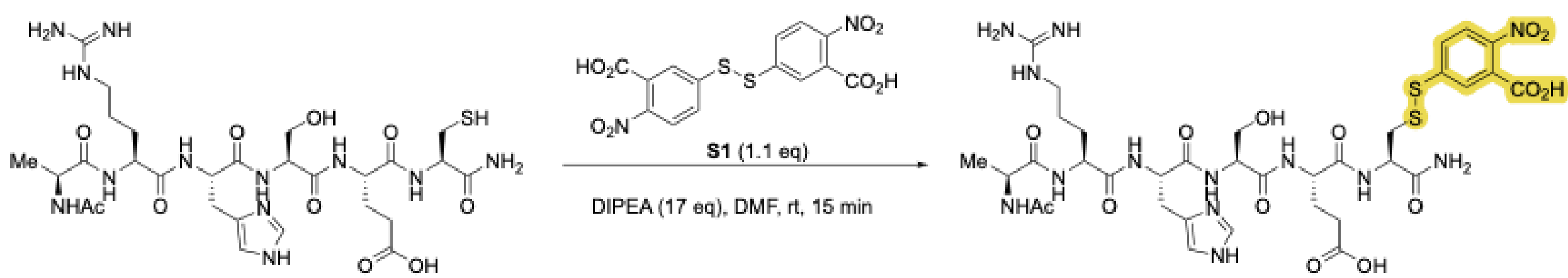

In a 1 dram vial equipped with a magnetic stirrer was added the purified peptide Ac-ARHSEC-CONH$_2$ (3.1 mg, 0.0041 mmol, 1 eq), *N,N*-diisopropylethylamine (DIPEA, 12 µL, 0.69 mmol, 17 eq) and *N,N*-dimethylformamide (DMF, 700 µL), while stirring. In a separate 1 dram vial, 4,4'-disulfanediylbis-2-nitrobenzoic acid **S1** (1.8 mg, 0.0045 mmol, 1.1 eq) was taken and dissolved in DMF (300 µL) and the solution was added dropwise to the peptide solution and resulting mixture was allowed to stir 15 min at room temperature. After that, conversion of the aryl-disulfide containing peptide Ac-ARHSEC(SAr^2)-CONH$_2$ was determined via analytical HPLC using 0.1% formic acid (FA) in both mobile phases A and B (pale white solid, 3.8 mg, >99% conversion).

Peptide Ac-ARHSEC(SAr^2)-CONH$_2$: **HRMS (ESI)** m/z: $C_{35}H_{50}N_{13}O_{14}S_2$ $[M+H]^+$ Calcd for 940.3036, Found 940.3024; $[M+2H]^{2+}$ Calcd for 470.6481, Found 470.6552. **Analytical HPLC Method** (C18, Water (A)/ACN (B); gradient: 0 to 80% B (0.1% formic acid in ACN) in 40 min, flow rate = 1.0 mL/min, I = 220 nm): retention time of 8.08 min.

Analytical HPLC trace of purified peptide Ac-ARHSEC(SAr^2)-CONH$_2$

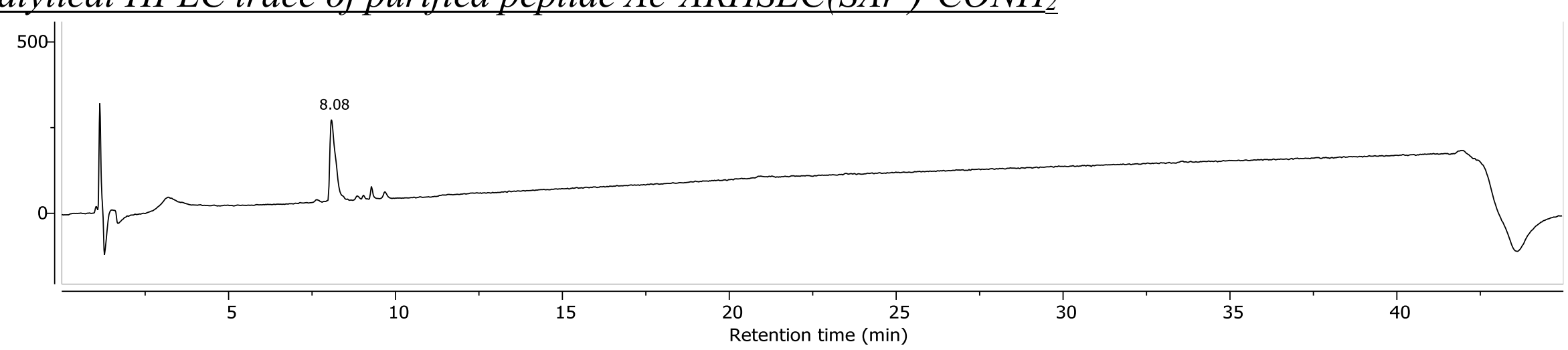

HRMS spectrum of purified peptide Ac-ARHSEC(SAr^2)-CONH$_2$

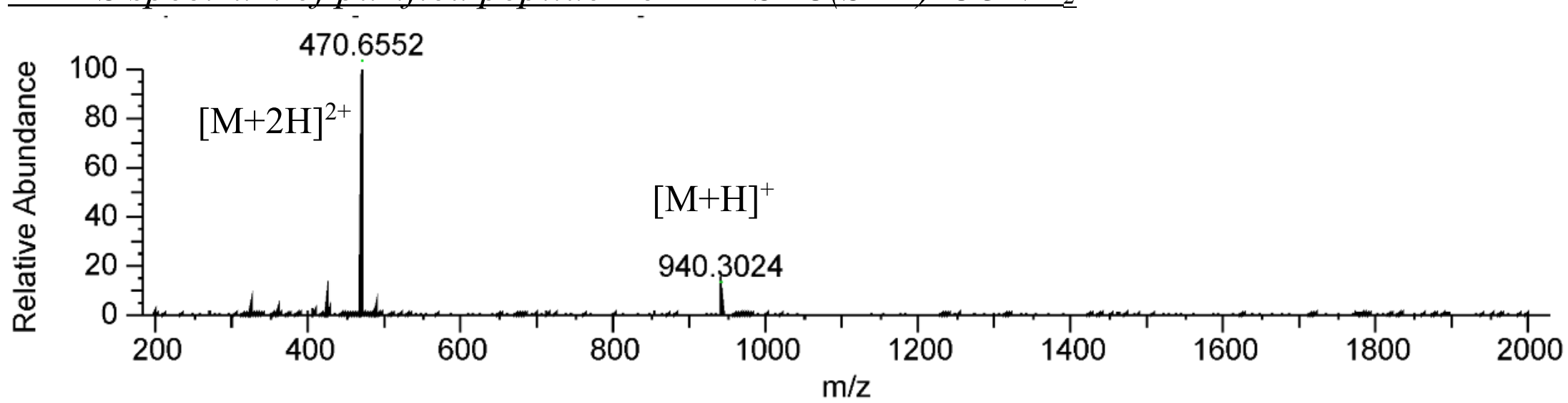

Orthogonal Cleavage of Aryl Containing Disulfide:

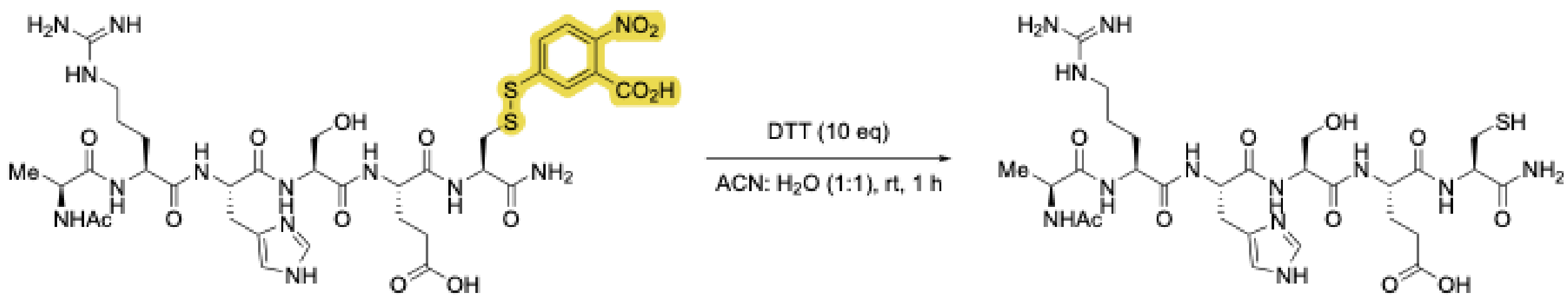

In a 1 dram vial equipped with a magnetic stirrer was added the purified peptide Ac-ARHSEC(SAr^2)-CONH$_2$ (3 mg, 0.004 mmol, 1 eq) and it was dissolved in ACN (500 µL), and H_2O (200 µL) while stirring. In a separate 1 dram vial, dithiothreitol (DTT) (6.2 mg, 0.04 mmol, 10 eq) was taken and dissolved in H_2O (300 µL) and the solution was added dropwise to the peptide solution and resulting mixture was allowed to stir 1 h at room temperature. After that, the selective reduced thiol product Ac-ARHSEC-CONH$_2$ (pale white solid, 2.9 mg, >99% conversion) was determined via analytical HPLC using 0.1% formic acid (FA) in both mobile phases A and B. Following analysis, product was subsequently purified using this same method.

Peptide Ac-ARHSEC-CONH$_2$: **HRMS (ESI)** m/z: $C_{28}H_{47}N_{12}O_{10}S$ $[M+H]^+$ Calcd for 743.3253, Found 743.3273; $[M+2H]^{2+}$ Calcd for 372.1590, Found 372.1664. **Analytical HPLC Method** (C18, Water (A)/ACN (B); gradient: 0 to 80% B (0.1% formic acid in ACN) in 40 min, flow rate = 1.0 mL/min, I = 220 nm): retention time of 4.24 min.

Analytical HPLC trace of the crude reaction mixture converting Ac-ARHSEC(SAr^2)-CONH$_2$ to Ac-ARHSEC-CONH$_2$

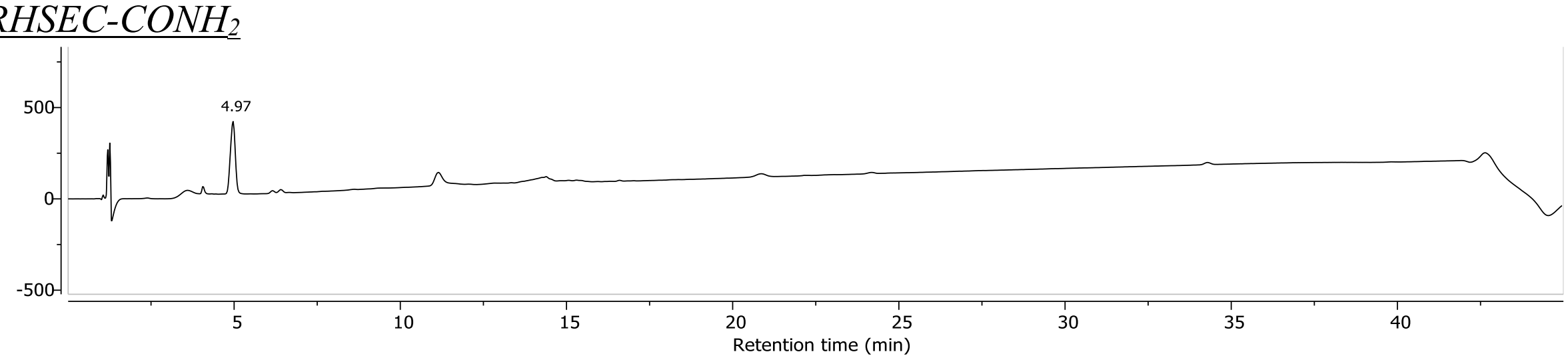

Analytical HPLC trace of purified peptide Ac-ARHSEC-CONH$_2$

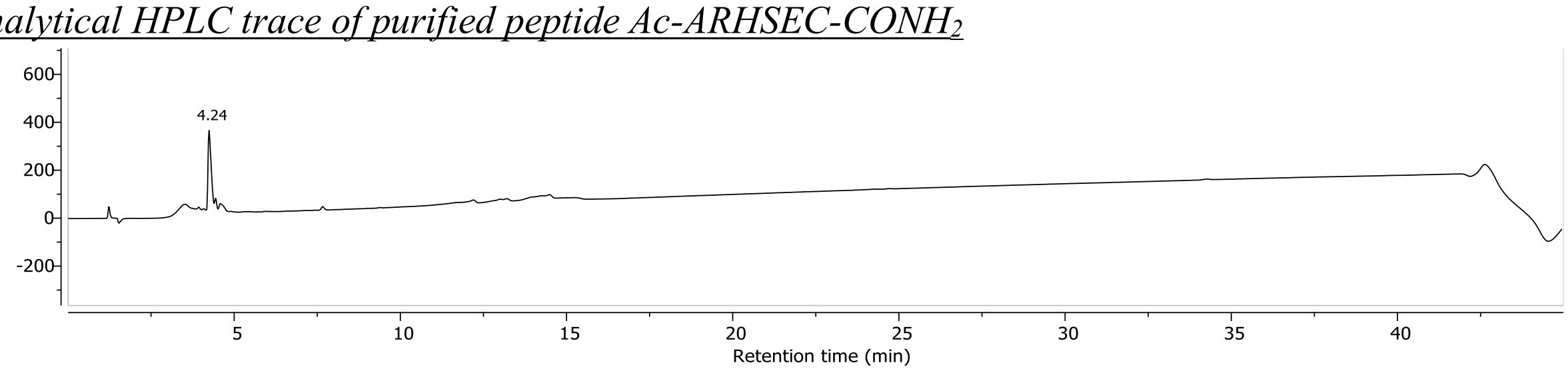

HRMS spectrum of purified peptide Ac-ARHSEC-CONH$_2$

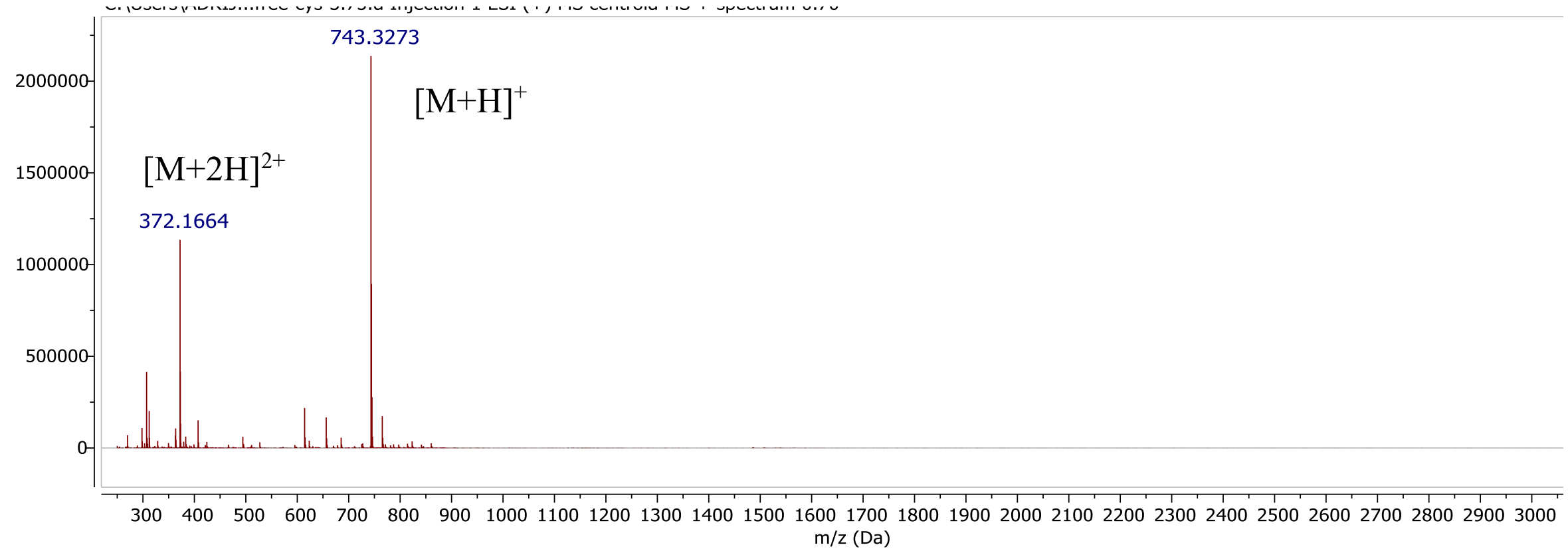

Orthogonal Modification of Free Cys:

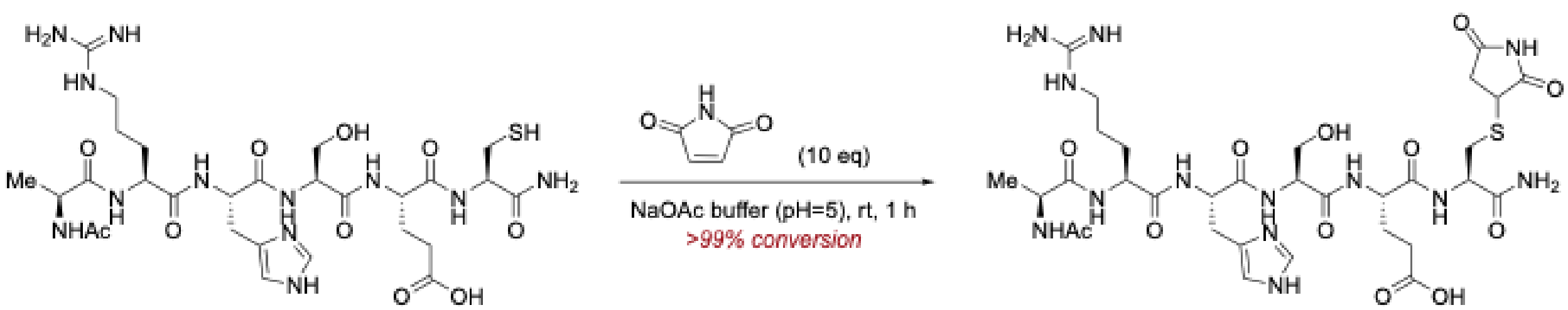

In a 1 dram vial equipped with a magnetic stirrer was added the purified peptide Ac-ARHSEC-CONH$_2$ (3 mg, 0.004 mmol, 1 eq) and it was dissolved in sodium acetate buffer (NaOAc buffer pH=5) (300 µL) while stirring. In a separate 1 dram vial, maleimide (3.9 mg, 0.04 mmol, 10 eq) was taken and dissolved in NaOAc buffer (200 µL) and the solution was added dropwise to the peptide solution and resulting mixture was allowed to stir 1 h at room temperature. After that, the selective maleimide adduct peptide Ac-ARHSEC(maleimide)-CONH$_2$ (pale white solid, 3.3 mg, >99% conversion) was determined via analytical HPLC using 0.1% formic acid (FA) in both mobile phases A and B. Following analysis, product was subsequently purified using this same method.

Peptide Ac-ARHSEC(maleimide)-CONH$_2$: **HRMS (ESI)** m/z: $C_{32}H_{50}N_{13}O_{12}S$ $[M+H]^+$ Calcd for 840.3417, Found 840.3400; $[M+2H]^{2+}$ Calcd for 420.6672, Found 420.6740. **Analytical HPLC Method** (C18, Water (A)/ACN (B); gradient: 0 to 80% B (0.1% formic acid in ACN) in 40 min, flow rate = 1.0 mL/min, I = 220 nm): retention time of 4.35 min.

Analytical HPLC trace of the crude reaction mixture converting Ac-ARHSEC-CONH$_2$ to Ac-ARHSEC(maleimide)-CONH$_2$

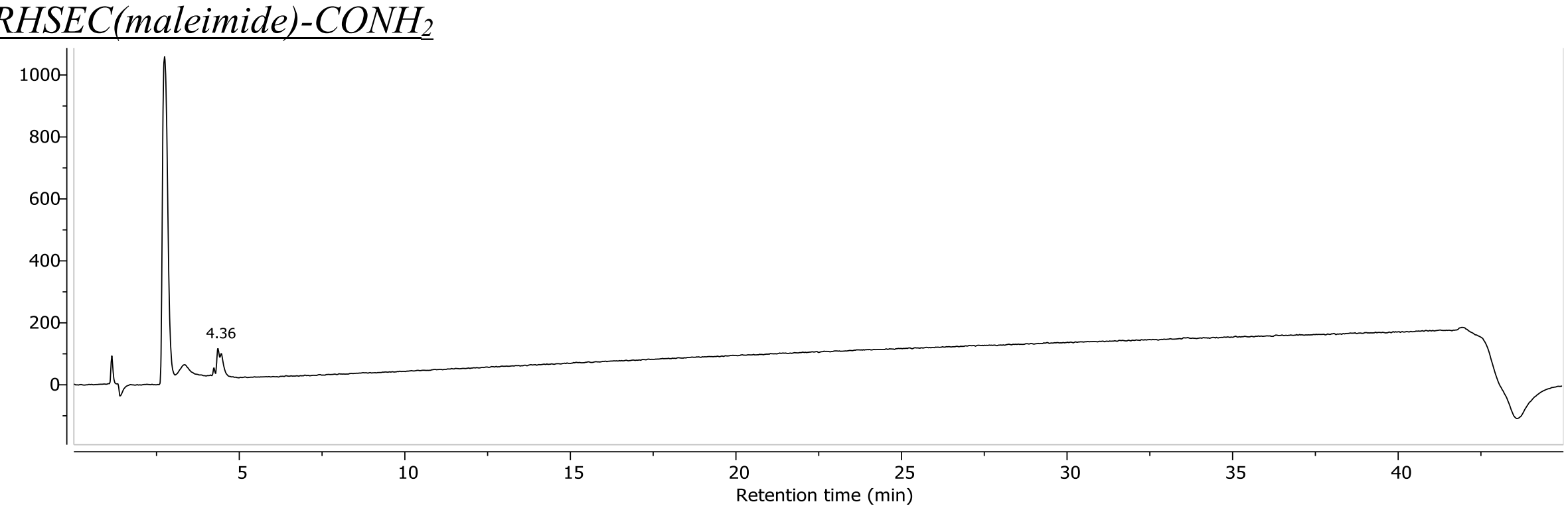

Analytical HPLC trace of purified peptide Ac-ARHSEC(maleimide)-CONH$_2$

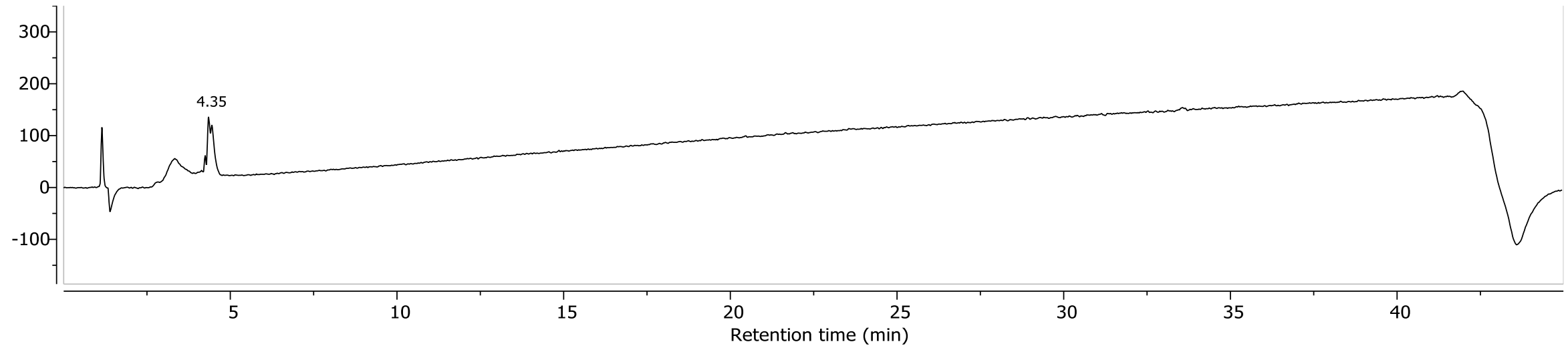

HRMS spectrum of purified peptide Ac-ARHSEC(maleimide)-CONH$_2$

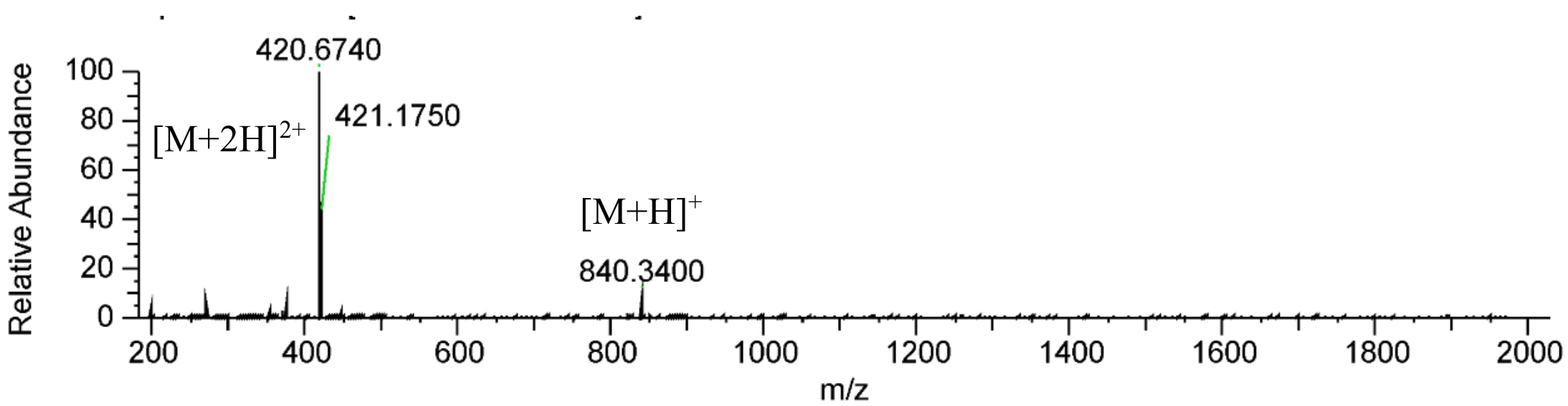

XII.c. Supplementary Figure 4. Orthogonal Cleavage and Modification of Ethyl and *tert*-Butyl Containing Disulfides in Fully Unprotected Peptide **1f**.

XII.c.1. Orthogonal Cleavage of Ethyl Containing Disulfide on Peptide **1f**.

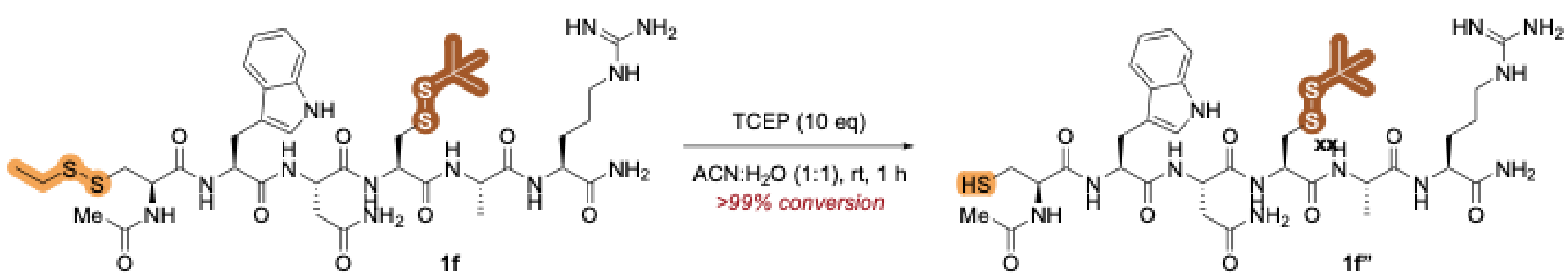

In a 1 dram vial equipped with a magnetic stirrer was added the purified peptide Ac-C(SEt)WNC(S^{t}Bu)AR-CONH$_2$ **1f** (4 mg, 0.0042 mmol, 1 eq) and it was dissolved in ACN (500 µL), and H_2O (200 µL) while stirring. In a separate 1 dram vial, tris(2-carboxyethyl)phosphine hydrochloride (TCEP) (12 mg, 0.042 mmol, 10 eq) was taken and dissolved in H_2O (300 µL) and the solution was added dropwise to the peptide solution and resulting mixture was allowed to stir 1 h at room temperature. After that, the selective reduced thiol product Ac-CWNC(S^{t}Bu)AR-CONH$_2$ **1f''** (pale white solid, 3.6 mg, >99% conversion) was determined via analytical HPLC using 0.1% formic acid (FA) in both mobile phases A and B. Following analysis, product was subsequently purified using this same method.

Peptide Ac-CWNC(S^{t}Bu)AR-CONH$_2$ 1f'': **HRMS (ESI)** m/z: $C_{36}H_{57}N_{12}O_8S_3$ $[M+H]^+$ Calcd for 881.3579, Found 881.3602; $[M+2H]^{+2}$ Calcd for 441.1753, Found 441.1837. **Analytical HPLC Method** (C18, Water (A)/ACN (B); gradient: 5 to 85% B (0.1% formic acid in ACN) in 20 min, flow rate = 1.0 mL/min, I = 220 nm)**:** retention time of 11.43 min.

*Analytical HPLC trace of the crude reaction mixture converting Ac-C(SEt)WNC(S^{t}Bu)AR-CONH$_2$ **1f** to Ac-CWNC(S^{t}Bu)AR-CONH$_2$ **1f''***

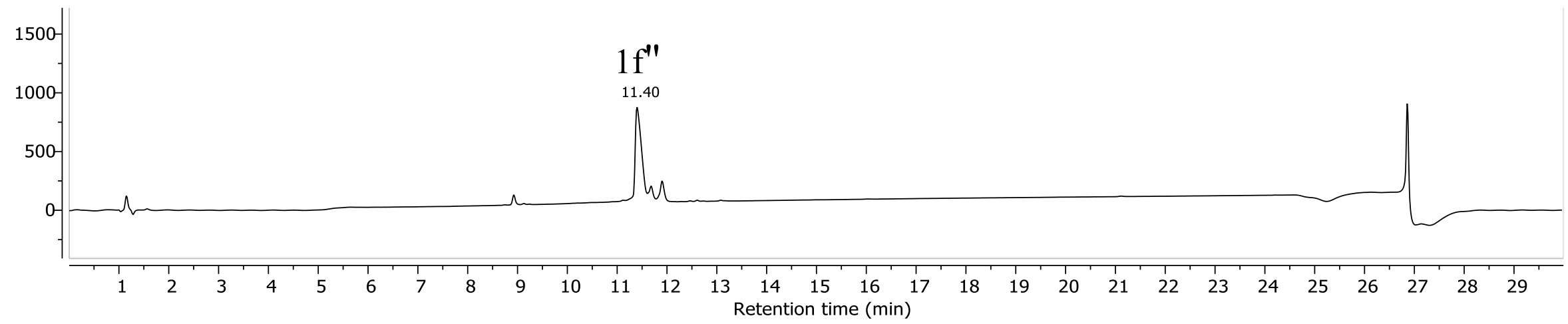

*Analytical HPLC trace of purified peptide Ac-CWNC(S^{t}Bu)AR-CONH$_2$ **1f''***

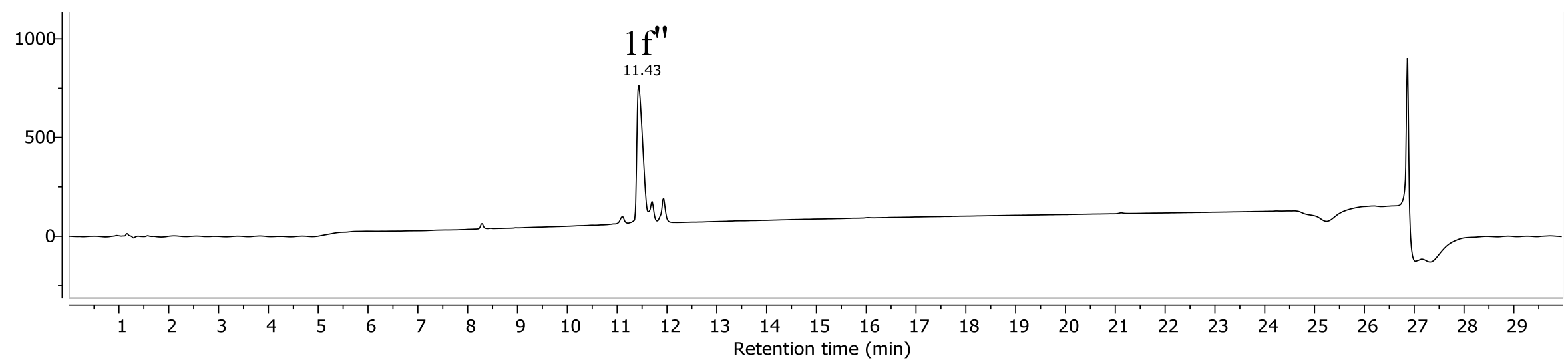

*HRMS spectrum of purified peptide Ac-CWNC(S^{t}Bu)AR-CONH$_2$ **1f''***

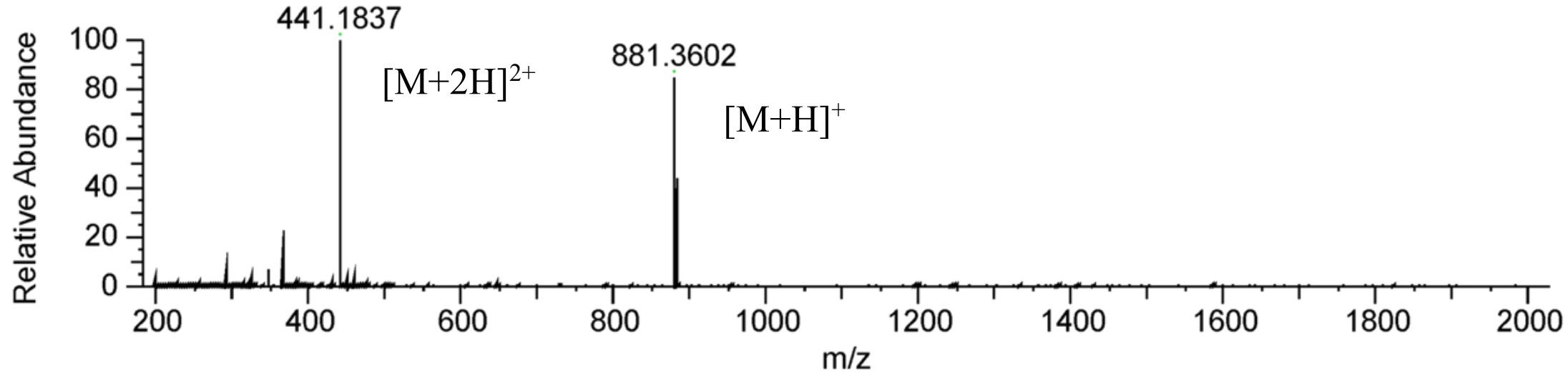

XII.c.2. Orthogonal Modification of Free Cys on Unprotected Peptide **1f''**.

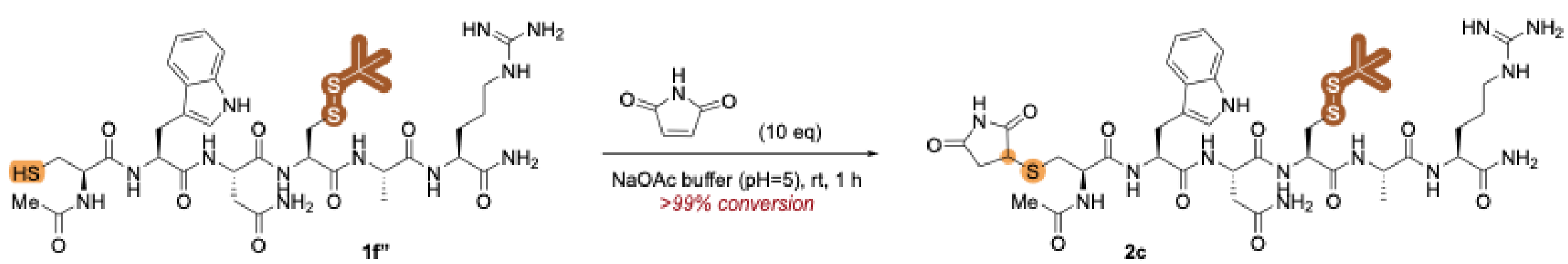

In a 1 dram vial equipped with a magnetic stirrer was added the purified peptide Ac-CWNC(S^{t}Bu)AR-CONH$_2$ **1f''** (3 mg, 0.0034 mmol, 1 eq) and it was dissolved in sodium acetate buffer (NaOAc buffer pH=5) (300 µL) while stirring. In a separate 1 dram vial, maleimide (3.3 mg, 0.034 mmol, 10 eq) was taken and dissolved in NaOAc buffer (200 µL) and the solution was added dropwise to the peptide solution and resulting mixture was allowed to stir 1 h at room temperature. After that, the selective maleimide adduct peptide **2c** (pale white solid, 3.3 mg, >99% conversion) was determined via analytical HPLC using 0.1% formic acid (FA) in both mobile phases A and B. Following analysis, product was subsequently purified using this same method.

Peptide Ac-C(maleimide)WNC(S^{t}Bu)AR-CONH$_2$ 2c: **HRMS (ESI)** m/z: $C_{40}H_{60}N_{13}O_{10}S_3$ $[M+H]^+$ Calcd for 978.3743, Found 978.3749. **Analytical HPLC Method** (C18, Water (A)/ACN (B); gradient: 5 to 85% B (0.1% formic acid in ACN) in 20 min, flow rate = 1.0 mL/min, I = 220 nm)**:** retention time of 10.91 min.

*Analytical HPLC trace of the crude reaction mixture converting Ac-CWNC(S^{t}Bu)AR-CONH$_2$ **1f''** to Ac-C(maleimide)WNC(S^{t}Bu)AR-CONH$_2$ **2c***

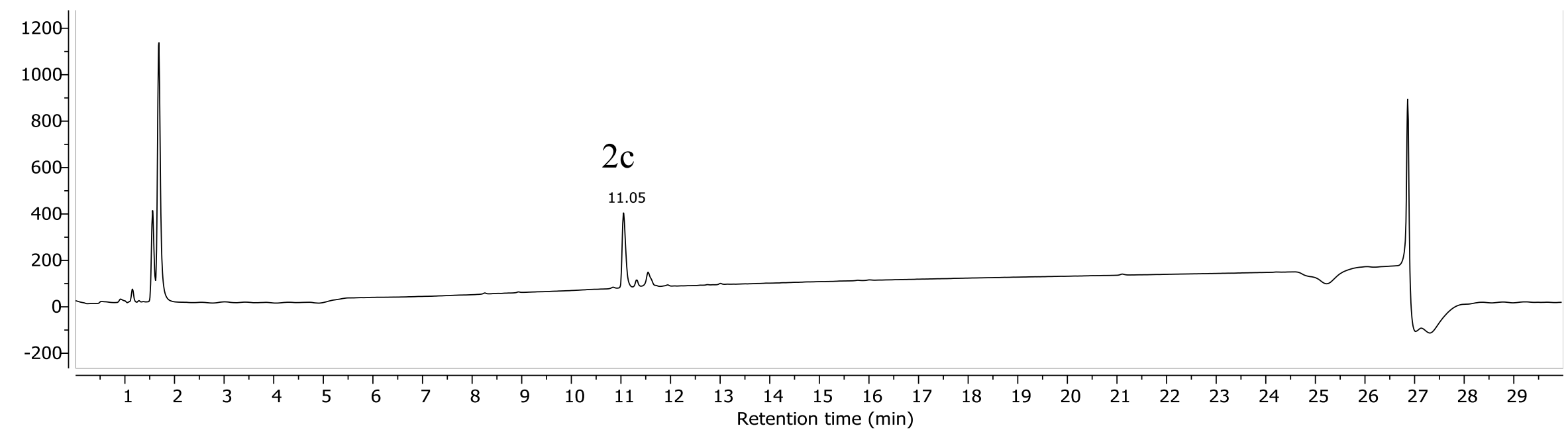

*Analytical HPLC trace of purified peptide Ac-C(maleimide)WNC(S^{t}Bu)AR-CONH$_2$ **2c***

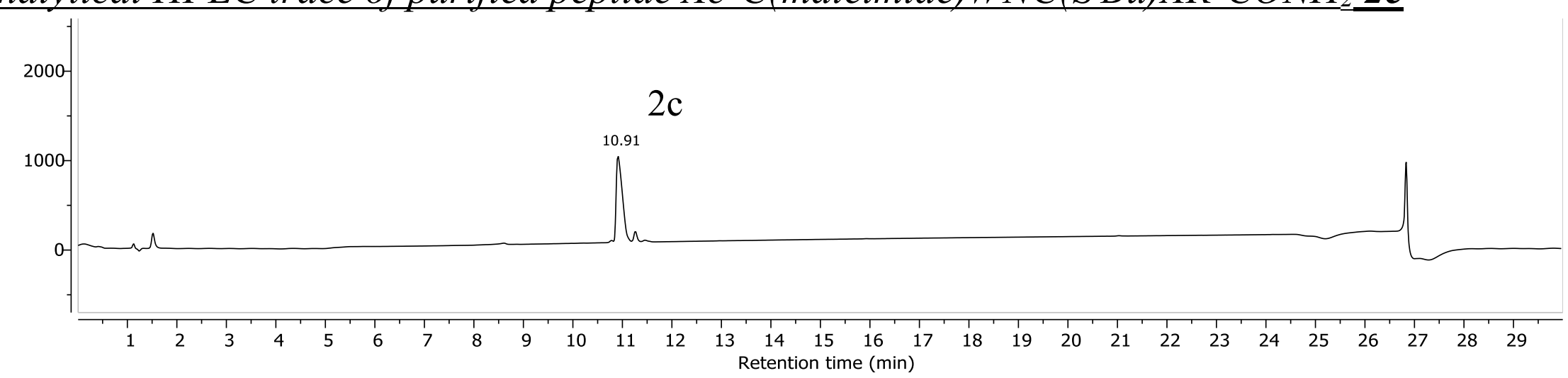

*HRMS spectrum of purified peptide Ac-C(maleimide)WNC(S^{t}Bu)AR-CONH$_2$ **2c***

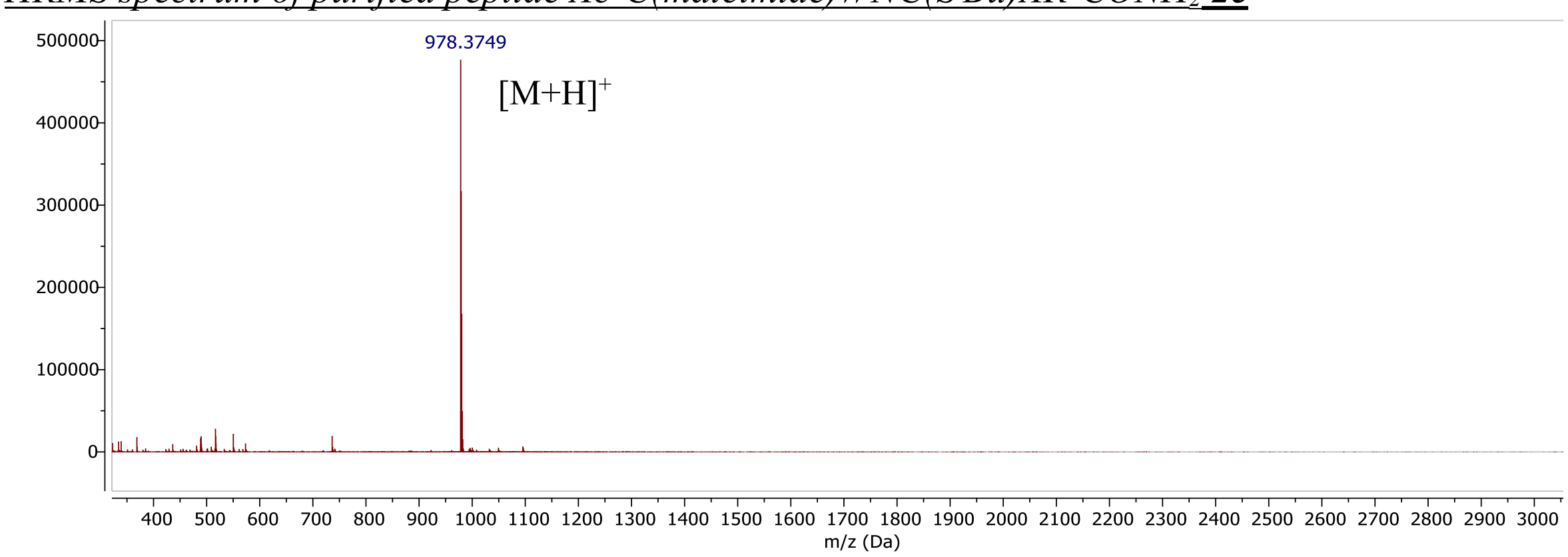

XII.c.3. Orthogonal Cleavage of *tert*-Butyl Containing Disulfide on Peptide **2c**.

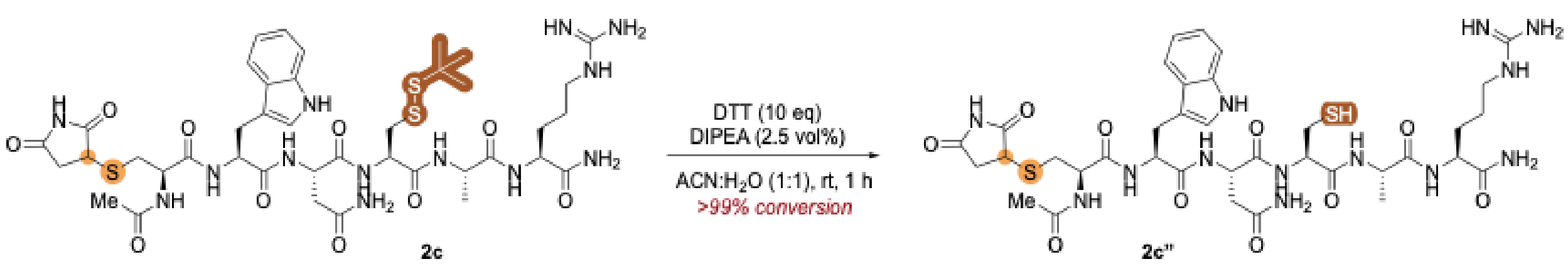

In a 1 dram vial equipped with a magnetic stirrer was added the purified peptide Ac-C(maleimide)WNC(S^{t}Bu)AR-CONH$_2$ **2c** (3 mg, 0.0030 mmol, 1 eq) and dissolved in ACN (500 µL), and

H_2O (200 µL) while stirring. In a separate 1 dram vial, dithiothreitol (DTT) (4.7 mg, 0.030 mmol, 10 eq) was taken and dissolved in H_2O (300 µL) and the solution was added dropwise to the peptide solution followed by addition of *N,N*-diisopropylethylamine (DIPEA, 25 µL) at once and resulting mixture was allowed to stir 1 h at room temperature. After that, the selective reduced thiol product **2c"** (pale white solid, 2.6 mg, >99% conversion) was determined via analytical HPLC using 0.1% formic acid (FA) in both mobile phases A and B. Following analysis, product was subsequently purified using this same method.

Peptide Ac-C(maleimide)WNCAR-CONH$_2$ 2c": HRMS (ESI) m/z: $C_{36}H_{52}N_{13}O_{10}S_2$ $[M+H]^+$ Calcd for 890.3396, Found 890.3357. **Analytical HPLC Method** (C18, Water (A)/ACN (B); gradient: 5 to 85% B (0.1% formic acid in ACN) in 20 min, flow rate = 1.0 mL/min, I = 220 nm)**:** retention time of 8.50 min.

*Analytical HPLC trace of the crude reaction mixture converting Ac-C(maleimide)WNC(S^{t}Bu)AR-CONH$_2$ **2c** to Ac-C(maleimide)WNCAR-CONH$_2$ **2c".***

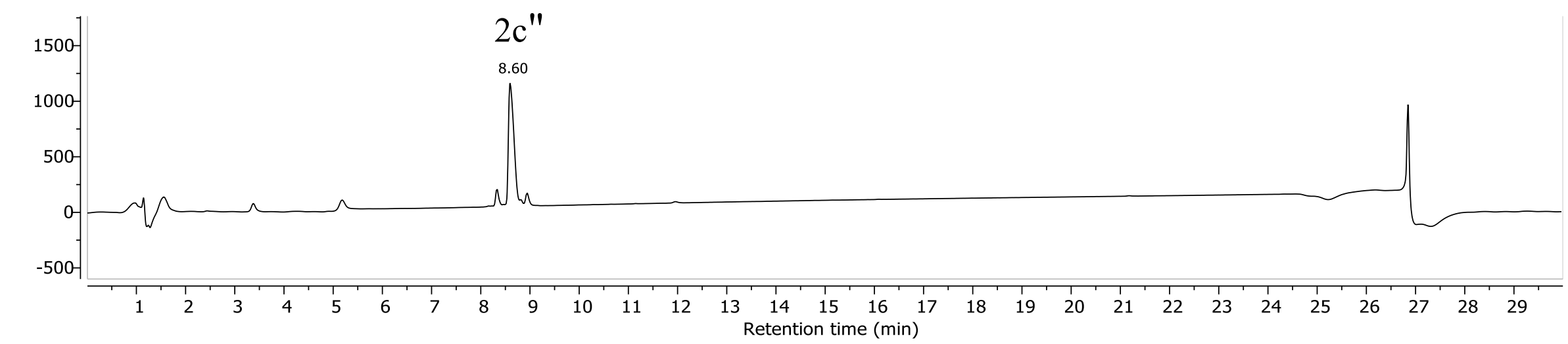

*Analytical HPLC trace of purified peptide Ac-C(maleimide)WNCAR-CONH$_2$ **2c"***

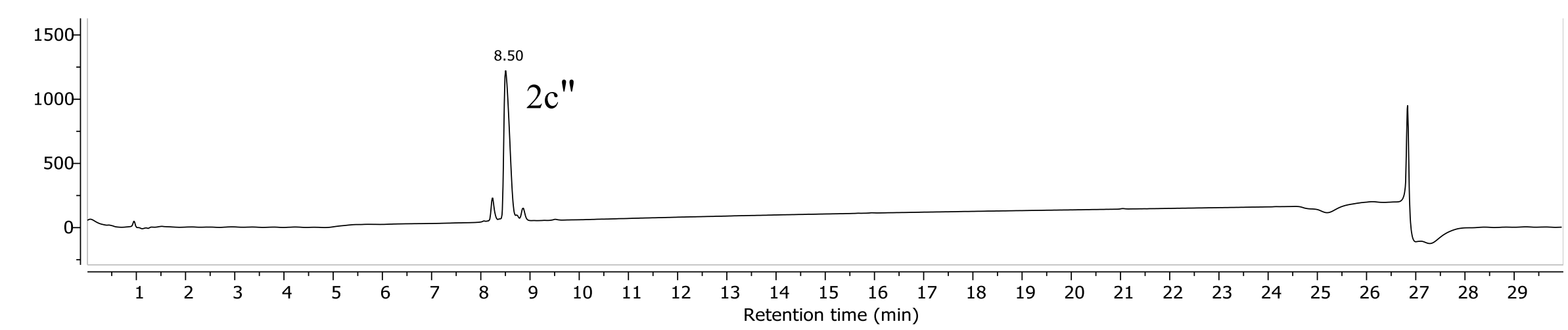

*HRMS spectrum of purified peptide Ac-C(maleimide)WNCAR-CONH$_2$ **2c"***

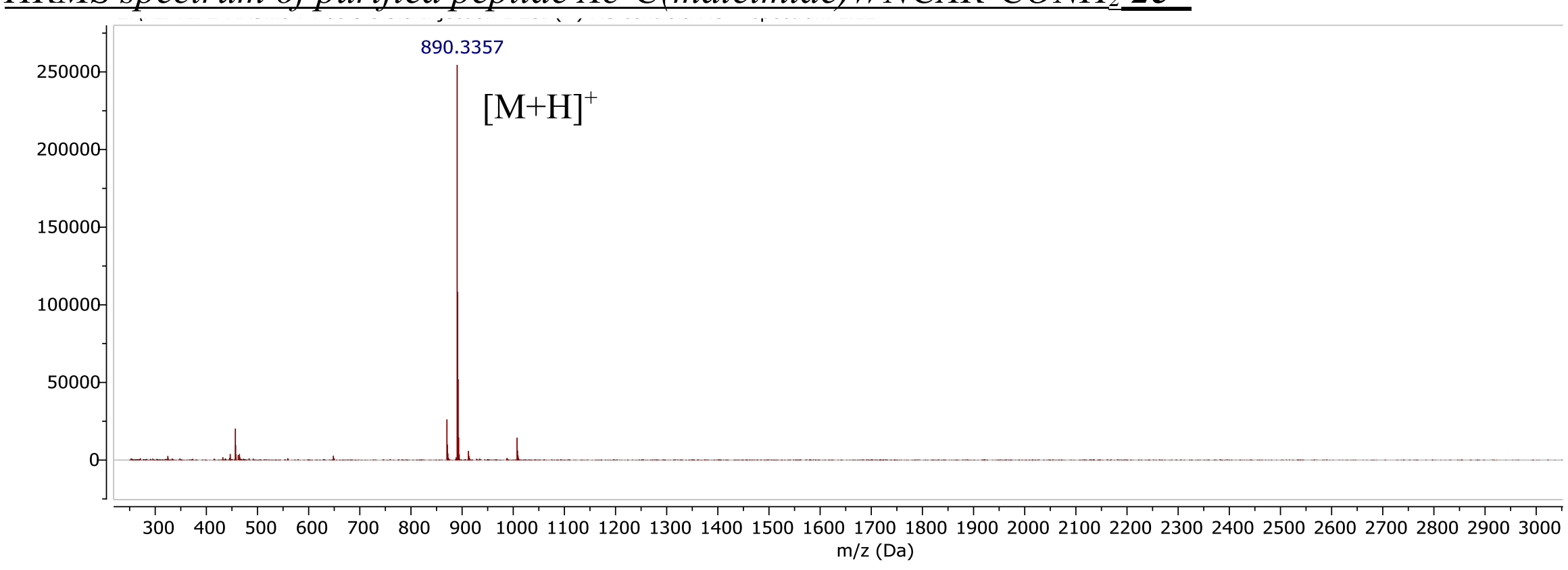

XII.c.4. Orthogonal Modification of Free Cys on Unprotected Peptide **2c''**.

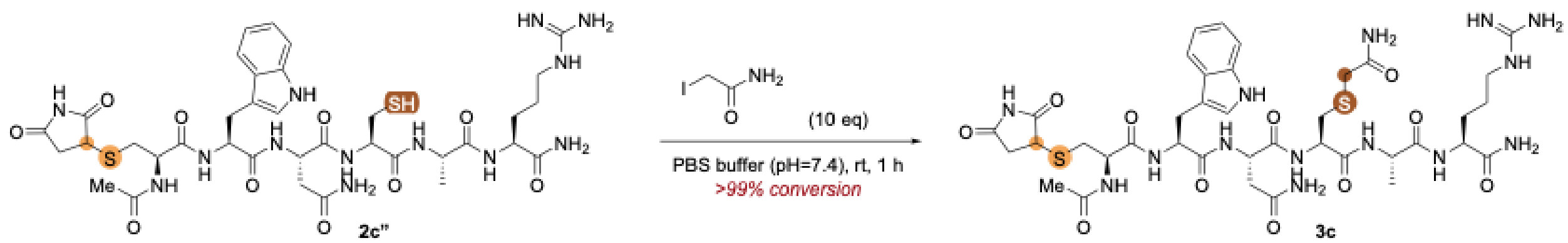

In a 1 dram vial equipped with a magnetic stirrer was added the purified peptide Ac-C(maleimide)WNCAR-$CONH_2$ **2c''** (2 mg, 0.0022 mmol, 1 eq) and it was dissolved in PBS buffer (pH=7.4) (300 µL) while stirring. In a separate 1 dram vial, iodoacetamide (4.1 mg, 0.022 mmol, 10 eq) was taken and dissolved in PBS buffer (200 µL) and the solution was added dropwise to the peptide solution and resulting mixture was allowed to stir 1 h at room temperature. After that, the selective iodoacetamide adduct peptide **3c** (pale white solid, 1.9 mg, >99% conversion) was determined via analytical HPLC using 0.1% formic acid (FA) in both mobile phases A and B. Following analysis, product was subsequently purified using this same method.

Peptide Ac-C(maleimide)WNC(iodoacetamide)AR-$CONH_2$ 3c: **HRMS (ESI)** m/z: $C_{38}H_{55}N_{14}O_{11}S_2$ $[M+H]^+$ Calcd for 947.3611, Found 947.3611. **Analytical HPLC Method** (C18, Water (A)/ACN (B); gradient: 5 to 85% B (0.1% formic acid in ACN) in 20 min, flow rate = 1.0 mL/min, I = 220 nm)**:** retention time of 7.99 min.

*Analytical HPLC trace of the crude reaction mixture converting Ac-C(maleimide)WNCAR-$CONH_2$ **2c''** to Ac-C(maleimide)WNC(iodoacetamide)AR-$CONH_2$ **3c***

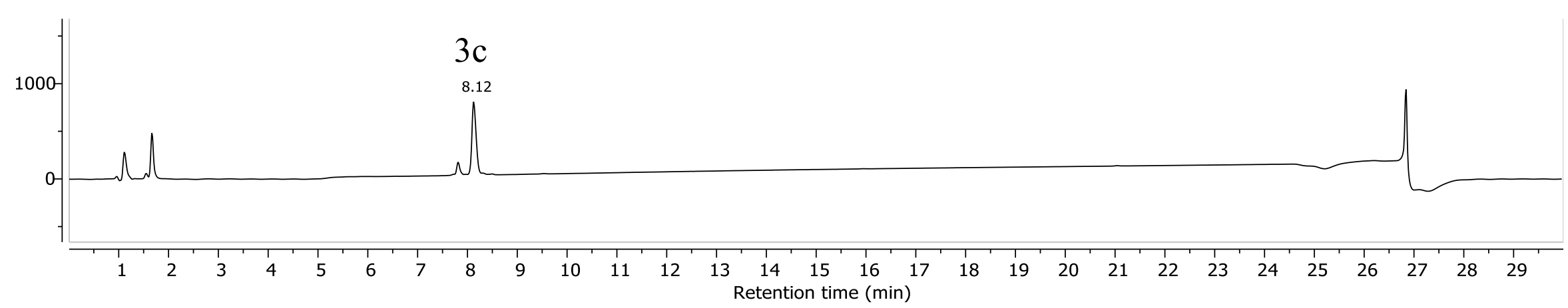

*Analytical HPLC trace of purified peptide Ac-C(maleimide)WNC(iodoacetamide)AR-$CONH_2$ **3c***

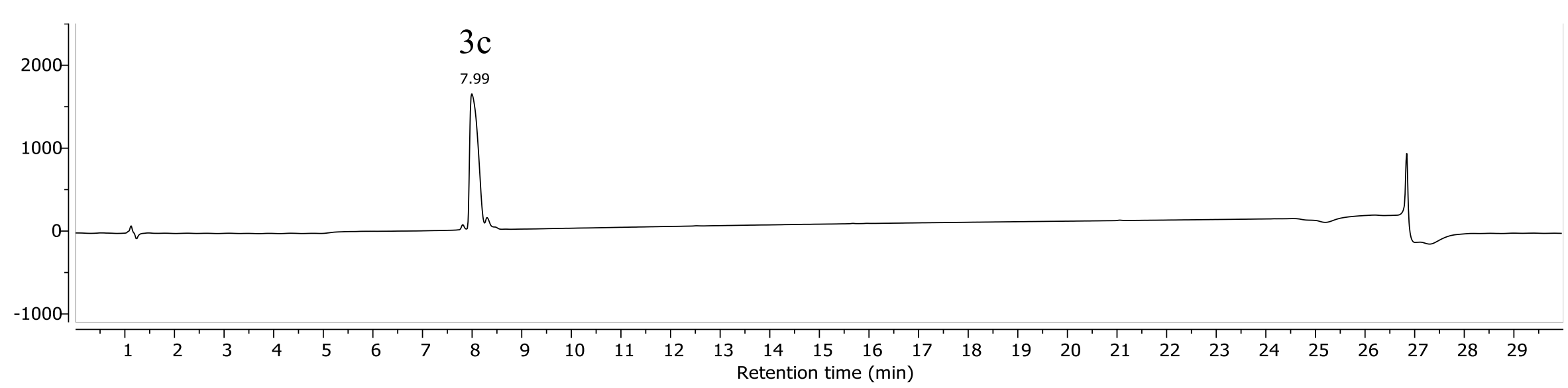

HRMS spectrum of purified peptide Ac-C(maleimide)WNC(iodoacetamide)AR-CONH₂ ***3c***

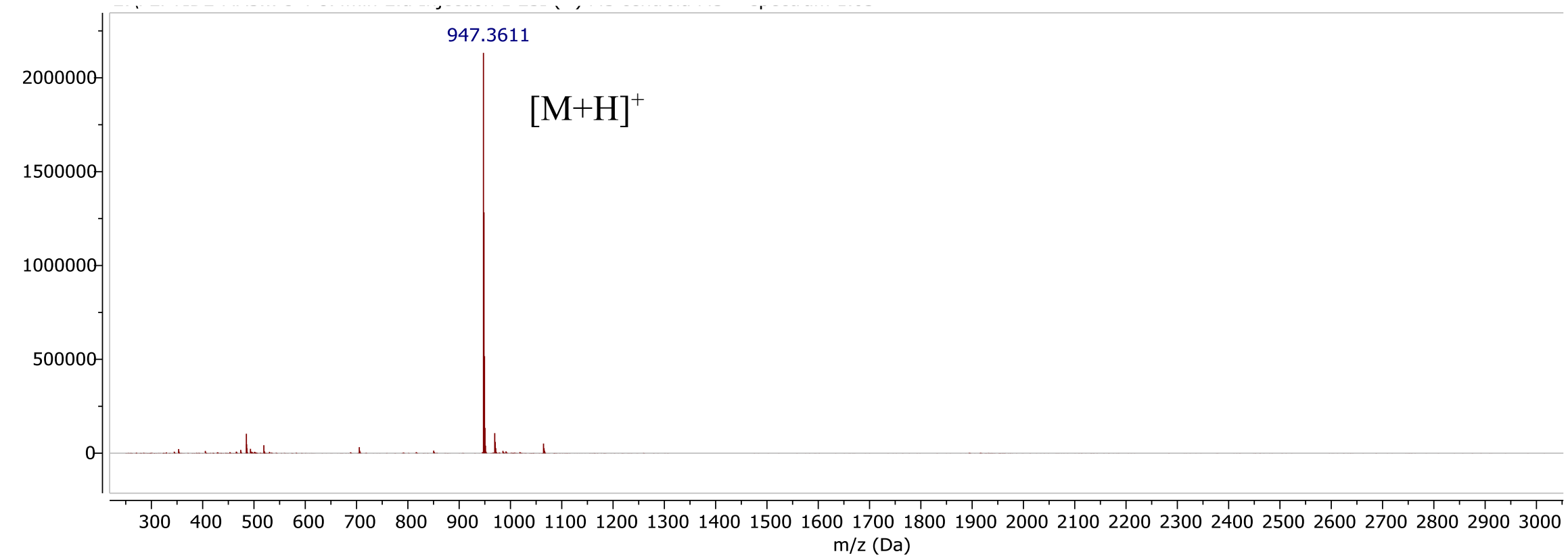

XII.d. Table S4: Screening of Reaction Conditions for Orthogonal Cleavage of Aryl-Containing Disulfide on 1g from Unprotected Peptide Mixture of **1g** and **1h**.

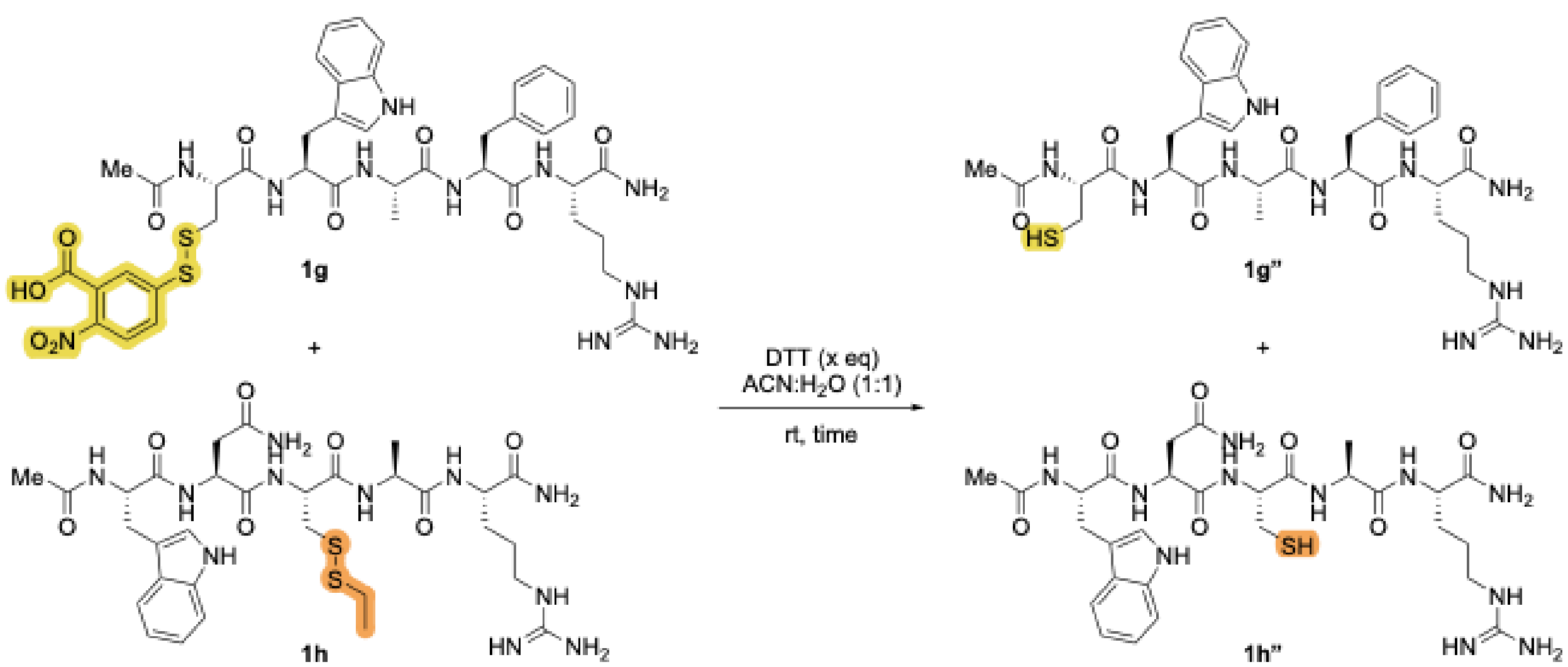

Entry	DTT (x eq)	Time (min)	(% of conversion)			
			**1g**	**1h**	**1g''**	**1h''**
1	5	0	-	87	100	13
2	5	10	-	89	100	11
3	5	20	-	83	100	17
4	5	40	-	72	100	28
5	10	10	-	83	100	16
6	10	20	-	78	100	22
7	10	40	-	65	100	35
9	2	10	32	89	68	11
10	2	20	16	73	84	27

11	2	30	9	55	91	45
12	2	40	8	47	92	53

In a 1 dram vial equipped with a magnetic stirrer were added the purified peptide Ac-C(SAr^2)WAFR-CONH$_2$ **1g** (1 mg, 0.0011 mmol, 1 eq) and Ac-WNC(SEt)AR-CONH$_2$ **1h** (1 mg, 0.0013 mmol, 1 eq) and dissolved in 500 µL of ACN:H$_2$O (1:1). In a separate 1 dram vial, dithiothreitol (DTT) was taken and dissolved in 100 µL of ACN:H$_2$O (1:1) and the solution was added at once to the peptide solution for screening under the conditions listed in **Table S4** at room temperature. Each reaction mixture was quenched with 50 µL 1N HCl solution followed by diluted with 150 µL of (ACN:H$_2$O = 1:1) after speculated time period mentioned in the **Table S4** and analyzed via **Analytical HPLC Method** (C18, Water (A)/ACN (B); gradient: 5 to 85% B (0.1% formic acid in ACN) in 20 min, flow rate = 1.0 mL/min, I = 220 nm) to determine the conversion. Under these conditions **1g**, **1h** along with the desired reduced free Cys containing peptide products **1g''** and **1h''** exhibited retention times of 12.05, 9.92, 10.65 and 8.42 min, respectively.

Peptide Ac-C(SAr^2)WAFR-CONH$_2$ 1g: HRMS (ESI) m/z: $C_{41}H_{50}N_{11}O_{10}S_2$ $[M+H]^+$ Calcd for 920.3184, Found 920.3179. **Analytical HPLC Method** (C18, Water (A)/ACN (B); gradient: 5 to 85% B (0.1% formic acid in ACN) in 20 min, flow rate = 1.0 mL/min, I = 220 nm)**:** retention time of 12.05 min.
Peptide Ac-WNC(SEt)AR-CONH$_2$ 1h: HRMS (ESI) m/z: $C_{31}H_{48}N_{11}O_7S_2$ $[M+H]^+$ Calcd for 750.3180, Found 750.3156. **Analytical HPLC Method** (C18, Water (A)/ACN (B); gradient: 5 to 85% B (0.1% formic acid in ACN) in 20 min, flow rate = 1.0 mL/min, I = 220 nm)**:** retention time of 9.92 min.
Peptide Ac-CWAFR-CONH$_2$ 1g'': HRMS (ESI) m/z: $C_{34}H_{47}N_{10}O_6S$ $[M+H]^+$ Calcd for 723.3395, Found 723.3400. **Analytical HPLC Method** (C18, Water (A)/ACN (B); gradient: 5 to 85% B (0.1% formic acid in ACN) in 20 min, flow rate = 1.0 mL/min, I = 220 nm)**:** retention time of 10.65 min.
Peptide Ac-WNCAR-CONH$_2$ 1h'': HRMS (ESI) m/z: $C_{29}H_{44}N_{11}O_7S$ $[M+H]^+$ Calcd for 690.3140, Found 690.3176. **Analytical HPLC Method** (C18, Water (A)/ACN (B); gradient: 5 to 85% B (0.1% formic acid in ACN) in 20 min, flow rate = 1.0 mL/min, I = 220 nm)**:** retention time of 8.42 min.

*Analytical HPLC trace of purified peptide mixture Ac-C(SAr^2)WAFR-CONH$_2$ **1g** and Ac-WNC(SEt)AR-CONH$_2$ **1h***

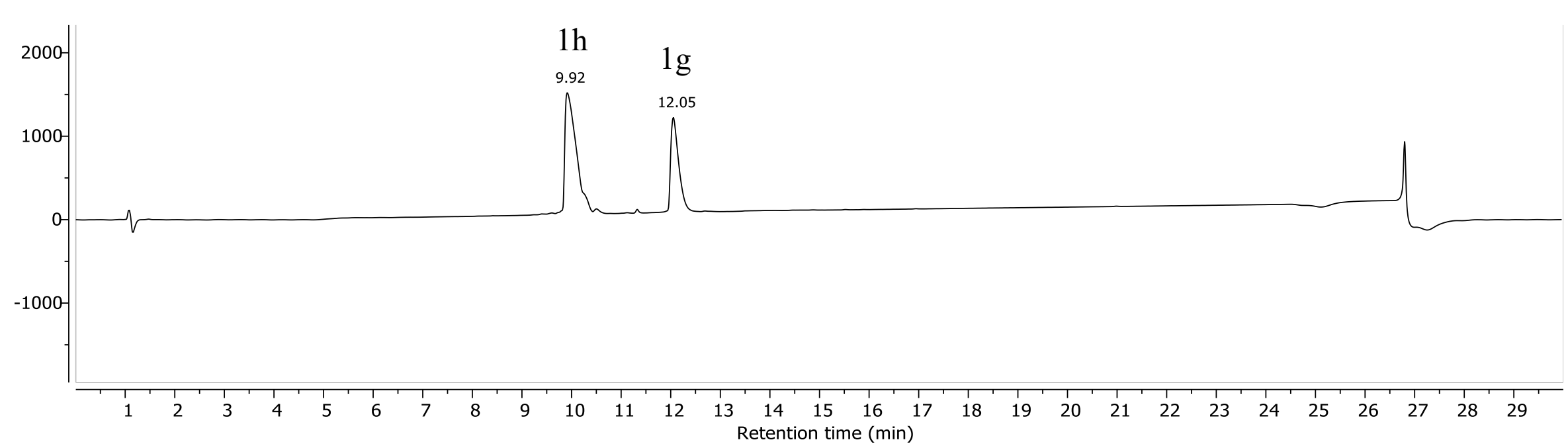

Analytical HPLC trace of the crude reaction mixture

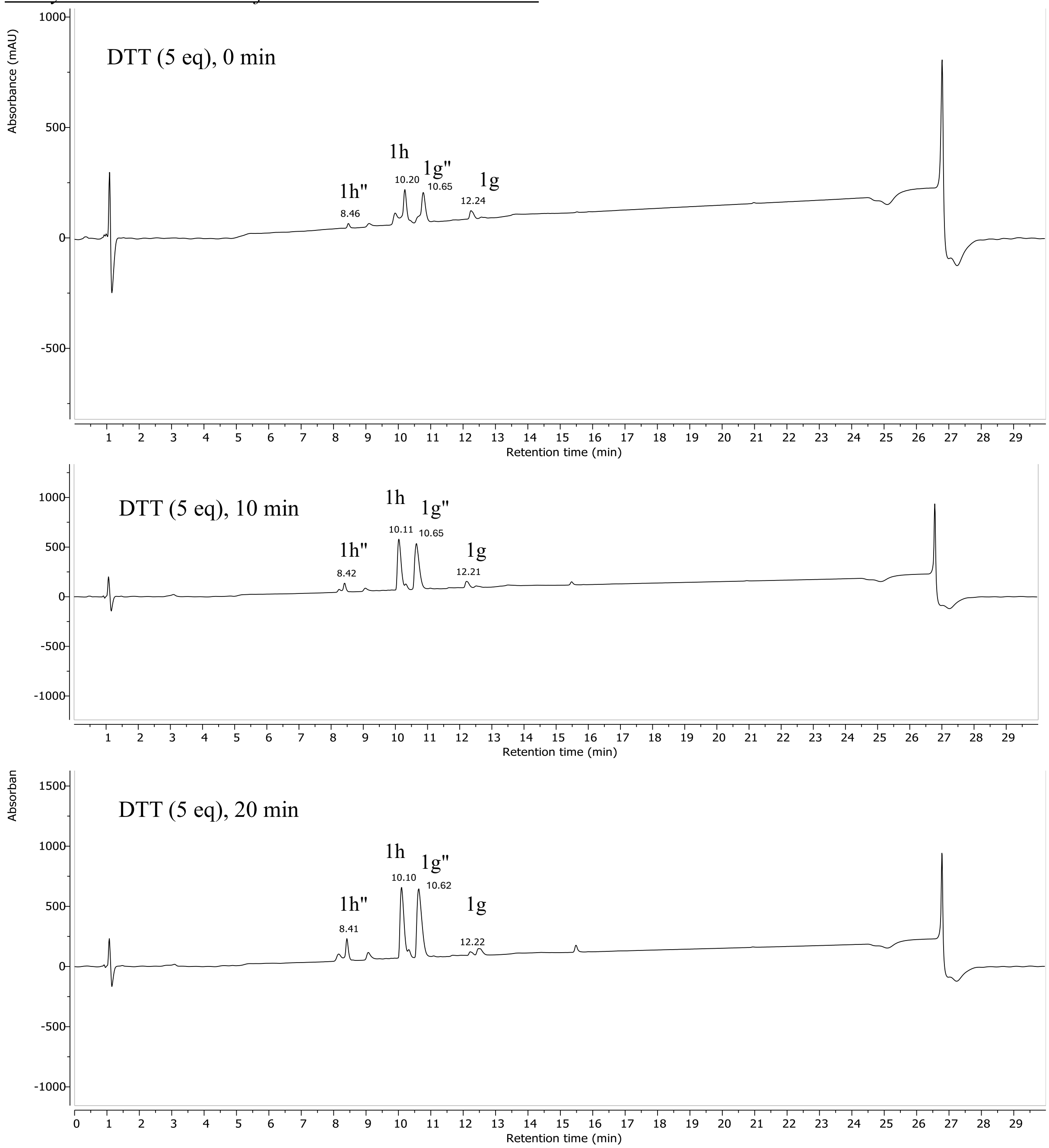

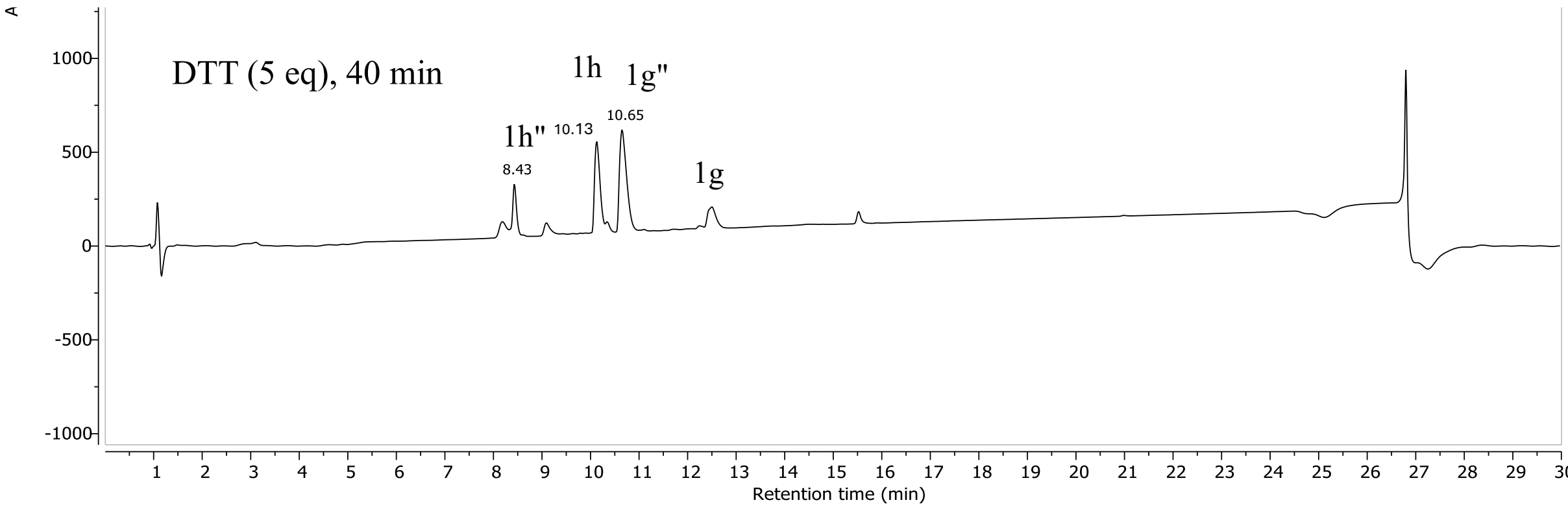
DTT (5 eq), 40 min
1h"
8.43
1h
10.13
1g"
10.65
1g
Retention time (min)

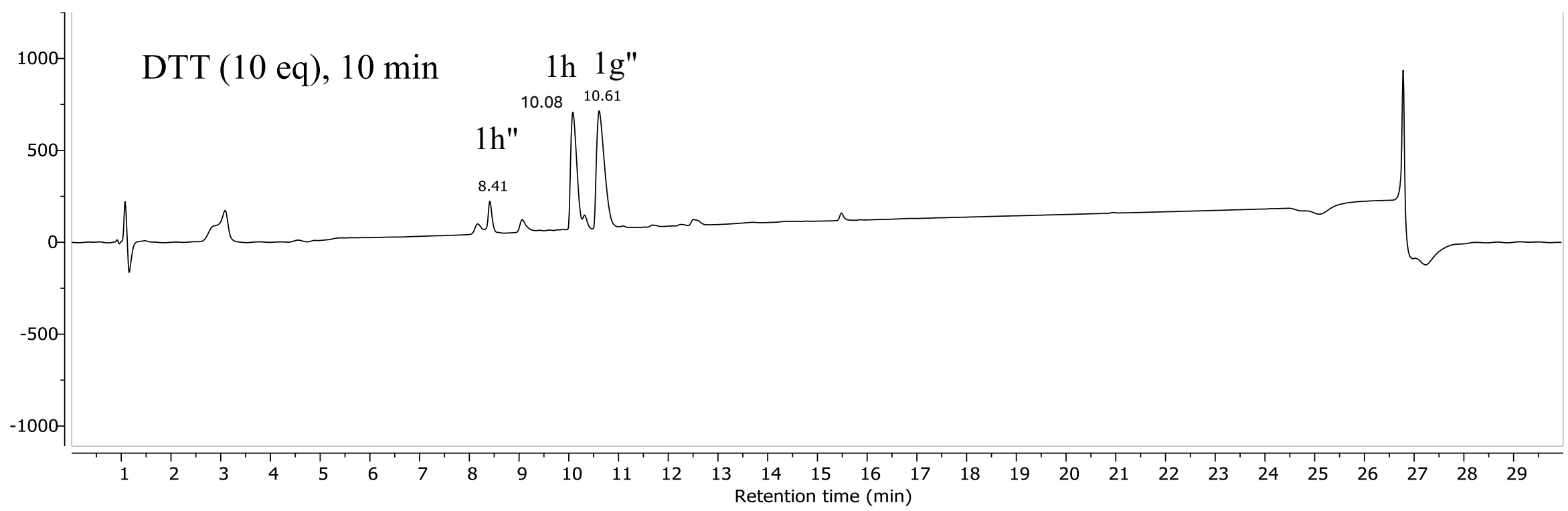
DTT (10 eq), 10 min
1h"
8.41
1h
10.08
1g"
10.61
Retention time (min)

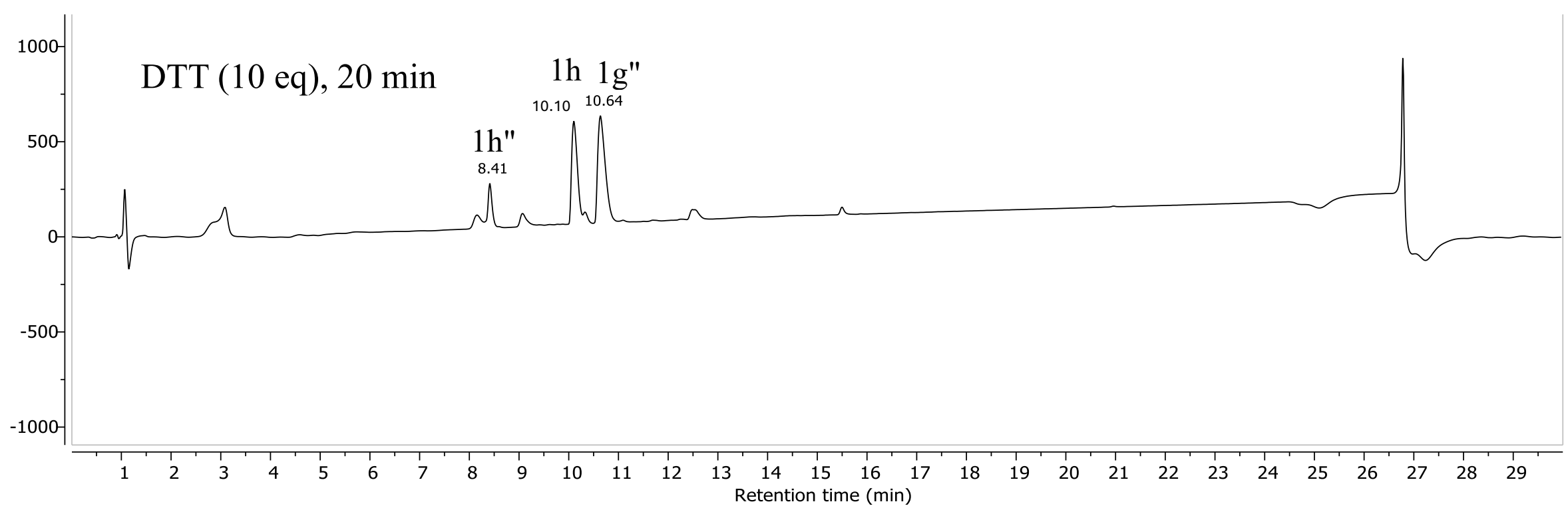
DTT (10 eq), 20 min
1h"
8.41
1h
10.10
1g"
10.64
Retention time (min)

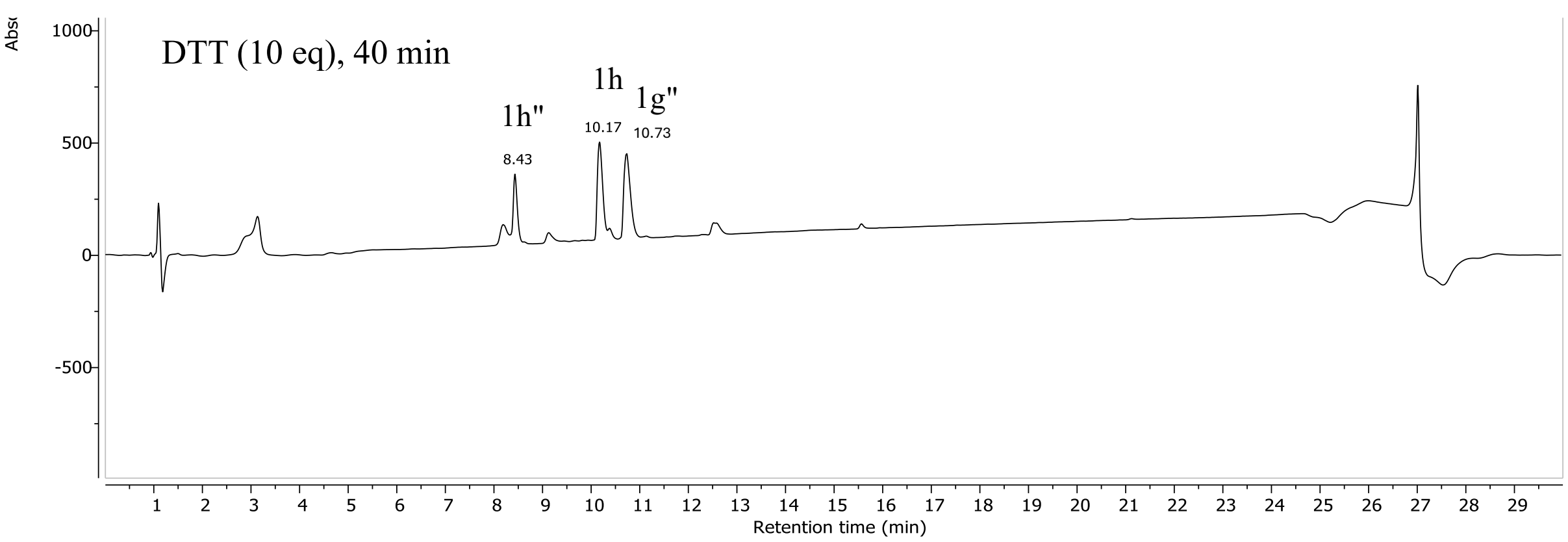
DTT (10 eq), 40 min
1h"
8.43
1h
10.17
1g"
10.73
Retention time (min)

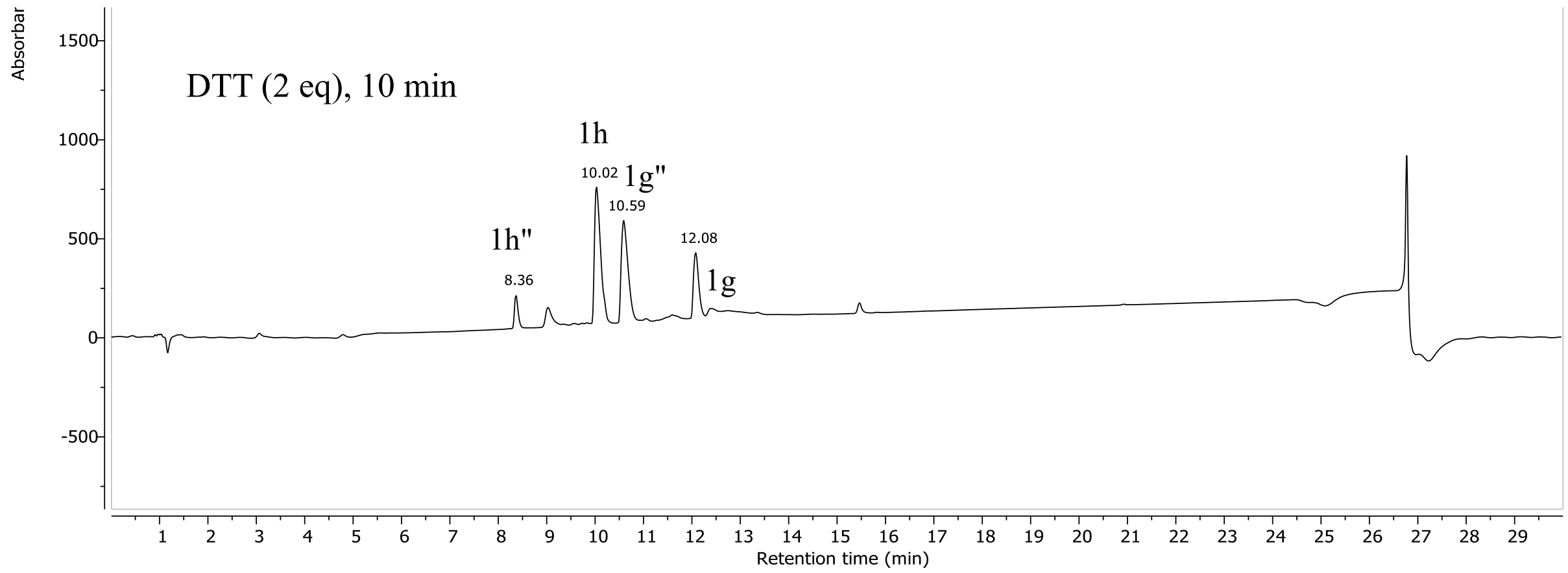
DTT (2 eq), 10 min
1h
10.02
1g''
10.59
1h''
8.36
12.08
1g
Absorbar
Retention time (min)

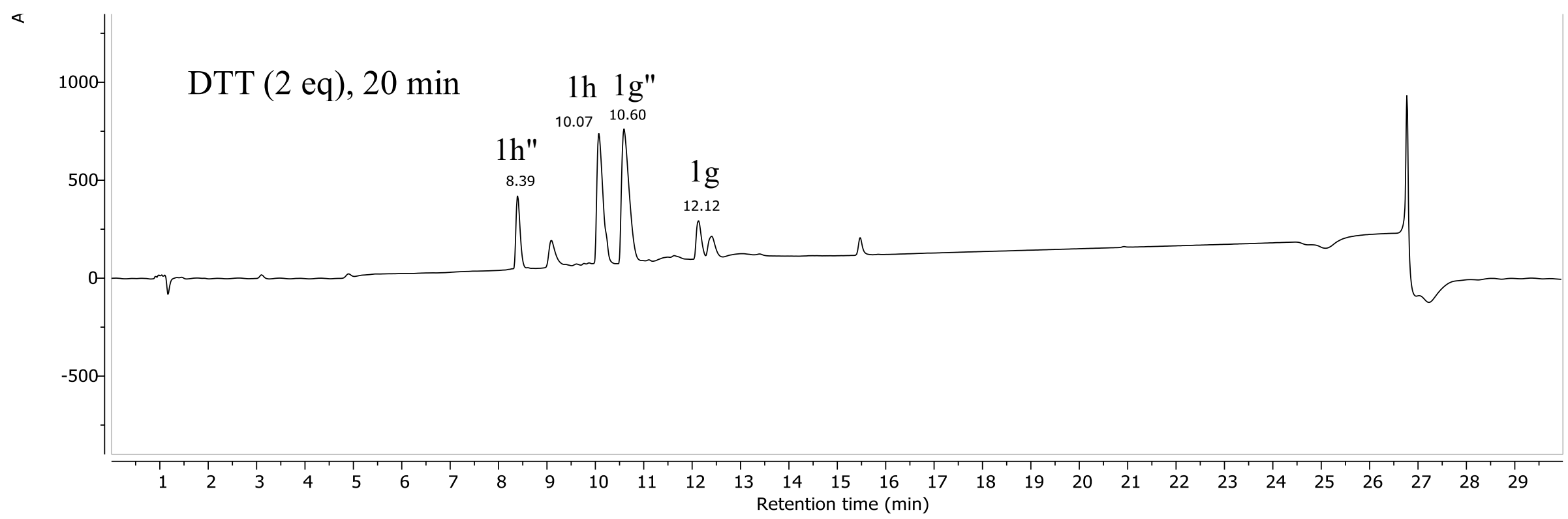
DTT (2 eq), 20 min
1h
10.07
1g''
10.60
1h''
8.39
1g
12.12
A
Retention time (min)

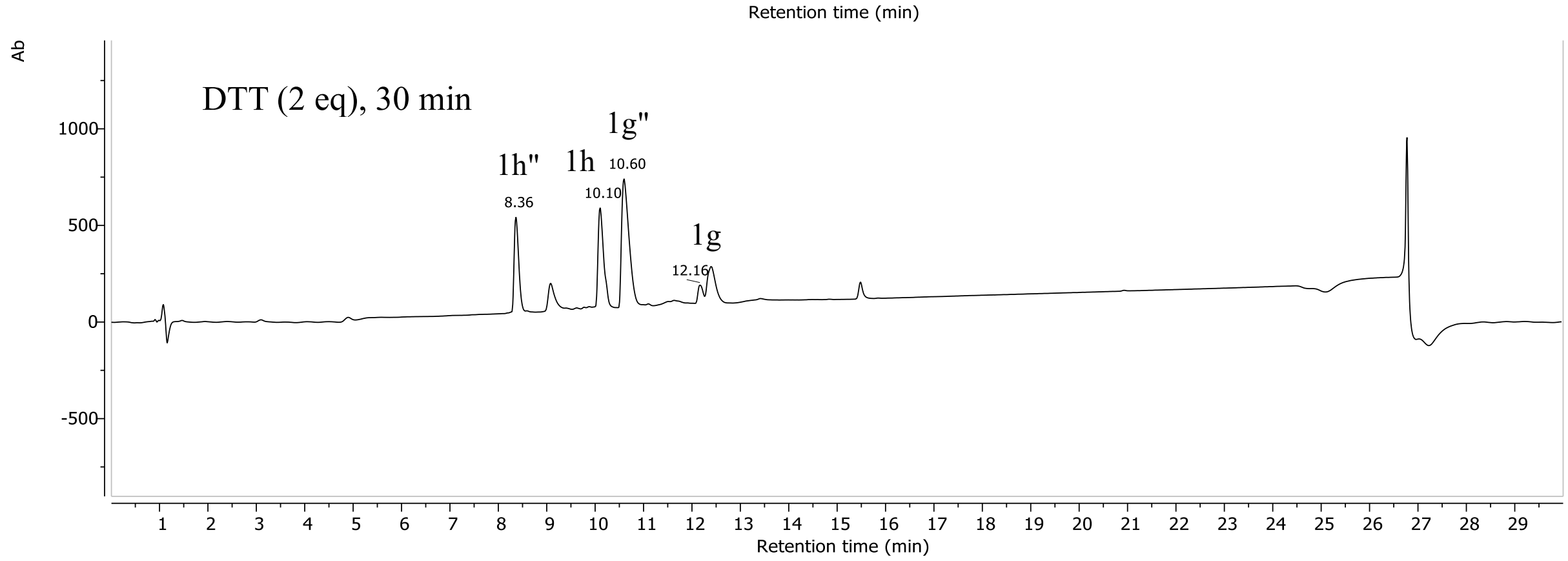
DTT (2 eq), 30 min
1g''
10.60
1h''
8.36
1h
10.10
1g
12.16
Ab
Retention time (min)

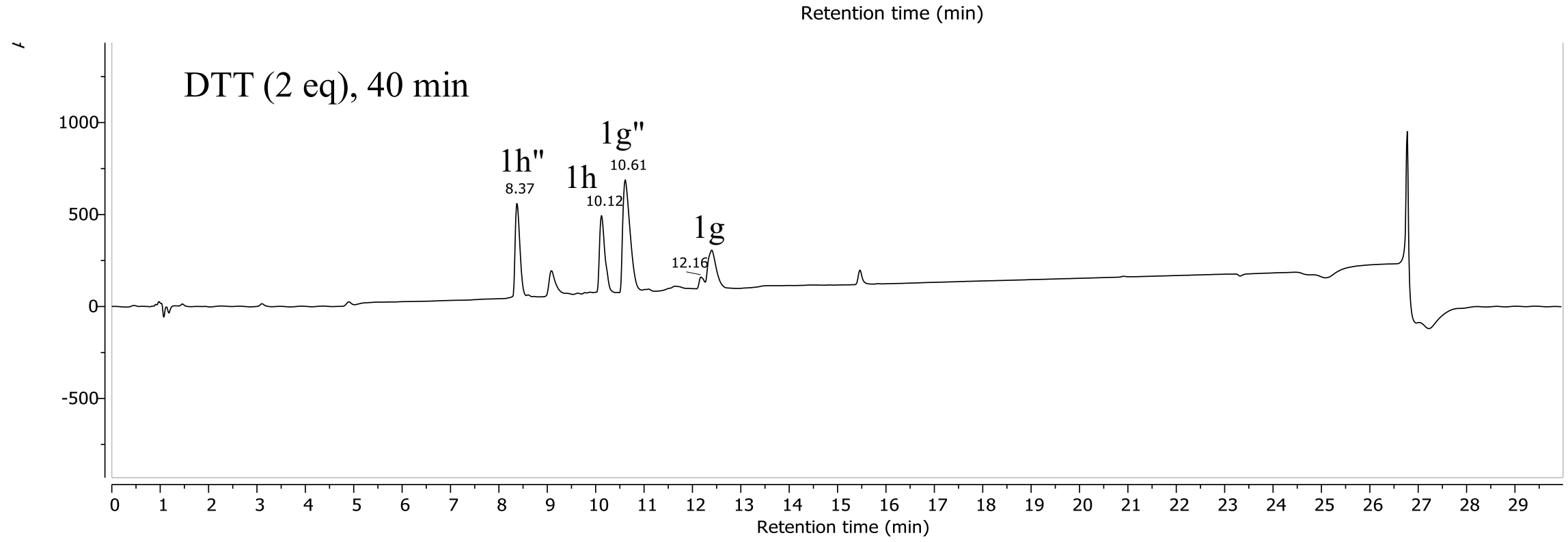
DTT (2 eq), 40 min
1g''
10.61
1h''
8.37
1h
10.12
1g
12.16
Retention time (min)

HRMS spectrum of purified peptide Ac-C(SAr[2])WAFR-CONH$_2$ ***1g***

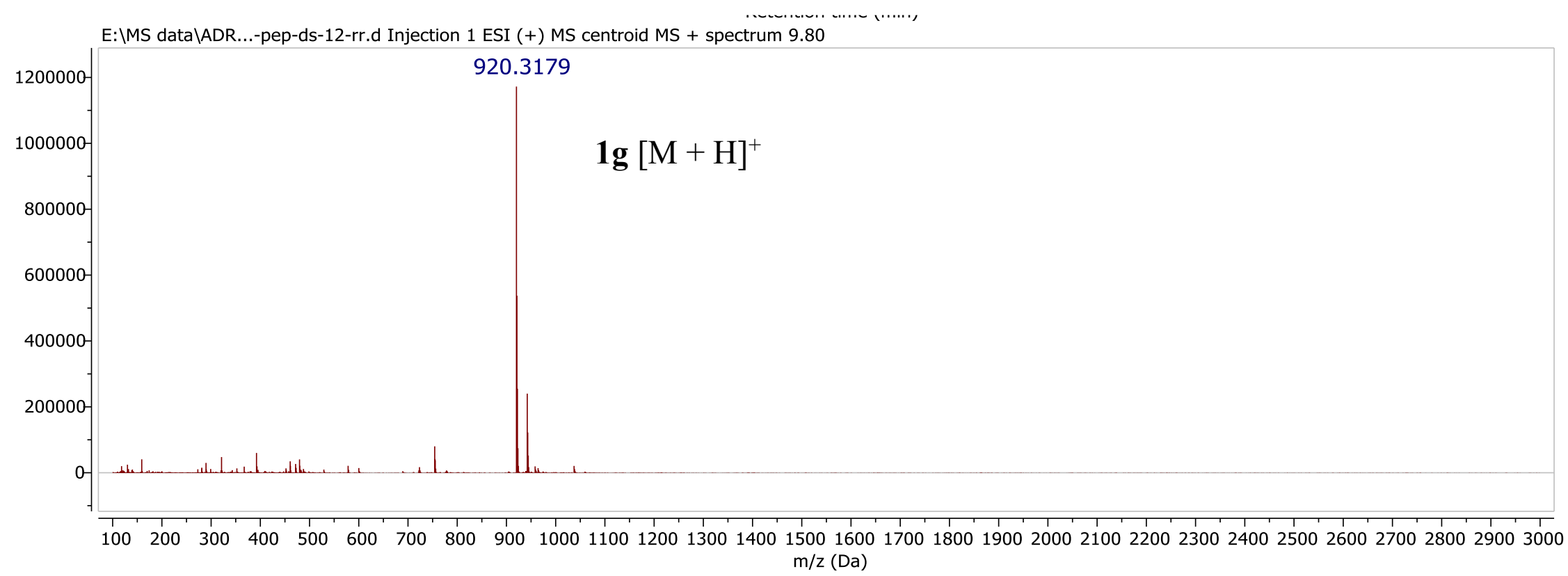

HRMS spectrum of purified peptide Ac-WNC(SEt)AR-CONH$_2$ ***1h***

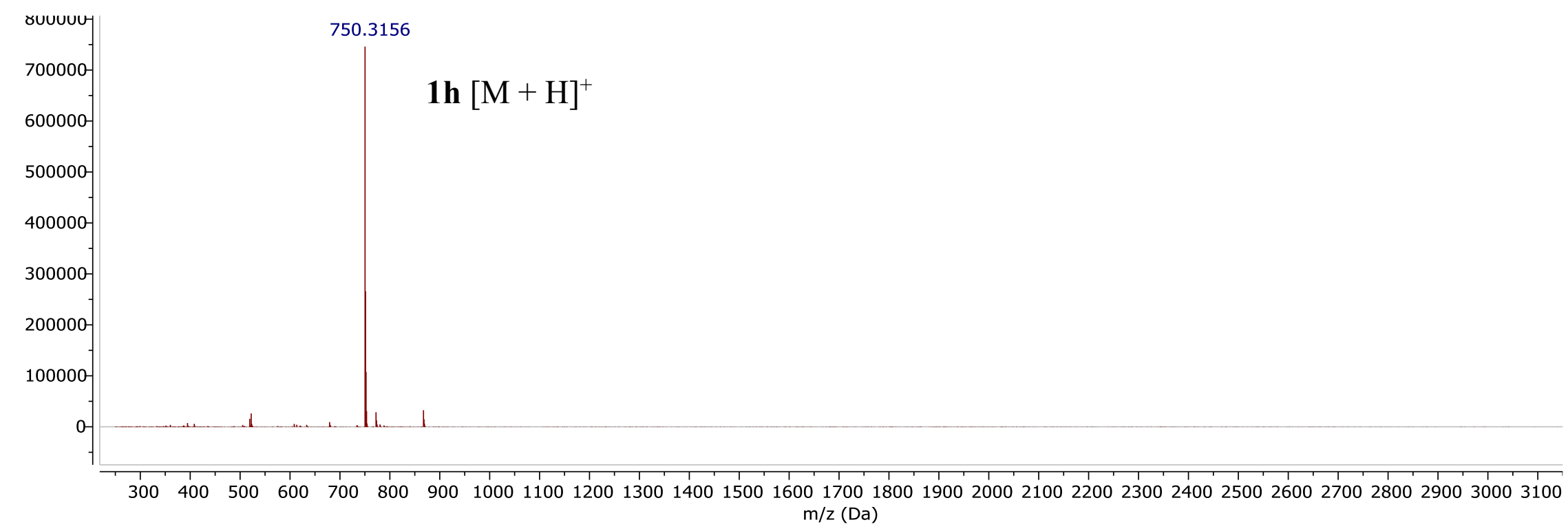

HRMS spectrum of purified peptide Ac-CWAFR-CONH$_2$ ***1g''***

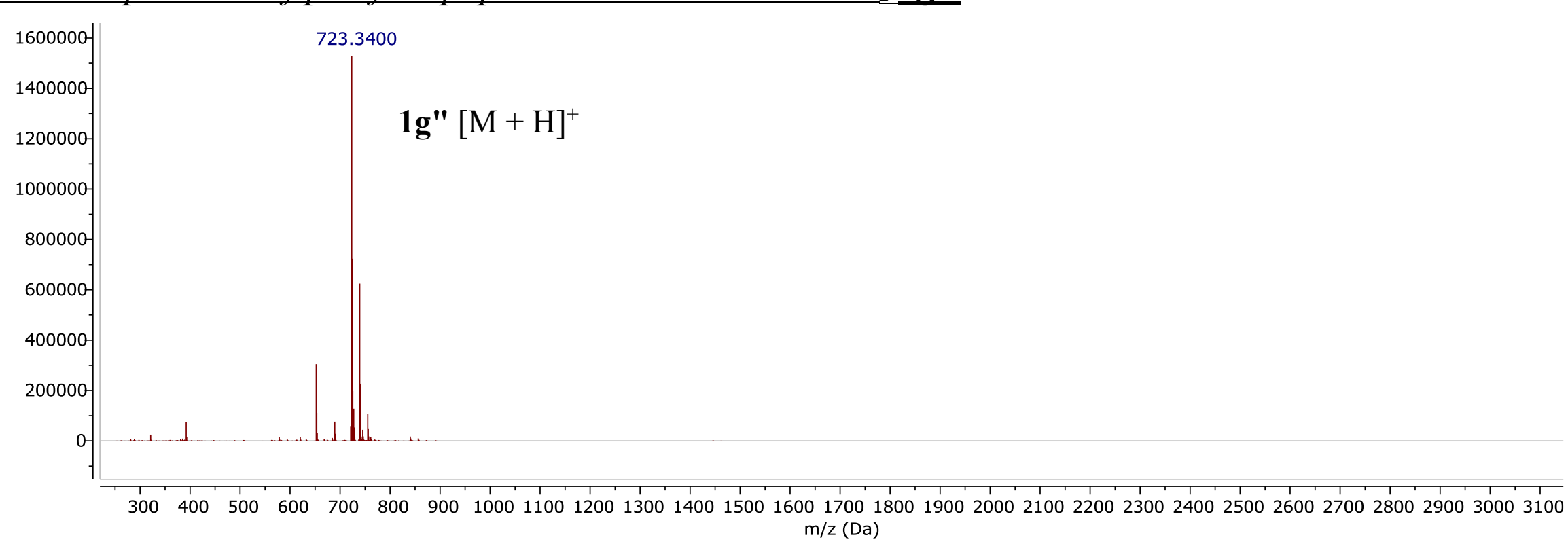

HRMS spectrum of purified peptide Ac-WNCAR-CONH$_2$ ***1h"***

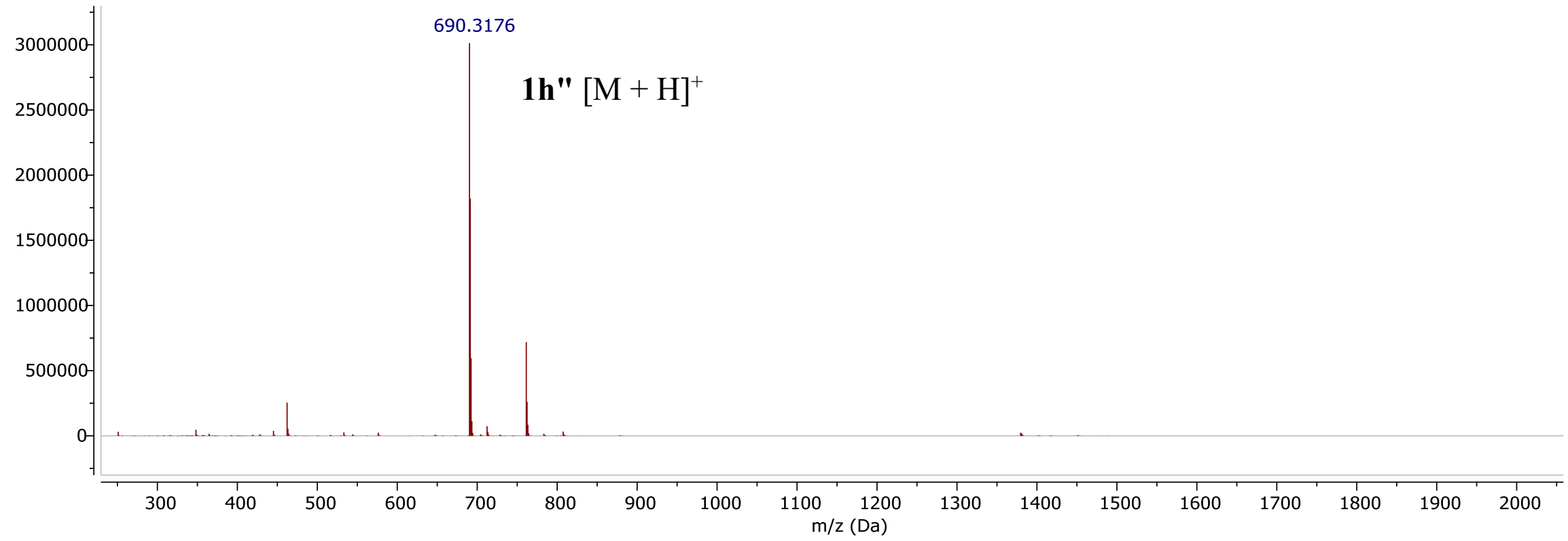

XII.d. Supplementary Figure 5. Orthogonal Cleavage and Modification of Aryl and Ethyl Containing Disulfides in Fully Unprotected Peptide mixture of **1g** and **1h**.

XII.d.1. Orthogonal Cleavage of Aryl Containing Disulfide on 1g from the Mixture of Peptides **1g** and **1h**.

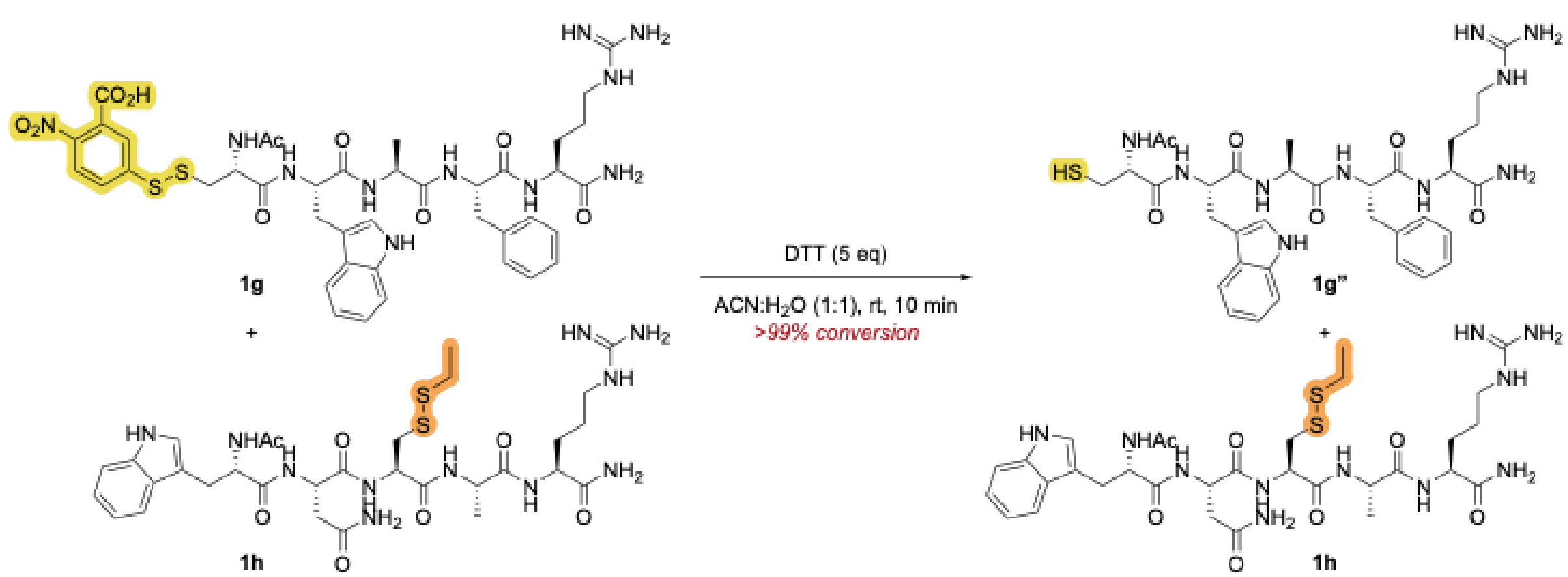

In a 1 dram vial equipped with a magnetic stirrer were added the purified peptide mixture Ac-C(SAr[2])WAFR-CONH$_2$ **1g** (1 mg, 0.0011 mmol, 1 eq) and Ac-WNC(SEt)AR-CONH$_2$ **1h** (1 mg, 0.0013 mmol, 1 eq) and dissolved in 300 µL of ACN:H$_2$O (1:1). In a separate 1 dram vial, dithiothreitol (DTT, 0.84 mg, 0.0055 mmol, 5 eq) was taken and dissolved in 100 µL of ACN:H$_2$O (1:1) and the solution was added at once to the peptide solution at room temperature and stirred the solution for 10 min. After that, the selective reduced thiol product Ac-CWAFR-CONH$_2$ **1g"** (pale white solid, 1.7 mg, >99% conversion) was determined via analytical HPLC using 0.1% formic acid (FA) in both mobile phases A and B. Following analysis, products were subsequently purified using this same method.

Peptide Ac-CWAFR-CONH$_2$ 1g": **HRMS (ESI)** m/z: $C_{34}H_{47}N_{10}O_6S$ [M + H]$^+$ Calcd for 723.3395, Found 723.3434. **Peptide Ac-CWAFR-CONH$_2$ 1h**: **HRMS (ESI)** m/z: $C_{31}H_{48}N_{11}O_7S_2$ [M+H]$^+$ Calcd for 750.3174, Found 750.3156. **Analytical HPLC Method** (C18, Water (A)/ACN (B); gradient: 5 to 85% B (0.1% formic acid in ACN) in 20 min, flow rate = 1.0 mL/min, I = 220 nm)**:** retention time of **1g"** and **1h** are 10.71 and 10.15 min respectively.

*Analytical HPLC trace of the crude reaction mixture containing Ac-CWAFR-CONH$_2$ **1g''** and Ac-WNC(SEt)AR-CONH$_2$ **1h***

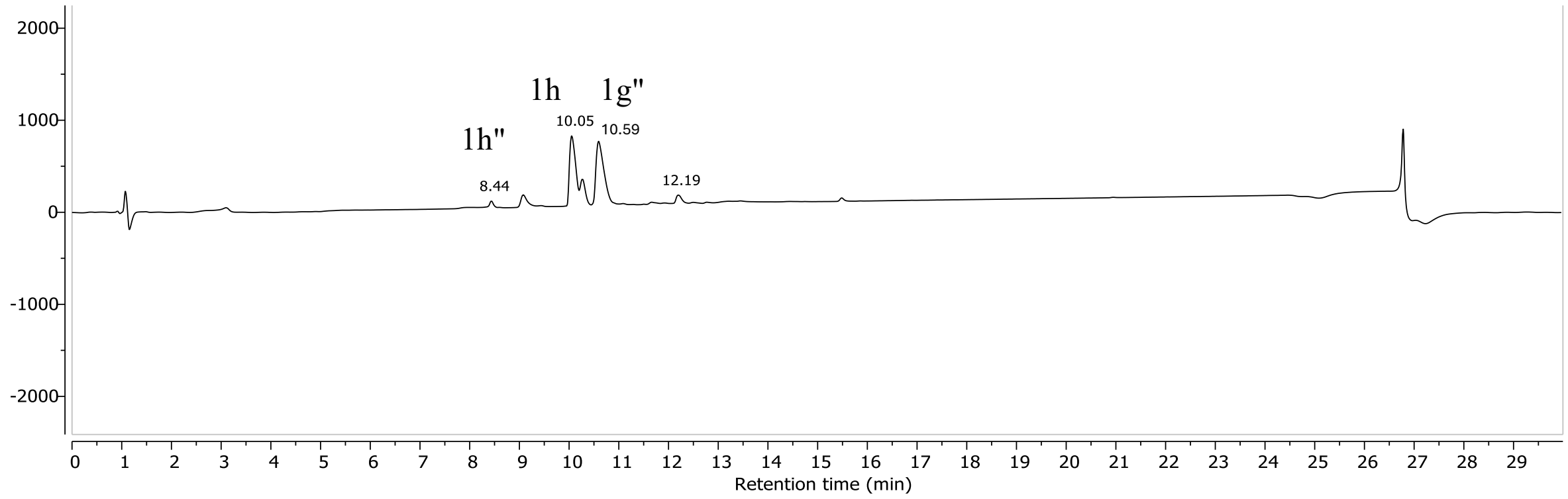

*Analytical HPLC trace of purified peptide mixture of Ac-CWAFR-CONH$_2$ **1g''** and Ac-WNC(SEt)AR-CONH$_2$ **1h***

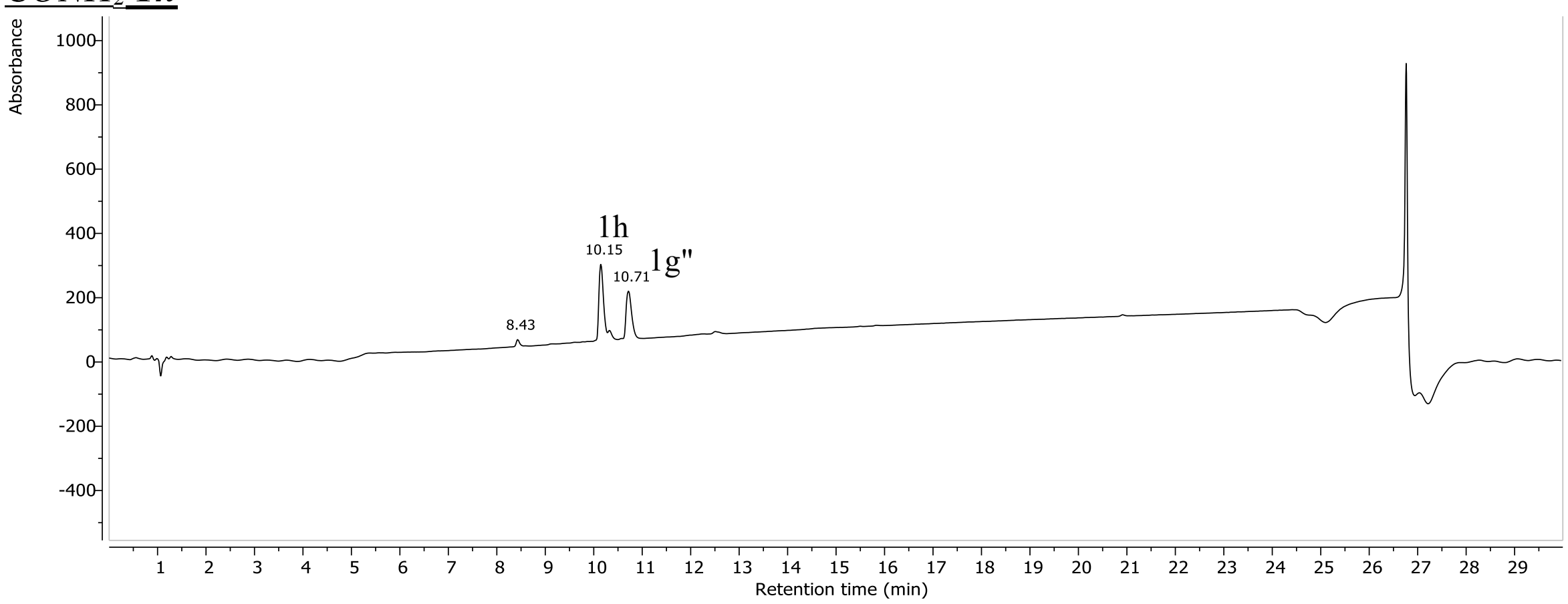

*HRMS spectrum of purified peptide mixture of Ac-CWAFR-CONH$_2$ **1g''** and Ac-WNC(SEt)AR-CONH$_2$ **1h***

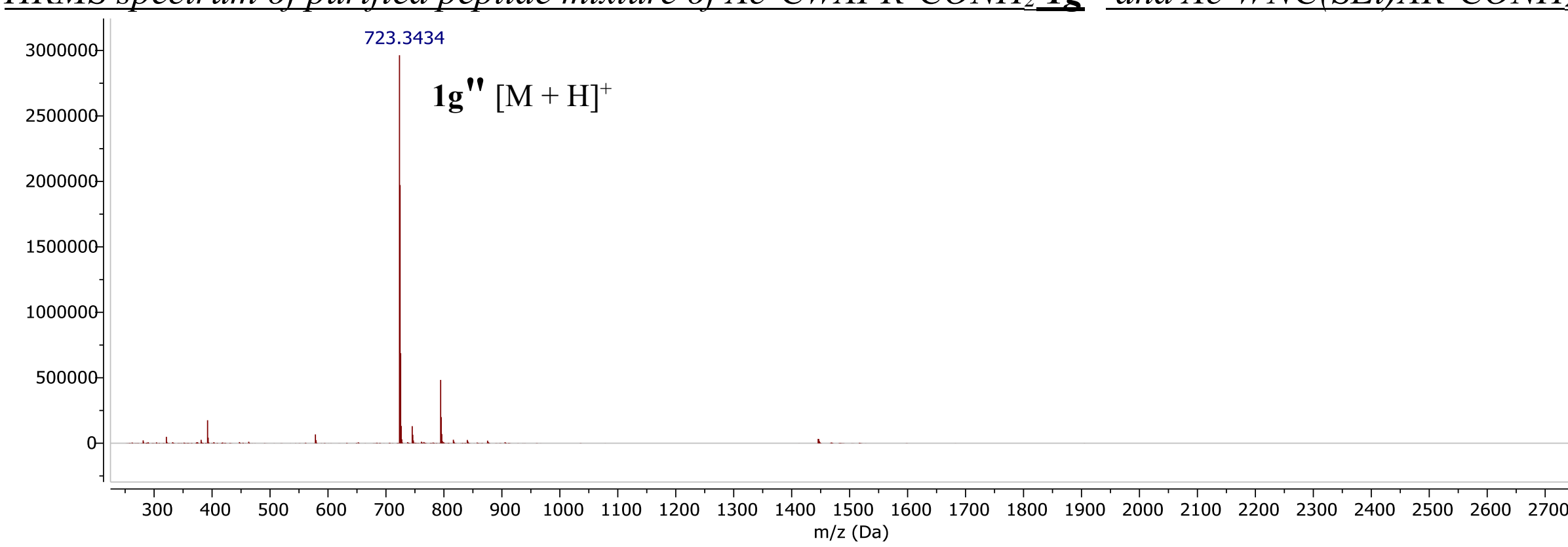

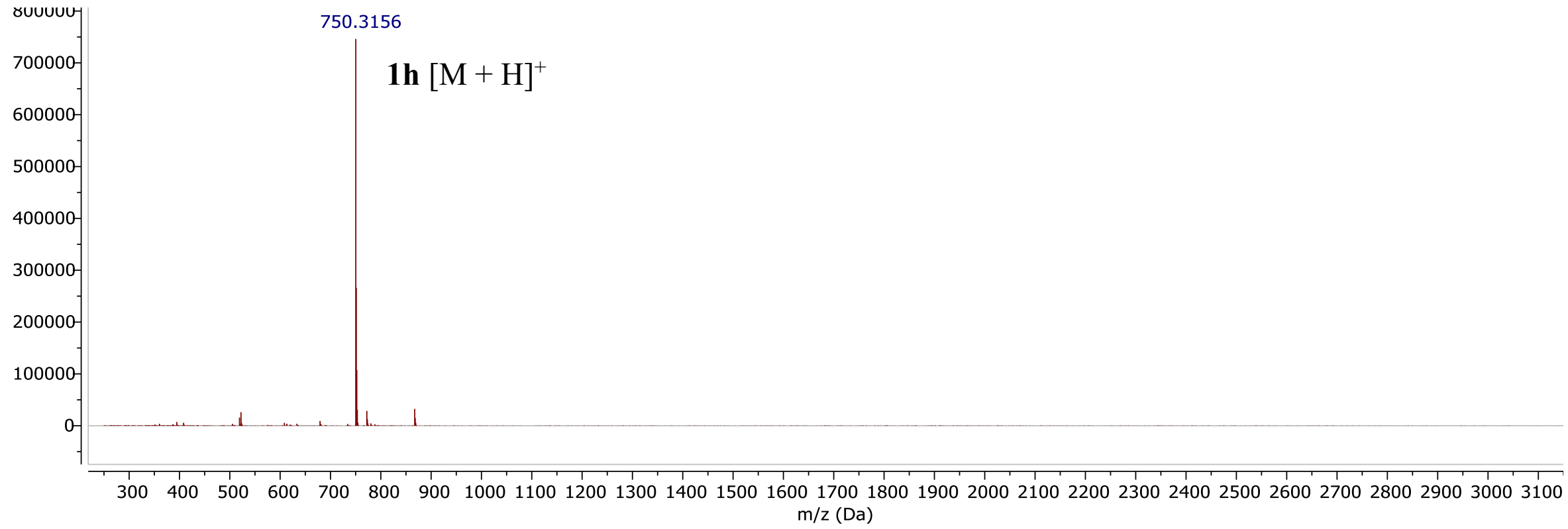

XII.d.2. Orthogonal Modification of Free Cys on **1g''** from the Mixture **1g''** and **1h**.

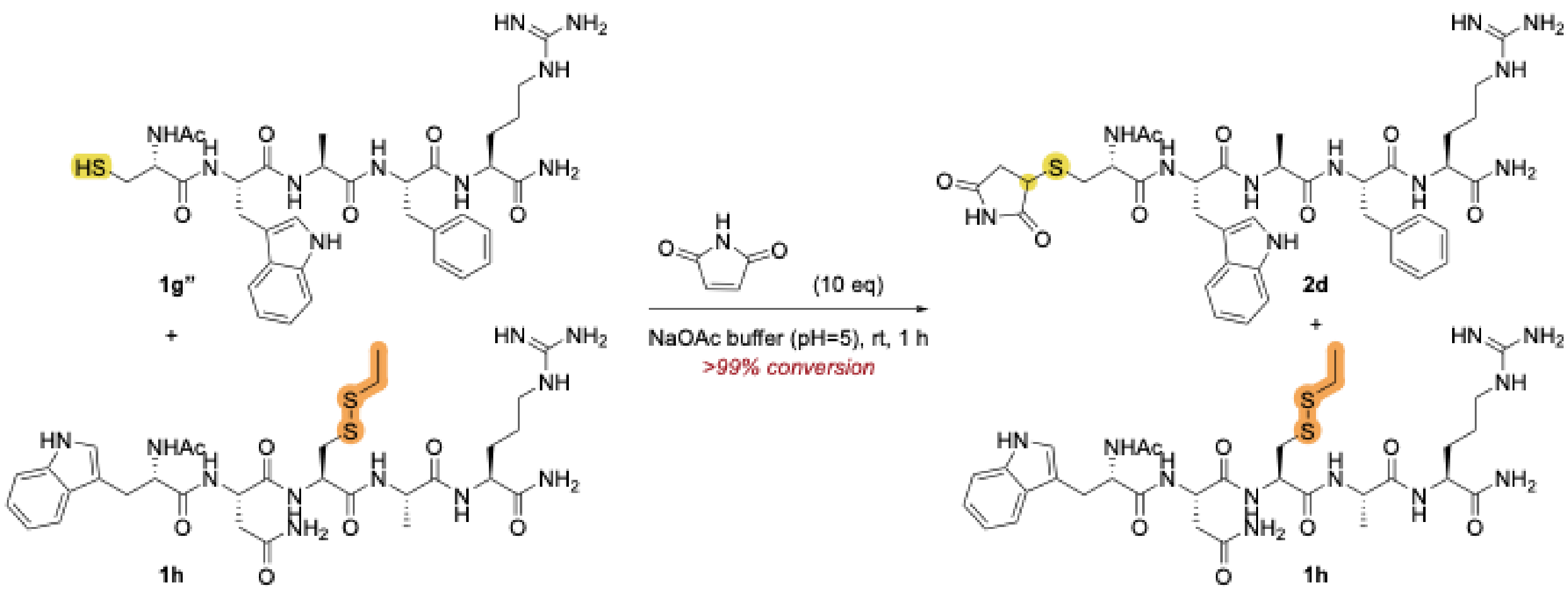

In a 1 dram vial equipped with a magnetic stirrer were added the purified peptide mixture Ac-CWAFR-$CONH_2$ **1g''** and Ac-WNC(SEt)AR-$CONH_2$ **1h** were dissolved in 300 µL of sodium acetate buffer (NaOAc buffer pH = 5) while stirring. In a separate 1 dram vial, maleimide (1.2 mg, 0.012 mmol, 10 eq) was taken and dissolved in NaOAc buffer (100 µL) and the solution was added dropwise to the peptide solution and resulting mixture was allowed to stir 1 h at room temperature. After that, the selective maleimide adduct peptide **2d** (pale white solid, 1.8 mg, >99% conversion) was determined via analytical HPLC using 0.1% formic acid (FA) in both mobile phases A and B. Following analysis, products were subsequently purified using this same method.

Peptide Ac-C(maleimide)WAFR-CONH₂ 2d: **HRMS (ESI)** m/z: $C_{38}H_{50}N_{11}O_8S$ $[M+H]^+$ Calcd for 820.3559, Found 820.3562. **Peptide Ac-WNC(SEt)AR-CONH₂ 1h**: **HRMS (ESI)** m/z: $C_{31}H_{48}N_{11}O_7S_2$ $[M+H]^+$ Calcd for 750.3174, Found 750.3156.

Analytical HPLC Method (C18, Water (A)/ACN (B); gradient: 5 to 85% B (0.1% formic acid in ACN) in 20 min, flow rate = 1.0 mL/min, I = 220 nm)**:** retention time of **2d** and **1h** are 10.21 10.07 min respectively.

*Analytical HPLC trace of the crude reaction mixture containing Ac-C(maleimide)WAFR-CONH$_2$ **2d** and Ac-WNC(SEt)AR-CONH$_2$ **1h***

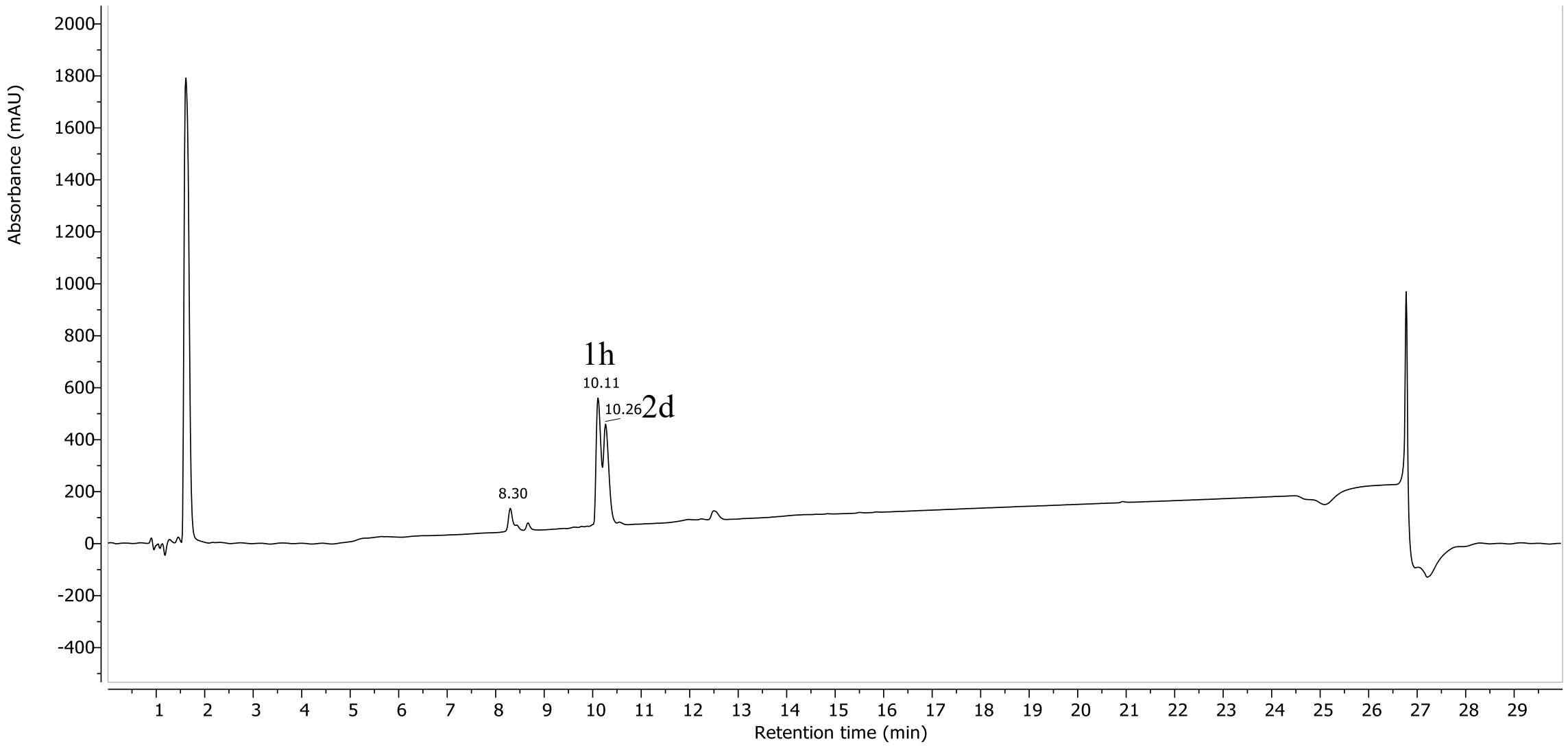

*Analytical HPLC trace of purified peptide mixture of Ac-C(maleimide)WAFR-CONH$_2$ **2d** and Ac-WNC(SEt)AR-CONH$_2$ **1h***

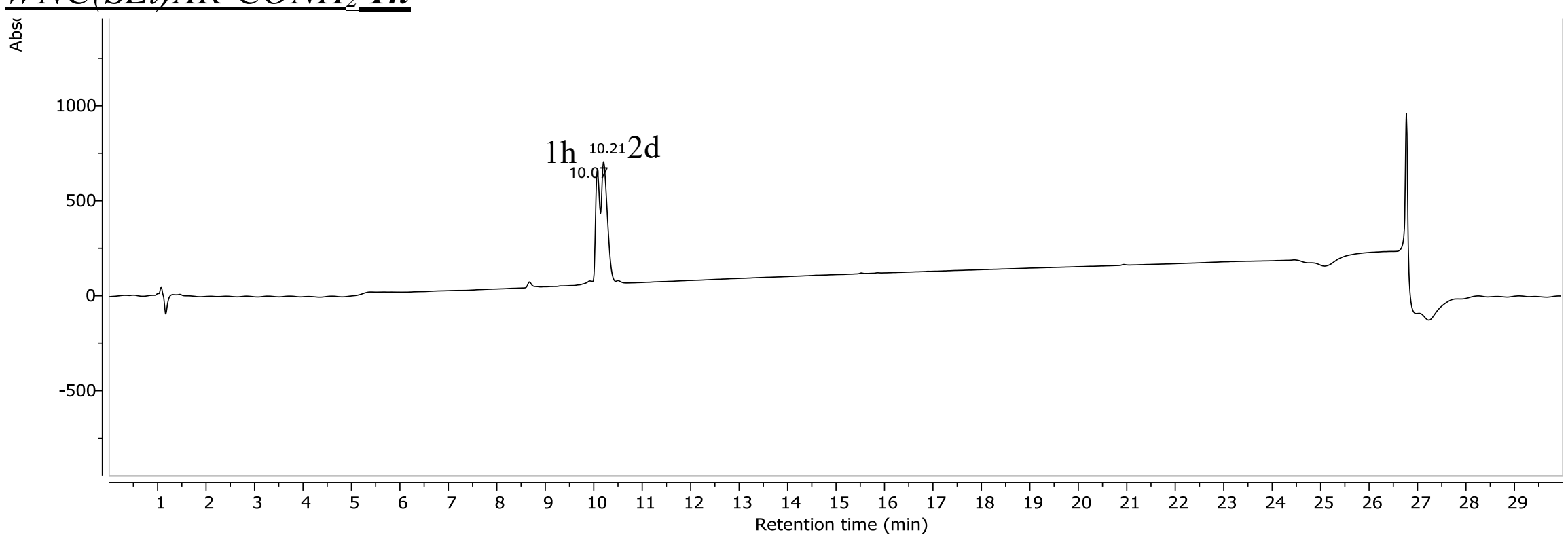

*HRMS spectrum of purified peptide mixture of Ac-C(maleimide)WAFR-CONH$_2$ **2d** and Ac-WNC(SEt)AR-CONH$_2$ **1h***

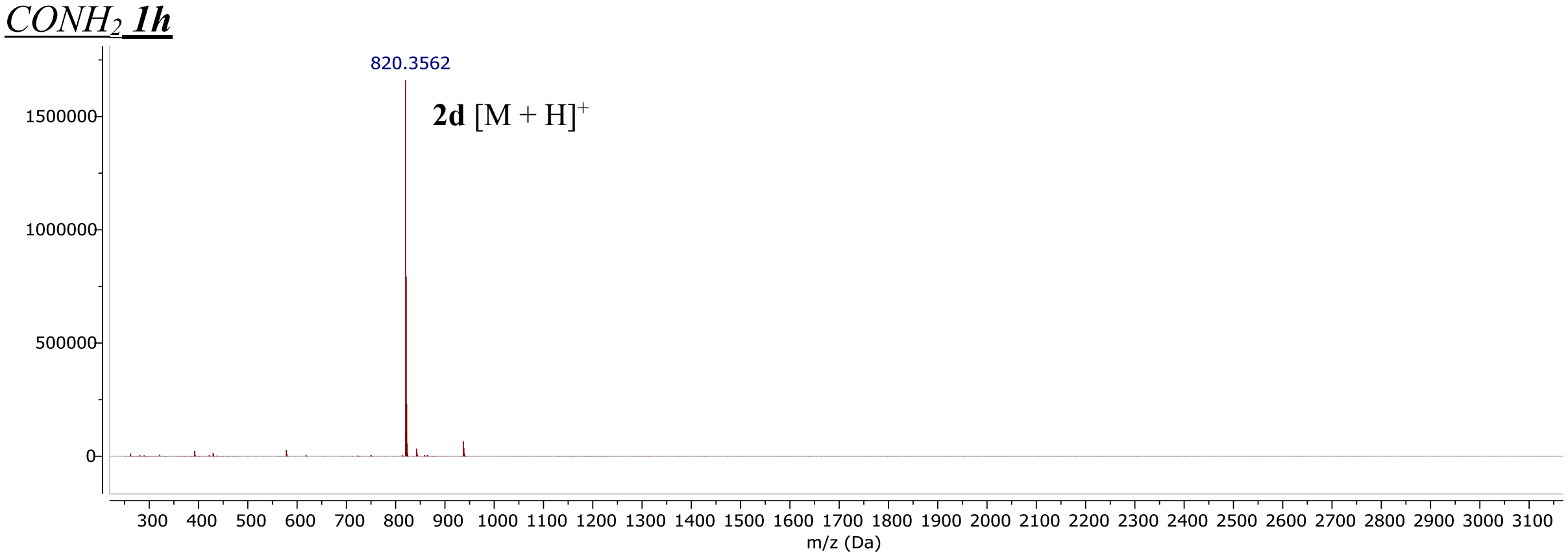

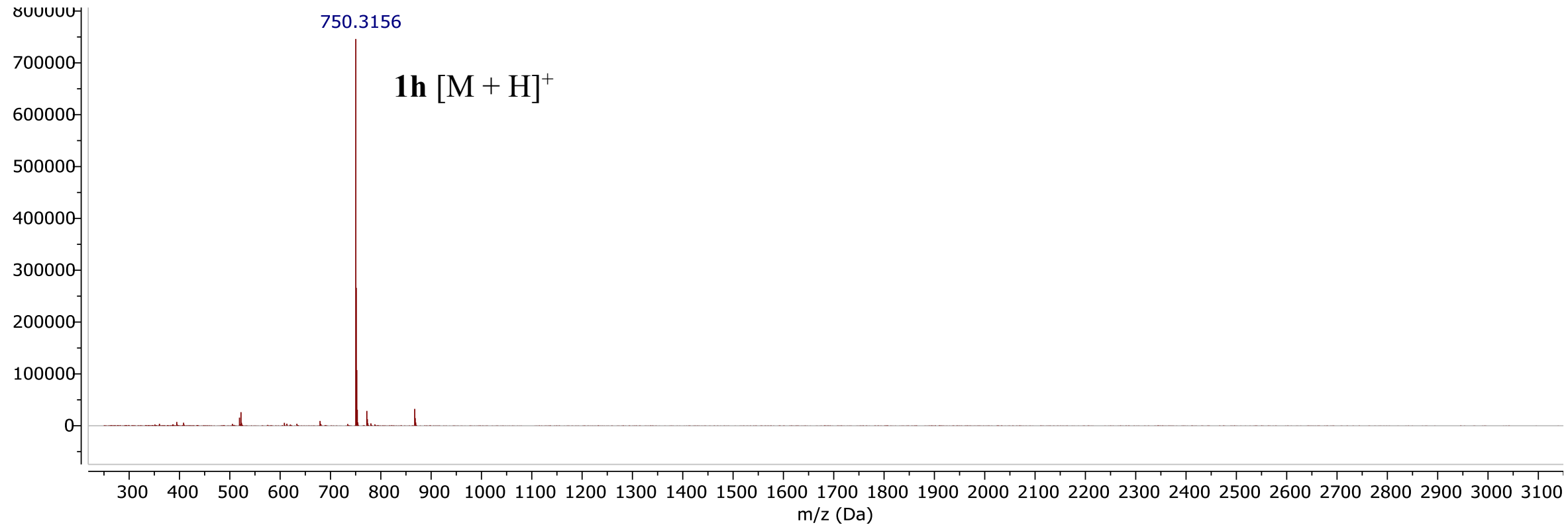

XII.d.3. Orthogonal Cleavage of Ethyl Containing Disulfide on **1h** in the Mixture of Peptides **1h** and **2d**.

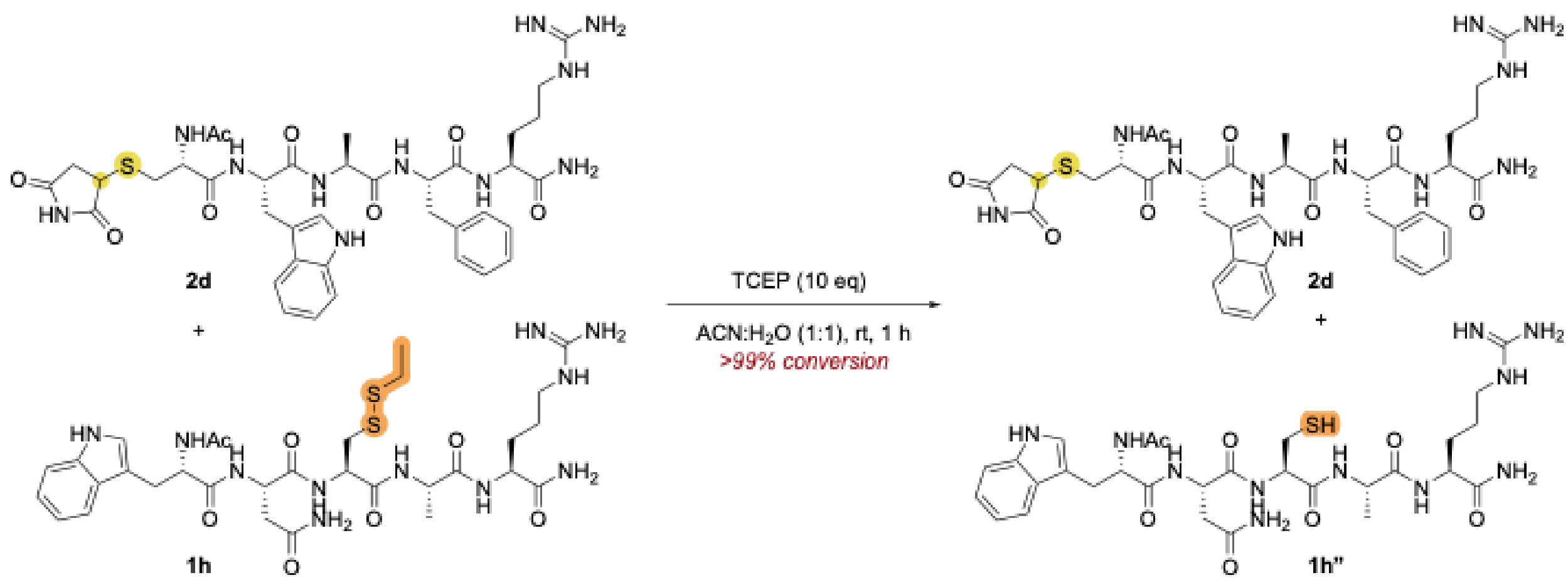

In a 1 dram vial equipped with a magnetic stirrer was added the purified peptide mixture Ac-C(maleimide)WAFR-$CONH_2$ **2d** and Ac-WNC(SEt)AR-$CONH_2$ **1h** were dissolved in ACN (300 µL), and H_2O (200 µL) while stirring. In a separate 1 dram vial, TCEP (3.7 mg, 0.013 mmol, 10 eq) was taken and dissolved in H_2O (100 µL) and the solution was added dropwise to the peptide solution and resulting mixture was allowed to stir 1 h at room temperature. After that, the selective reduced thiol product **1h"** (pale white solid, 1.7 mg, >99% conversion) was determined via analytical HPLC using 0.1% formic acid (FA) in both mobile phases A and B. Following analysis, products were subsequently purified using this same method.

Peptide Ac-WNCAR-$CONH_2$ 1h": HRMS (ESI) m/z: $C_{29}H_{44}N_{11}O_7S$ $[M+H]^+$ Calcd for 690.3140, Found 690.3140. **Peptide Ac-C(maleimide)WAFR-$CONH_2$ 2d: HRMS (ESI)** m/z: $C_{38}H_{50}N_{11}O_8S$ $[M+H]^+$ Calcd for 820.3559, Found 820.3556.

Analytical HPLC Method (C18, Water (A)/ACN (B); gradient: 5 to 85% B (0.1% formic acid in ACN) in 20 min, flow rate = 1.0 mL/min, I = 220 nm)**:** retention time of **1h"** and **2d** are 8.44 and 10.33 min respectively.

Analytical HPLC trace of the crude reaction mixture containing Ac-C(maleimide)WAFR-CONH$_2$ ***2d*** *and Ac-WNCAR-CONH$_2$* ***1h''***

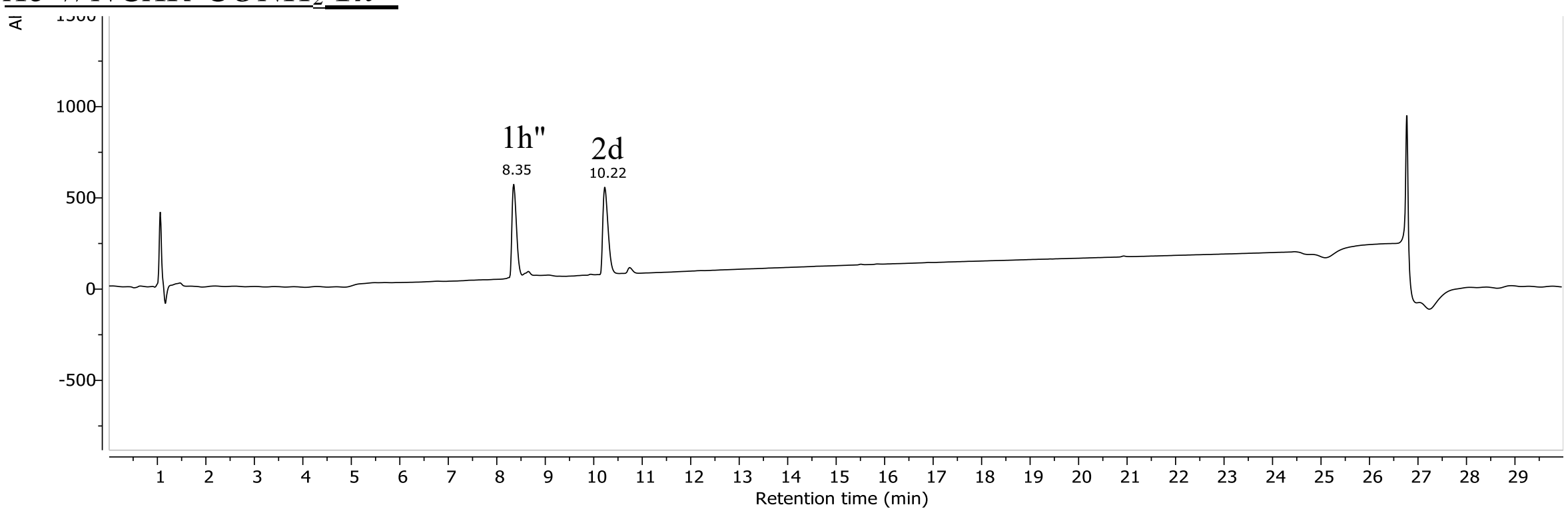

Analytical HPLC trace of purified peptide mixture of Ac-C(maleimide)WAFR-CONH$_2$ ***2d*** *and Ac-WNCAR-CONH$_2$* ***1h''***

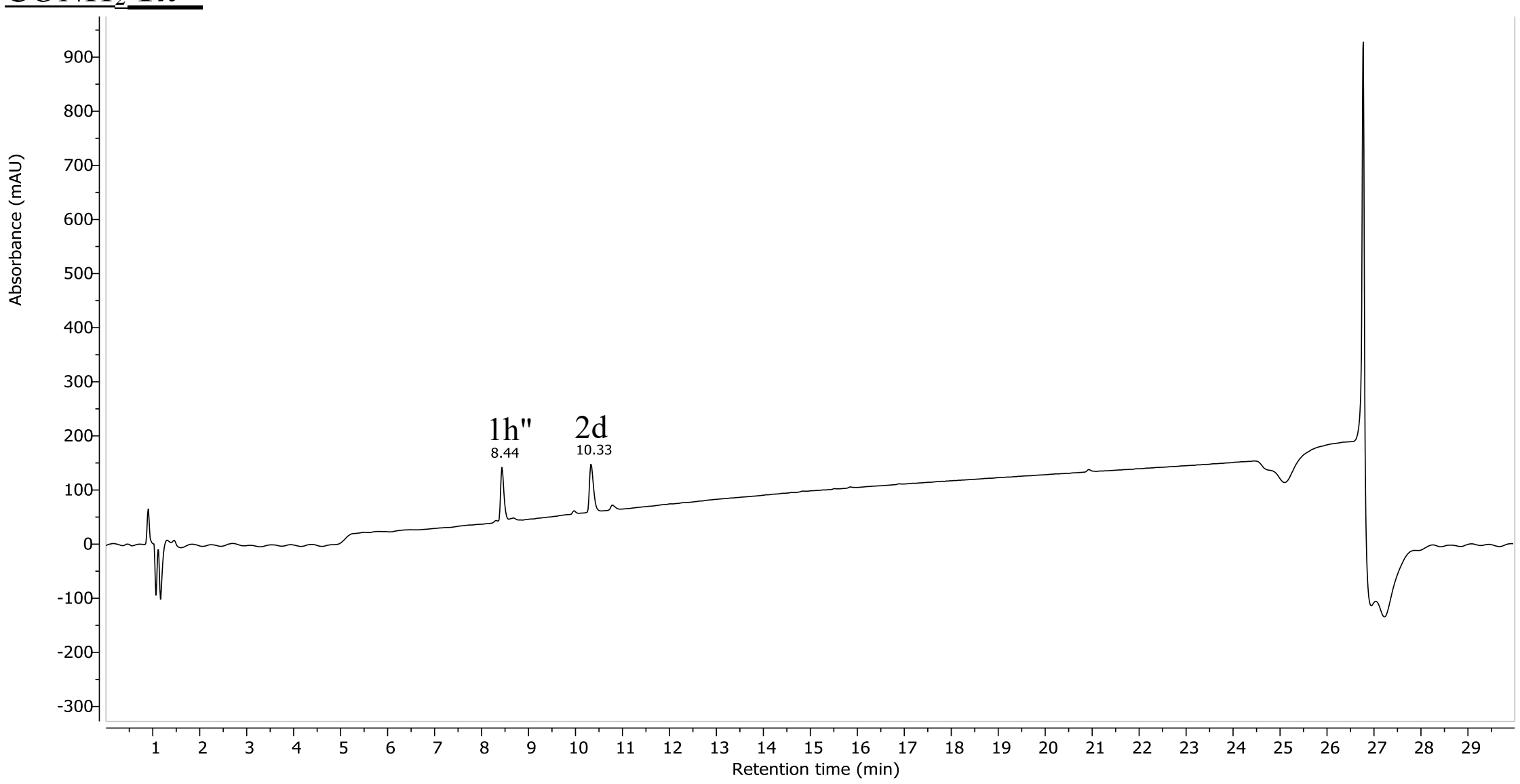

HRMS spectrum of purified peptide mixture of Ac-C(maleimide)WAFR-CONH$_2$ ***2d*** *and Ac-WNCAR-CONH$_2$* ***1h''***

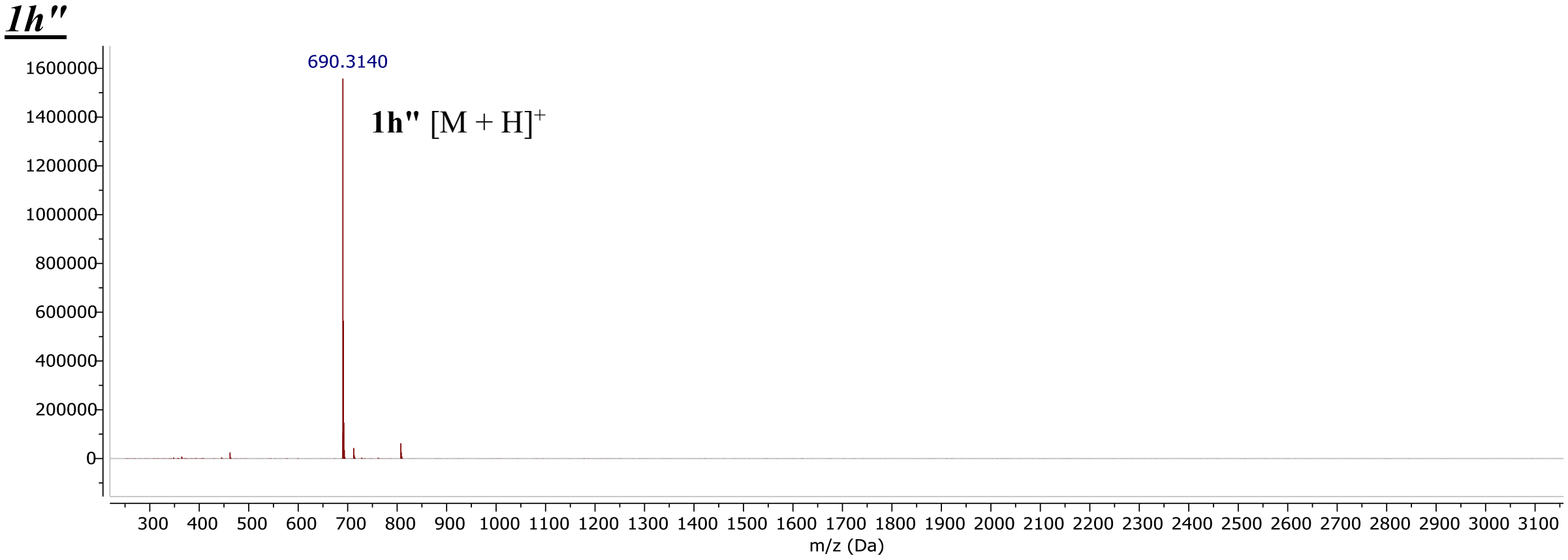

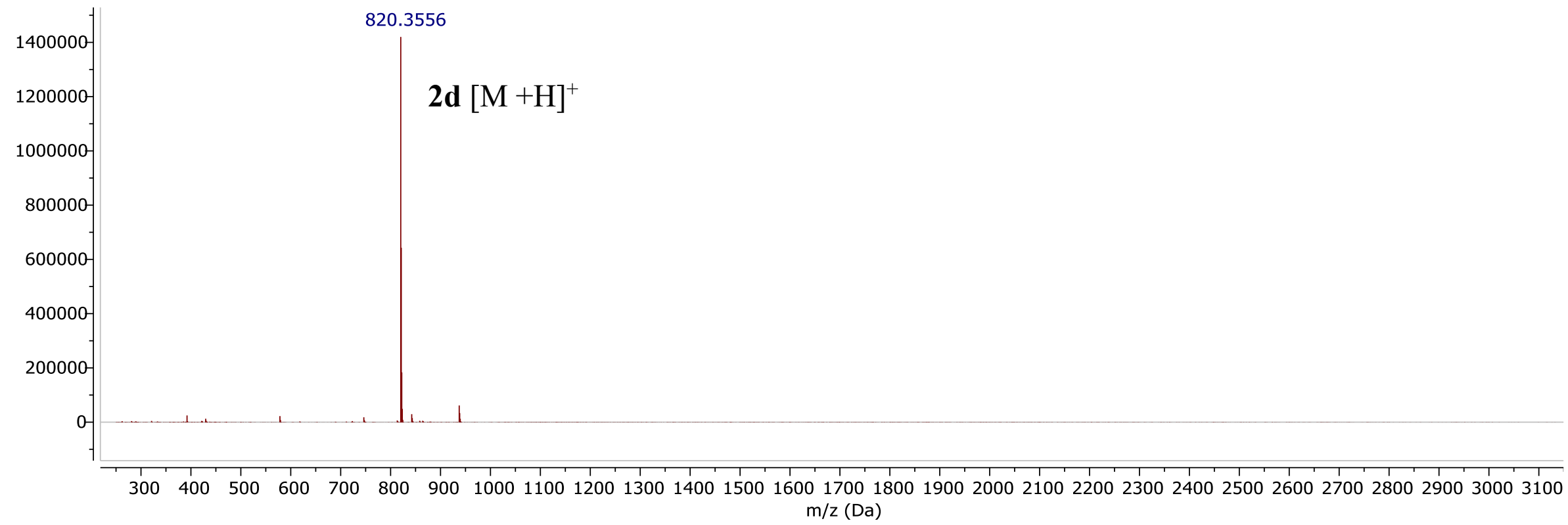

XII.d.4. Orthogonal Modification of Free Cys in Peptide **1h"** in the Mixture of **1h"** and **2d**.

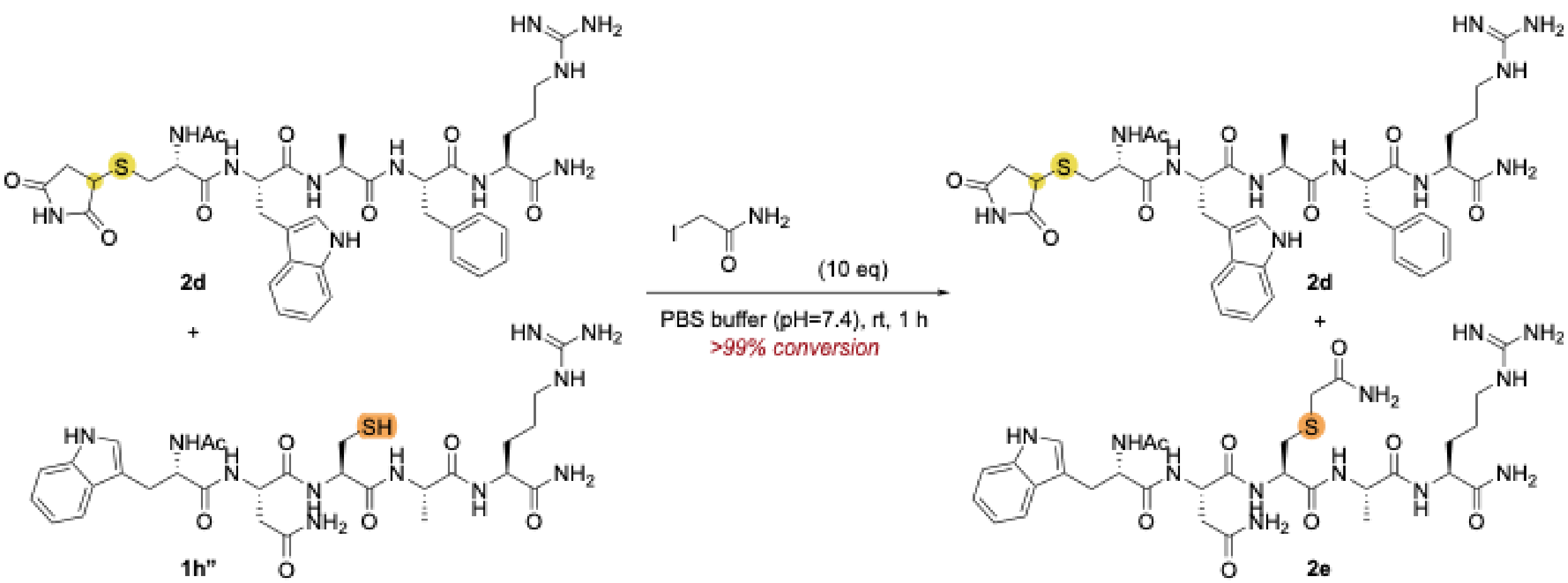

In a 1 dram vial equipped with a magnetic stirrer was added the purified peptide mixture Ac-C(maleimide)WAFR-CONH$_2$ **2d** and Ac-WNCAR-CONH$_2$ **1h"** were dissolved in PBS buffer (pH=7.4) (300 μL) while stirring. In a separate 1 dram vial, iodoacetamide (2.2 mg, 0.012 mmol, 10 eq) was taken and dissolved in PBS buffer (100 μL) and the solution was added dropwise to the peptide solution and resulting mixture was allowed to stir 1 h at room temperature. After that, the selective iodoacetamide adduct peptide **2e** (pale white solid, 1.8 mg, >99% conversion) was determined via analytical HPLC using 0.1% formic acid (FA) in both mobile phases A and B. Following analysis, product was subsequently purified using this same method.

Peptide Ac-WNC(iodoacetamide)AR-CONH$_2$ 2e: **HRMS (ESI)** m/z: $C_{31}H_{47}N_{12}O_8S$ [M+H]$^+$ Calcd for 747.3365, Found 747.3362. **Peptide Ac-C(maleimide)WAFR-CONH$_2$ 2d**: **HRMS (ESI)** m/z: $C_{38}H_{50}N_{11}O_8S$ [M+H]$^+$ Calcd for 820.3559, Found 820.3562.

Analytical HPLC Method (C18, Water (A)/ACN (B); gradient: 5 to 85% B (0.1% formic acid in ACN) in 20 min, flow rate = 1.0 mL/min, I = 220 nm)**:** retention time of **2e** and **2d** are 7.85 and 10.25 min respectively.

*Analytical HPLC trace of the crude reaction mixture containing Ac-C(maleimide)WAFR-$CONH_2$ **2d** and Ac-WNC(iodoacetamide)AR-$CONH_2$ **2e***

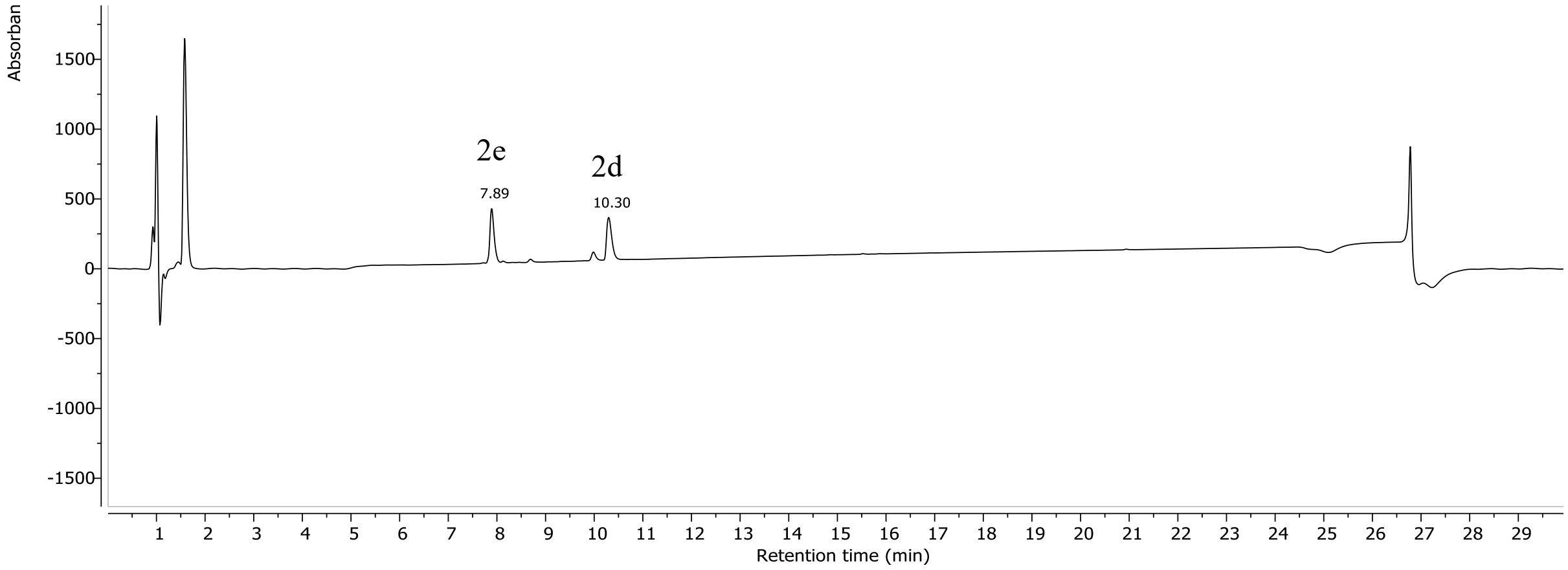

*Analytical HPLC trace of purified peptide mixture of Ac-C(maleimide)WAFR-$CONH_2$ **2d** and Ac-WNC(iodoacetamide)AR-$CONH_2$ **2e***

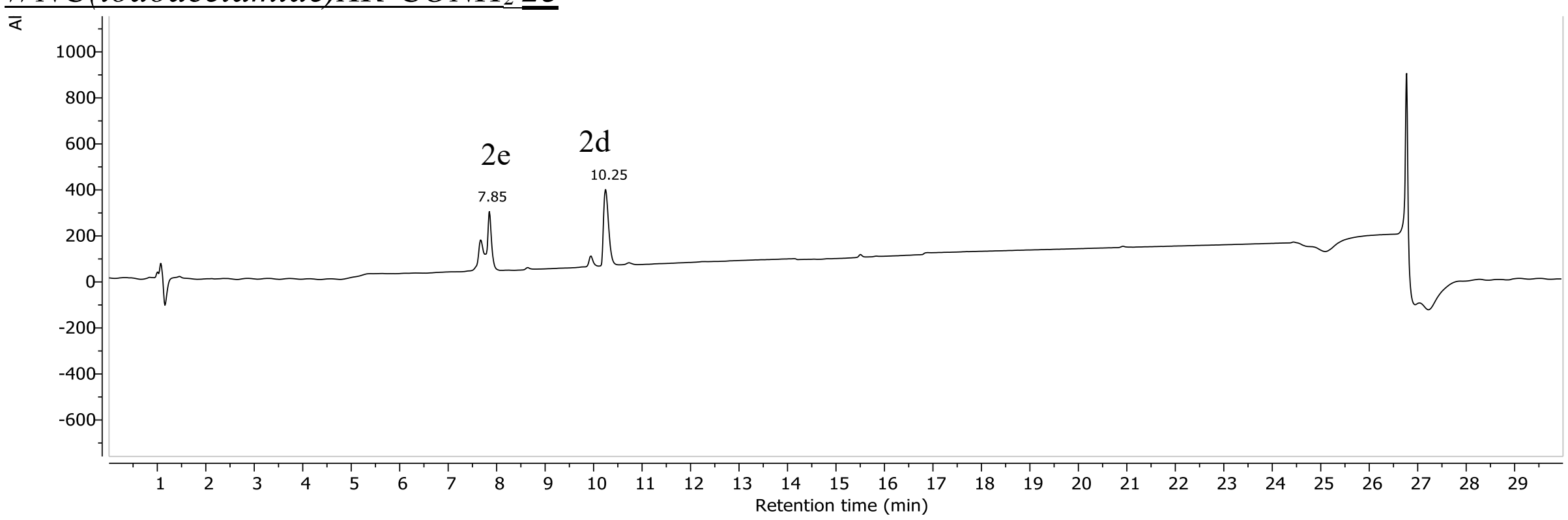

*HRMS spectrum of purified peptide mixture of Ac-C(maleimide)WAFR-$CONH_2$ **2d** and Ac-WNC(iodoacetamide)AR-$CONH_2$ **2e***

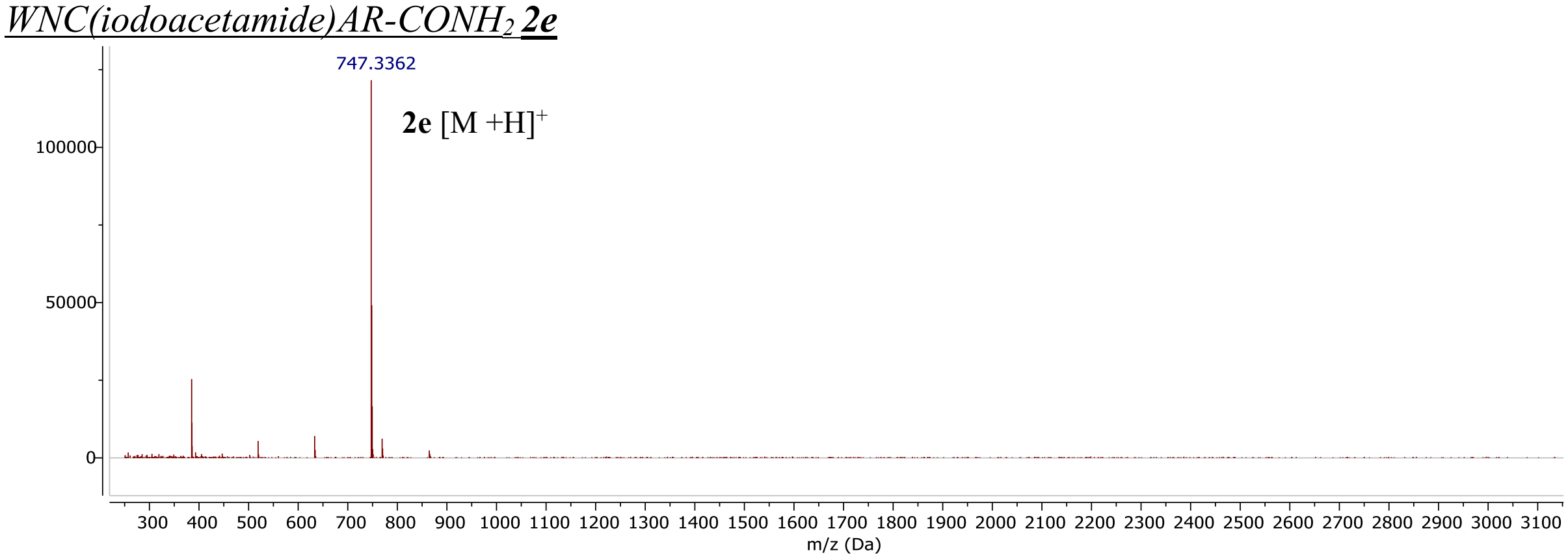

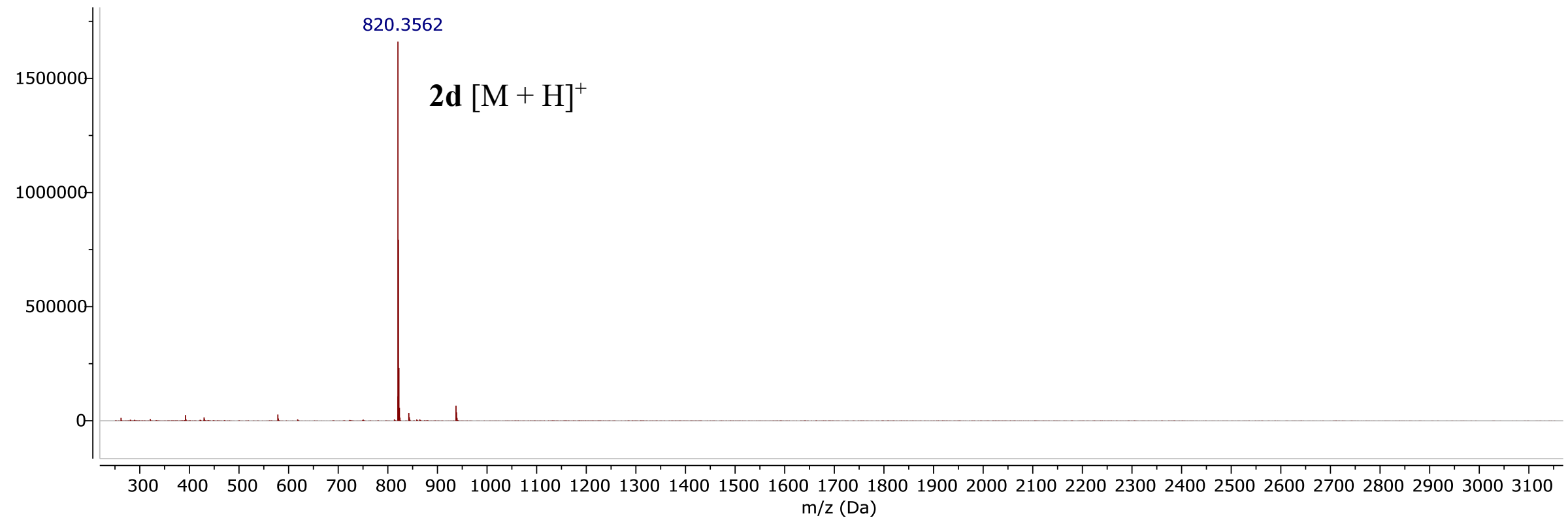

XIII. Supplementary Figure 6. Synthesis of Methyl *N*-acetyl-*S*-(phenylthio)-*L*-cysteinate **1i**.

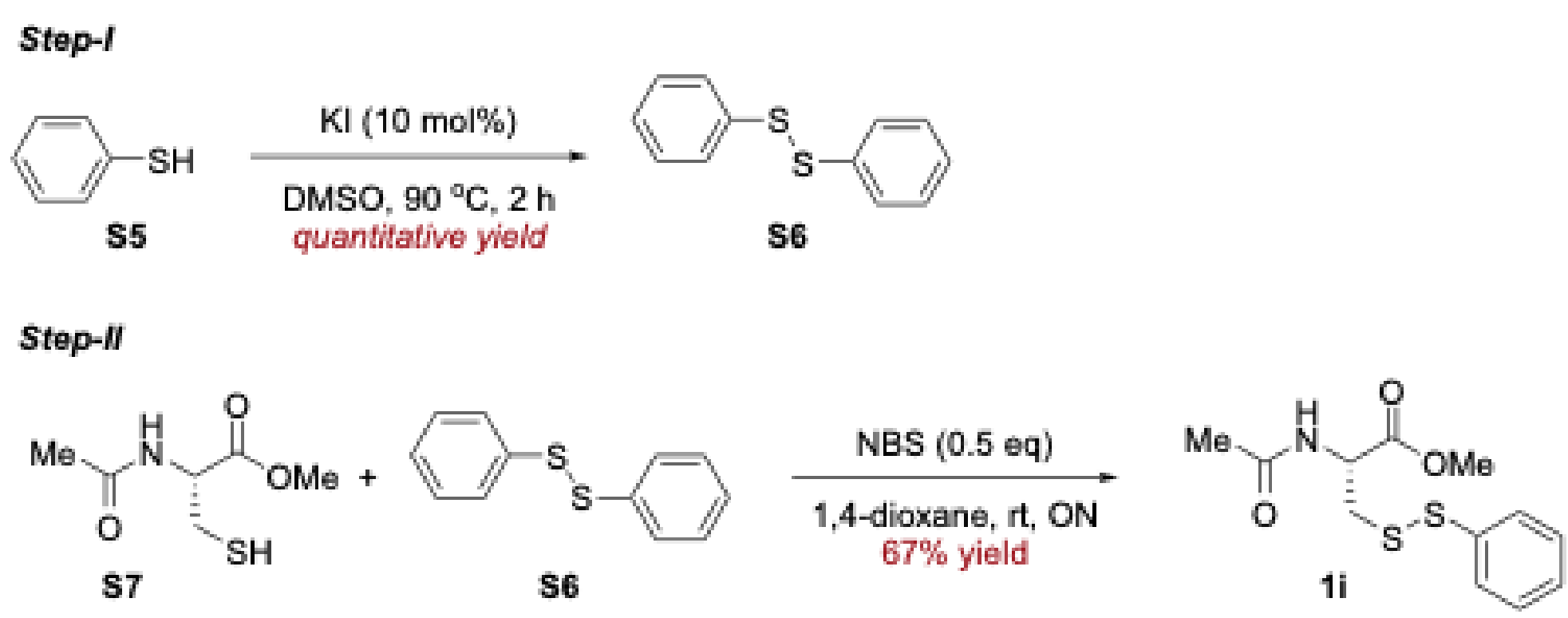

Compound **1i** was synthesized according to the modified literature procedure.

Step-I: *Synthesis of 1,2-diphenyldisulfane:*[3]

In a 25 mL round bottom flask equimolar mixture of thiophenol **S5** (0.5 mL, 5 mmol, 1 eq) and dimethylsulfoxide (DMSO, 0.35 mL, 5 mmol, 1 eq) were added followed by addition of potassium iodide (KI, 83 mg, 0.5 mmol, 0.1 eq) and left the reaction mixture stirred at 90 °C in oil bath for 2 h. Then the reaction mixture was cooled to room temperature and diluted with diethyl ether. The organic layer was washed with water (10 mL x 5). The accumulated organic layer was dried over anhydrous sodium sulfate and concentrated under vacuum to afford the desired product 1,2-diphenyldisulfane (**S6**, 544 mg, off white solid) in quantitative yield. **^{1}H NMR** (400 MHz, $CDCl_3$) δ (ppm): 7.52 (d, *J* = 7.1 Hz, 4H), 7.32 (t, *J* = 7.5 Hz, 4H), 7.24 (t, *J* = 7.3 Hz, 2H).

Step-II: *Synthesis of methyl N-acetyl-S-(phenylthio)-L-cysteinate:*[3]

In a 50 mL Schlenk tube, methyl acetyl-*L*-cysteinate **S7** (177 mg, 1 mmol, 1 eq), diphenyldisulfane **S6** (436 mg, 2 mmol, 2 eq) and *N*-bromosuccinimide (89 mg, 0.5 mmol, 0.5 eq) were taken and the tube was evacuated followed by backfilled with nitrogen three times. Then 1,4-dioxane (10 mL) was added to the tube under positive pressure of nitrogen and the solution was stirred for overnight at room temperature. After complete consumption of starting material **S7** the reaction mixture was diluted with ethyl acetate and transferred to a 50 mL round bottom flask. The organic layer was washed with saturated $NaHCO_3$ (20 mL x 3) followed by brine solution (20 mL). The accumulated organic layer was evaporated to dryness under vacuum, and the crude reaction mixture was purified by silica gel column chromatography using 60% ethyl

acetate-hexane as eluent to afford the desired product methyl *N*-acetyl-*S*-(phenylthio)-*L*-cysteinate **1i** (191 mg, pale yellow solid, 67% yield). $R_f = 0.3$ (in 50% ethyl acetate-hexane). **^{1}H NMR (400 MHz, $CDCl_3$)** δ (ppm): 7.52 – 7.49 (m, 2H), 7.35 (t, *J* = 7.4 Hz, 2H), 7.28 – 7.24 (m, 1H), 6.28 (d, *J* = 7.5 Hz, 1H), 4.89 (dt, *J* = 7.5, 4.9 Hz, 1H), 3.74 (s, 3H), 3.29 – 3.20 (m, 2H), 1.94 (s, 3H). **^{13}C {^{1}H} NMR (101 MHz, $CDCl_3$)** δ (ppm) 170.9, 169.9, 136.8, 129.3, 128.5, 127.6, 52.8, 51.8, 40.8, 23.1.

XIV. Supplementary Figure 7. Synthesis of tris(1-(2-Nitrophenyl)ethyl)phosphite **S10**.[4]

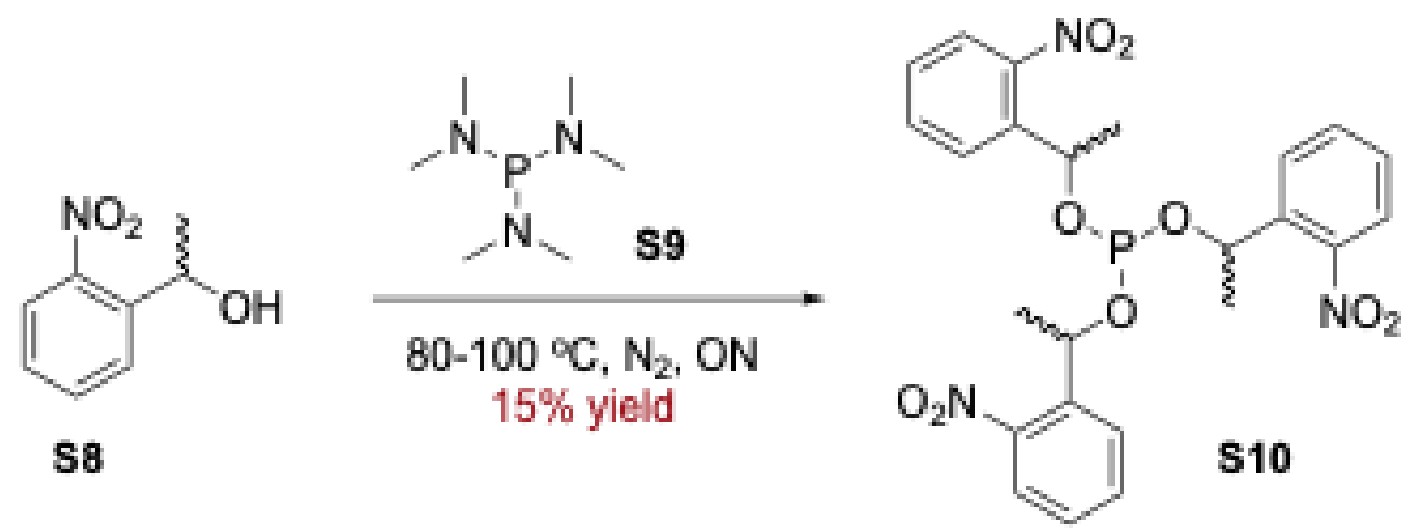

In a 25 mL Schlenk tube, 1-(2-nitrophenyl)ethan-1-ol **S8** (830 mg, 4.9 mmol, 3 eq) was taken and the tube was evacuated followed by backfilled with nitrogen three times. The compound was then heated to 80 ºC in oil bath under positive pressure of nitrogen for 10 min while stirring followed by dropwise addition of *N*,*N*,*N*',*N*',*N*'',*N*''-hexamethylphosphanetriamine **S9** (0.3 ml, 1.65 mmol, 1 eq) at the same temperature under N_2. After that, the temperature was increased to 100 ºC and left the reaction mixture stirred for overnight in the oil bath. During this process the colour of the solution was turned blackish brown from greenish. After completion, the system was cooled to room temperature, the solution was diluted with ethyl acetate and transferred to 50 mL round bottom flask. The solvent was evaporated to dryness under vacuum, and the crude product was isolated through silica gel column chromatography using 7-8% ethyl acetate-hexane as eluent to afford the desired product tris(1-(2-nitrophenyl)ethyl) phosphite **S10** (131 mg, yellowish liquid, 15% yield). $R_f = 0.6$ (in 20% ethyl acetate-hexane).

^{1}H NMR (400 MHz, CD_3CN) δ (ppm): 7.87 – 7.83 (m, 6H), 7.79 – 7.76 (m, 3H), 7.73 (dd, *J* = 8.2, 1.3 Hz, 3H), 7.65 – 7.56 (m, 12H), 7.54 – 7.50 (m, 6H), 7.49 – 7.32 (m, 18H), 5.73 – 5.58 (m, 12H), 1.45 (d, *J* = 6.4 Hz, 9H), 1.37 (dd, *J* = 6.4, 4.3 Hz, 18H), 1.22 (d, *J* = 6.4 Hz, 9H). **^{13}C {^{1}H} NMR (101 MHz, CD_3CN)** δ (ppm): 147.8 (d), 147.6, 147.3, 139.99, 139.97, 139.95, 139.93, 139.91, 139.8 (d), 134.7, 134.6, 129.4, 129.3, 129.2, 128.9 (d), 128.8, 128.6, 125.00, 124.97 (d), 67.8 (d), 67.6, 67.5, 67.4, 25.2 (d), 25.12 (d), 25.05 (d), 24.9 (d). **^{31}P NMR (162 MHz, CD_3CN)** δ (ppm): 137.8 (q, *J* = 8.8 Hz), 135.9 (q, *J* = 8.8 Hz).

Caution: Aromatic Phosphite S10 is unstable in room temperature and moisture sensitive. It should have been stored under argon in cold condition (2-4 ºC) immediately after purification. Under air, it converts to higher oxidized species P(V). It is also advised to check ^{31}P NMR each time before performing the reaction to confirm its oxidation state.

*<u>Analytical HPLC trace of tris(1-(2-nitrophenyl)ethyl) phosphite **S10** in DMF</u>*
(* = side products from **S10**)

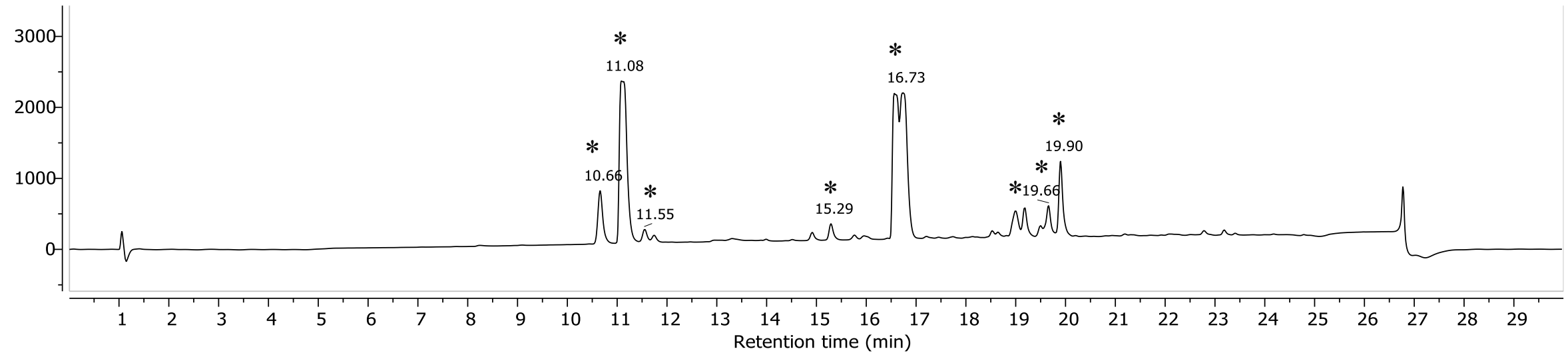

XV. Supplementary Figure 8. Reaction of Aryl-Phosphite **S10** on Methyl *N*-acetyl-*S*-(phenylthio)-*L*-cysteinate **1i**.

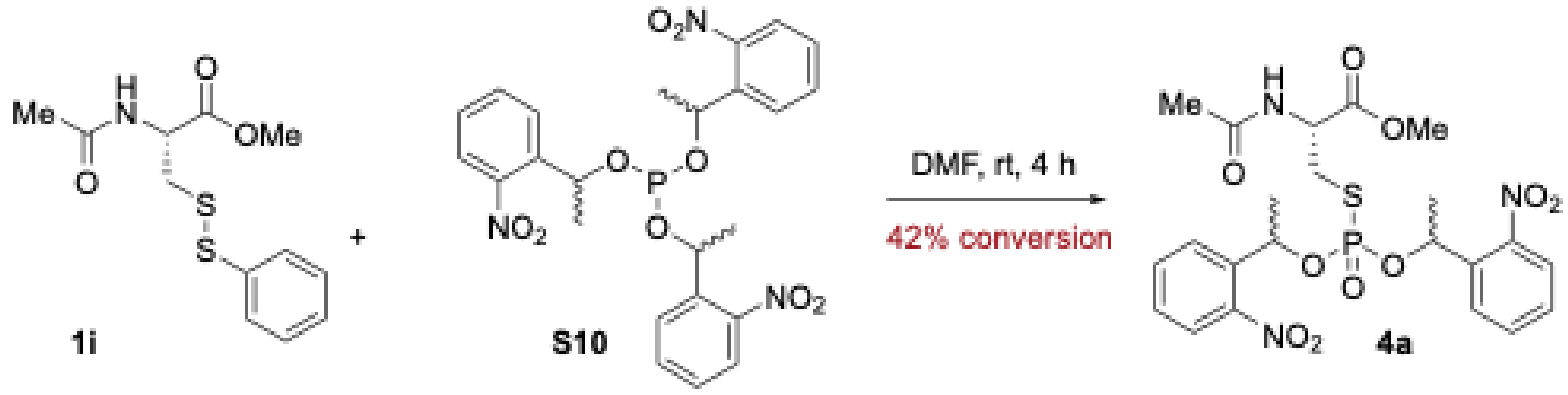

Purified amino-acid disulfide methyl *N*-acetyl-*S*-(phenylthio)-*L*-cysteinate **1i** (2.85 mg, 0.01 mmol, 1 eq) and tris(1-(2-nitrophenyl)ethyl) phosphite **S10** (52 mg, 0.1 mmol, 10 eq) were added to a 1 dram vial and dissolved in 500 µL DMF. 60 µL aliquot was taken after 4 h and diluted with 500 µL of (ACN:H_2O = 1:1 with 0.1% Formic acid). The resulting crude mixture was analyzed via **Analytical HPLC Method** (C18, Water (A)/ACN (B); gradient: 5 to 85% B (0.1% formic acid in ACN) in 20 min, flow rate = 1.0 mL/min, I = 220 nm) and resulted the conversion 42%. Under these conditions, the amino-acid disulfide **1i** and product methyl *N*-acetyl-*S*-(bis(1-(2-nitrophenyl)ethoxy)phosphoryl)-*L*-cysteinate **4a** exhibited retention times of **14.05** min and **16.42** min, respectively. **HRMS (ESI)** m/z: for $C_{22}H_{27}N_3O_{10}PS$ $[M+H]^+$ Calcd for 556.1149, Found 556.1143.

*<u>Analytical HPLC trace of tris(1-(2-nitrophenyl)ethyl) phosphite **S10** in DMF</u>*
(* = side products from **S10**)

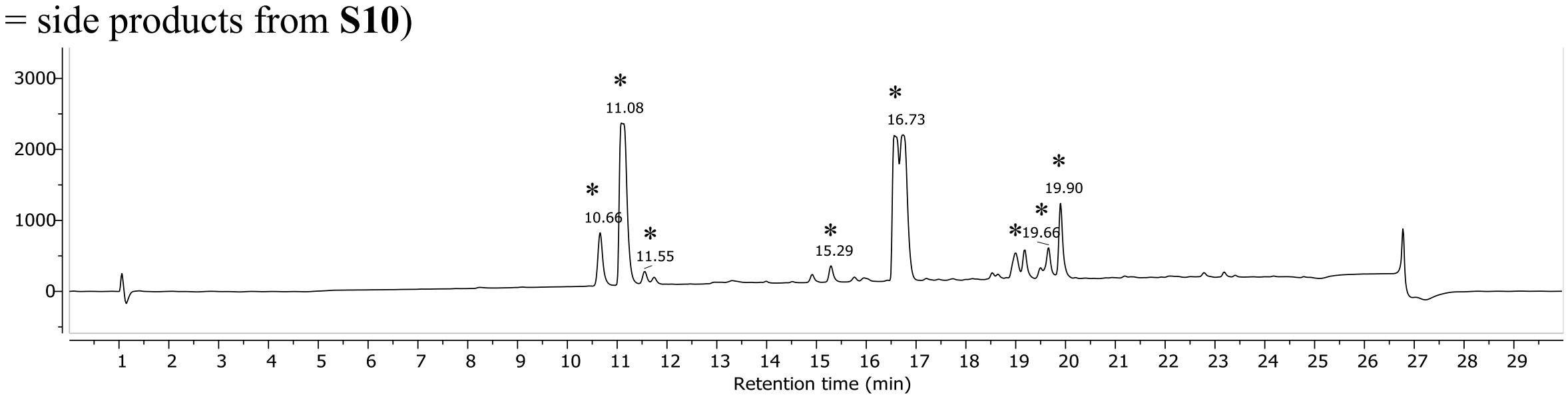

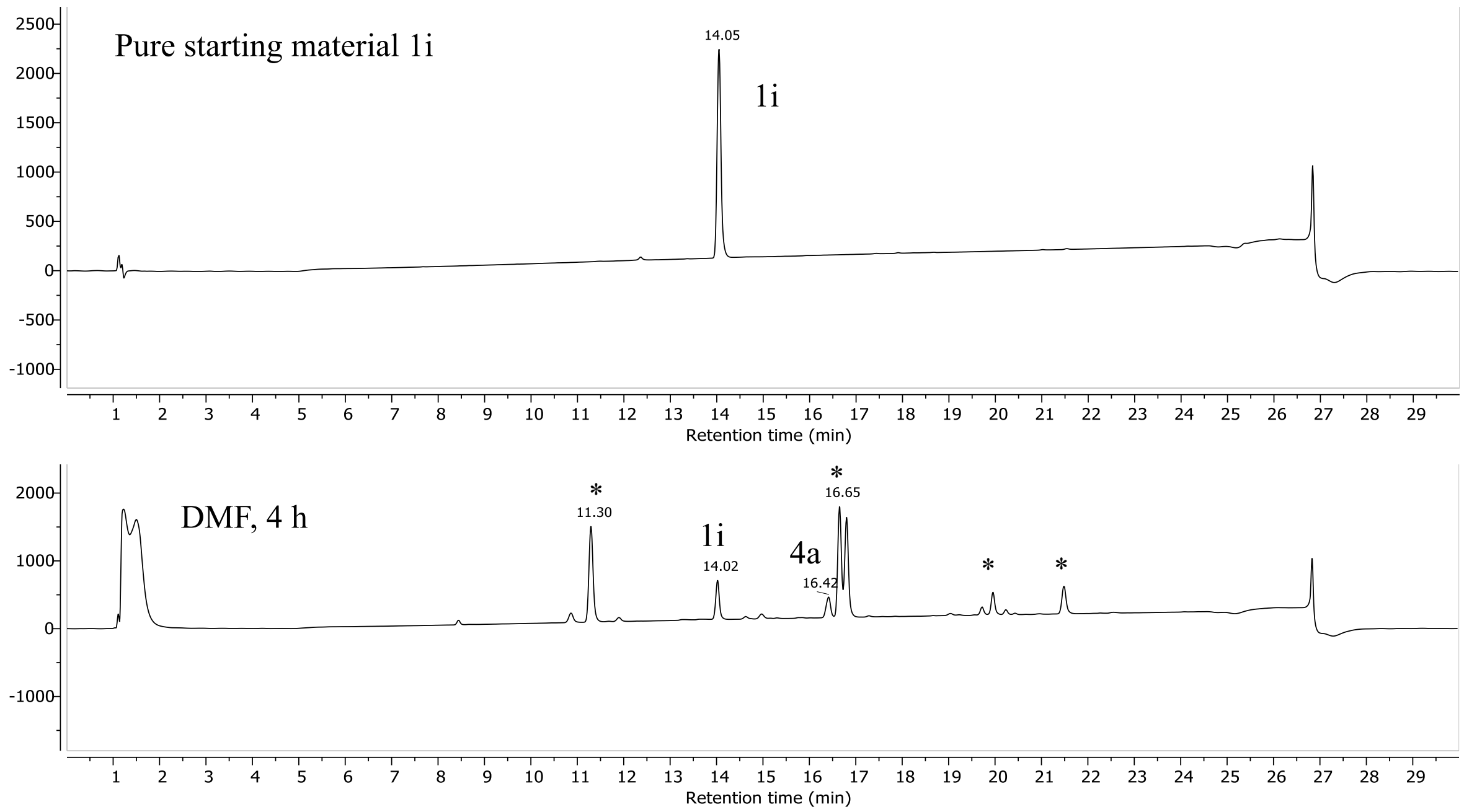

(* = side products from **S10**)

*HRMS spectrum of purified **4a***

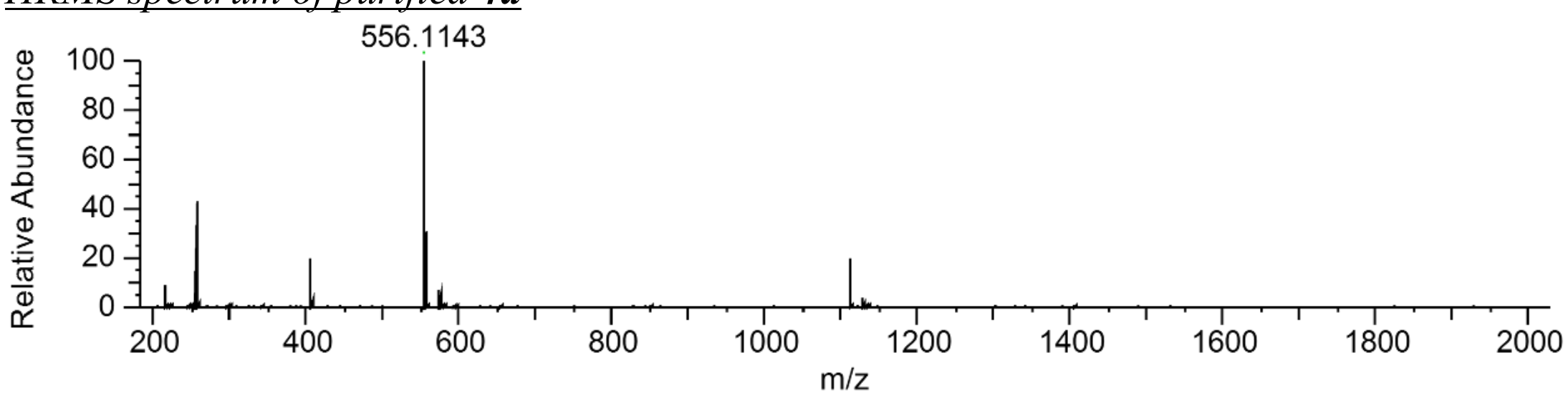

XV.1. Large Scale Synthesis of Methyl N-Acetyl-S-(bis(1-(2-nitrophenyl)ethoxy)phosphoryl)-L-cysteinate **4a**.

In a 25 mL round bottom flask methyl *N*-acetyl-*S*-(phenylthio)-*L*-cysteinate **1i** (62 mg, 0.22 mmol, 1 eq) was taken and dissolved in 2 mL of *N,N*-dimethylformamide (DMF) while stirring. To this solution, previously soluble tris(1-(2-nitrophenyl)ethyl) phosphite **S10** (233 mg, 0.44 mmol, 2 eq dissolved in 1 mL of DMF) was added dropwise at room temperature and left the reaction mixture stirred for 4 h. After complete consumption of **1i** (monitered by TLC), reaction mixture was evaporated to dryness under vacuum and the crude product was purified through silica gel column chromatography using 50 % ethyl acetate-hexane as eluent to afford the desired product methyl *N*-acetyl-*S*-(bis(1-(2-nitrophenyl)ethoxy)phosphoryl)-*L*-cysteinate **4a** (40 mg, colorless liquid, 36% yield). $R_f = 0.3$ (in 70% ethyl acetate-hexane). **^{1}H NMR (600 MHz, CD_3CN)** δ (ppm): 7.98 (d, $J = 8.3$ Hz, 1H), 7.95 (m, 3H), 7.75 (t, $J = 7.7$ Hz, 1H), 7.59 (m, 6H), 7.58 (m, 2H), 7.49 (m, 3H), 7.08 – 6.94 (m, 3H), 6.06 – 5.93 (m, 4H), 4.61 (td, $J = 7.7, 4.8$ Hz, 1H), 4.55 (td, $J = 7.7, 4.7$ Hz, 1H), 4.43 (m, 2H), 3.68 (d, $J = 13.6$ Hz, 3H), 3.63 (d, $J = 7.6$ Hz, 3H), 3.20 – 3.17 (m, 1H), 3.14 (dd, $J = 10.7, 4.7$ Hz, 1H), 3.10 (ddd, $J = 15.8, 5.7, 1.9$ Hz, 1H), 3.07 – 3.06 (m, 1H), 3.05 (m, 2H), 2.94 – 2.87 (m, 2H), 1.90 (d, $J = 11.4$ Hz, 3H), 1.86 (d, $J = 9.5$ Hz, 3H), 1.65 (d, $J = 7.8$ Hz, 3H), 1.62 – 1.59 (m, 7H). **^{13}C {^{1}H} NMR (151 MHz, CD_3CN)** δ (ppm): 171.2, 171.15 (d), 170.9, 170.83 (d), 147.97

(d), 137.7, 135.17 (d), 135.11 (t), 130.32 (d), 130.24 (d), 128.91 (d), 128.83 (d), 128.8, 125.44, 125.39, 73.79 (t), 73.67 (d), 73.60 (d), 73.56 (d), 73.50 (d), 73.5, 53.6, 53.6, 53.5, 53.14 (t), 32.56 (d), 32.5, 32.4, 32.4, 24.46 (d), 24.4, 24.36 (d), 22.73 (dd). **^{31}P NMR (243 MHz, CD_3CN)** δ (ppm): 26.62, 25.89, 25.35. **HRMS (ESI)** m/z: for $C_{22}H_{27}N_3O_{10}PS$ $[M+H]^+$ Calcd for 556.1149, Found 556.1143.

*HRMS spectrum of purified **4a***

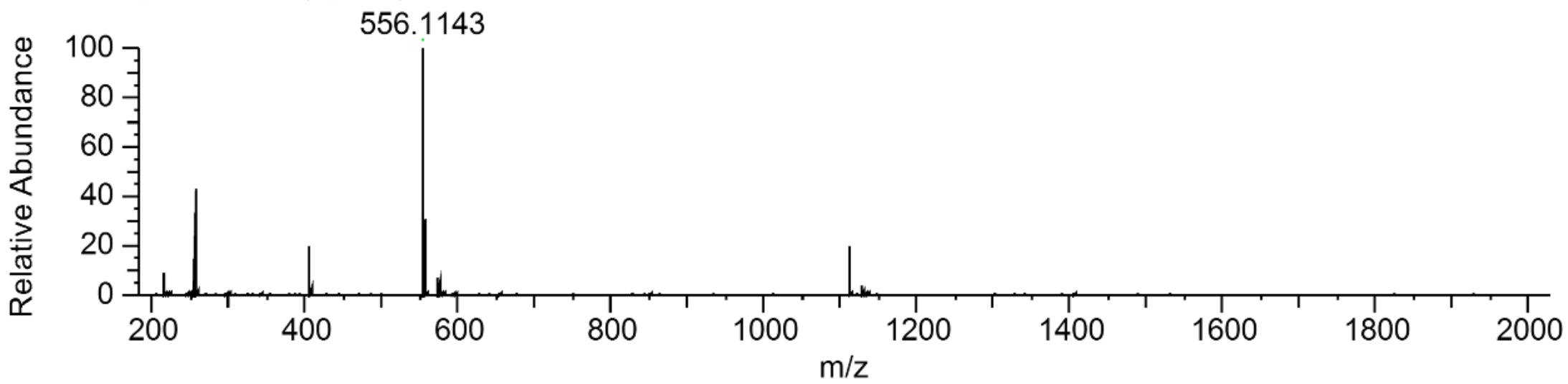

XVI. Supplementary Figure 9. Reaction of alkyl-phosphite S11 on Methyl *N*-acetyl-*S*-(phenylthio)-*L*-cysteinate **1i**.

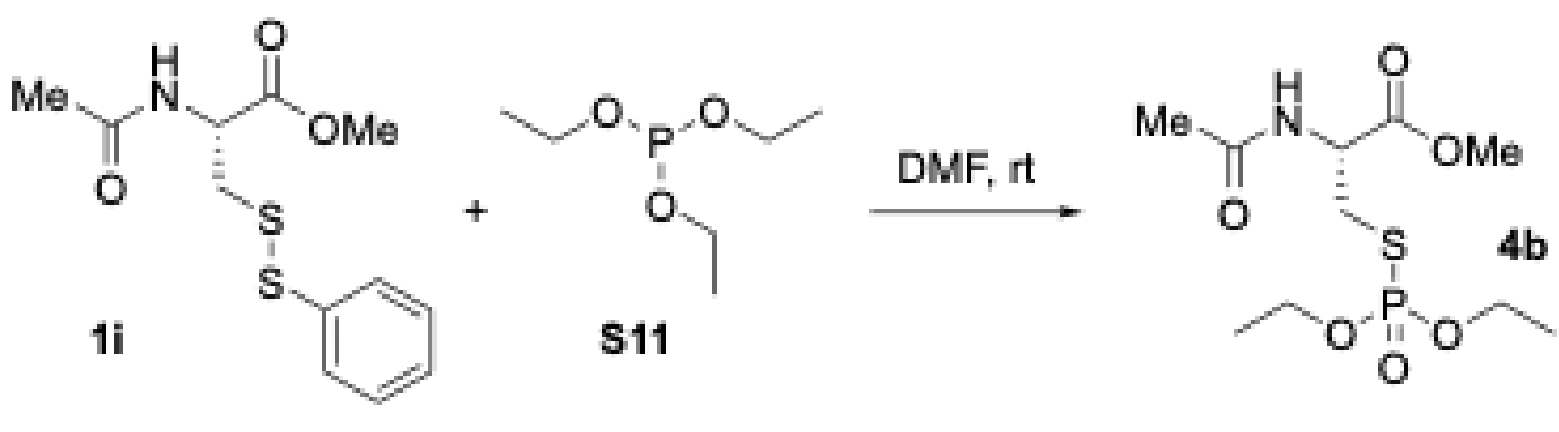

Entry	Time (h)	Conversion (%)
1	4	26
2	24	100

Purified amino-acid disulfide methyl *N*-acetyl-*S*-(phenylthio)-*L*-cysteinate **1i** (2.85 mg, 0.01 mmol, 1 eq) and triethyl phosphite **S11** (17 mg, 0.1 mmol, 10 eq) were added to a 1 dram vial and dissolved in 500 µL DMF for screening. 60 µL aliquot was taken after 4 h, 24 h separately and diluted with 500 µL of (ACN:H_2O = 1:1). The resulting crude mixtures were analyzed via **Analytical HPLC Method** (C18, Water (A)/ACN (B); gradient: 5 to 85% B (0.1% formic acid in ACN) in 20 min, flow rate = 1.0 mL/min, I = 220 nm) to determine the conversion. Under these conditions, the amino-acid disulfide **1i** and product methyl *N*-acetyl-*S*-(diethoxyphosphoryl)-*L*-cysteinate **4b** exhibited retention times of 14.14 min and 15.01 min, respectively.

*<u>Analytical HPLC trace of the crude reaction mixture converting Ac-C(SPh)-OMe **1i** to Ac-C(P(O)(OEt)$_2$)OMe **4b**</u>*

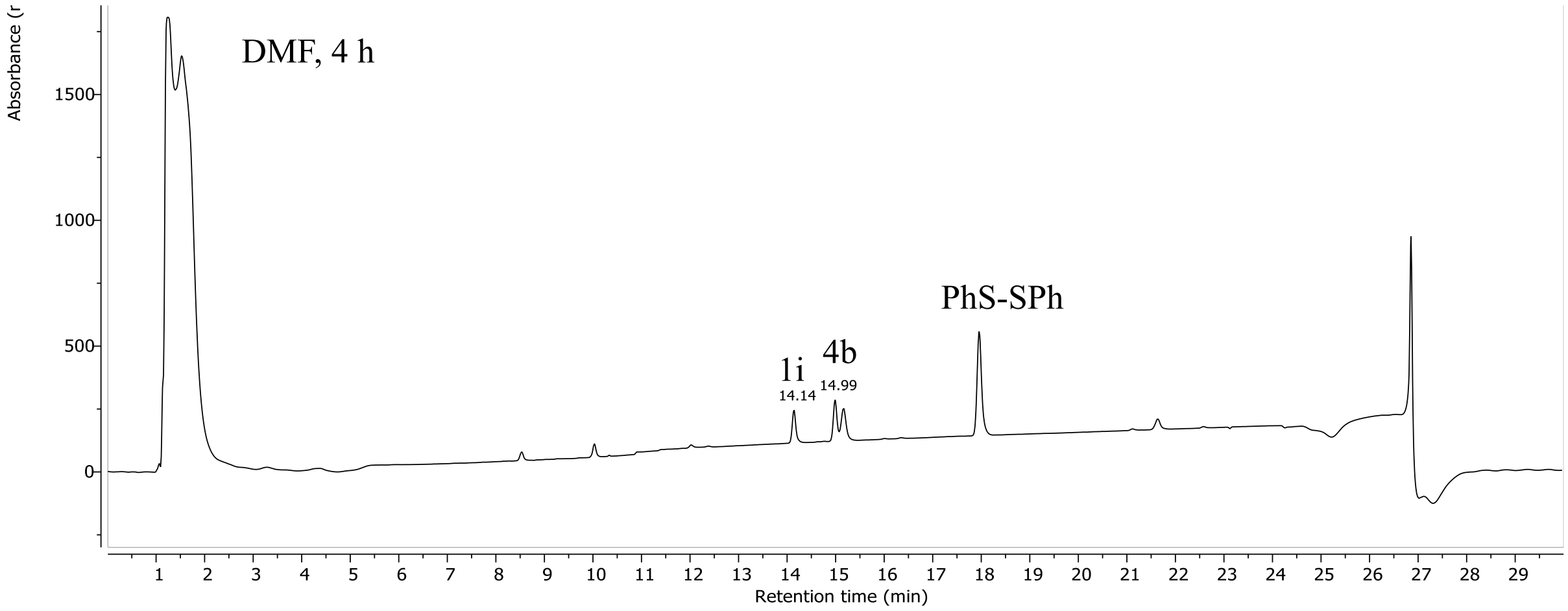

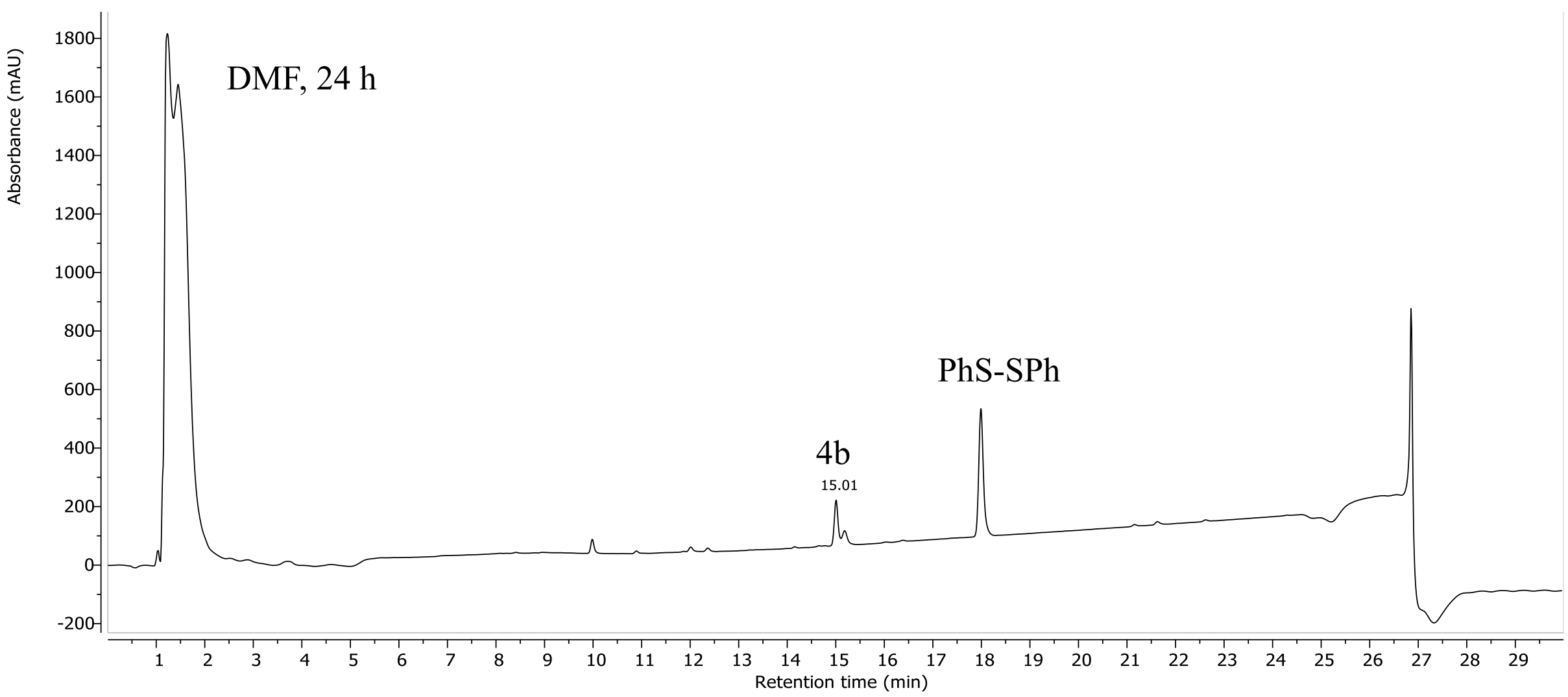

XVI.1. Large Scale Synthesis of methyl *N*-acetyl-*S*-(diethoxyphosphoryl)cysteinate **4b**.

In a 25 mL round bottom flask methyl *N*-acetyl-*S*-(phenylthio)-*L*-cysteinate **1i** (62 mg, 0.22 mmol, 1 eq) was taken and dissolved in 2 mL of *N,N*-dimethylformamide (DMF) while stirring. To this solution, triethyl phosphite **S11** (0.35 mL, 2 mmol, 10 eq) was added dropwise at room temperature and left the reaction mixture stirred for 24 h. After complete consumption of **1i**, reaction mixture was evaporated to dryness under vacuum and the crude product was purified through **Preparatory HPLC Method** (C18, Water (A)/ACN (B); gradient: 10 to 65% **B** (0.1 % FA) in 35 min; 65 to 90% **B** (0.1 % FA) in 35.1 to 45 min, flow rate = 4.7 mL/min, I = 220 nm) to obtain the desired product **4b** (colorless liquid, 46 mg) in 74% conversion. **^{1}H NMR** (400 MHz, DMSO-d_6) δ (ppm): 8.48 (d, *J* = 7.8 Hz, 1H), 4.51 (ddd, *J* = 8.8, 7.9, 5.2 Hz, 1H), 4.12 – 4.02 (m, 4H), 3.65 (s, 3H), 3.17 (td, *J* = 13.4, 5.2 Hz, 1H), 3.02 (ddd, *J* = 15.6, 13.2, 8.8 Hz, 1H), 1.87 (s, 3H), 1.27 (t, *J* = 7.1 Hz, 6H). **^{13}C {^{1}H} NMR** (101 MHz, DMSO-d_6) δ (ppm): 170.4, 169.5, 63.44, 63.39, 52.29, 52.27, 52.2, 31.20, 31.16, 22.3, 15.84, 15.77. **^{31}P NMR** (162 MHz, DMSO-d_6) δ (ppm): 25.84 (tq, J = 18.1, 9.0, 8.5 Hz). **HRMS (ESI)** m/z: for $C_{10}H_{21}NNaO_6PS$ [M + Na]$^+$ Calcd for 336.0641, Found 336.0651.

*HRMS spectrum of purified **4b***

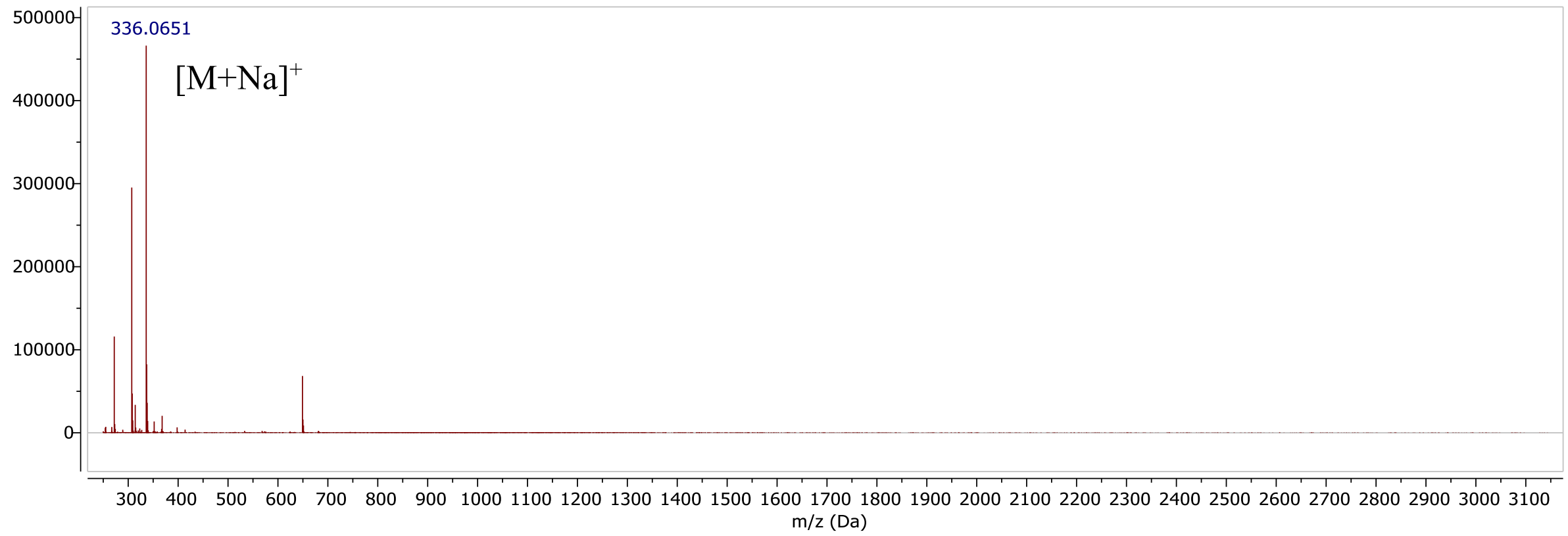

XVII. Reaction of Aryl-Phosphite **S10** on Aryl-Disulfide Contain Peptide **1g**.

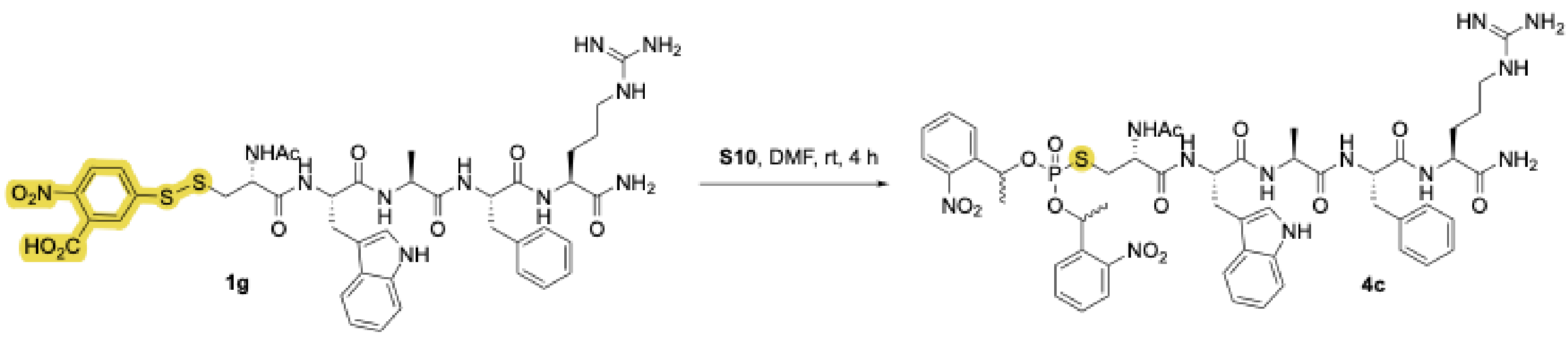

Purified amino-acid aryl-disulfide Ac-C(SAr)WAFR-CONH$_2$ **1g** (1 mg, 0.001 mmol, 1 eq) and different equivalent of tris(1-(2-nitrophenyl)ethyl) phosphite **S10** were added to different 1 dram vial and dissolved in 500 µL DMF to each. 60 µL aliquot was taken after 4 h and diluted with 500 µL of (ACN:H$_2$O = 1:1). The resulting crude mixture was analyzed via **Analytical HPLC Method** (C18, Water (A)/ACN (B); gradient: 5 to 85% B (0.1% formic acid in ACN) in 20 min, flow rate = 1.0 mL/min, I = 220 nm) and resulted the conversion (pale yellow solid, 0.24 mg, >22% conversion) and (pale yellow solid, 1.1 mg, >99% conversion) for 1.7 eq and 10 eq of **S10** respectively. Under these conditions, the amino-acid disulfide **1g** and phosphorylated product **4c** were exhibited retention times of 12.05 min and 14.71 min, respectively.

Peptide Ac-C(P(O)(OAr^1)$_2$)WAFR-CONH$_2$ 4c: **HRMS (ESI)** m/z: $C_{50}H_{62}N_{12}O_{13}PS$ [M+H]$^+$ Calcd for 1101.4012, Found 1101.4019. **Analytical HPLC Method** (C18, Water (A)/ACN (B); gradient: 5 to 85% B (0.1% formic acid in ACN) in 20 min, flow rate = 1.0 mL/min, I = 220 nm)**:** retention time of 14.71 min.

*Analytical HPLC trace of tris(1-(2-nitrophenyl)ethyl) phosphite **S10** in DMF*

(* = side products from **S10**)

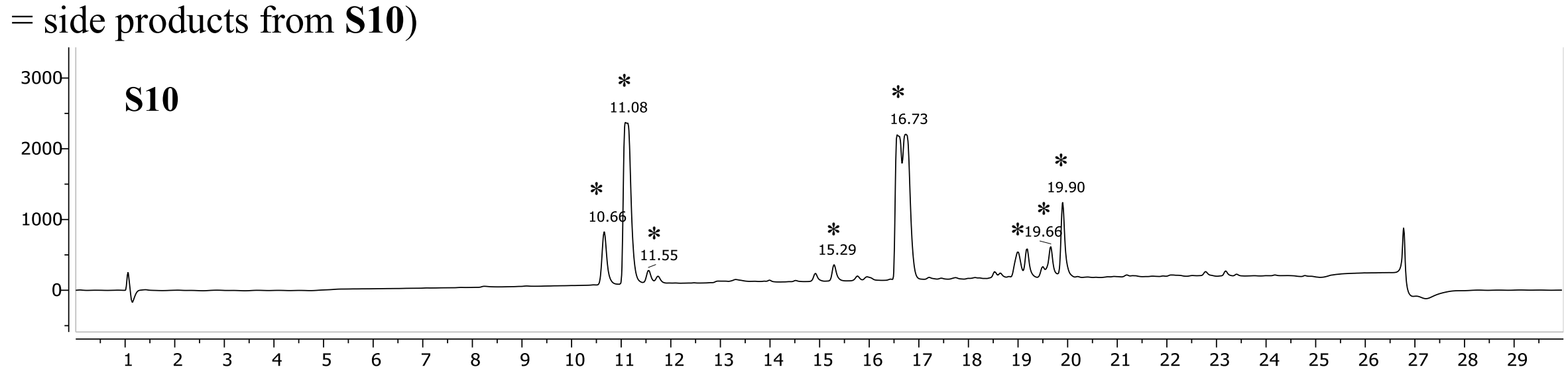

Analytical HPLC trace of the crude reaction mixture

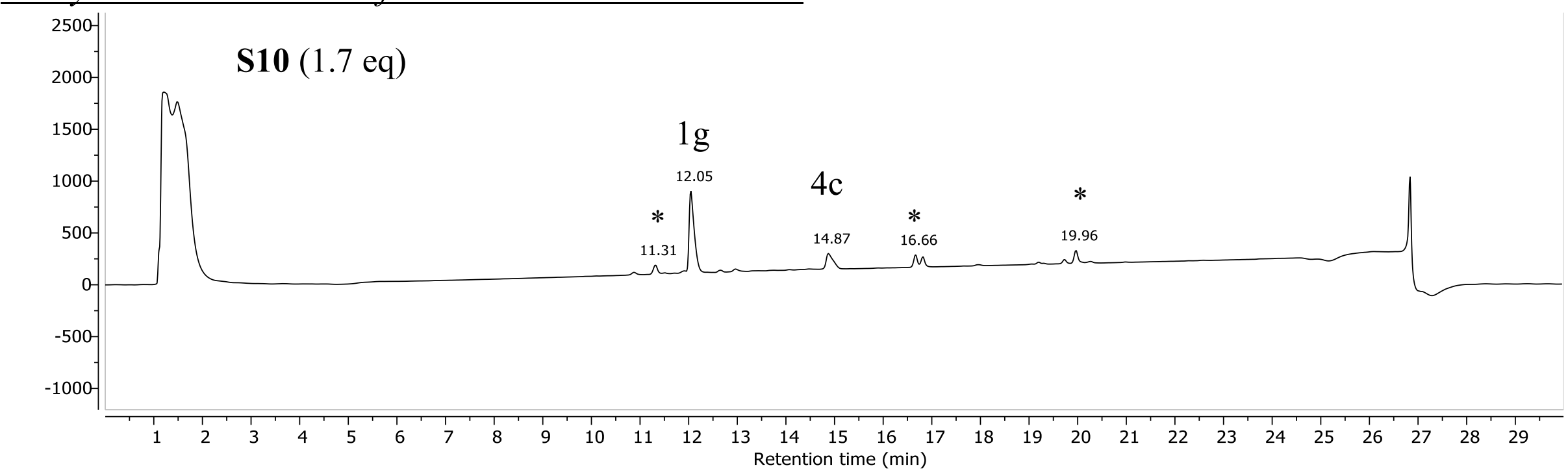

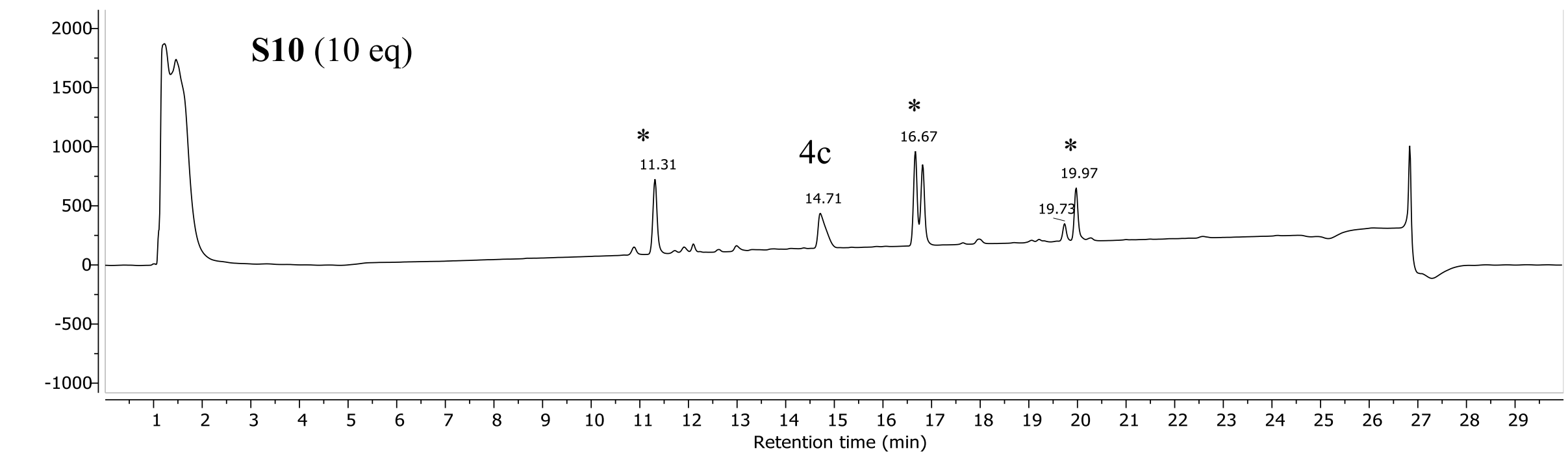

(* = side products from **S10**)

HRMS spectrum of purified peptide Ac-C(P(O)(OAr[1])$_2$)WAFR-CONH$_2$ ***4c***

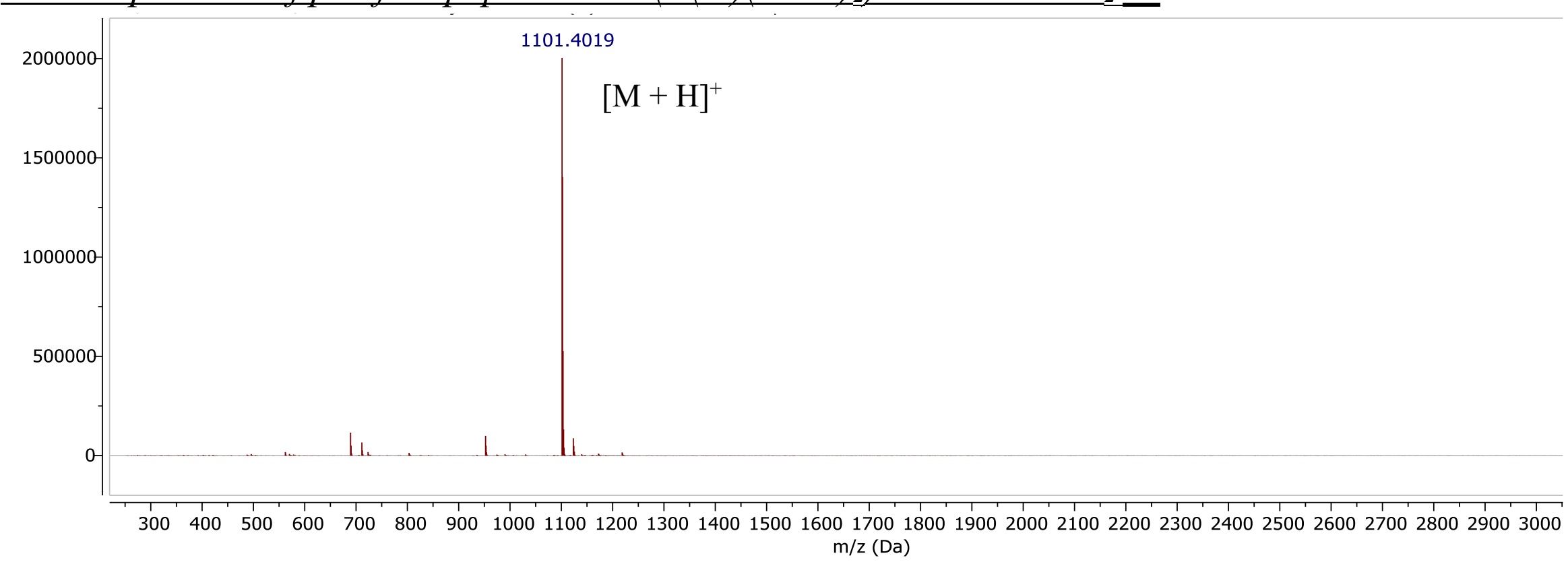

XVIII. Reaction of Aryl-Phosphite **S10** on Fmoc-C(SEt)-OH **1b**.

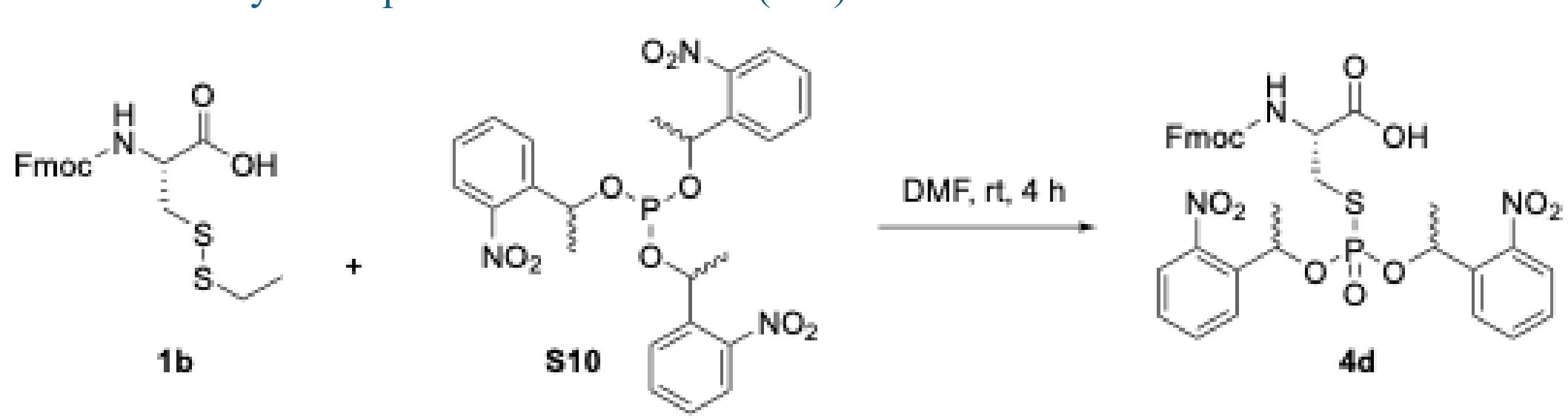

The amino acid N-(((9H-fluoren-9-yl)methoxy)carbonyl)-S-(ethylthio)-L-cysteine **1b** (0.5 mg, 0.001 mmol, 1 eq) and tris(1-(2-nitrophenyl)ethyl) phosphite **S10** (5.2 mg, 0.01 mmol, 10 eq) were added to 1 dram vial and dissolved in 500 µL DMF. 60 µL aliquot was taken after 4 h and diluted with 500 µL of (ACN:H_2O = 1:1). The resulting crude mixture was analyzed via **Analytical HPLC Method** (C18, Water (A)/ACN (B); gradient: 5 to 85% B (0.1% formic acid in ACN) in 20 min, flow rate = 1.0 mL/min, I = 220 nm) and resulted no conversion to product **4d** was observed. Under this condition, the amino-acid **1b** was exhibited retention time of 18.47 min.

*Analytical HPLC trace of tris(1-(2-nitrophenyl)ethyl) phosphite **S10** in DMF*

(* = side products from **S10**)

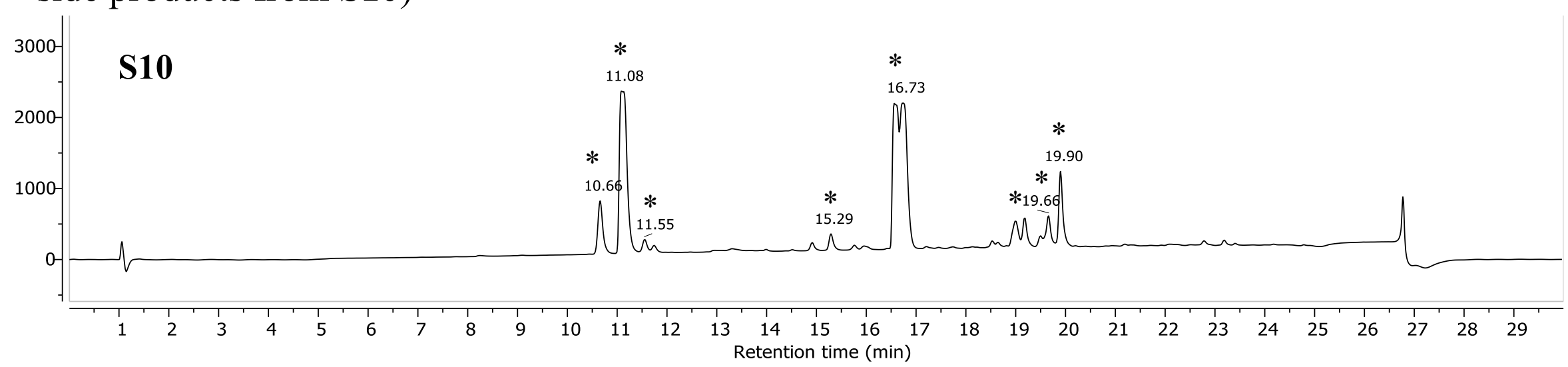

Analytical HPLC trace of the crude reaction mixture

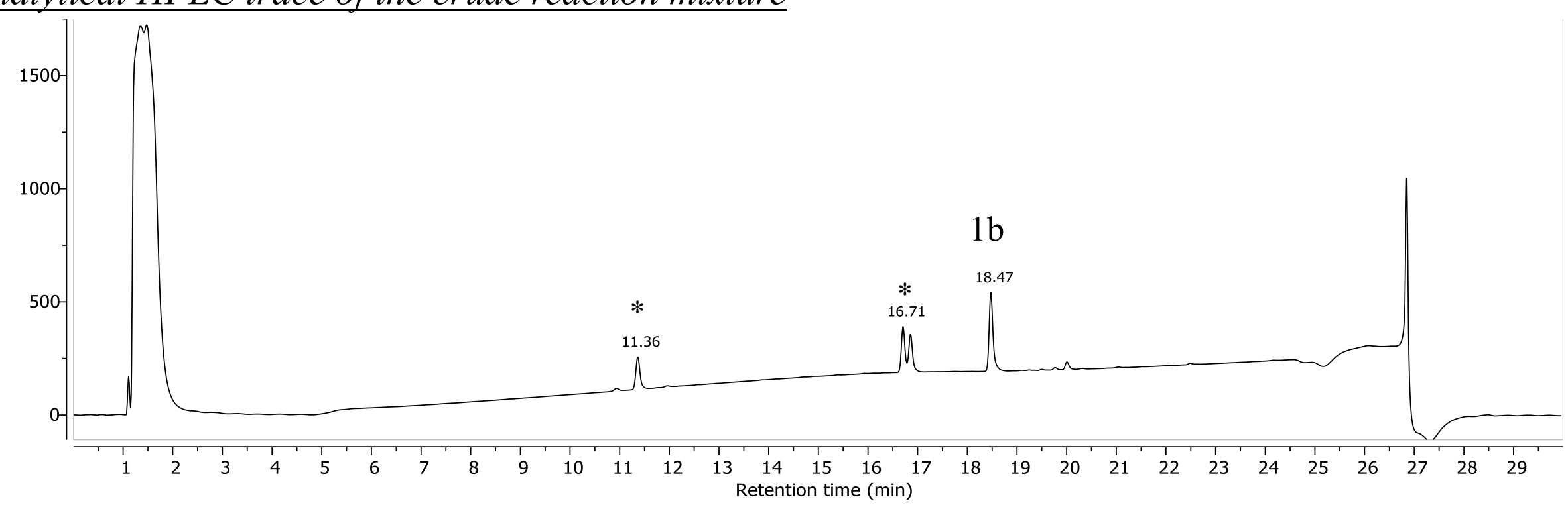

(* = side products from **S10**)

XIX. Reaction of Aryl-Phosphite **S10** on Fmoc-C(S^{t}Bu)-OH **1c**.

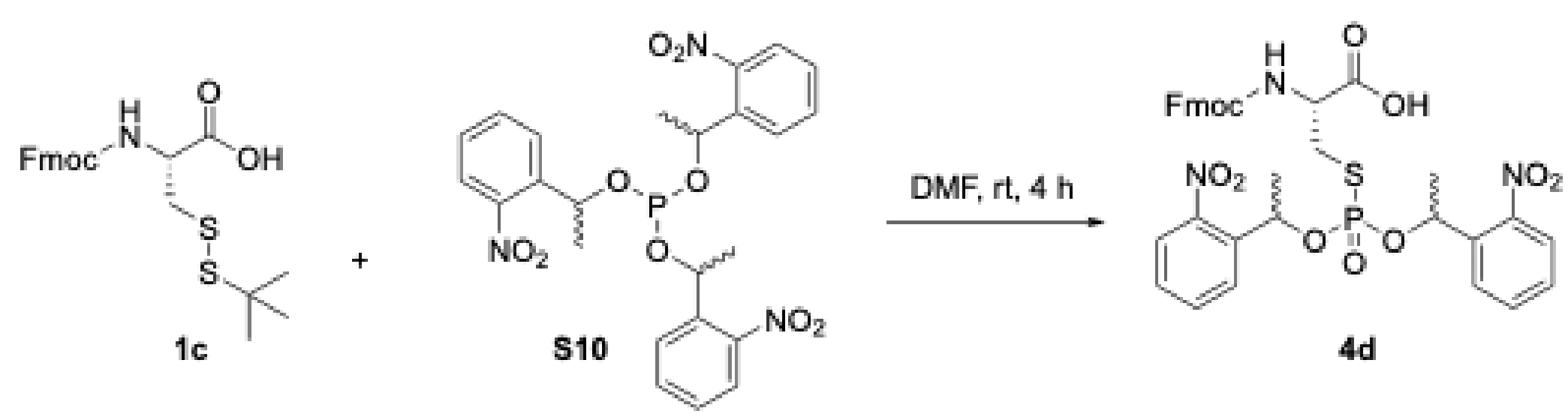

The amino acid N-(((9H-fluoren-9-yl)methoxy)carbonyl)-S-(tert-butylthio)-L-cysteine **1c** (0.5 mg, 0.001 mmol, 1 eq) and tris(1-(2-nitrophenyl)ethyl) phosphite **S10** (5.2 mg, 0.01 mmol, 10 eq) were added to 1 dram vial and dissolved in 500 µL DMF. 60 µL aliquot was taken after 4 h and diluted with 500 µL of (ACN:H_2O = 1:1). The resulting crude mixture was analyzed via **Analytical HPLC Method** (C18, Water (A)/ACN (B); gradient: 5 to 85% B (0.1% formic acid in ACN) in 20 min, flow rate = 1.0 mL/min, I = 220

nm) and resulted no conversion of product **4d** was observed. Under this condition, the amino-acid **1c** was exhibited retention time of 19.81 min.

*Analytical HPLC trace of tris(1-(2-nitrophenyl)ethyl) phosphite **S10** in DMF*

(* = side products from **S10**)

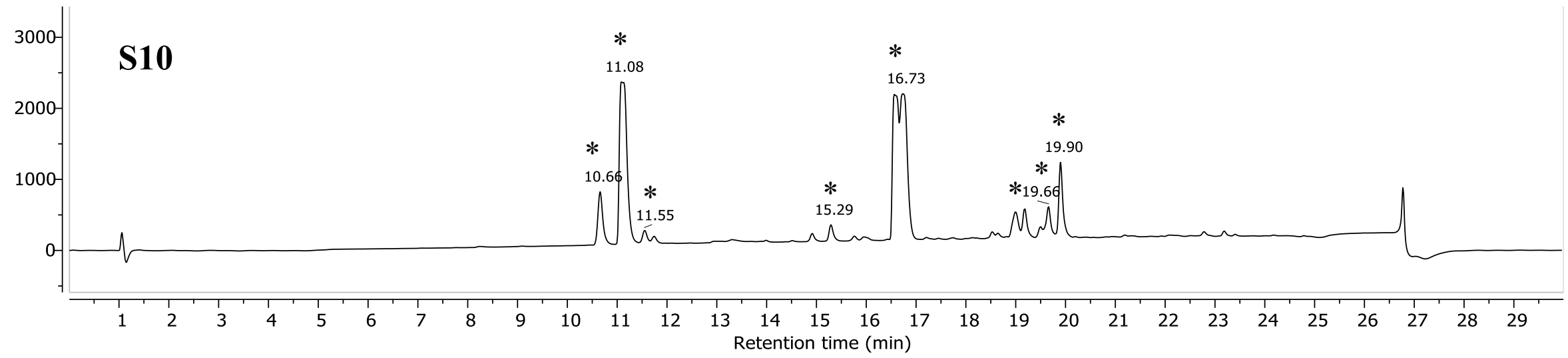

Analytical HPLC trace of the crude reaction mixture

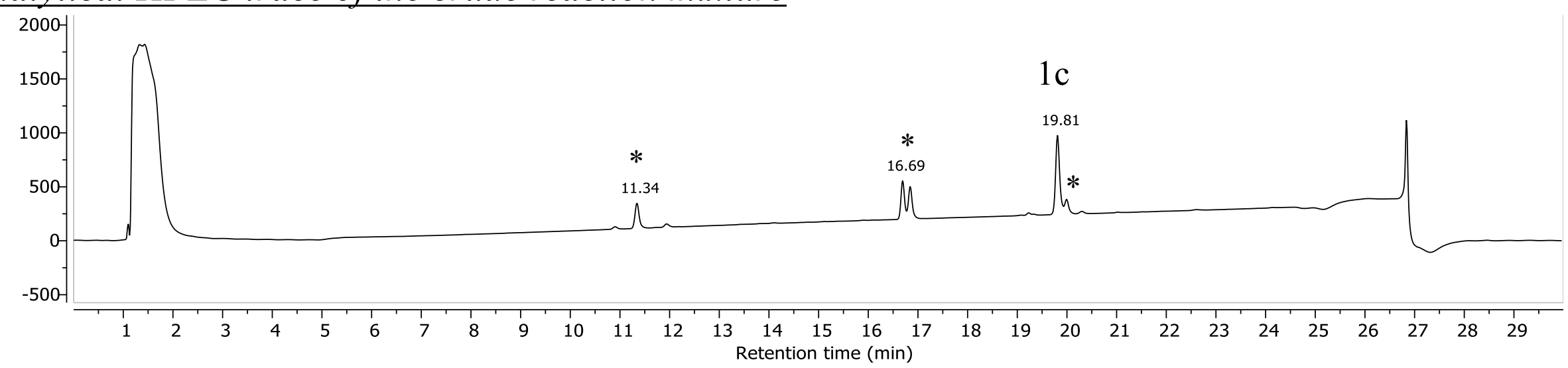

(* = side products from **S10**)

XX.a. Supplementary Figure 10. Phosphite Mediated Orthogonal Cleavage and Modification of Aryl and *tert*-Butyl Containing Disulfides on Fully Unprotected Peptide **1e**.

XX.a.1. Orthogonal Phosphite-Modification of Aryl Disulfide on Peptide **1e**.

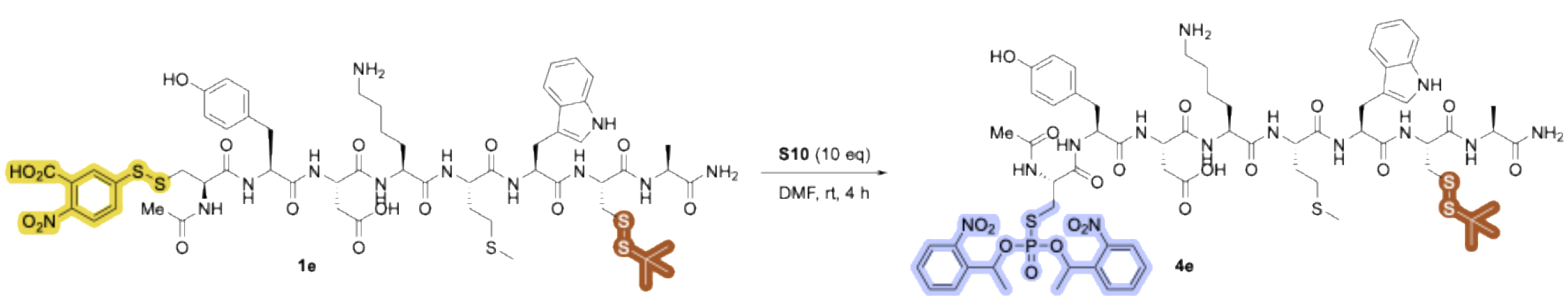

In a 1 dram vial equipped with a magnetic stirrer was added the purified peptide Ac-C(SAr[2])YDKMWC(S^{t}Bu)A-CONH$_2$ **1e** (4 mg, 0.0029 mmol, 1 eq) and it was dissolved in 400 µL DMF while stirring. In a separate 1 dram vial, tris(1-(2-nitrophenyl)ethyl) phosphite **S10** (16 mg, 0.029 mmol, 10 eq) was taken and dissolved in DMF (100 µL) and the solution was added dropwise to the peptide solution and resulting mixture was allowed to stir 4 h at room temperature. After that, the selective phosphite modified product Ac-C(P(O)(OAr[1])$_2$)YDKMWC(S^{t}Bu)A-CONH$_2$ **4e** (pale yellow solid, 4.4 mg, >99% conversion) was determined via analytical HPLC using 0.1% formic acid (FA) in both mobile phases A and B. Following analysis, product was subsequently purified using this same method.

Peptide Ac-C(P(O)(OAr[1])$_2$)YDKMWC(S^{t}Bu)A-CONH$_2$ 4e: **HRMS (ESI)** m/z: $C_{66}H_{89}N_{13}O_{19}PS_4$ $[M+H]^+$ Calcd for 1526.5013, Found 1526.5014. **Analytical HPLC Method** (C18, Water (A)/ACN (B);

gradient: 5 to 85% B (0.1% formic acid in ACN) in 20 min, flow rate = 1.0 mL/min, I = 220 nm)**:** retention time of 15.33 min.

Analytical HPLC trace of tris(1-(2-nitrophenyl)ethyl) phosphite ***S10*** *in DMF*

(* = side products from **S10**)

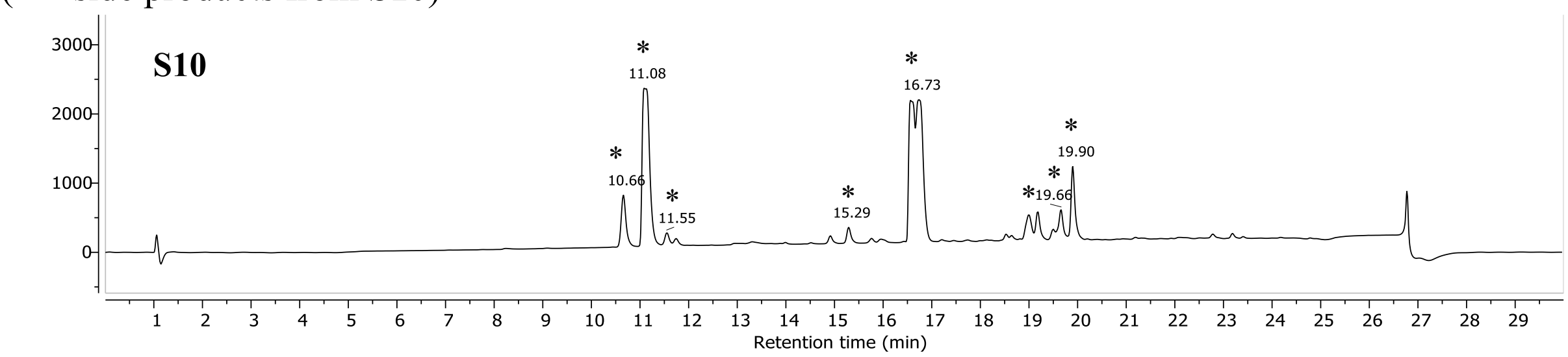

Analytical HPLC trace of the crude reaction mixture converting Ac-C(SAr)YDKMWC(S^{t}Bu)A-CONH$_2$ ***1e*** *to Ac-C(P(O)(OAr^1)$_2$)YDKMWC(S^{t}Bu)A-CONH$_2$* ***4e***

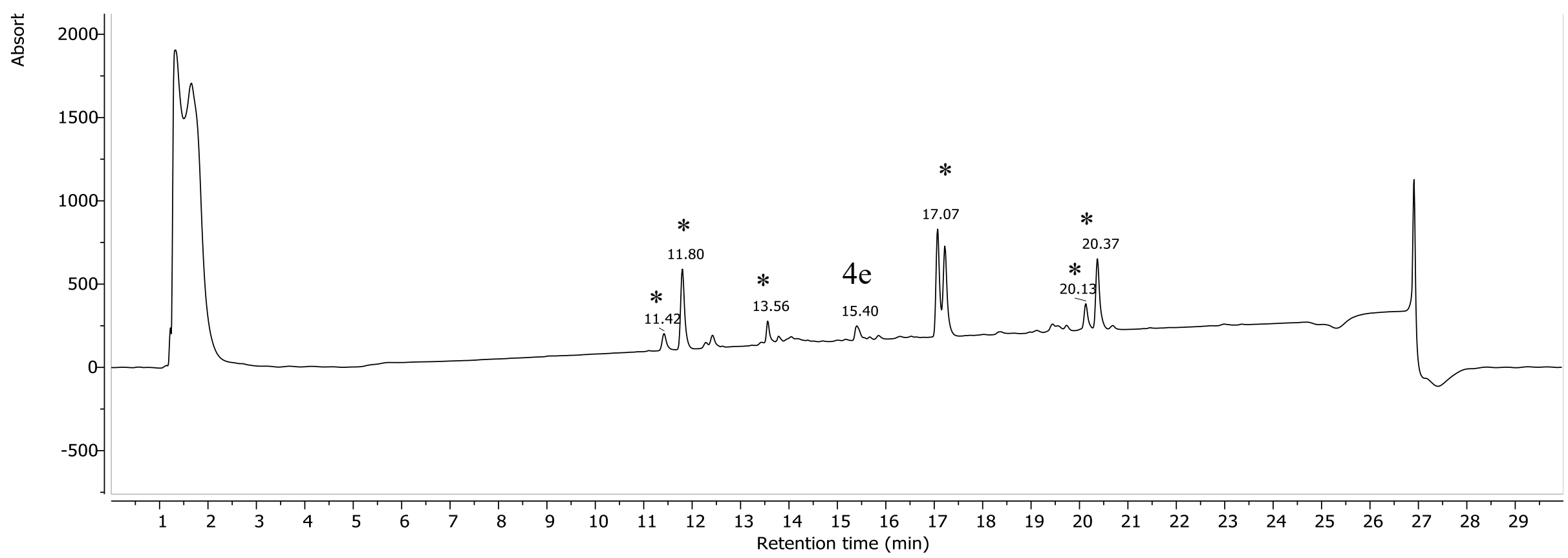

(* = side products from **S10**)

Analytical HPLC trace of purified peptide Ac-C(P(O)(OAr^1)$_2$)YDKMWC(S^{t}Bu)A-CONH$_2$ ***4e***

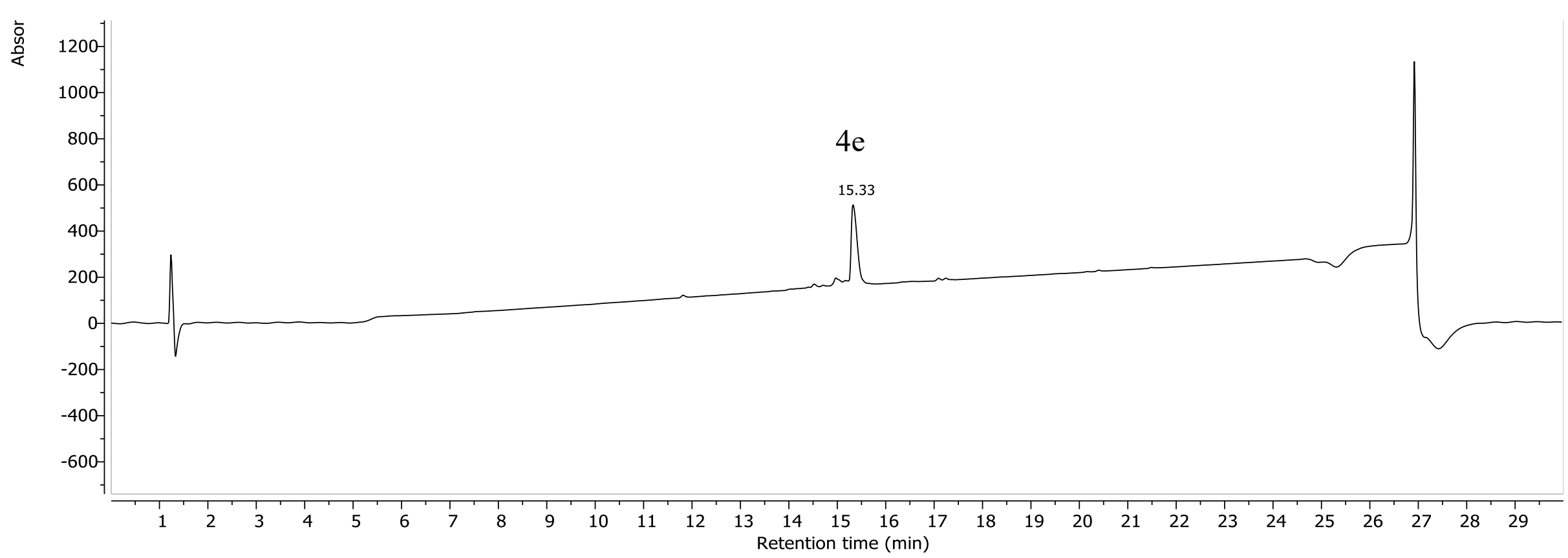

HRMS spectrum of purified peptide Ac-C(P(O)(OAr[1])$_2$)YDKMWC(S[t]Bu)A-CONH$_2$ ***4e***

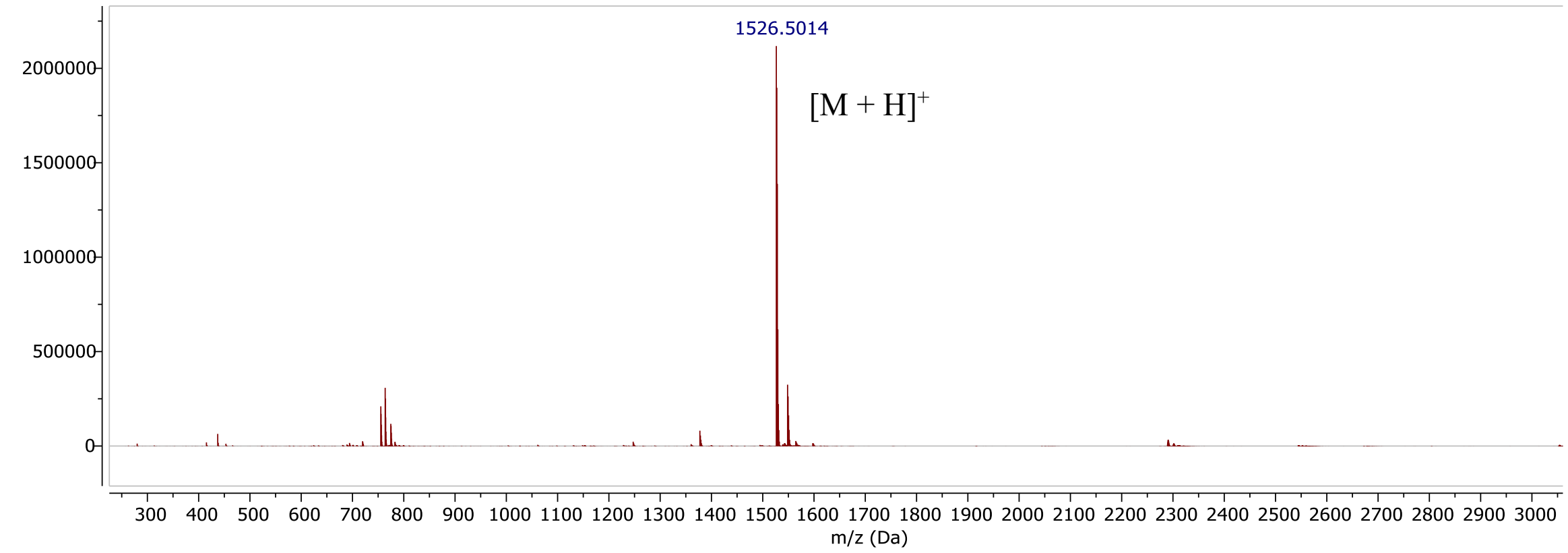

XX.a.2. Orthogonal Cleavage of *tert*-Butyl Containing Disulfide on Compound **4e**.

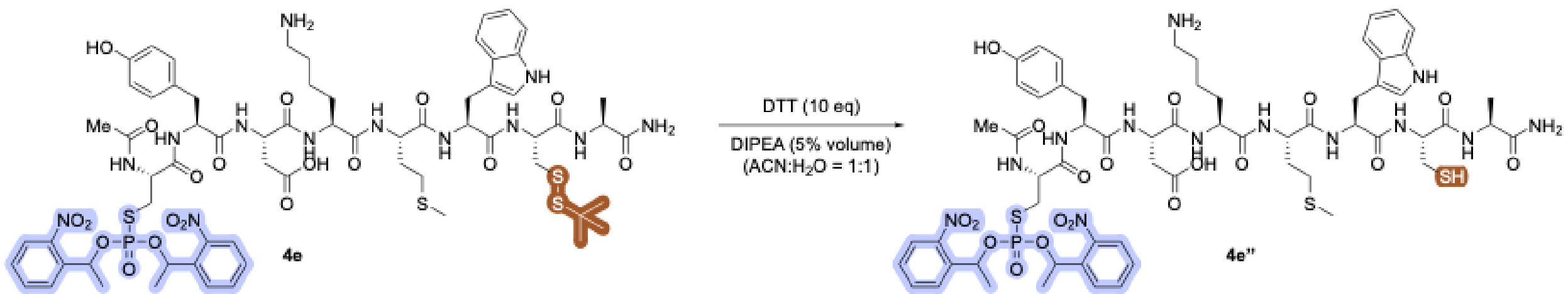

In a 1 dram vial equipped with a magnetic stirrer was added the purified peptide Ac-C(P(O)(OAr[1])$_2$)YDKMWC(S[t]Bu)A-CONH$_2$ **4e** (3 mg, 0.0019 mmol, 1 eq) and dissolved in ACN (500 µL), and H_2O (200 µL) while stirring. In a separate 1 dram vial, dithiothreitol (DTT) (3.6 mg, 0.019 mmol, 10 eq) was taken and dissolved in H_2O (300 µL) and the solution was added dropwise to the peptide solution followed by addition of *N,N*-diisopropylethylamine (DIPEA, 25 µL) at once and resulting mixture was allowed to stir 1 h at room temperature. After that, the selective reduced thiol product **4e''** (pale yellow solid, 2.7 mg, >99% conversion) was determined via analytical HPLC using 0.1% formic acid (FA) in both mobile phases A and B. Following analysis, solvent was evaporated in speedvac and used for the next step without purification.

XX.a.3. Orthogonal Modification of Free Cys on Unprotected Peptide **4e''**.

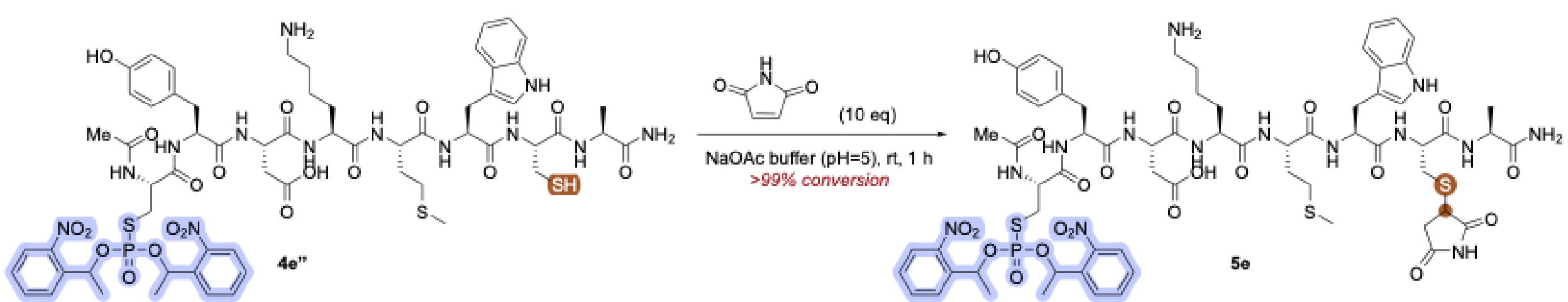

In a 1 dram vial equipped with a magnetic stirrer was added the crude peptide Ac-C(P(O)(OAr[1])$_2$)YDKMWCA-CONH$_2$ **4e''** (3 mg, 0.0020 mmol, 1 eq) and it was dissolved in sodium acetate buffer (NaOAc buffer pH=5) (300 µL) while stirring. In a separate 1 dram vial, maleimide (2 mg, 0.02 mmol, 10 eq) was taken and dissolved in NaOAc buffer (200 µL) and the solution was added dropwise to the peptide solution and resulting mixture was allowed to stir 1 h at room temperature. After that, the selective maleimide adduct peptide **5e** (pale yellow solid, 3 mg, >99% conversion) was determined via

analytical HPLC using 0.1% formic acid (FA) in both mobile phases A and B. Following analysis, product was subsequently purified using this same method.

Peptide Ac-C(P(O)(OAr[1])$_2$)YDKMWC(maleimide)A-CONH$_2$ 5e: **HRMS (ESI)** m/z: $C_{66}H_{84}N_{14}O_{21}PS_3$ $[M+H]^+$ Calcd for 1535.4830, Found 1535.4800. **Analytical HPLC Method** (C18, Water (A)/ACN (B); gradient: 5 to 85% B (0.1% formic acid in ACN) in 20 min, flow rate = 1.0 mL/min, I = 220 nm)**:** retention time of 14.27 min.

*Analytical HPLC trace of the crude reaction mixture converting Ac-C(P(O)(OAr^1)$_2$)YDKMWCA-CONH$_2$ **4e''** to Ac-C(P(O)(OAr^1)$_2$)YDKMWC(maleimide)A-CONH$_2$ **5e***

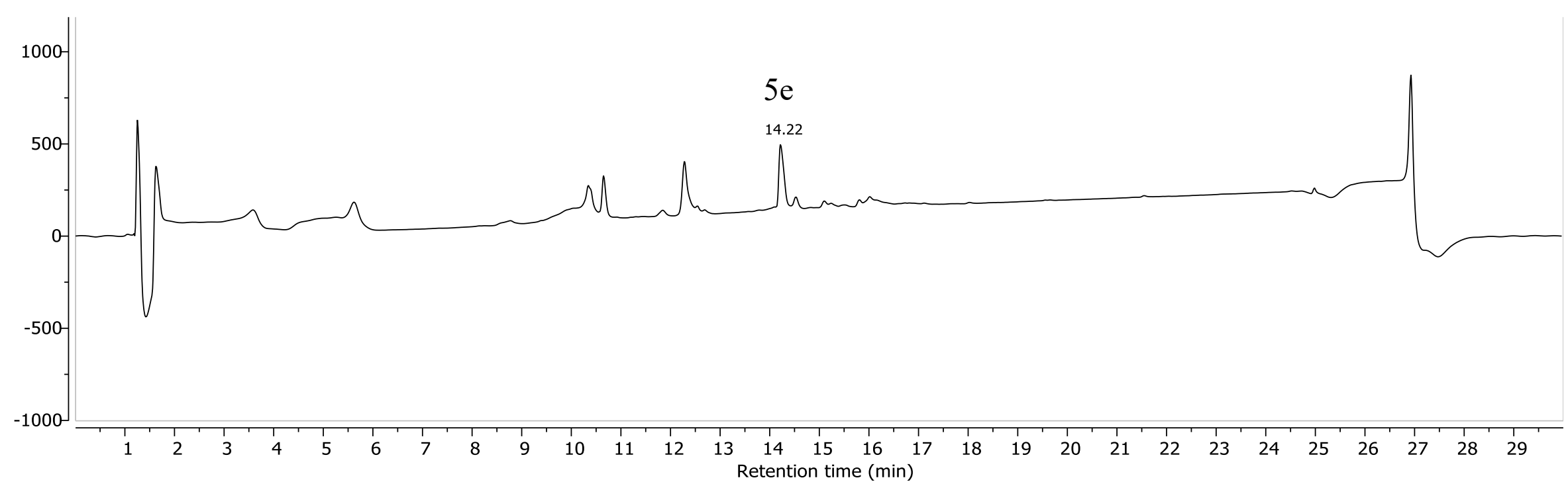

*Analytical HPLC trace of purified peptide Ac-C(P(O)(OAr^1)$_2$)YDKMWC(maleimide)A-CONH$_2$ **5e***

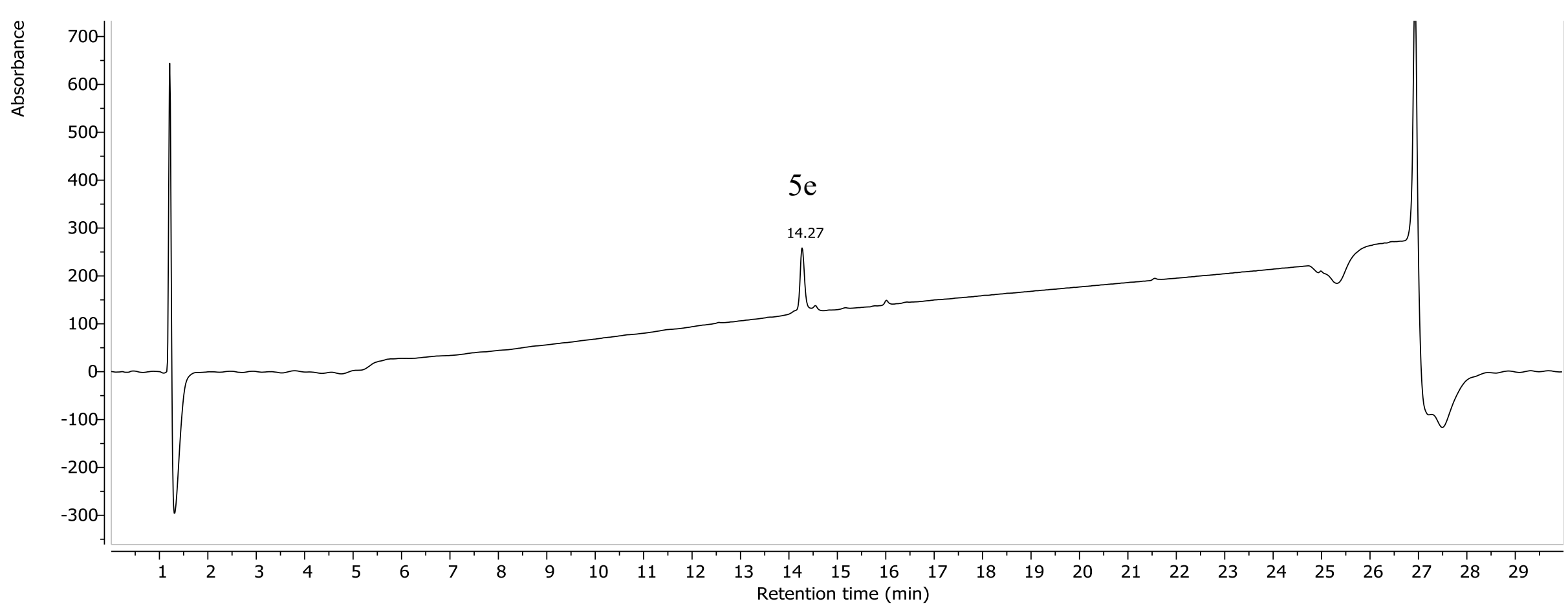

HRMS spectrum of purified peptide Ac-C(P(O)(OAr[1])$_2$)YDKMWC(maleimide)A-CONH$_2$ ***5e***

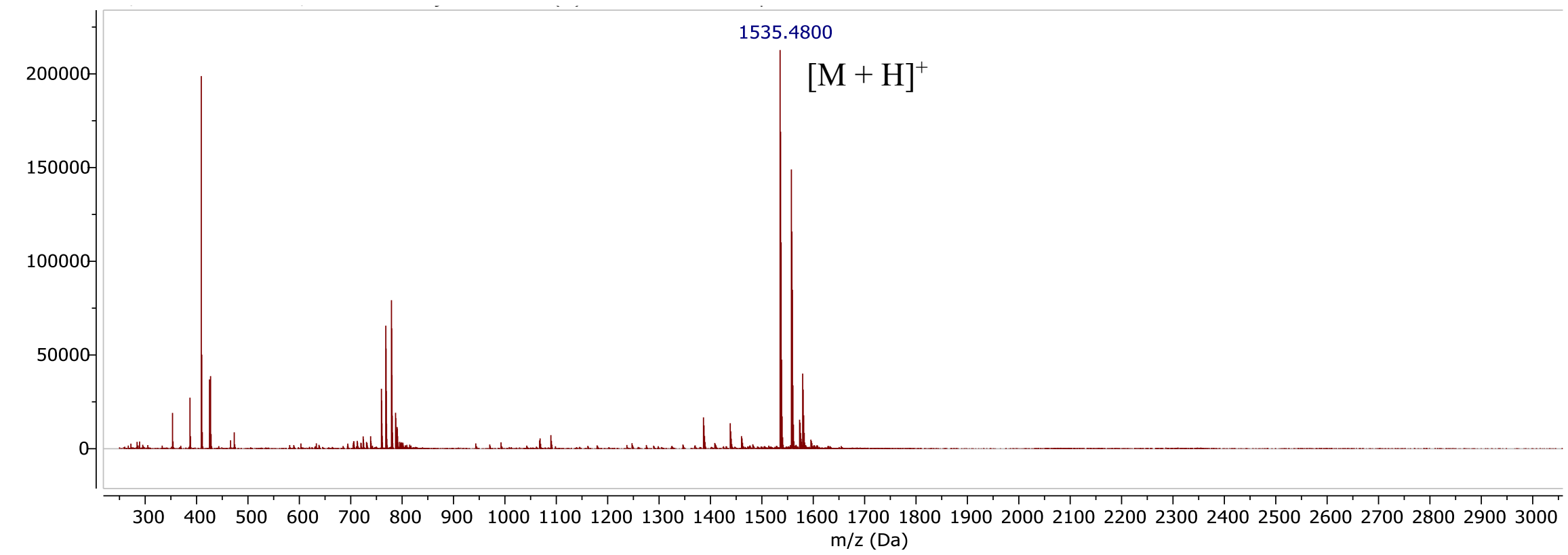

XX.b. Supplementary Figure 11. Phosphite Mediated Orthogonal Cleavage and Modification of Aryl and Ethyl Containing Disulfides on Fully Unprotected Peptide mixture of **1g** and **1h**.

XX.b.1. Orthogonal Phosphite-Modification of Aryl Disulfide on Peptide **1g** in the Mixture of Peptides **1g** and **1h.**

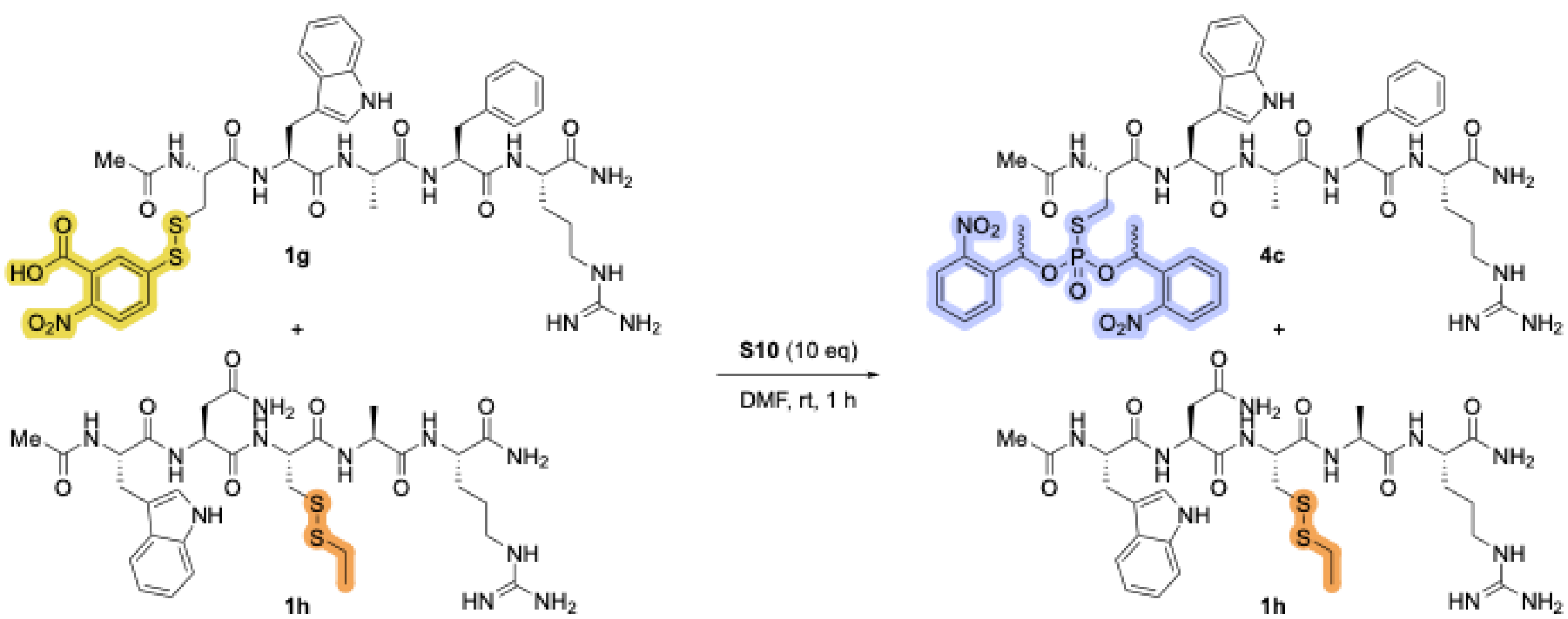

In a 1 dram vial equipped with a magnetic stirrer were added the purified peptide Ac-C(SAr^2)WAFR-CONH$_2$ **1g** (1 mg, 0.0011 mmol, 1 eq) and Ac-WNC(SEt)AR-CONH$_2$ **1h** (1 mg, 0.0013 mmol, 1 eq) and dissolved in 400 µL DMF while stirring. In a separate 1 dram vial, tris(1-(2-nitrophenyl)ethyl) phosphite **S10** (16 mg, 0.029 mmol, 10 eq) was taken and dissolved in DMF (100 µL) and the solution was added dropwise to the peptide solution and resulting mixture was allowed to stir 1 h at room temperature. After that, the selective phosphite modified product Ac-C(P(O)(OAr^1)$_2$)WAFR-CONH$_2$ **4c** (pale yellow solid, 2.1 mg, >99% conversion) was determined via analytical HPLC using 0.1% formic acid (FA) in both mobile phases A and B. Following analysis, product was subsequently purified using this same method.

Peptide Ac-C(P(O)(OAr^1)$_2$)WAFR-CONH$_2$ 4c: **HRMS (ESI)** m/z: $C_{50}H_{62}N_{12}O_{13}PS$ [M+H]$^+$ Calcd for 1101.4012, Found 1101.4019. **Peptide Ac-WNC(SEt)AR-CONH$_2$ 1h: HRMS (ESI)** m/z: $C_{31}H_{48}N_{11}O_7S_2$ [M+H]$^+$ Calcd for 750.3174, Found 750.3156.

Analytical HPLC Method (C18, Water (A)/ACN (B); gradient: 5 to 85% B (0.1% formic acid in ACN) in 20 min, flow rate = 1.0 mL/min, I = 220 nm)**:** retention time of **1h** and **4c** are 10.17 and 15.01 min respectively.

*Analytical HPLC trace of tris(1-(2-nitrophenyl)ethyl) phosphite **S10** in DMF*

(* = side products from **S10**)

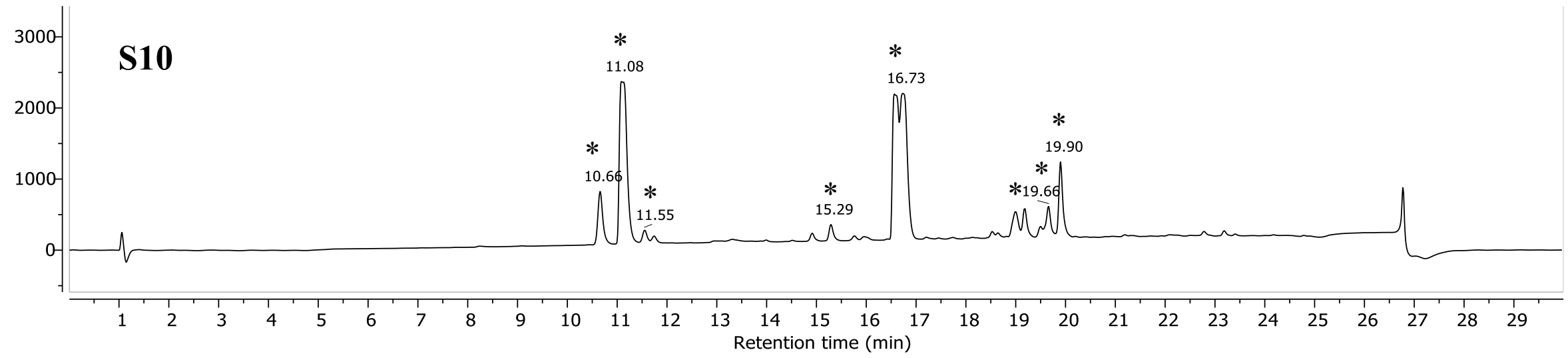

*Analytical HPLC trace of the crude reaction mixture containing Ac-WNC(SEt)AR-$CONH_2$ **1h** and Ac-C(P(O)(OAr[1])$_2$)WAFR-$CONH_2$ **4c***

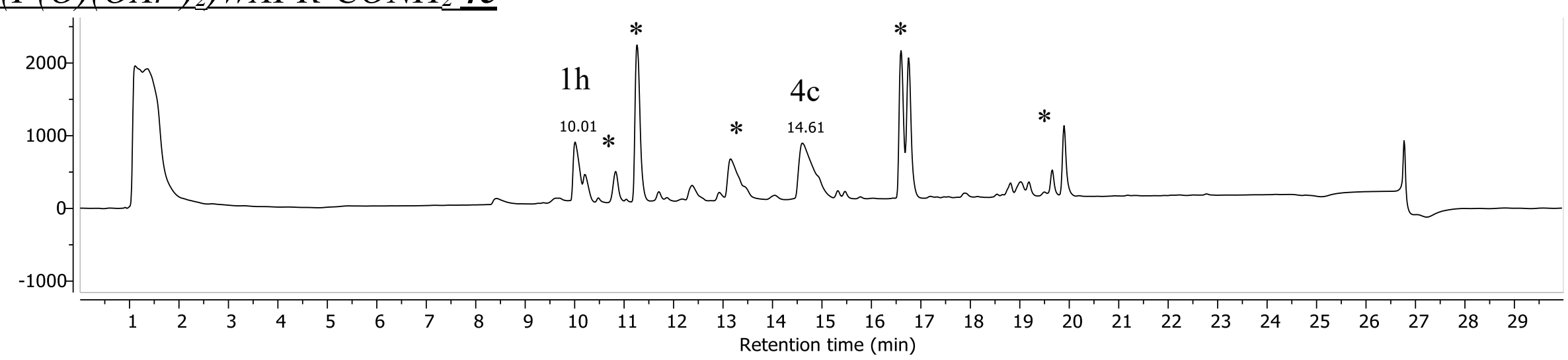

(* = side products from **S10**)

*Analytical HPLC trace of purified peptide mixture Ac-WNC(SEt)AR-$CONH_2$ **1h** and Ac-C(P(O)(OAr[1])$_2$)WAFR-$CONH_2$ **4c***

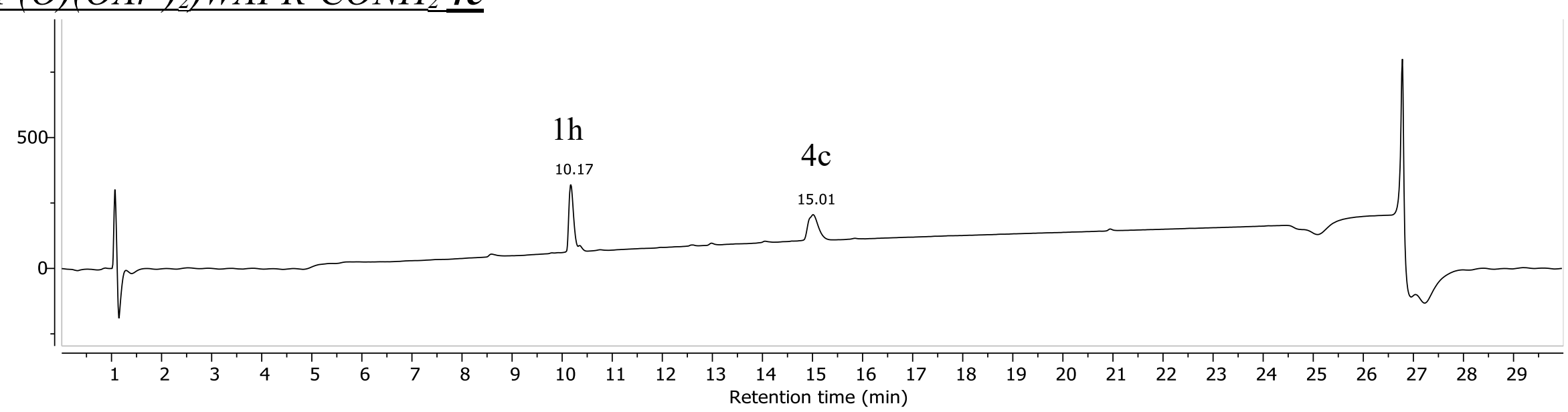

*HRMS spectrum of purified peptide peptide mixture Ac-WNC(SEt)AR-CONH$_2$ **1h** and Ac-C(P(O)(OAr[1])$_2$)WAFR-CONH$_2$ **4c***

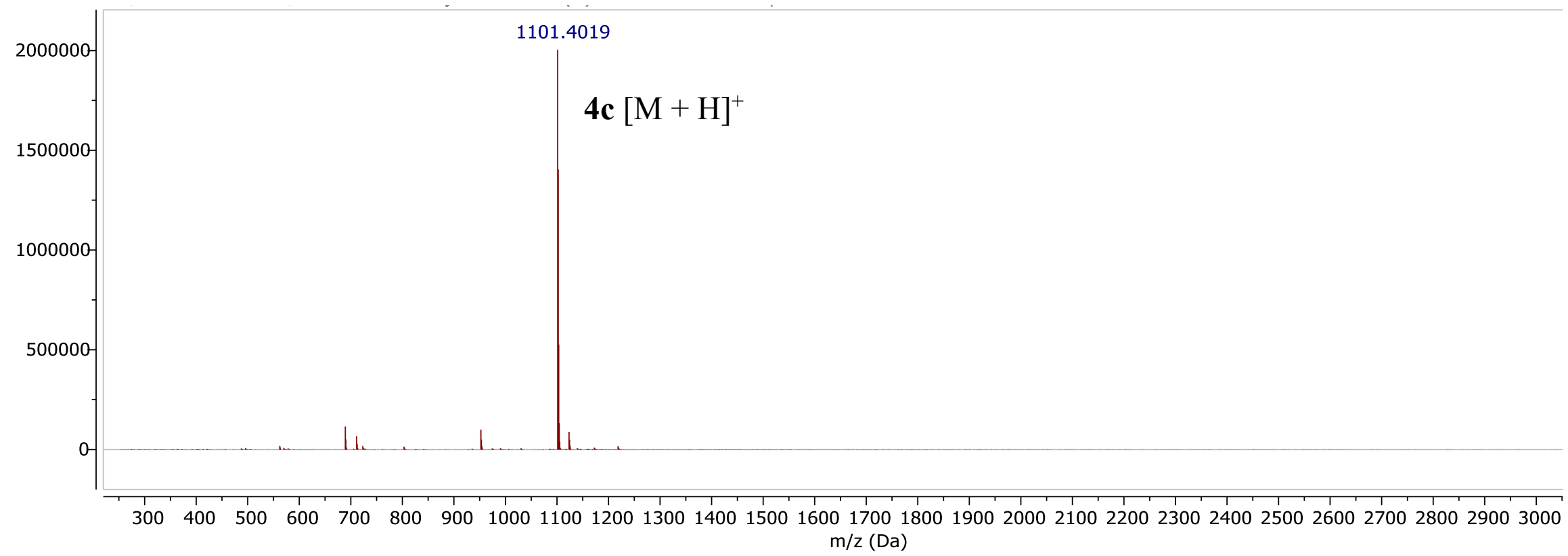

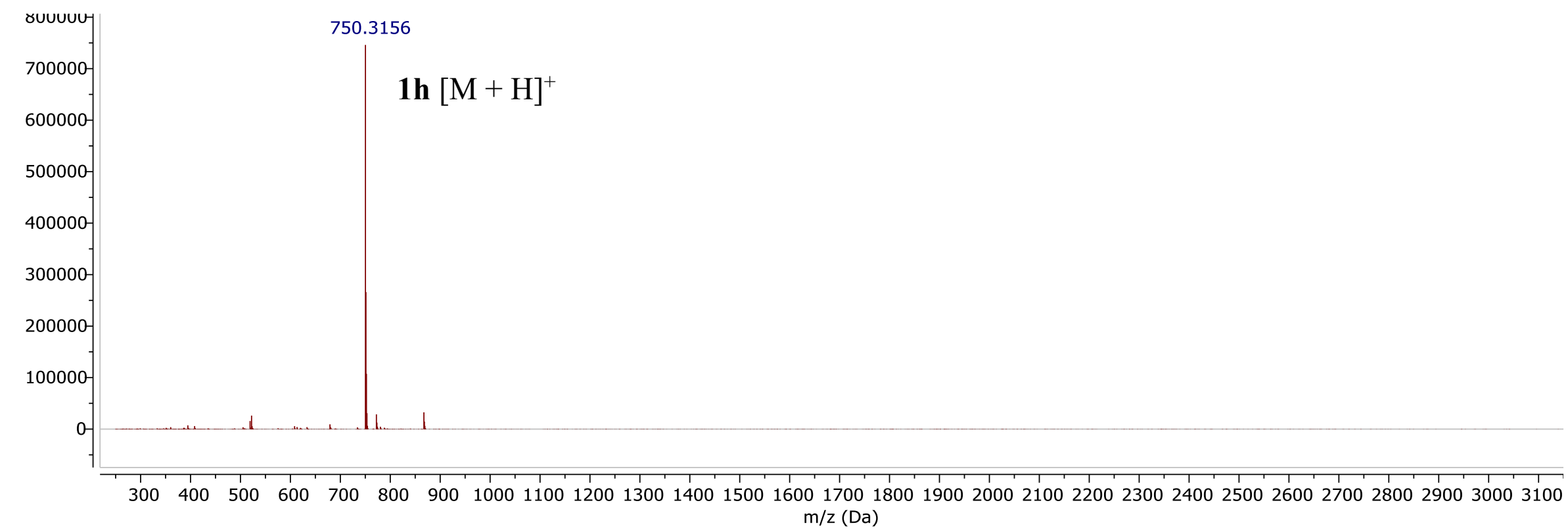

XX.b.2. Orthogonal Cleavage of Ethyl Containing Disulfide on Compound **1h** in the Mixture of Peptides **4c** and **1h**.

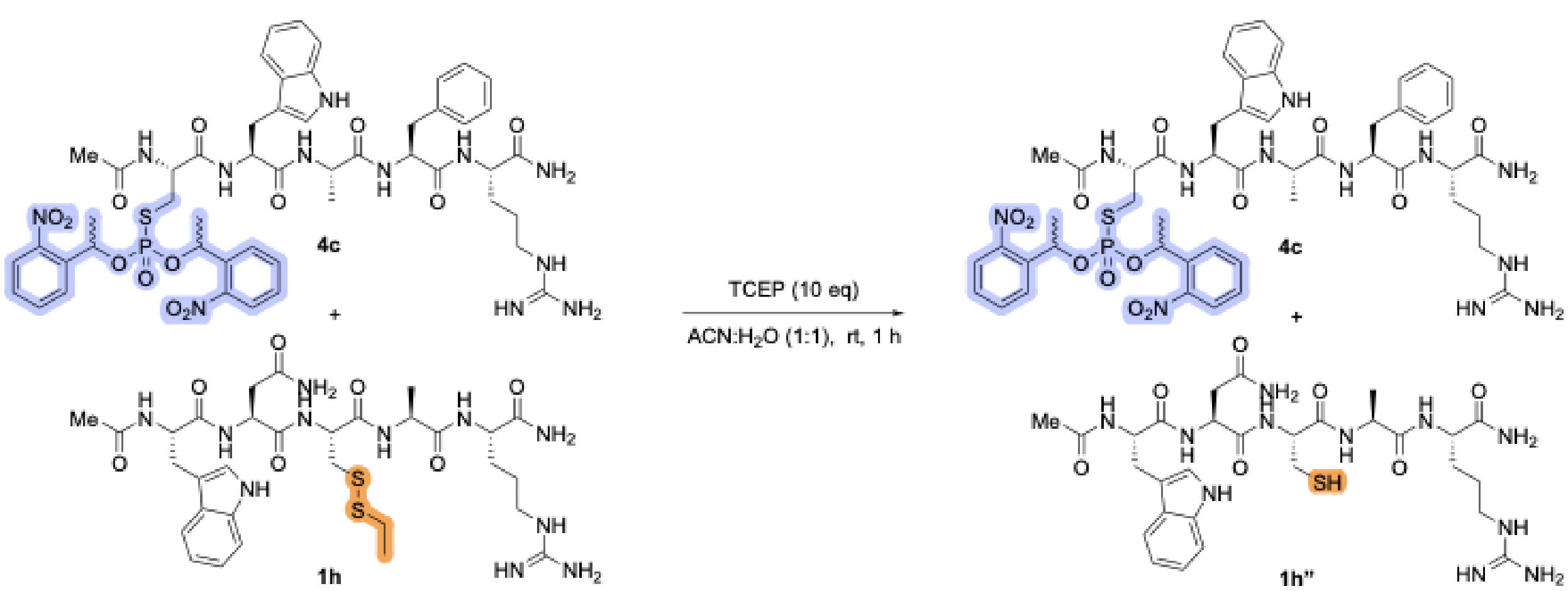

In a 1 dram vial equipped with a magnetic stirrer were added the purified peptide Ac-C(P(O)(OAr[1])$_2$)WAFR-CONH$_2$ **4c** (1 mg, 0.0011 mmol, 1 eq) and Ac-WNC(SEt)AR-CONH$_2$ **1h** (1 mg, 0.0013 mmol, 1 eq) and dissolved in ACN (500 µL), and H_2O (200 µL) while stirring. In a separate 1 dram vial, tris(2-carboxyethyl)phosphine hydrochloride (TCEP) (3.4 mg, 0.013 mmol, 10 eq) was taken and dissolved in H_2O (300 µL) and the solution was added dropwise to the peptide solution and resulting mixture was allowed to stir 1 h at room temperature. After that, the selective reduced thiol product **1h''**

(pale yellow solid, 2 mg, >99% conversion) was determined via analytical HPLC using 0.1% formic acid (FA) in both mobile phases A and B. Following analysis, product was subsequently purified using this same method.

Peptide Ac-WNCAR-CONH$_2$ **1h''**: **HRMS (ESI)** m/z: $C_{29}H_{44}N_{11}O_7S$ [M+H]$^+$ Calcd for 690.3140, Found 690.3140. **Peptide Ac-C(P(O)(OAr^1)$_2$)WAFR-CONH$_2$ 4c**: **HRMS (ESI)** m/z: $C_{50}H_{62}N_{12}O_{13}PS$ [M+H]$^+$ Calcd for 1101.4012, Found 1101.4019.

Analytical HPLC Method (C18, Water (A)/ACN (B); gradient: 5 to 85% B (0.1% formic acid in ACN) in 20 min, flow rate = 1.0 mL/min, I = 220 nm)**:** retention time of **1h''** and **4c** are 8.42 and 14.96 min respectively.

*Analytical HPLC trace of the crude reaction mixture containing peptides Ac-C(P(O)(OAr^1)$_2$)WAFR-CONH$_2$ **4c** and Ac-WNCAR-CONH$_2$ **1h''***

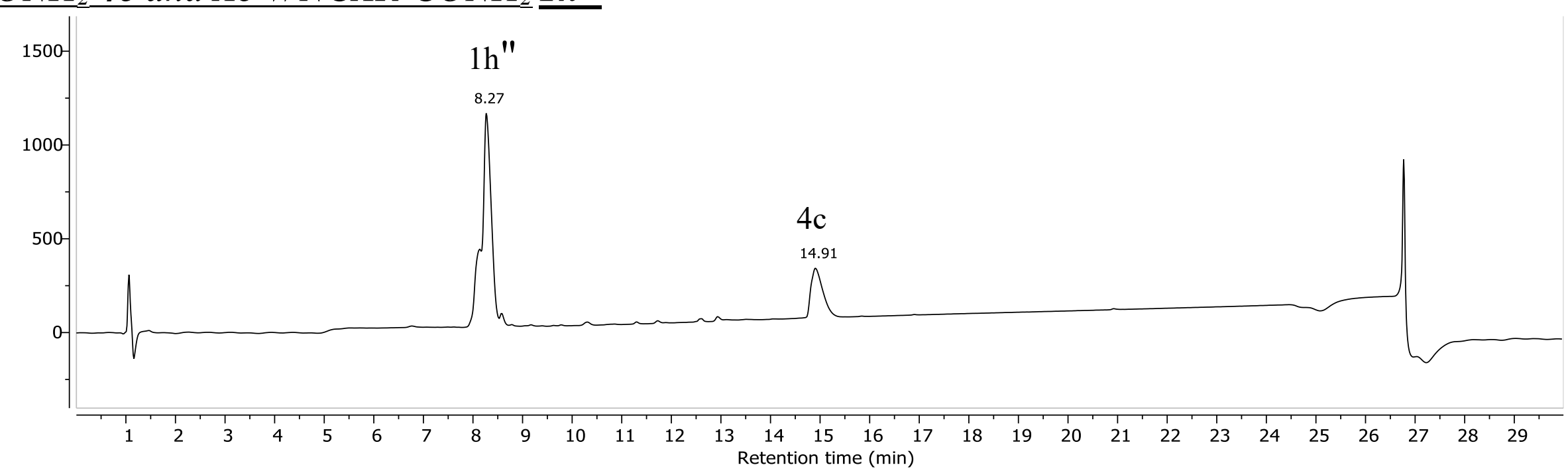

*Analytical HPLC trace of purified peptides Ac-C(P(O)(OAr[1])$_2$)WAFR-CONH$_2$ **4c** and Ac-WNCAR-CONH$_2$ **1h''***

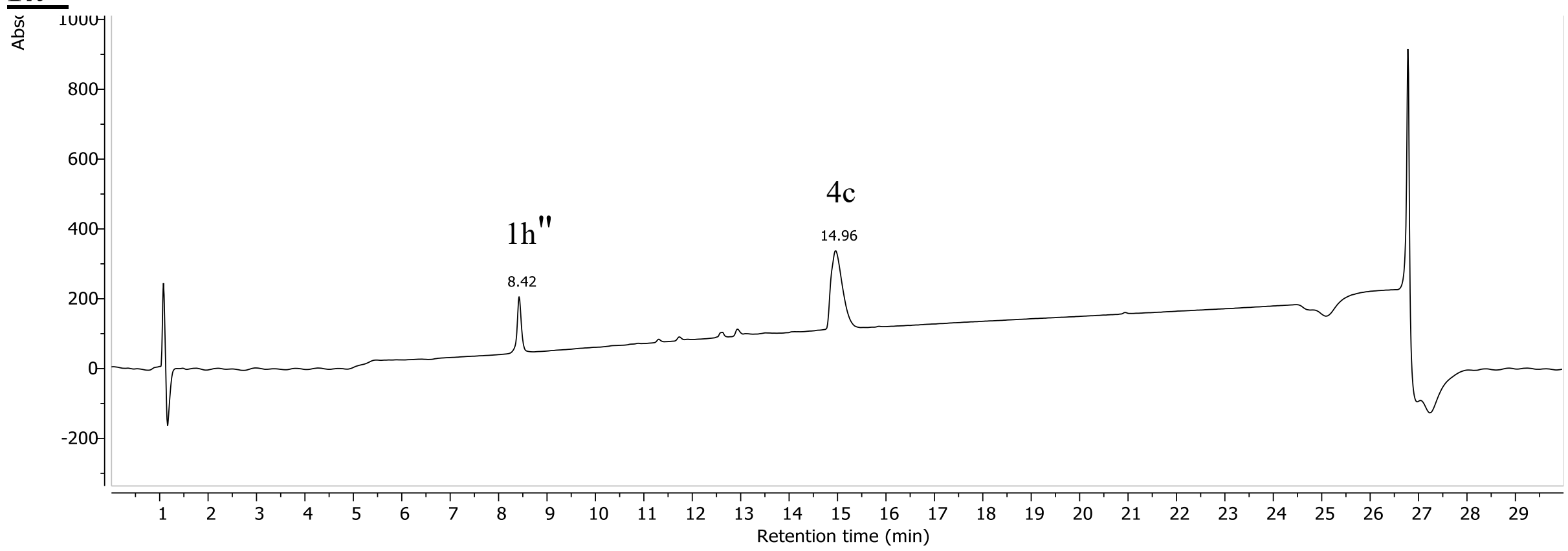

*HRMS spectrum of purified peptides Ac-C(P(O)(OAr[1])$_2$)WAFR-CONH$_2$ **4c** and Ac-WNCAR-CONH$_2$ **1h''***

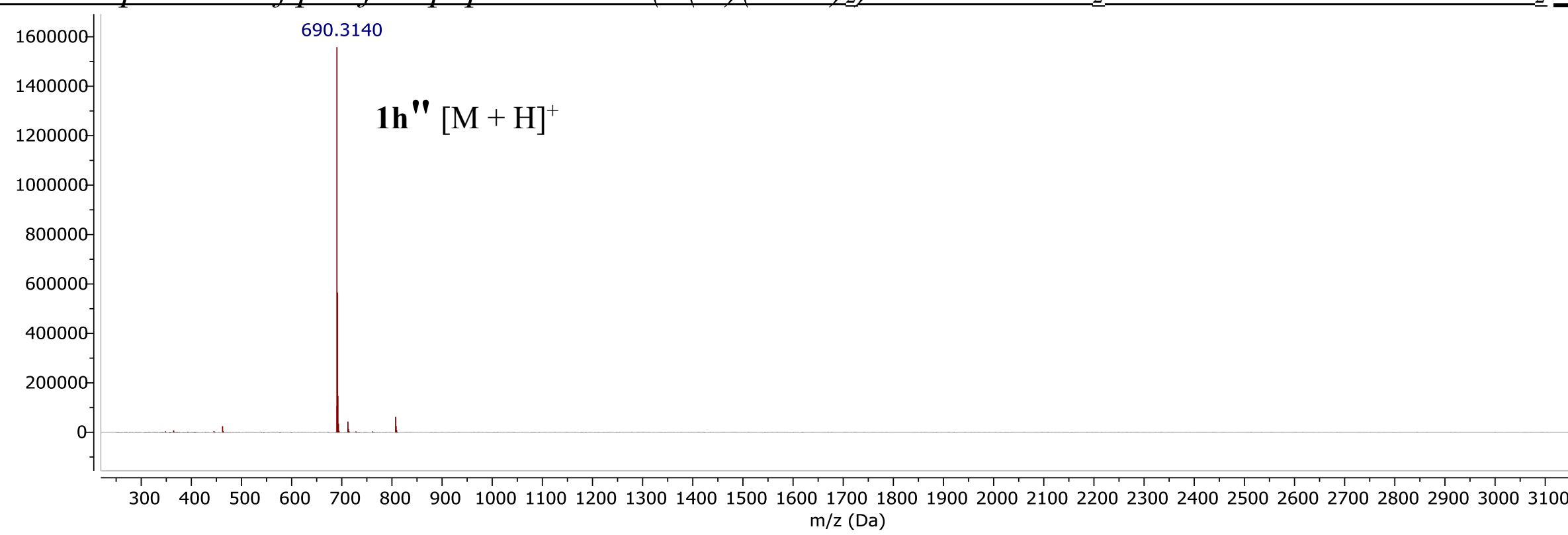

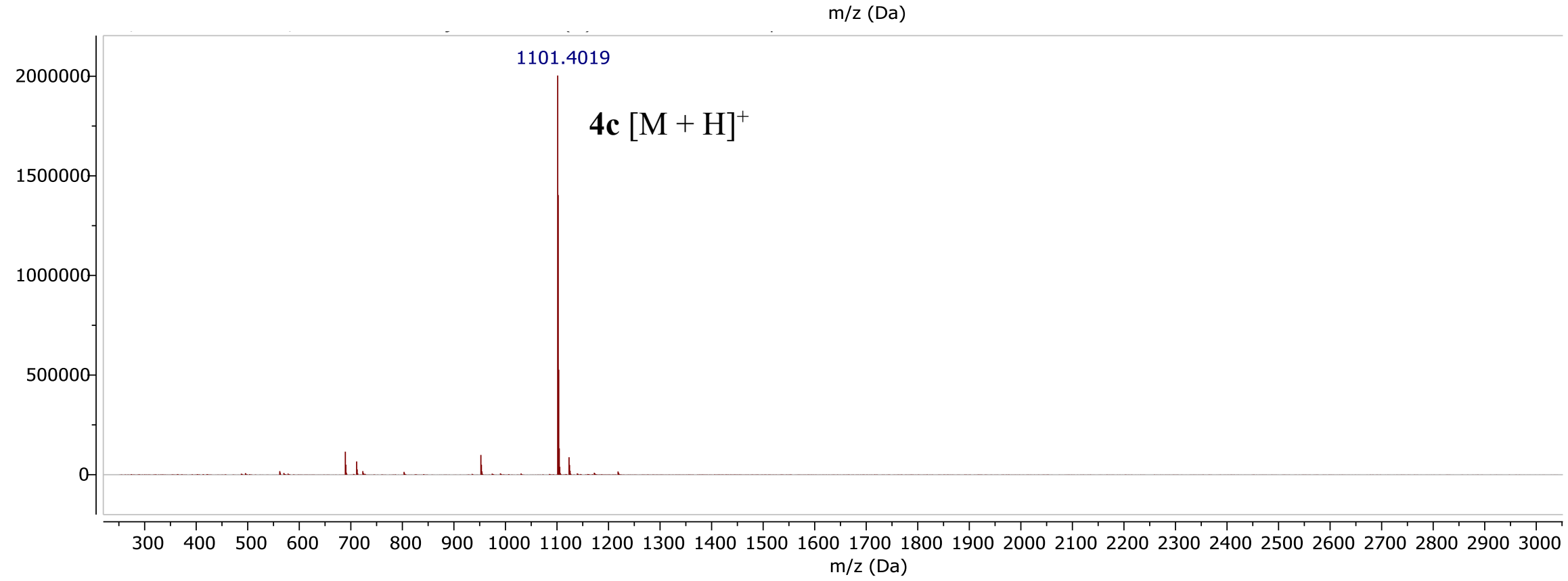

XX.b.3. Orthogonal Modification of Free Cys on **1h"** in the Mixture Peptides of **4c** and **1h"**.

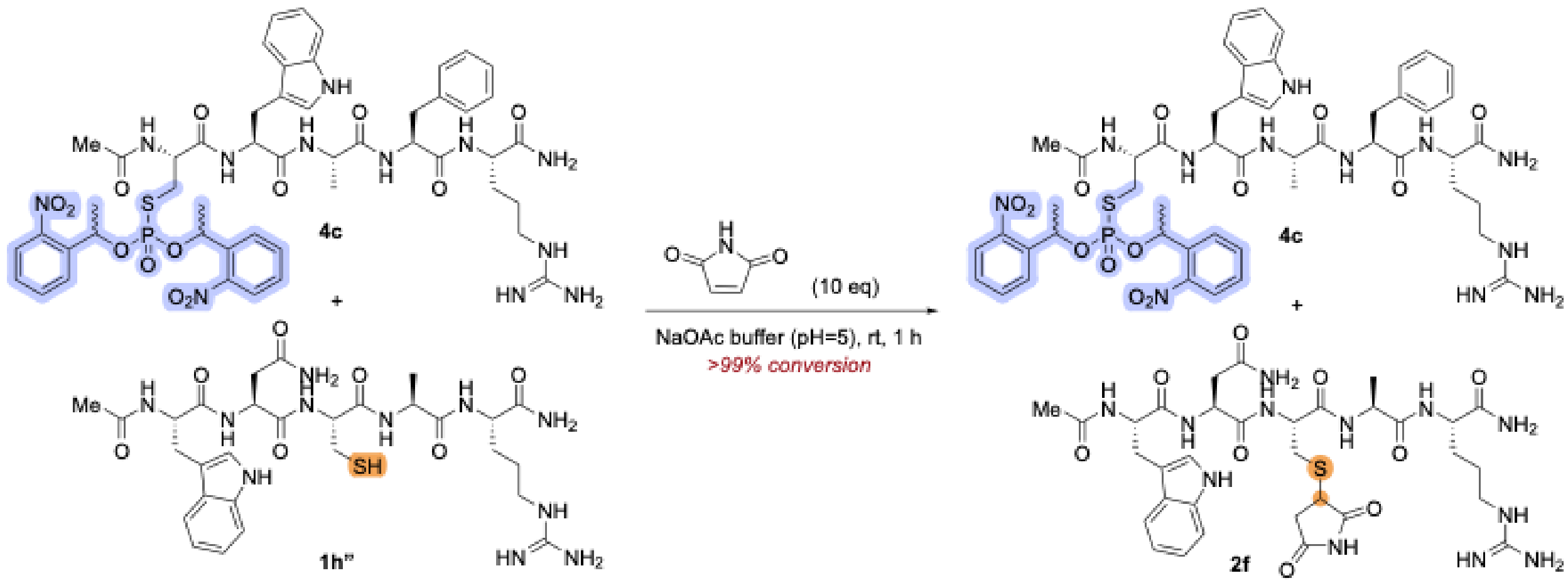

In a 1 dram vial equipped with a magnetic stirrer were added the purified peptide Ac-C(P(O)(OAr[1])2)WAFR-CONH2 **4c** (1 mg, 0.0011 mmol, 1 eq) and Ac-WNCAR-CONH2 **1h"** (1 mg, 0.0013 mmol, 1 eq) and dissolved in sodium acetate buffer (NaOAc buffer pH = 5) (300 μL) while stirring. In a separate 1 dram vial, maleimide (2 mg, 0.02 mmol, 10 eq) was taken and dissolved in NaOAc buffer (200 μL) and the solution was added dropwise to the peptide solution and resulting mixture was allowed to stir 1 h at room temperature. After that, the selective maleimide adduct peptide **2f** (pale yellow solid, 2.1 mg, >99% conversion) was determined via analytical HPLC using 0.1% formic acid (FA) in both mobile phases A and B. Following analysis, product was subsequently purified using this same method.

Peptide Ac-WNC(maleimide)AR-CONH2 2f: **HRMS (ESI)** m/z: $C_{33}H_{47}N_{12}O_9S$ $[M+H]^+$ Calcd for 787.3304, Found 787.3307. **Peptide Ac-C(P(O)(OAr[1])2)WAFR-CONH2 4c**: **HRMS (ESI)** m/z: $C_{50}H_{62}N_{12}O_{13}PS$ $[M+H]^+$ Calcd for 1101.4012, Found 1101.4019.

Analytical HPLC Method (C18, Water (A)/ACN (B); gradient: 5 to 85% B (0.1% formic acid in ACN) in 20 min, flow rate = 1.0 mL/min, I = 220 nm)**:** retention time of **2f** and **4c** 8.26 and 14.78 min respectively.

*Analytical HPLC trace of the crude reaction mixture containing Ac-C(P(O)(OAr[1])2)WAFR-CONH2 **4c** and Ac-WNC(maleimide)AR-CONH2 **2f***

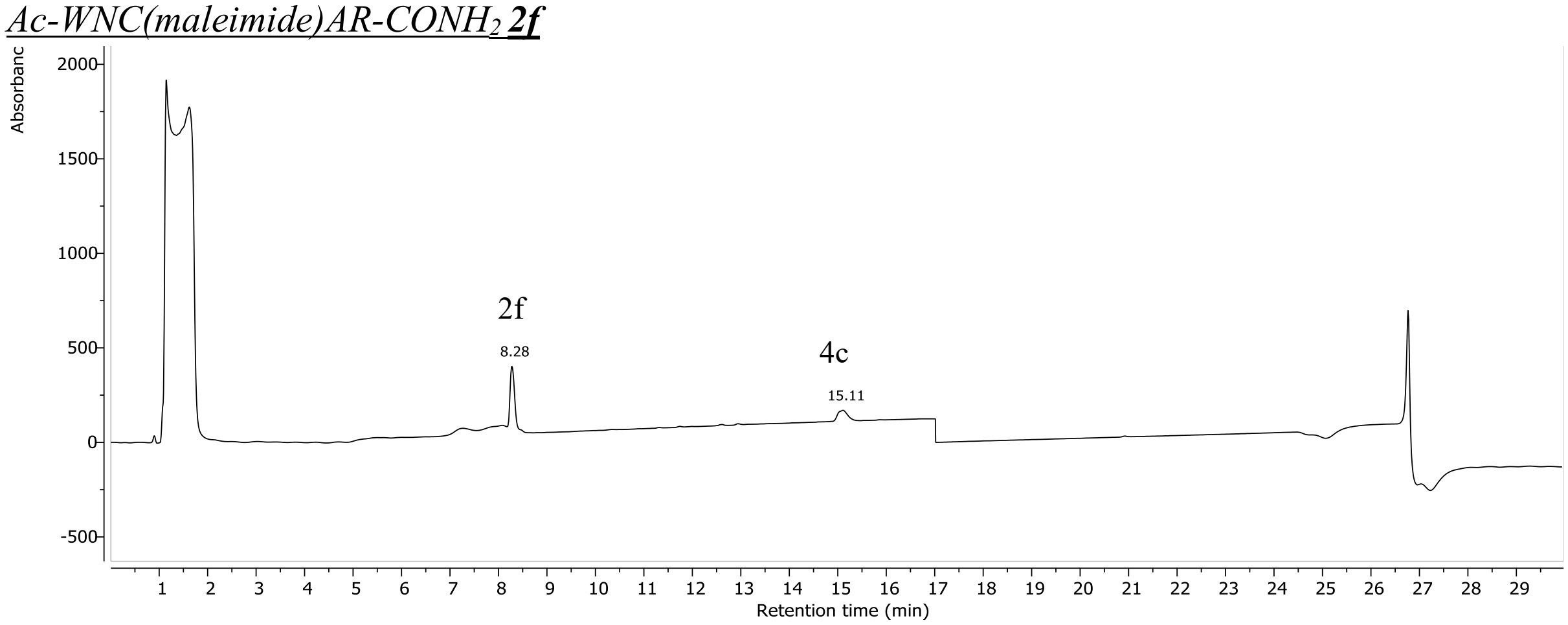

Analytical HPLC trace of purified peptide containing Ac-C(P(O)(OAr[1])$_2$)WAFR-CONH$_2$ ***4c*** *and Ac-WNC(maleimide)AR-CONH$_2$* ***2f***

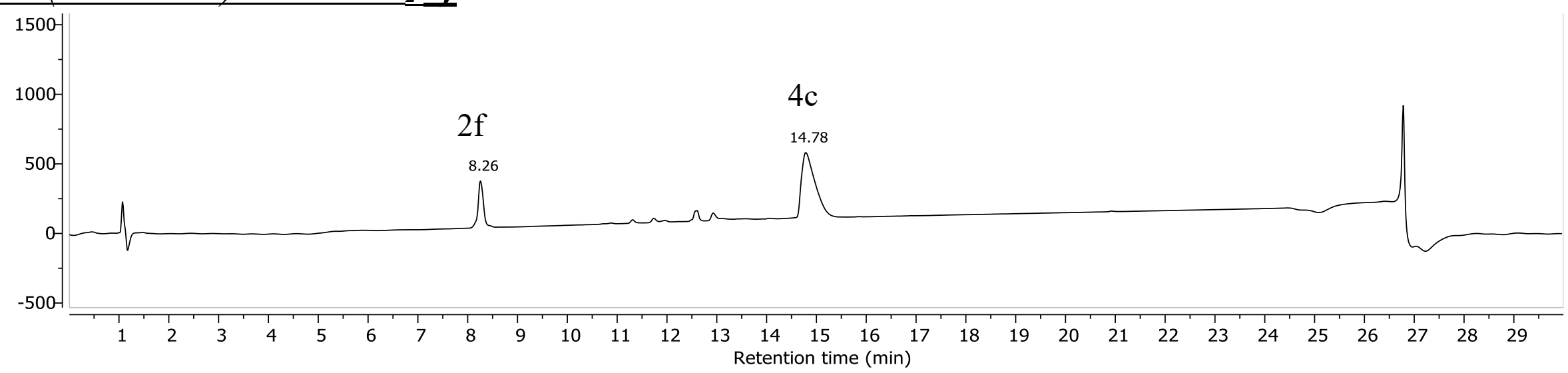

HRMS spectrum of purified peptide containing Ac-C(P(O)(OAr[1])$_2$)WAFR-CONH$_2$ ***4c*** *and Ac-WNC(maleimide)AR-CONH$_2$* ***2f***

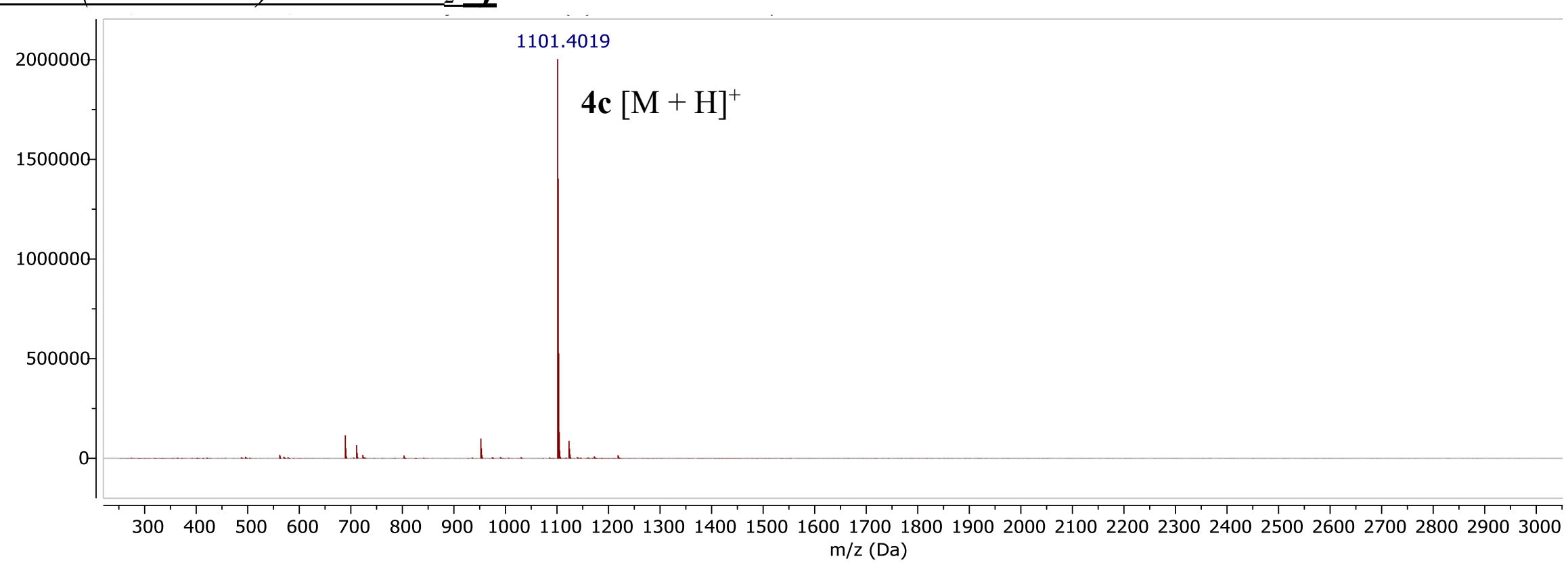

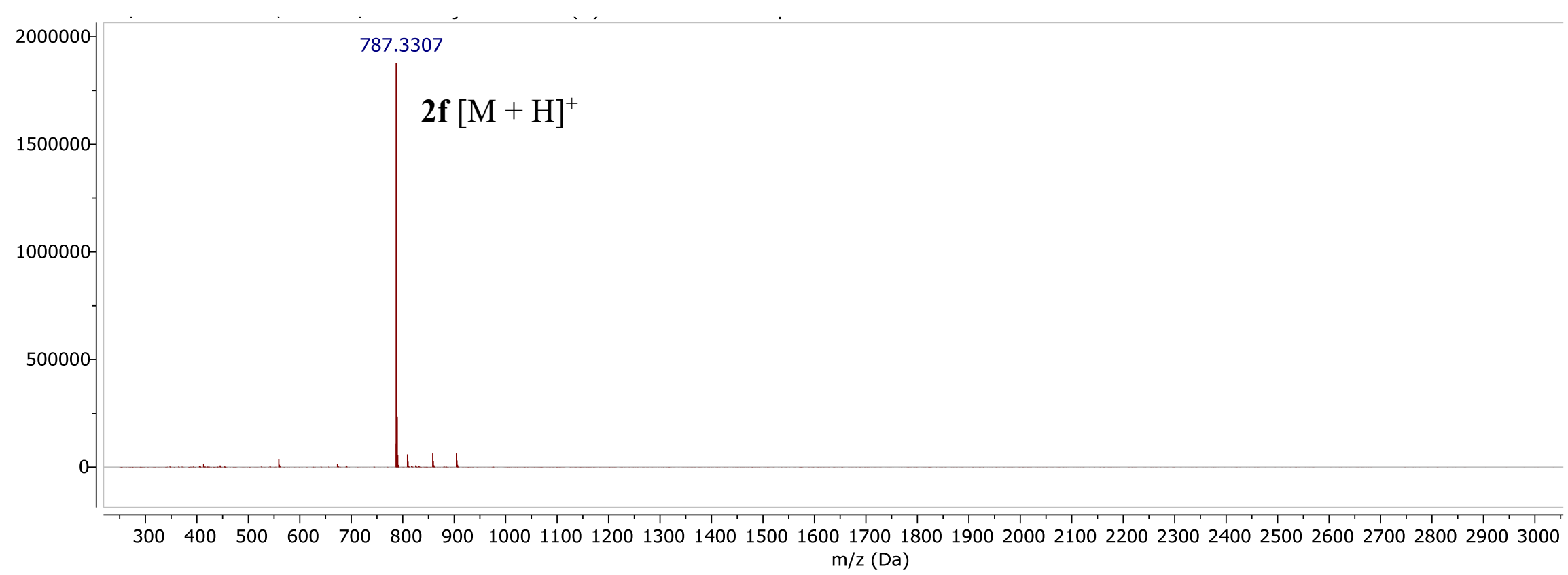

XXI.a. Supplementary Figure 12. Orthogonal Cleavage and Modification of Aryl, Ethyl and *tert*-Butyl Containing Disulfides in Fully Unprotected Peptide mixture of **1g**, **1h** and **1j**.

XXI.a.1. Orthogonal Cleavage of Aryl Containing Disulfide on **1g** from the Mixture of Peptides **1g**, **1h** and **1j**.

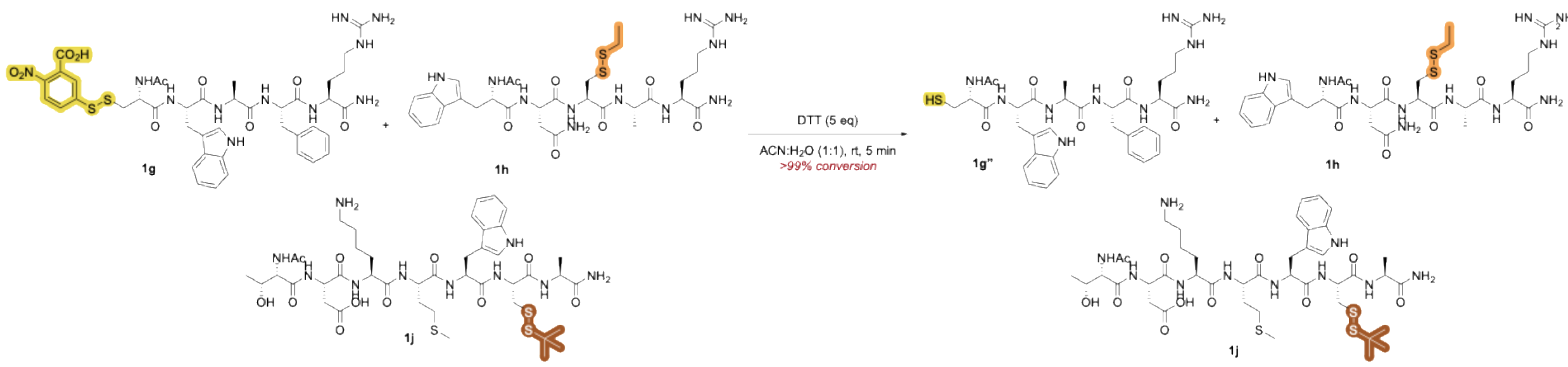

In a 1 dram vial equipped with a magnetic stirrer were added the purified peptide Ac-C(SAr[2])WAFR-CONH$_2$ **1g** (1 mg, 0.0011 mmol, 1 eq), Ac-WNC(SEt)AR-CONH$_2$ **1h** (1 mg, 0.0013 mmol, 1 eq) and Ac-TDKMWC(S^{t}Bu)A-CONH$_2$ **1j** (1 mg, 0.0010 mmol, 1 eq) dissolved in 300 µL of ACN:H$_2$O (1:1). In a separate 1 dram vial, dithiothreitol (DTT, 0.84 mg, 0.0055 mmol, 5 eq) was taken and dissolved in 100 µL of ACN:H$_2$O (1:1) and the solution was added at once to the peptide solution at room temperature and stirred the solution for 5 min. After that, the selective reduced thiol product Ac-CWAFR-CONH$_2$ **1g''** (pale white solid, 2.7 mg, >99% conversion) was determined via analytical HPLC using 0.1% formic acid (FA) in both mobile phases A and B. Following analysis, products were subsequently purified using this same method. Purified peptides **1g**, **1h**, and **1j** have the retention times in the mixture at 11.94, 9.98 and 11.54 respectively.

Peptide Ac-CWAFR-CONH$_2$ 1g'': **HRMS (ESI)** m/z: $C_{34}H_{47}N_{10}O_6S$ $[M+H]^+$ Calcd for 723.3395, Found 723.3434. **Peptide Ac-WNC(SEt)AR-CONH$_2$ 1h: HRMS (ESI)** m/z: $C_{31}H_{48}N_{11}O_7S_2$ $[M+H]^+$ Calcd for 750.3174, Found 750.3156. **Peptide Ac-TDKMWC(S^{t}Bu)A-CONH$_2$ 1j: HRMS (ESI)** m/z: $C_{42}H_{67}N_{10}O_{11}S_3$ $[M+H]^+$ Calcd for 983.4147, Found 983.4130; $C_{42}H_{66}N_{10}NaO_{11}S_3$ $[M+Na]^+$ Calcd for 1005.3967, Found 1005.3954

Analytical HPLC Method (C18, Water (A)/ACN (B); gradient: 5 to 85% B (0.1% formic acid in ACN) in 20 min, flow rate = 1.0 mL/min, I = 220 nm)**:** retention time of **1g'', 1h** and **1j** are 10.45, 9.86 and 11.55 min respectively.

*Analytical HPLC trace of the pure peptides Ac-CWAFR-CONH$_2$ **1g**, Ac-WNC(SEt)AR-CONH$_2$ **1h** and Ac-TDKMWC(S^{t}Bu)A-CONH$_2$ **1j***

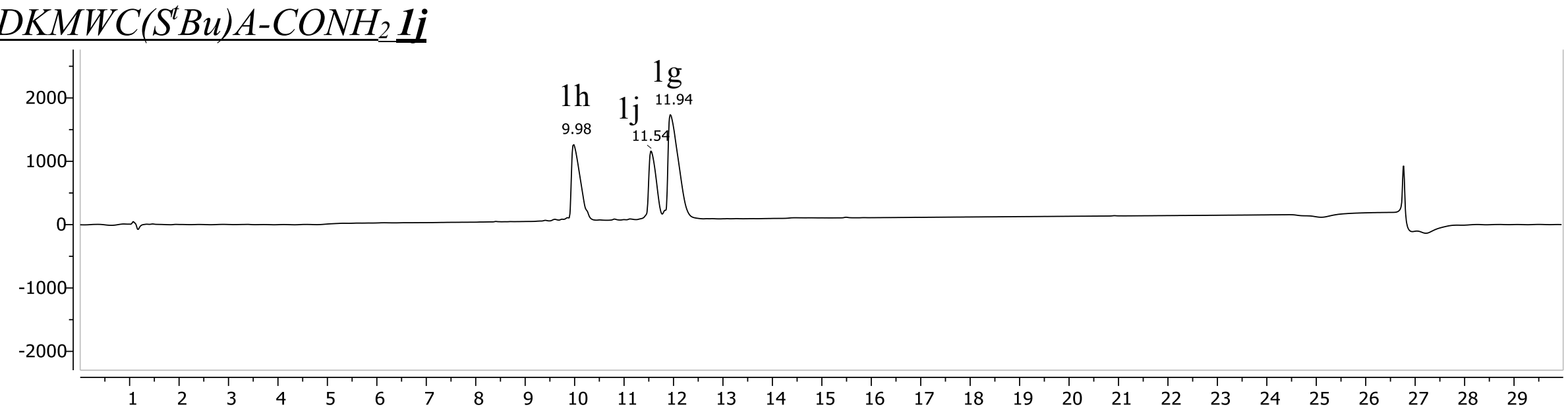

*Analytical HPLC trace of the crude reaction mixture containing Ac-CWAFR-CONH$_2$ **1g''**,Ac-WNC(SEt)AR-CONH$_2$ **1h** and Ac-TDKMWC(S^{t}Bu)A-CONH$_2$ **1j***

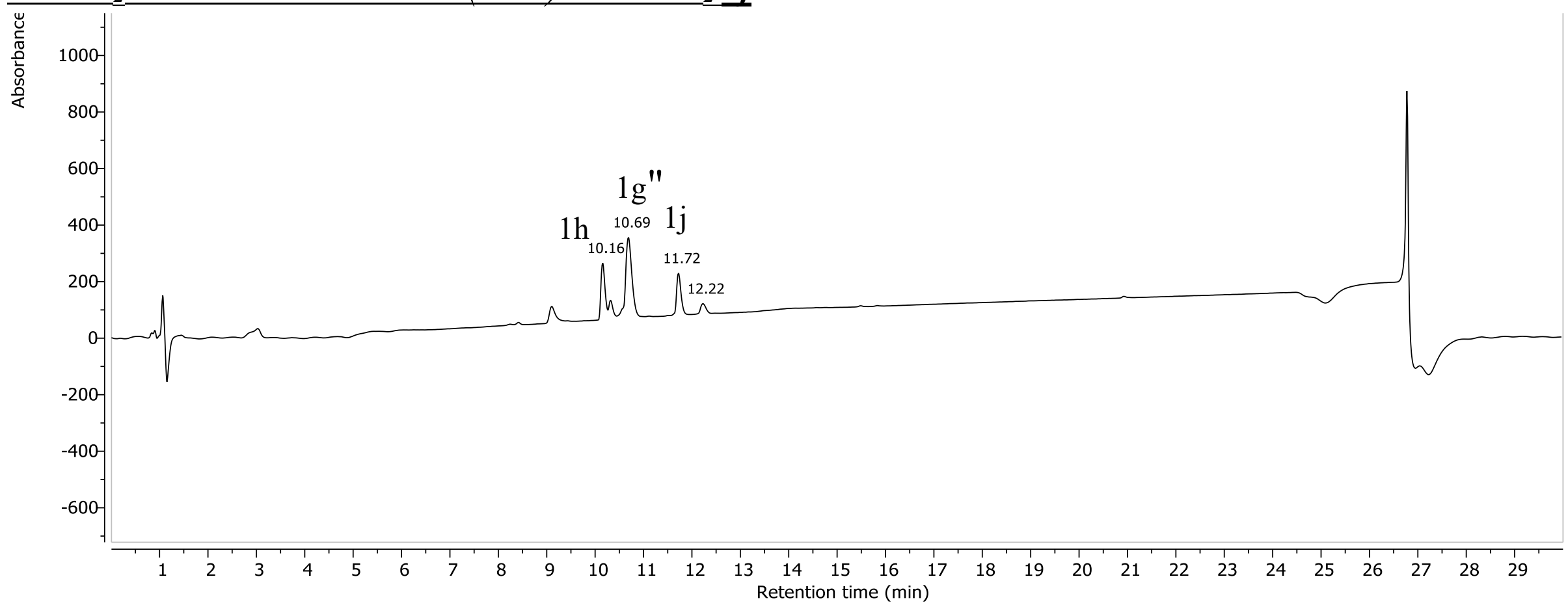

*Analytical HPLC trace of purified peptide mixture of Ac-CWAFR-CONH$_2$ **1g''**,Ac-WNC(SEt)AR-CONH$_2$ **1h** and Ac-TDKMWC(S^{t}Bu)A-CONH$_2$ **1j***

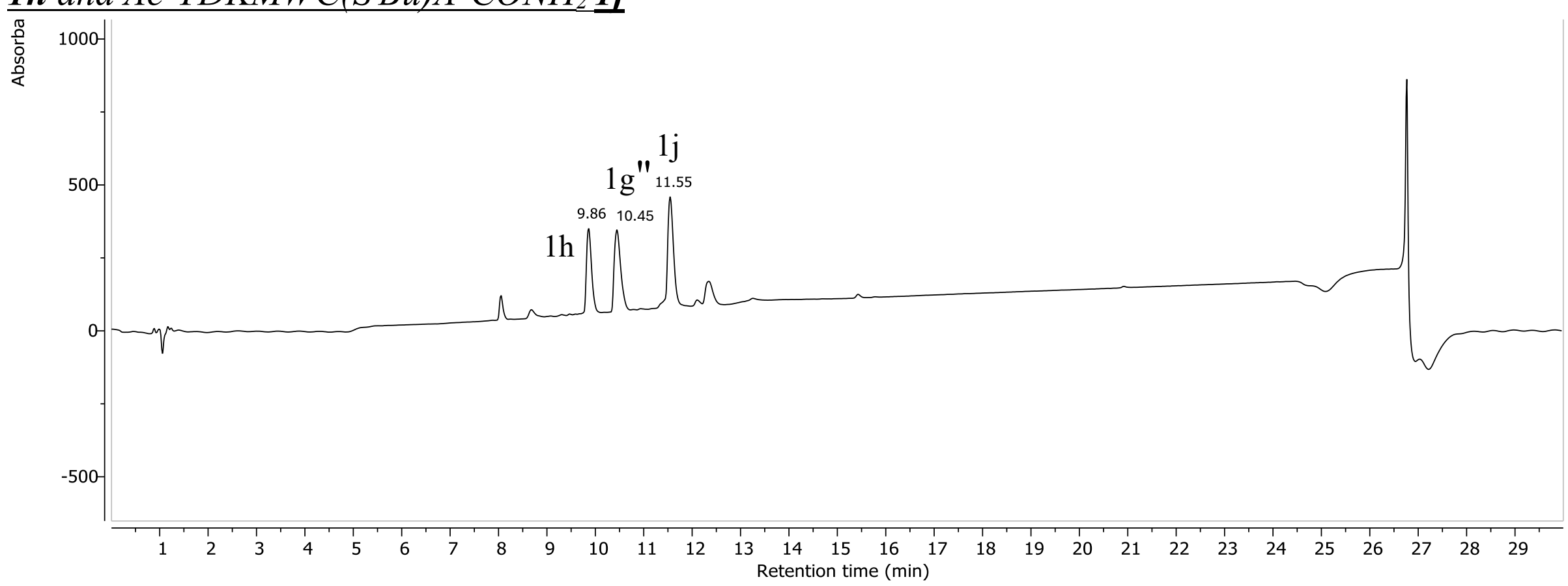

*HRMS spectrum of purified peptide mixture of Ac-CWAFR-CONH$_2$ **1g''**,Ac-WNC(SEt)AR-CONH$_2$ **1h** and Ac-TDKMWC(S^{t}Bu)A-CONH$_2$ **1j***

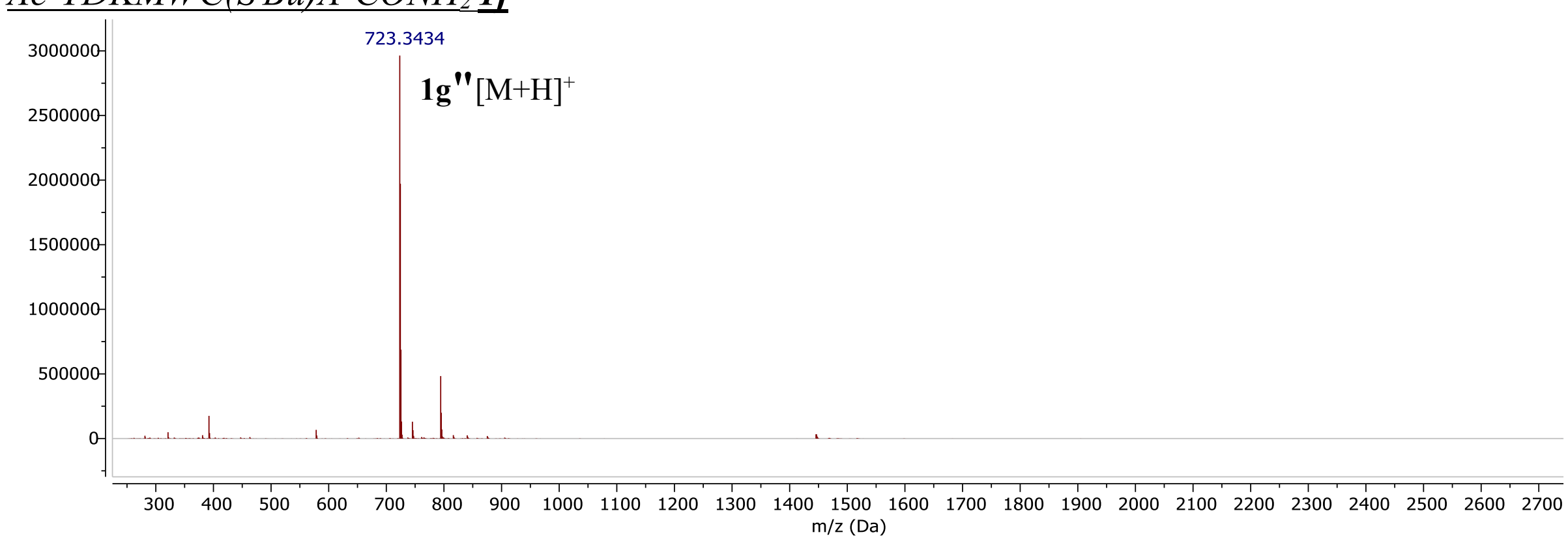

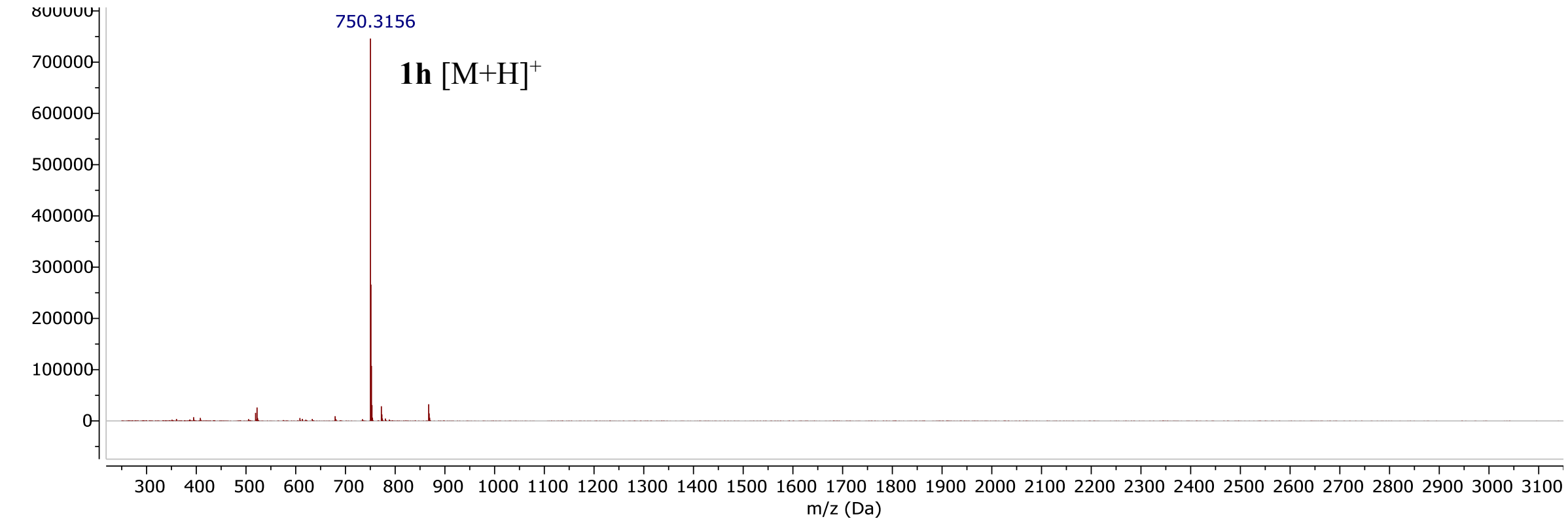

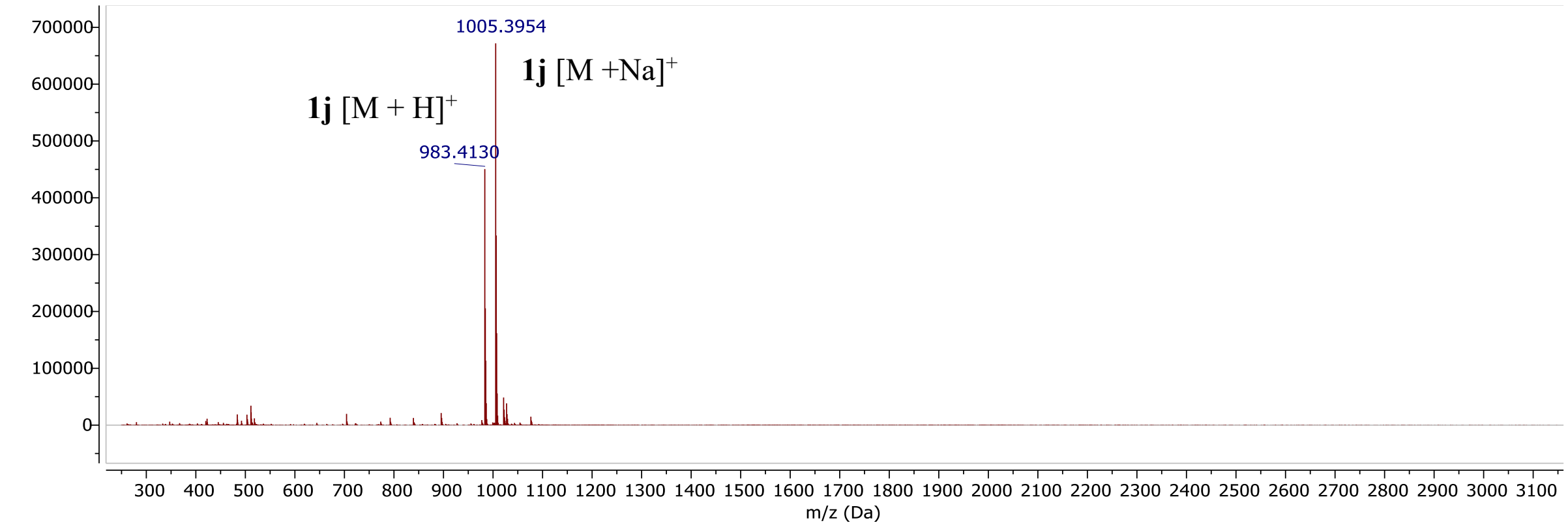

XXI.a.2. Orthogonal Modification of Free Cys on **1g''** from the Mixture **1g''**,**1h** and **1j**.

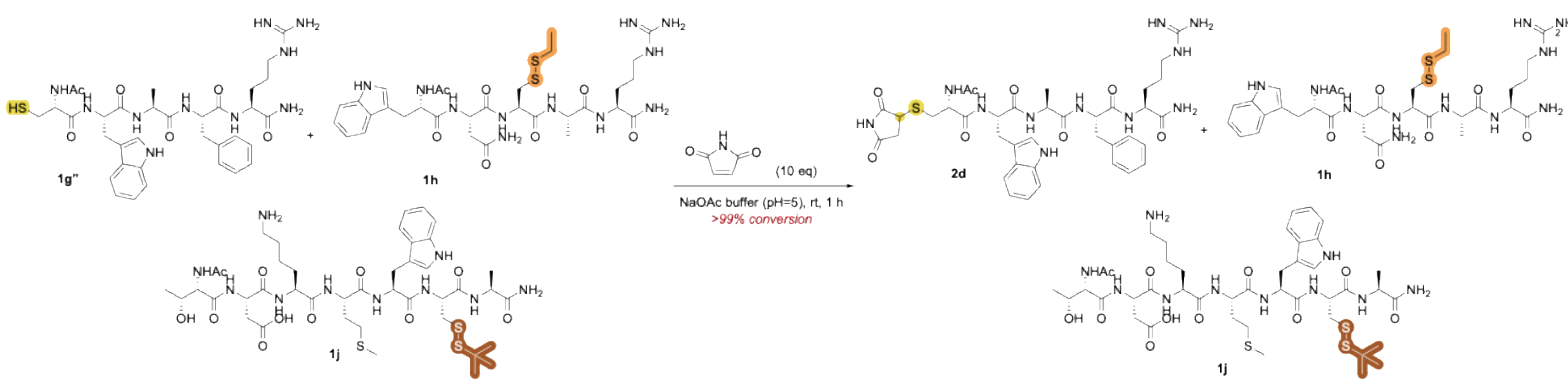

In a 1 dram vial equipped with a magnetic stirrer were added the purified peptide mixture Ac-CWAFR-$CONH_2$ **1g''**, Ac-WNC(SEt)AR-$CONH_2$ **1h** and Ac-TDKMWC(S^tBu)A-$CONH_2$ **1j** were dissolved in 300 µL of sodium acetate buffer (NaOAc buffer pH = 5) while stirring. In a separate 1 dram vial, maleimide (1.2 mg, 0.012 mmol, 10 eq) was taken and dissolved in NaOAc buffer (100 µL) and the solution was added dropwise to the peptide solution and resulting mixture was allowed to stir 1 h at room temperature. After that, the selective maleimide adduct peptide **2d** (pale white solid, 2.8 mg, >99% conversion) was determined via analytical HPLC using 0.1% formic acid (FA) in both mobile phases A and B. Following analysis, products were subsequently purified using this same method.

Peptide Ac-C(maleimide)WAFR-$CONH_2$ 2d: **HRMS (ESI)** m/z: $C_{38}H_{50}N_{11}O_8S$ $[M+H]^+$ Calcd for 820.3559, Found 820.3562. **Peptide Ac-WNC(SEt)AR-$CONH_2$ 1h: HRMS (ESI)** m/z: $C_{31}H_{48}N_{11}O_7S_2$ $[M+H]^+$ Calcd for 750.3174, Found 750.3156. **Peptide Ac-TDKMWC(S^tBu)A-$CONH_2$ 1j: HRMS (ESI)** m/z: $C_{42}H_{67}N_{10}O_{11}S_3$ $[M+H]^+$ Calcd for 983.4147, Found 983.4130; $C_{42}H_{66}N_{10}NaO_{11}S_3$ $[M+Na]^+$ Calcd for 1005.3967, Found 1005.3954

Analytical HPLC Method (C18, Water (A)/ACN (B); gradient: 5 to 85% B (0.1% formic acid in ACN) in 20 min, flow rate = 1.0 mL/min, I = 220 nm)**:** retention time of **2d**, **1h** and **1j** are 10.02, 9.88 and 11.44 min respectively.

*Analytical HPLC trace of the crude reaction mixture containing Ac-C(maleimide)WAFR-CONH₂ **2d**, Ac-WNC(SEt)AR-CONH₂ **1h** and Ac-TDKMWC(S^tBu)A-CONH₂ **1j***

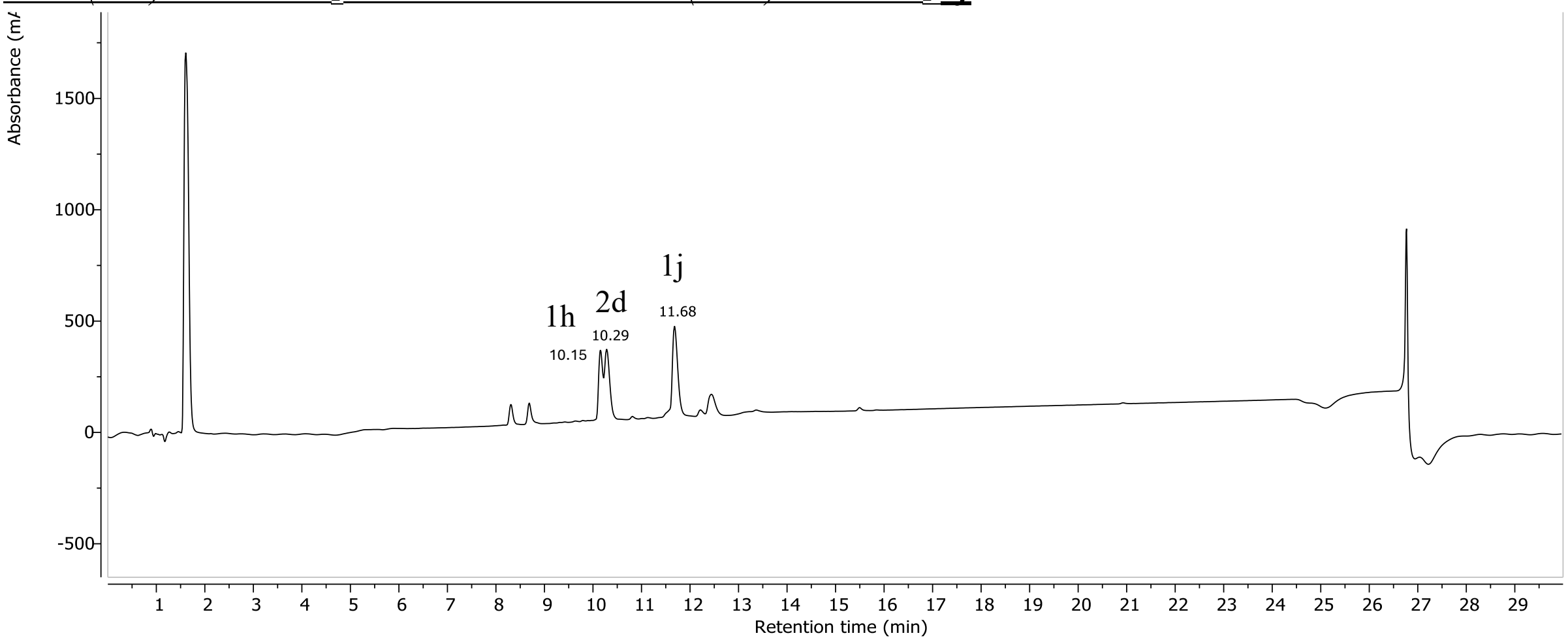

*Analytical HPLC trace of purified peptide mixture of Ac-C(maleimide)WAFR-CONH₂ **2d**, Ac-WNC(SEt)AR-CONH₂ **1h** and Ac-TDKMWC(S^tBu)A-CONH₂ **1j***

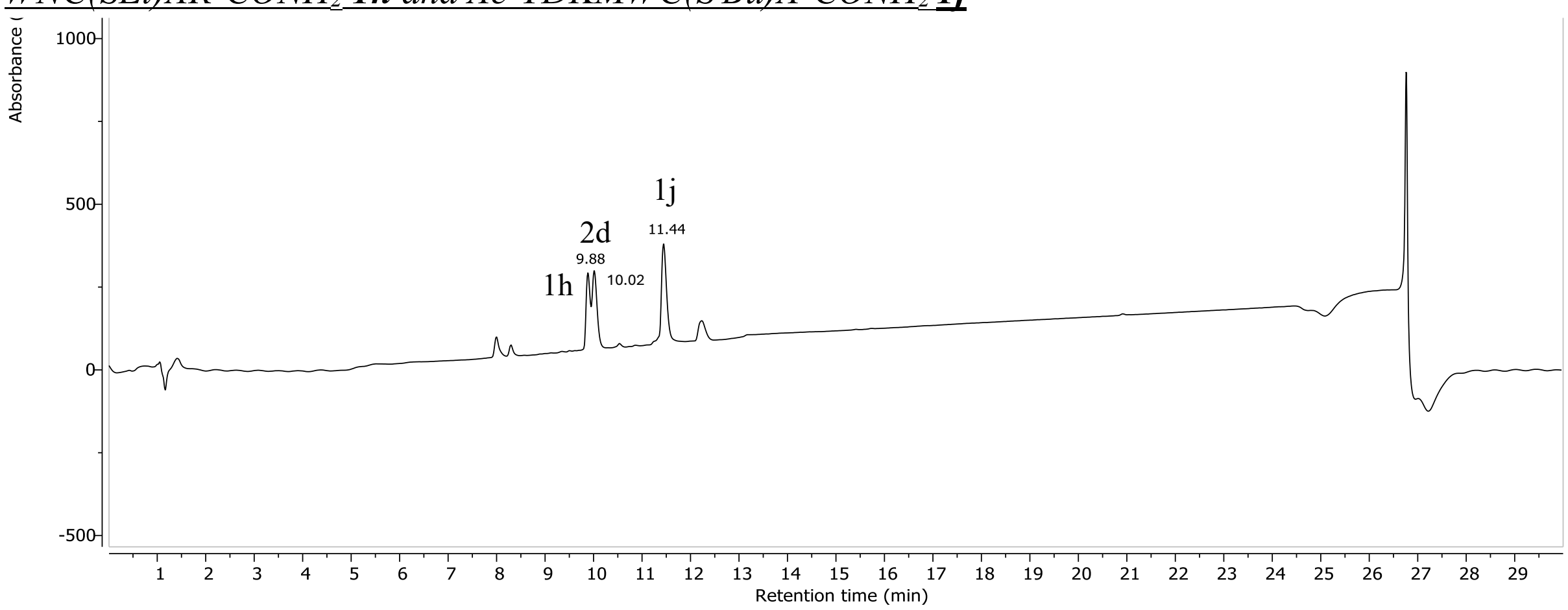

*HRMS spectrum of purified peptide mixture of Ac-C(maleimide)WAFR-CONH$_2$ **2d**, Ac-WNC(SEt)AR-CONH$_2$ **1h** and Ac-TDKMWC(S^{t}Bu)A-CONH$_2$ **1j***

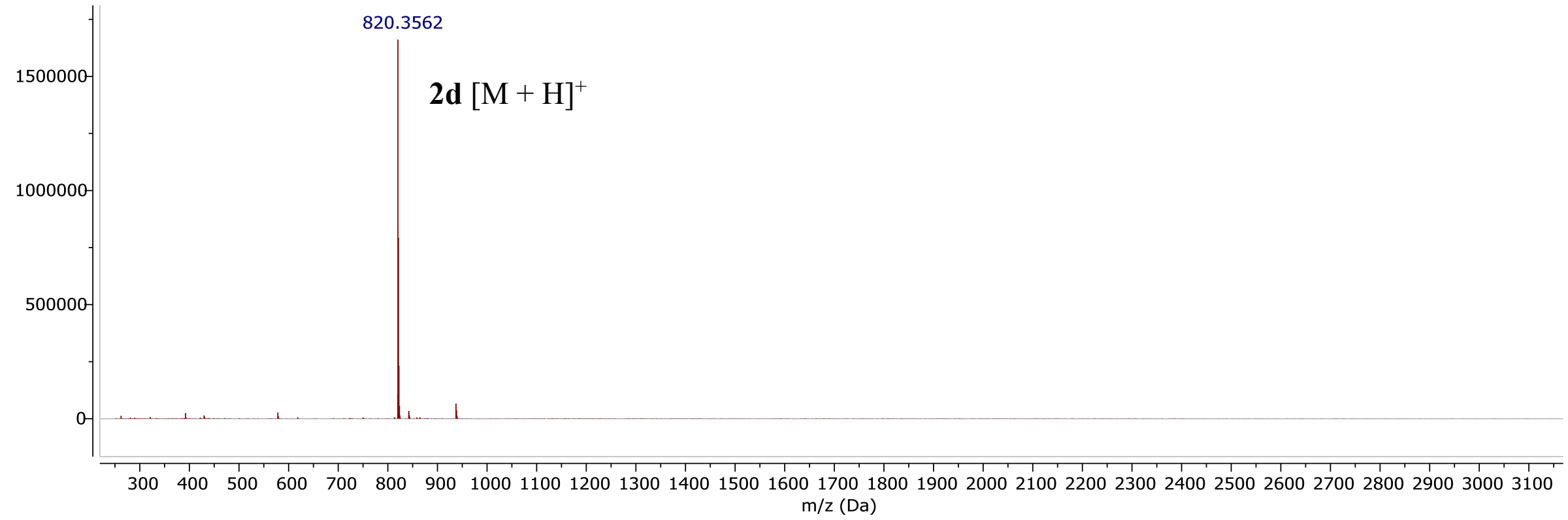

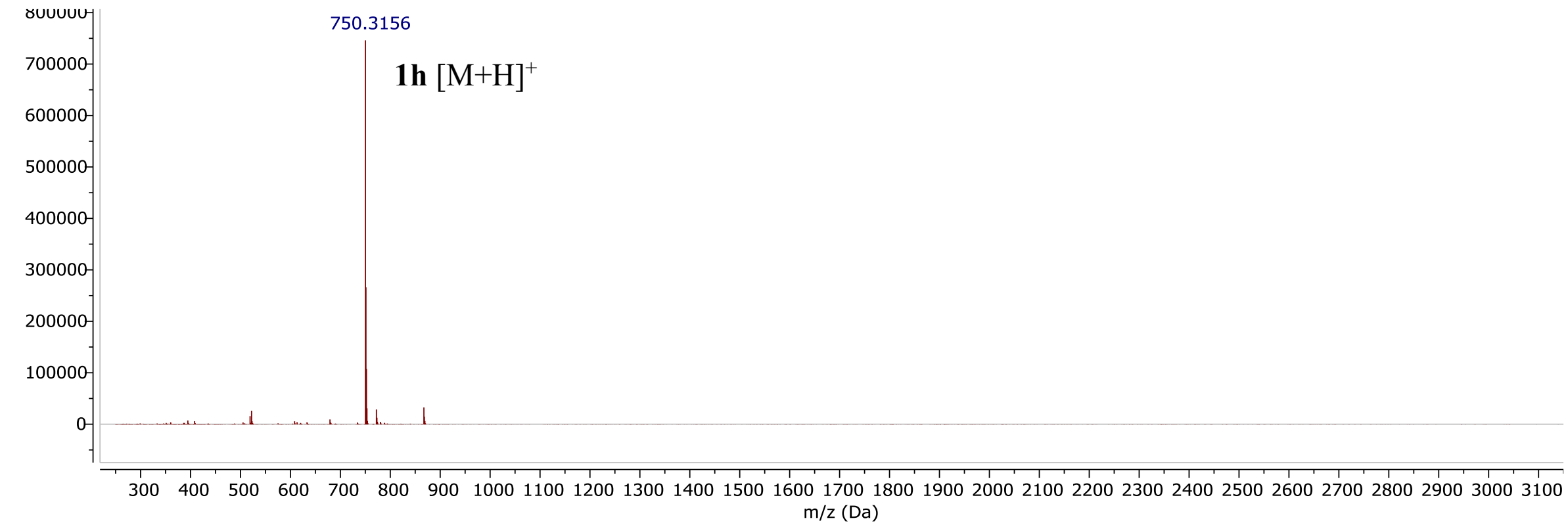

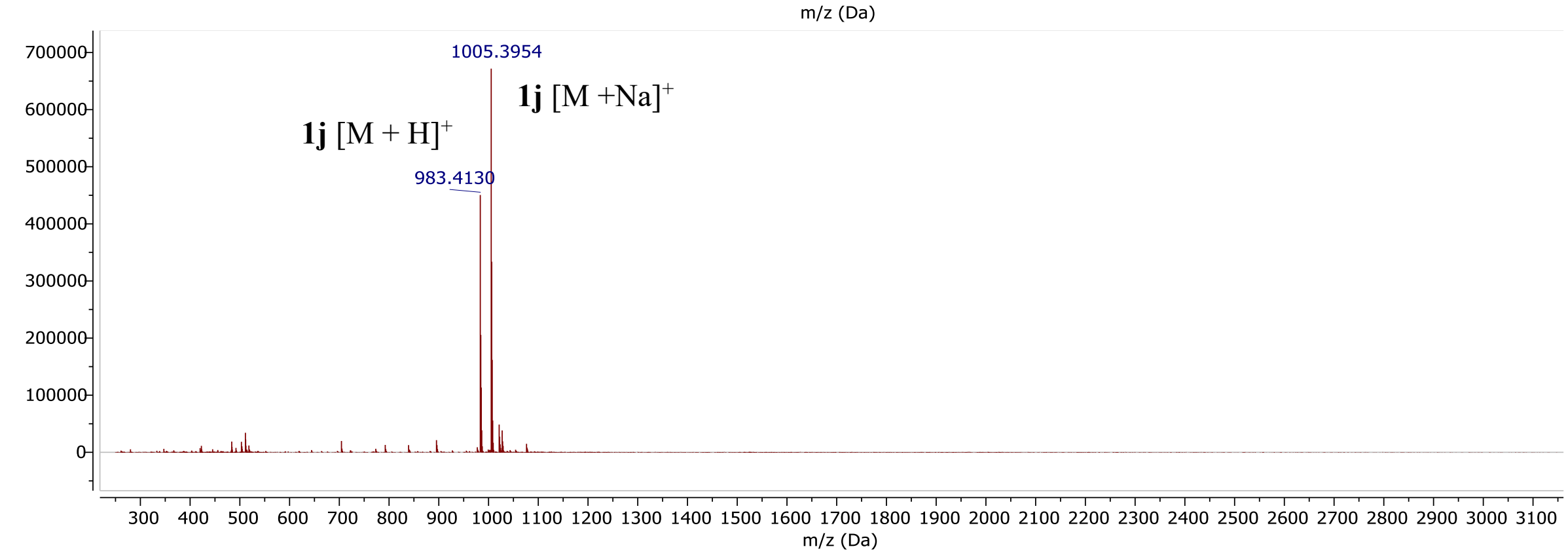

XXI.a.3. Orthogonal Cleavage of Ethyl Containing Disulfide on **1h** in the Mixture of Peptides **1h**, **1j** and **2d**.

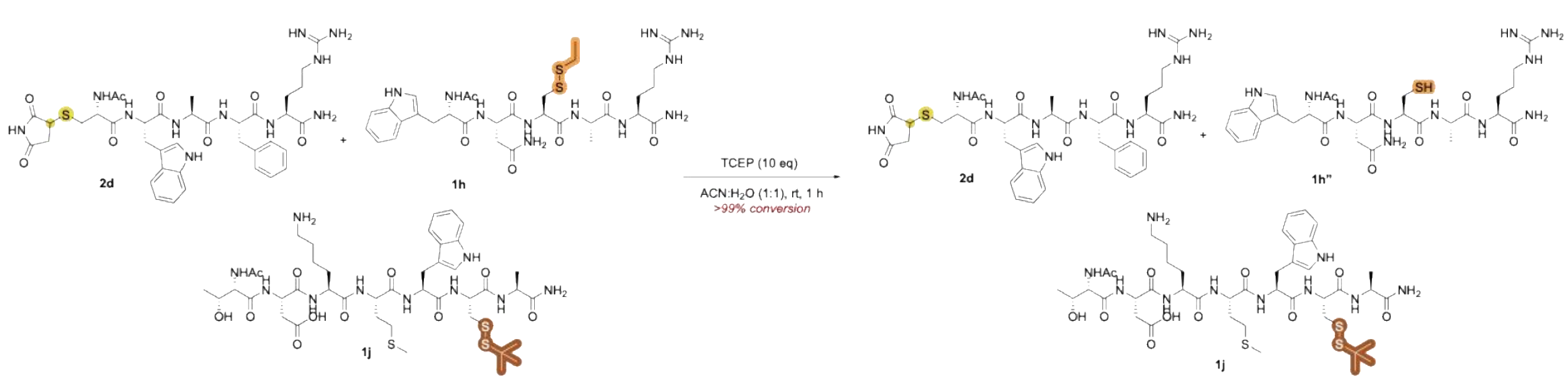

In a 1 dram vial equipped with a magnetic stirrer was added the purified peptide mixture Ac-C(maleimide)WAFR-CONH$_2$ **2d**, Ac-WNC(SEt)AR-CONH$_2$ **1h** and Ac-TDKMWC(S^{t}Bu)A-CONH$_2$ **1j** were dissolved in ACN (300 µL), and H_2O (200 µL) while stirring. In a separate 1 dram vial, TCEP (3.7 mg, 0.013 mmol, 10 eq) was taken and dissolved in H_2O (100 µL) and the solution was added dropwise to the peptide solution and resulting mixture was allowed to stir 1 h at room temperature. After that, the selective reduced thiol product **1h''** (pale white solid, 2.7 mg, >99% conversion) was determined via analytical HPLC using 0.1% formic acid (FA) in both mobile phases A and B. Following analysis, products were subsequently purified using this same method.

Peptide Ac-WNCAR-CONH$_2$ 1h'': HRMS (ESI) m/z: $C_{29}H_{44}N_{11}O_7S$ [M+H]$^+$ Calcd for 690.3140, Found 690.3146. **Peptide Ac-C(maleimide)WAFR-CONH$_2$ 2d**: **HRMS (ESI)** m/z: $C_{38}H_{50}N_{11}O_8S$ [M+H]$^+$ Calcd for 820.3559, Found 820.3562. **Peptide Ac-TDKMWC(S^{t}Bu)A-CONH$_2$ 1j: HRMS (ESI)** m/z: $C_{42}H_{67}N_{10}O_{11}S_3$ [M+H]$^+$ Calcd for 983.4147, Found 983.4130; $C_{42}H_{66}N_{10}NaO_{11}S_3$ [M+Na]$^+$ Calcd for 1005.3967, Found 1005.3954.

Analytical HPLC Method (C18, Water (A)/ACN (B); gradient: 5 to 85% B (0.1% formic acid in ACN) in 20 min, flow rate = 1.0 mL/min, I = 220 nm)**:** retention time of **1h'', 2d** and **1j** are 8.41, 10.3 and 11.7 min respectively.

*Analytical HPLC trace of the crude reaction mixture containing Ac-C(maleimide)WAFR-CONH$_2$ **2d**, Ac-WNCAR-CONH$_2$ **1h''** and Ac-TDKMWC(S^{t}Bu)A-CONH$_2$ **1j***

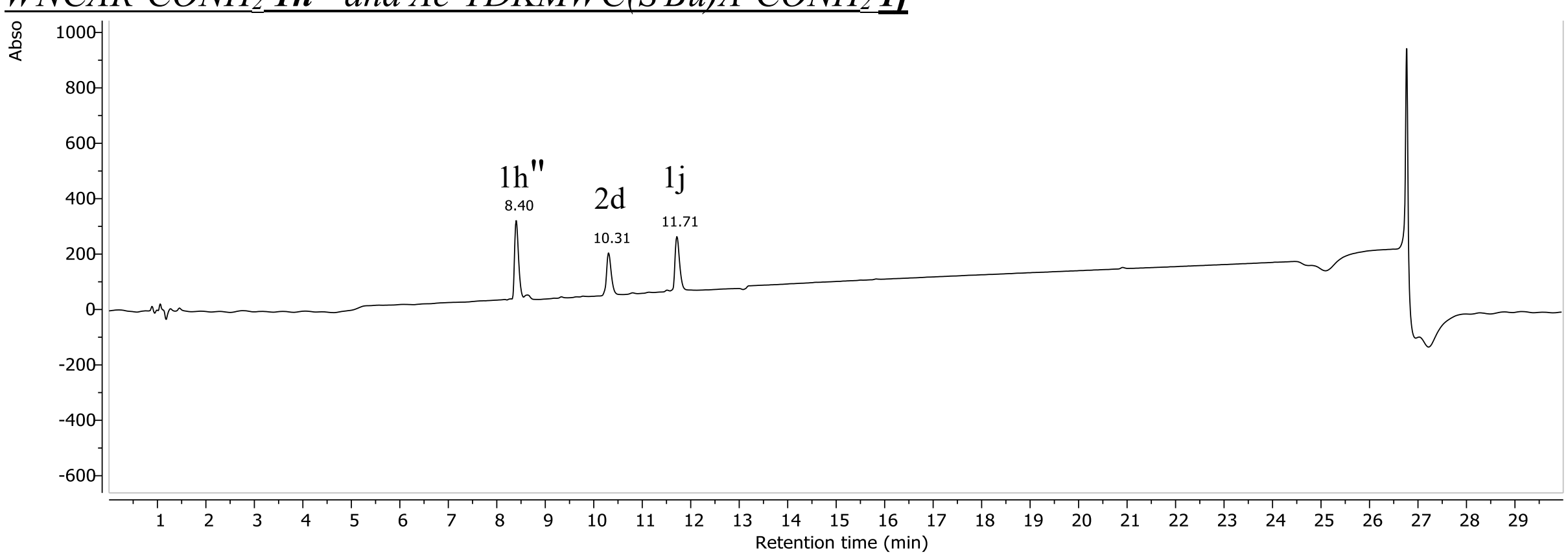

*Analytical HPLC trace of purified peptide mixture of Ac-C(maleimide)WAFR-CONH$_2$ **2d**, Ac-WNCAR-CONH$_2$ **1h''** and Ac-TDKMWC(S^{t}Bu)A-CONH$_2$ **1j***

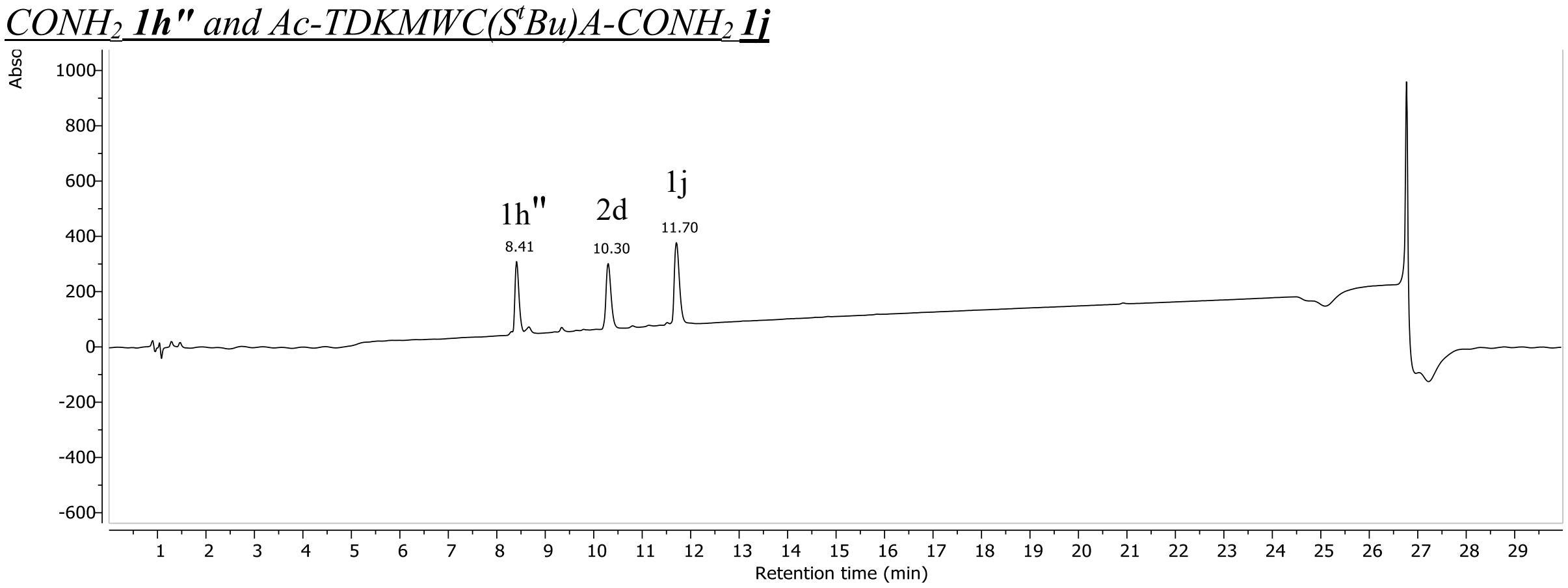

*HRMS spectrum of purified peptide mixture of Ac-C(maleimide)WAFR-CONH$_2$ **2d**, Ac-WNCAR-CONH$_2$ **1h''** and Ac-TDKMWC(S^{t}Bu)A-CONH$_2$ **1j***

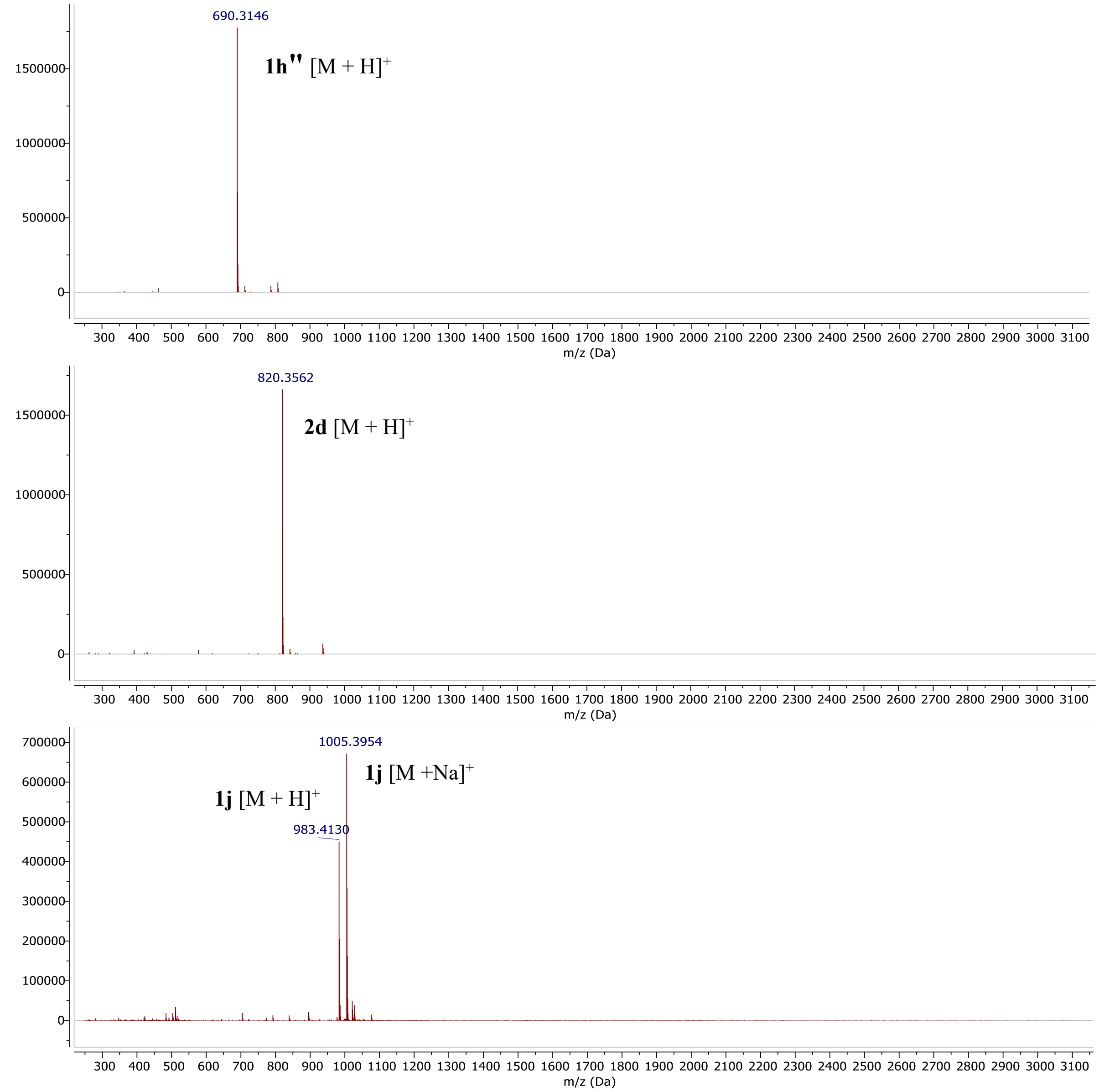

XXI.a.4. Orthogonal Modification of Free Cys in Peptide **1h''** in the Mixture of **1h''**, **1j** and **2d**.

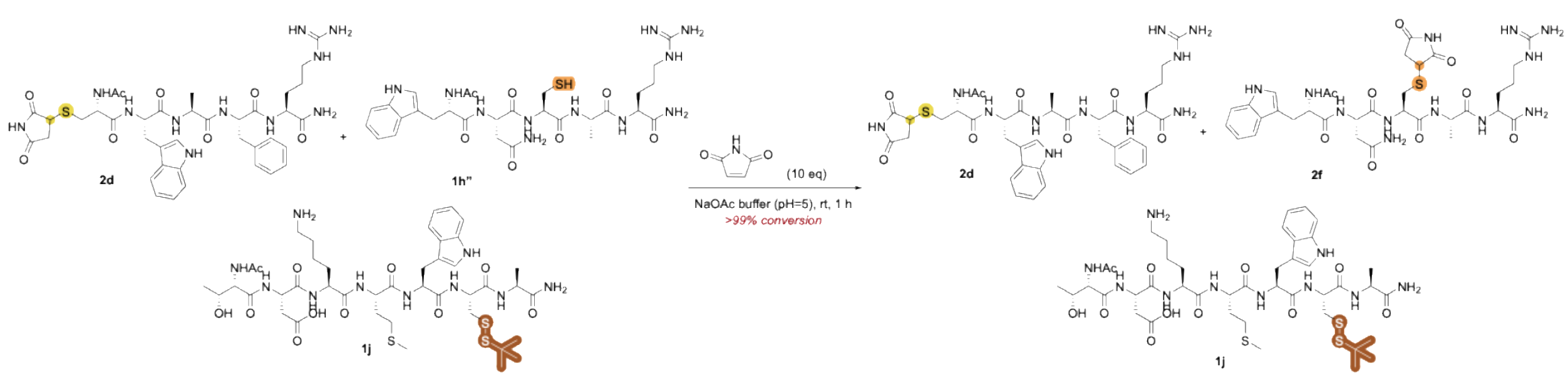

In a 1 dram vial equipped with a magnetic stirrer was added the purified peptide mixture Ac-C(maleimide)WAFR-$CONH_2$ **2d** Ac-WNCAR-$CONH_2$ **1h"** and Ac-TDKMWC(S^{t}Bu)A-$CONH_2$ **1j** were dissolved in 300 µL of sodium acetate buffer (NaOAc buffer pH = 5) while stirring. In a separate 1 dram vial, maleimide (1.2 mg, 0.012 mmol, 10 eq) was taken and dissolved in NaOAc buffer (100 µL) and the solution was added dropwise to the peptide solution and resulting mixture was allowed to stir 1 h at room temperature. After that, the selective maleimide adduct peptide **2f** (pale white solid, 2.9 mg, >99% conversion) was determined via analytical HPLC using 0.1% formic acid (FA) in both mobile phases A and B. Following analysis, products were subsequently purified using this same method.

Peptide Ac-WNC(maleimide)AR-$CONH_2$ 2f: **HRMS (ESI)** m/z: $C_{33}H_{47}N_{12}O_9S$ $[M+H]^+$ Calcd for 787.3304, Found 787.3276. **Peptide Ac-C(maleimide)WAFR-$CONH_2$ 2d**: **HRMS (ESI)** m/z: $C_{38}H_{50}N_{11}O_8S$ $[M+H]^+$ Calcd for 820.3559, Found 820.3562. **Peptide Ac-TDKMWC(S^{t}Bu)A-$CONH_2$ 1j: HRMS (ESI)** m/z: $C_{42}H_{67}N_{10}O_{11}S_3$ $[M+H]^+$ Calcd for 983.4147, Found 983.4130; $C_{42}H_{66}N_{10}NaO_{11}S_3$ $[M+Na]^+$ Calcd for 1005.3967, Found 1005.3954.

Analytical HPLC Method (C18, Water (A)/ACN (B); gradient: 5 to 85% B (0.1% formic acid in ACN) in 20 min, flow rate = 1.0 mL/min, I = 220 nm)**:** retention time of **2f**, **2d** and **1j** are 8.26, 10.28 and 11.69 min respectively.

*Analytical HPLC trace of the crude reaction mixture containing Ac-C(maleimide)WAFR-$CONH_2$ **2d**, Ac-WNC(maleimide)AR-$CONH_2$ **2f** and Ac-TDKMWC(S^{t}Bu)A-$CONH_2$ **1j***

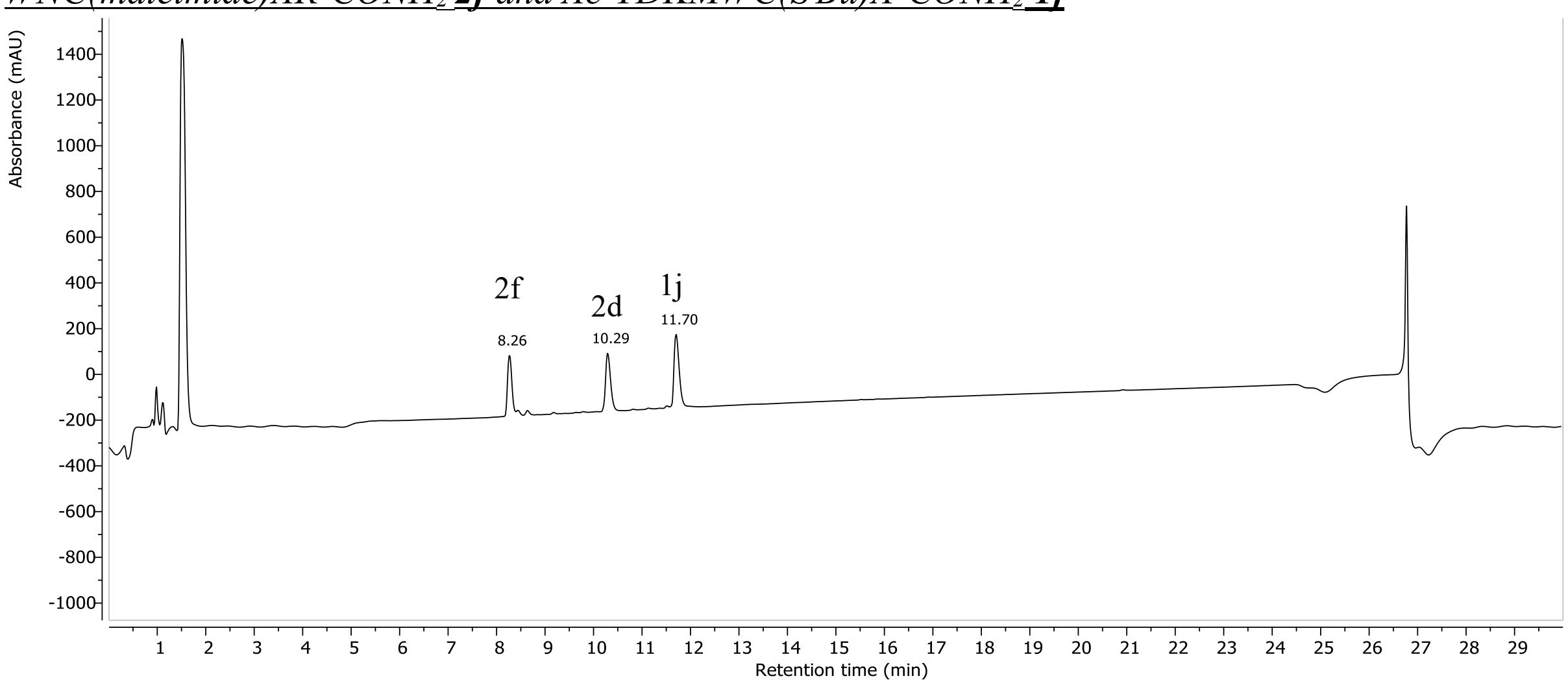

*Analytical HPLC trace of purified peptide mixture of Ac-C(maleimide)WAFR-CONH$_2$ **2d**, Ac-WNC(maleimide)AR-CONH$_2$ **2f** and Ac-TDKMWC(S^{t}Bu)A-CONH$_2$ **1j***

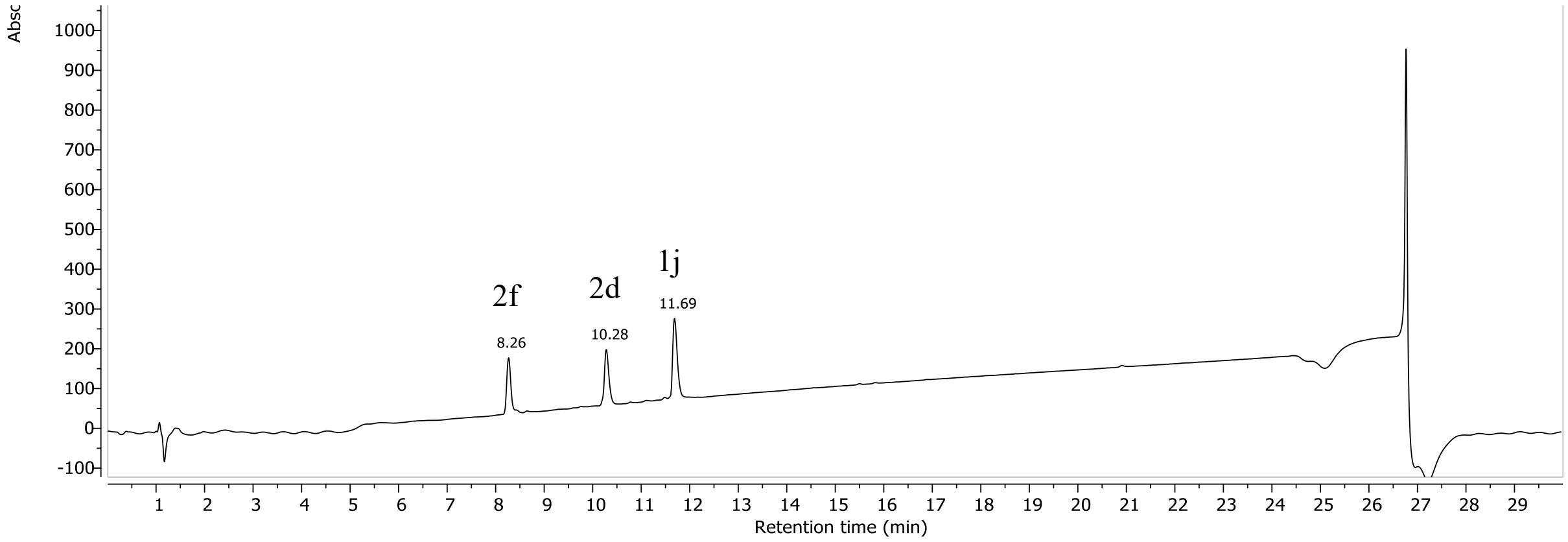

*HRMS spectrum of purified peptide mixture of Ac-C(maleimide)WAFR-CONH$_2$ **2d**, Ac-WNC(maleimide)AR-CONH$_2$ **2f** and Ac-TDKMWC(S^{t}Bu)A-CONH$_2$ **1j***

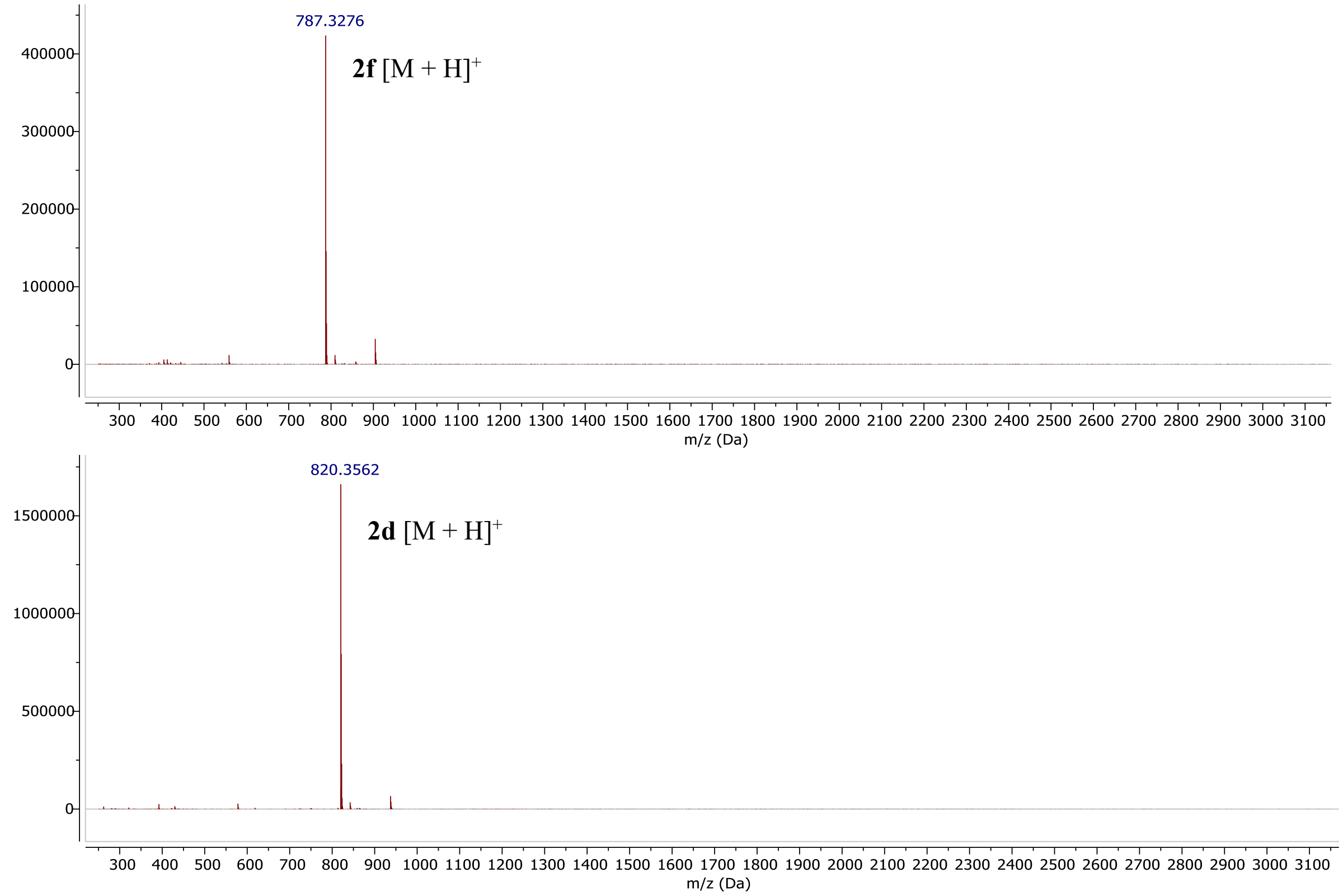

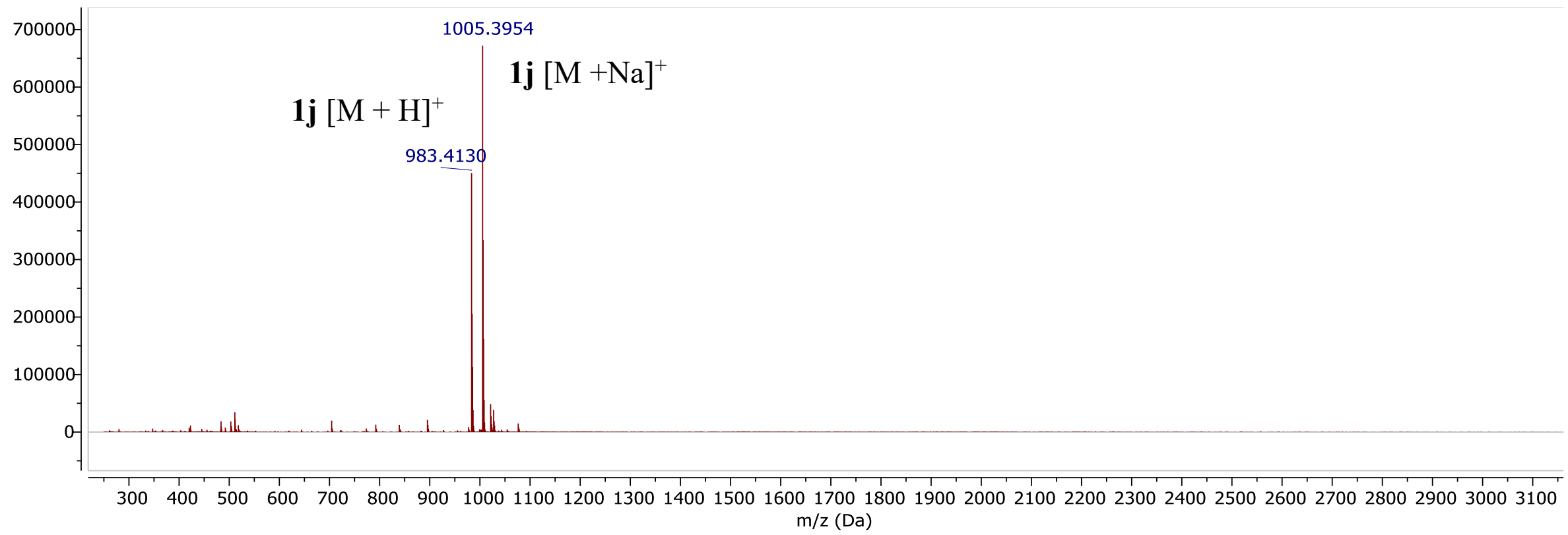

XXI.a.5. Orthogonal Cleavage of *tert*-Butyl Containing Disulfide on **1j** in the Mixture of Peptides **2f**, **1j** and **2d**.

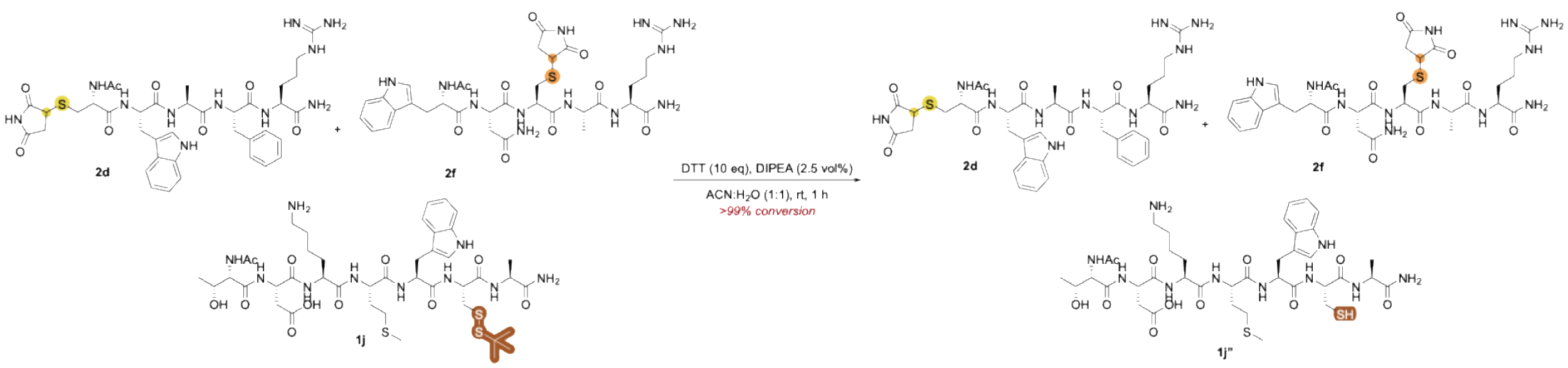

In a 1 dram vial equipped with a magnetic stirrer was added the purified peptide mixture Ac-C(maleimide)WAFR-CONH$_2$ **2d**, Ac-WNC(maleimide)AR-CONH$_2$ **2f** and Ac-TDKMWC(S^{t}Bu)A-CONH$_2$ **1j** were dissolved in ACN (500 µL), and H_2O (200 µL) while stirring. In a separate 1 dram vial, dithiothreitol (DTT, 1.6 mg, 0.01 mmol, 10 eq) was taken and dissolved in H_2O (300 µL) and the solution was added dropwise to the peptide solution followed by addition of *N,N*-diisopropylethylamine (DIPEA, 25 µL) at once and resulting mixture was allowed to stir 1 h at room temperature. After that, the selective reduced thiol product **1j"** (pale white solid, 2.8 mg, >99% conversion) was determined via analytical HPLC using 0.1% formic acid (FA) in both mobile phases A and B. Following analysis, product was subsequently purified using this same method.

Peptide Ac-TDKMWCA-CONH$_2$ 1j": **HRMS (ESI)** m/z: $C_{38}H_{59}N_{10}O_{11}S_2$ [M+H]$^+$ Calcd for 895.3801, Found 895.3786; $C_{38}H_{59}N_{10}NaO_{11}S_2$ [M+Na]$^+$ Calcd for 917.3620, Found 917.3606. **Peptide Ac-WNC(maleimide)AR-CONH$_2$ 2f**: **HRMS (ESI)** m/z: $C_{33}H_{47}N_{12}O_9S$ [M+H]$^+$ Calcd for 787.3304, Found 787.3276. **Peptide Ac-C(maleimide)WAFR-CONH$_2$ 2d**: **HRMS (ESI)** m/z: $C_{38}H_{50}N_{11}O_8S$ [M+H]$^+$ Calcd for 820.3559, Found 820.3562.

Analytical HPLC Method (C18, Water (A)/ACN (B); gradient: 5 to 85% B (0.1% formic acid in ACN) in 20 min, flow rate = 1.0 mL/min, I = 220 nm)**:** retention time of **1j", 2d** and **2f** are 9.26, 10.28 and 8.27 min respectively.

*Analytical HPLC trace of the crude reaction mixture containing Ac-C(maleimide)WAFR-CONH$_2$ **2d**, Ac-WNC(maleimide)AR-CONH$_2$ **2f** and Ac-TDKMWCA-CONH$_2$ **1j''***

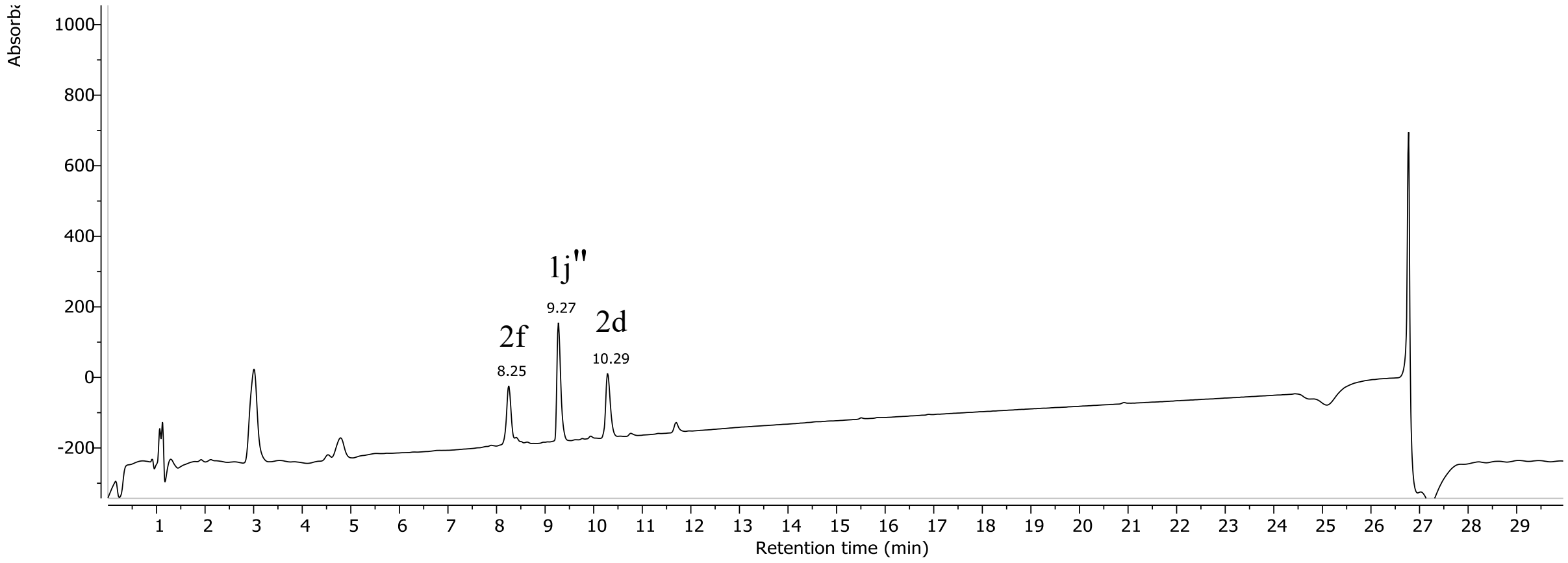

*Analytical HPLC trace of purified peptide mixture of Ac-C(maleimide)WAFR-CONH$_2$ **2d**, Ac-WNC(maleimide)AR-CONH$_2$ **2f** and Ac-TDKMWCA-CONH$_2$ **1j''***

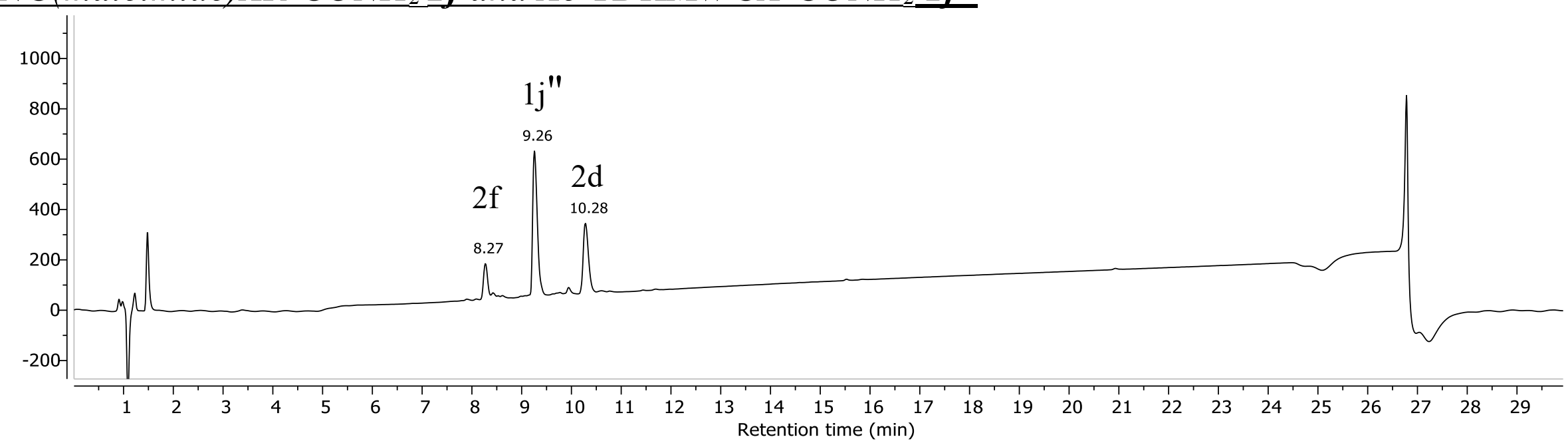

*HRMS spectrum of purified peptide mixture of Ac-C(maleimide)WAFR-CONH$_2$ **2d**, Ac-WNC(maleimide)AR-CONH$_2$ **2f** and Ac-TDKMWCA-CONH$_2$ **1j''***

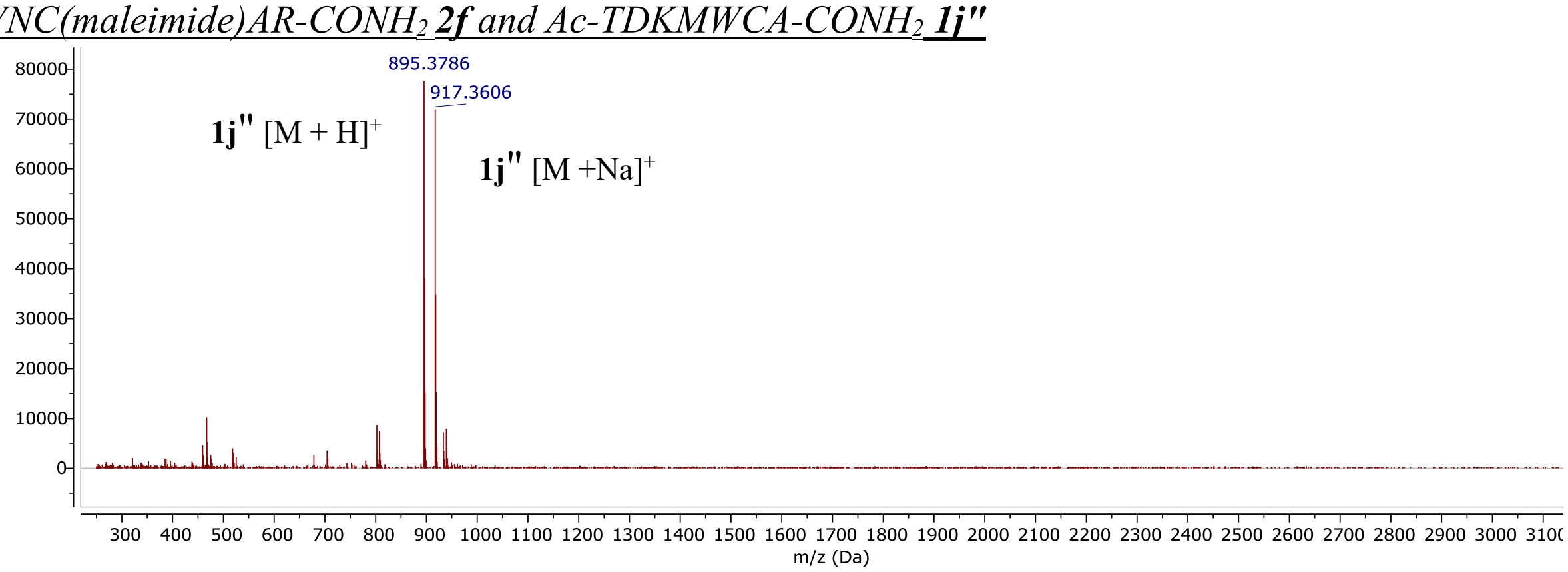

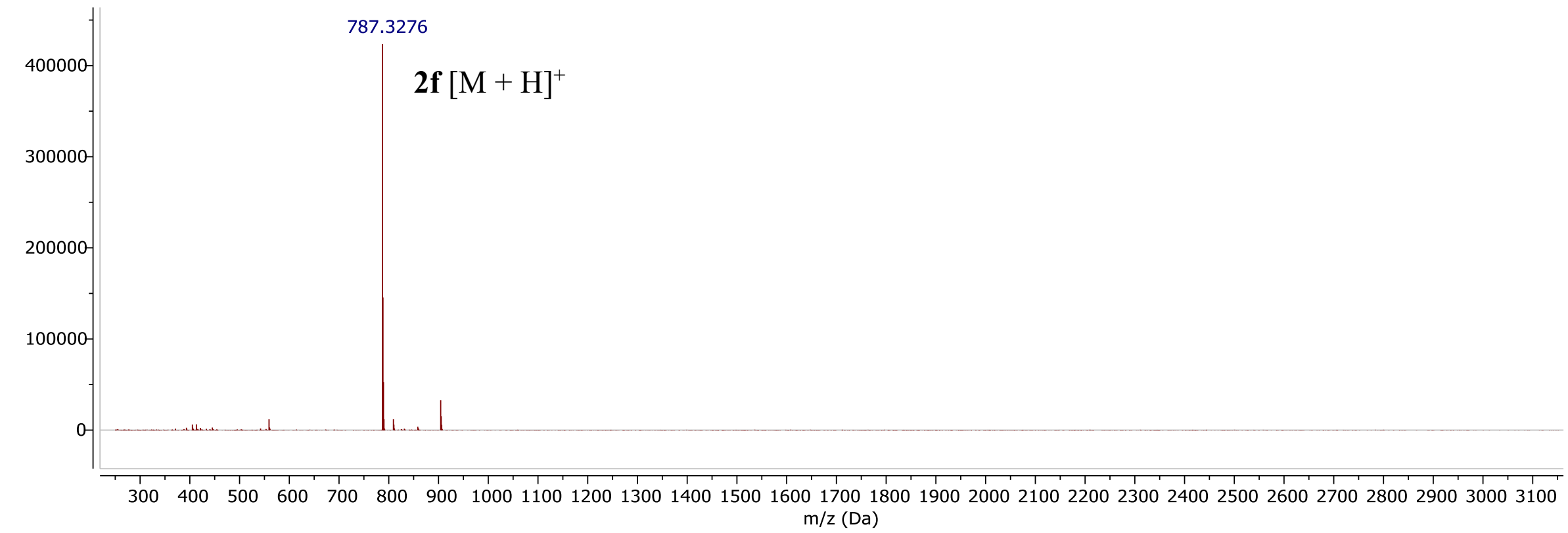

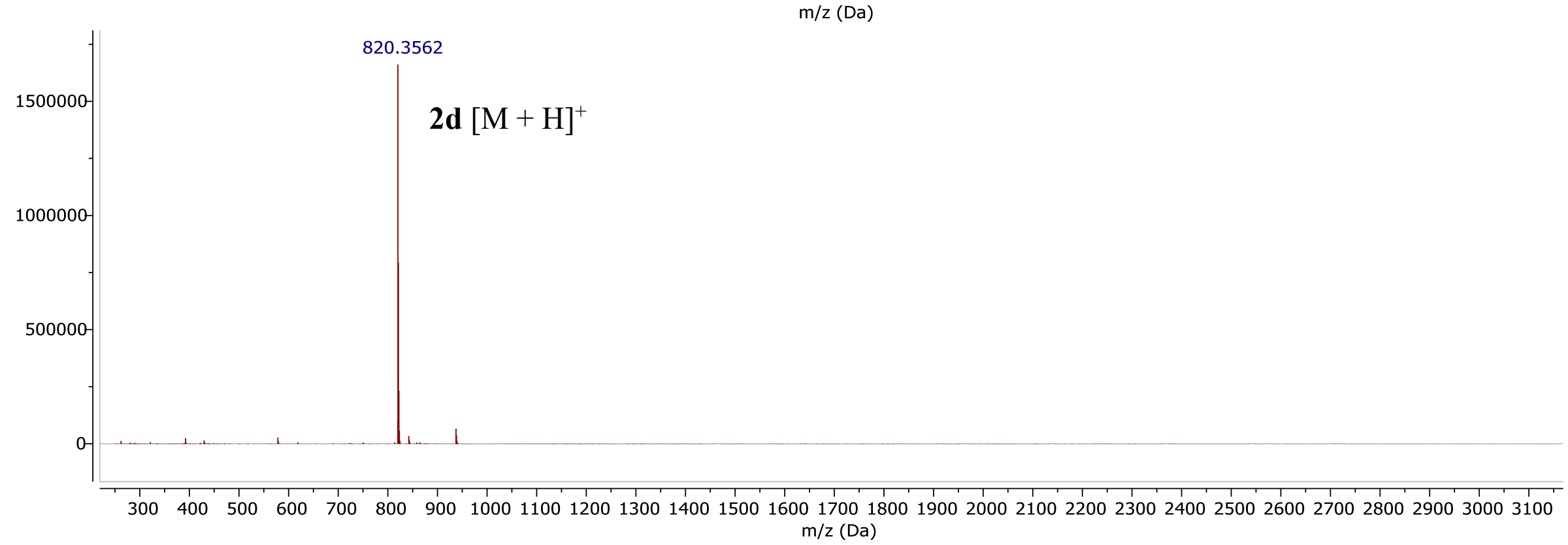

XXI.a.6. Orthogonal Modification of Free Cys in Peptide **1j"** in the Mixture of **1j"**, **2f** and **2d**.

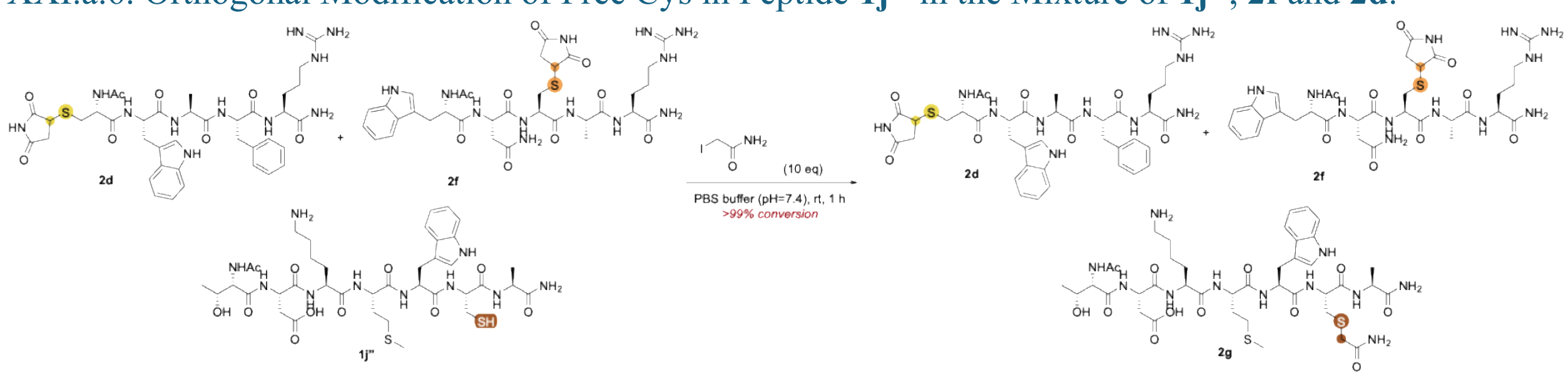

In a 1 dram vial equipped with a magnetic stirrer was added the purified peptide mixture Ac-C(maleimide)WAFR-CONH$_2$ **2d**, Ac-WNC(maleimide)AR-CONH$_2$ **2f** and Ac-TDKMWCA-CONH$_2$ **1j"** were dissolved in PBS buffer (pH=7.4) (300 µL) while stirring. In a separate 1 dram vial, iodoacetamide (2.2 mg, 0.012 mmol, 10 eq) was taken and dissolved in PBS buffer (100 µL) and the solution was added dropwise to the peptide solution and resulting mixture was allowed to stir 1 h at room temperature. After that, the selective iodoacetamide adduct peptide **2g** (pale white solid, 2.8 mg, >99% conversion) was determined via analytical HPLC using 0.1% formic acid (FA) in both mobile phases A and B. Following analysis, product was subsequently purified using this same method.

Peptide Ac-TDKMWC(iodoacetamide)A-CONH$_2$ 2g: **HRMS (ESI)** m/z: $C_{40}H_{62}N_{11}O_{12}S_2$ [M+H]$^+$ Calcd for 952.4015, Found 952.3995. **Peptide Ac-WNC(maleimide)AR-CONH$_2$ 2f**: **HRMS (ESI)** m/z: $C_{33}H_{47}N_{12}O_9S$ [M+H]$^+$ Calcd for 787.3304, Found 787.3276. **Peptide Ac-C(maleimide)WAFR-CONH$_2$ 2d**: **HRMS (ESI)** m/z: $C_{38}H_{50}N_{11}O_8S$ [M+H]$^+$ Calcd for 820.3559, Found 820.3562.

Analytical HPLC Method (C18, Water (A)/ACN (B); gradient: 5 to 85% B (0.1% formic acid in ACN) in 20 min, flow rate = 1.0 mL/min, I = 220 nm)**:** retention time of **2g**, **2d** and **2f** are 8.82, 10.22 and 8.30 min respectively.

*Analytical HPLC trace of the crude reaction mixture containing Ac-C(maleimide)WAFR-CONH$_2$ **2d**, Ac-WNC(maleimide)AR-CONH$_2$ **2f** and Ac-TDKMWC(iodoacetamide)A-CONH$_2$ **2g***

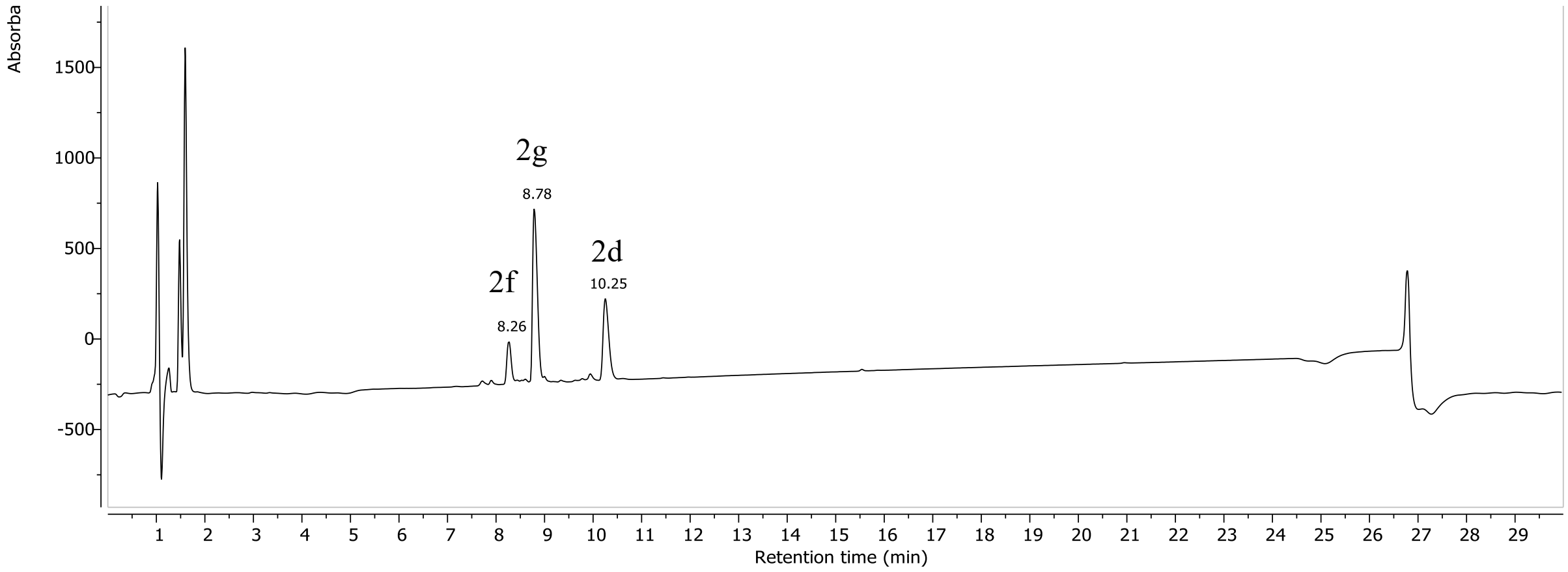

*Analytical HPLC trace of purified peptide mixture of Ac-C(maleimide)WAFR-CONH$_2$ **2d**, Ac-WNC(maleimide)AR-CONH$_2$ **2f** and Ac-TDKMWC(iodoacetamide)A-CONH$_2$ **2g***

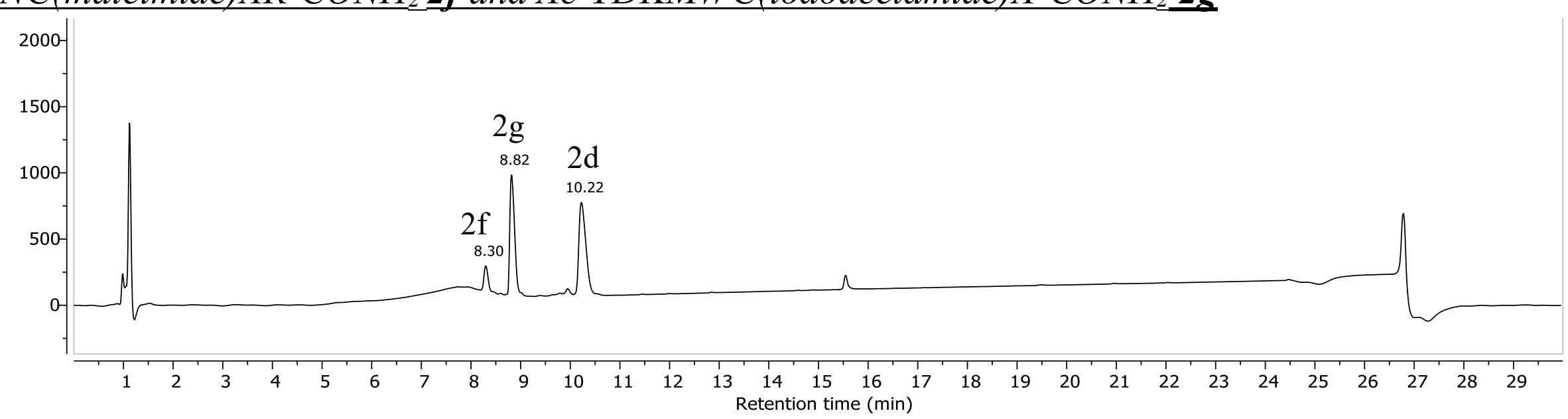

*HRMS spectrum of purified peptide mixture of Ac-C(maleimide)WAFR-CONH$_2$ **2d**, Ac-WNC(maleimide)AR-CONH$_2$ **2f** and Ac-TDKMWC(iodoacetamide)A-CONH$_2$ **2g***

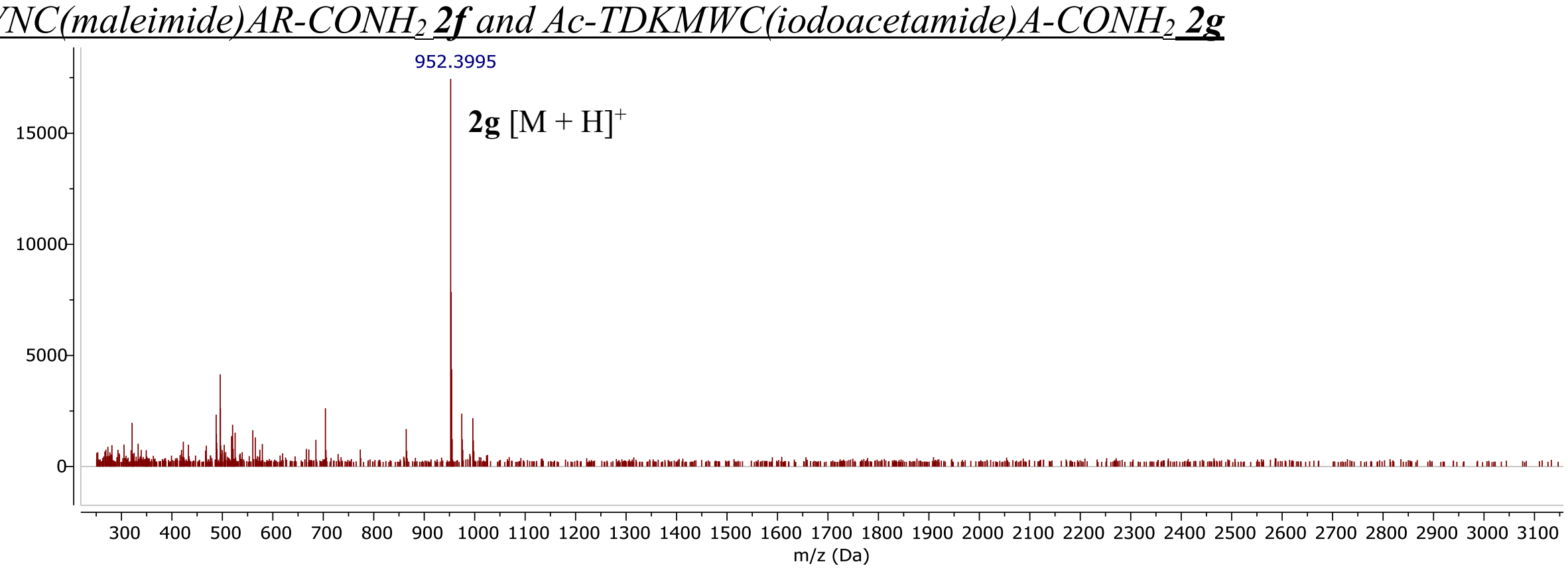

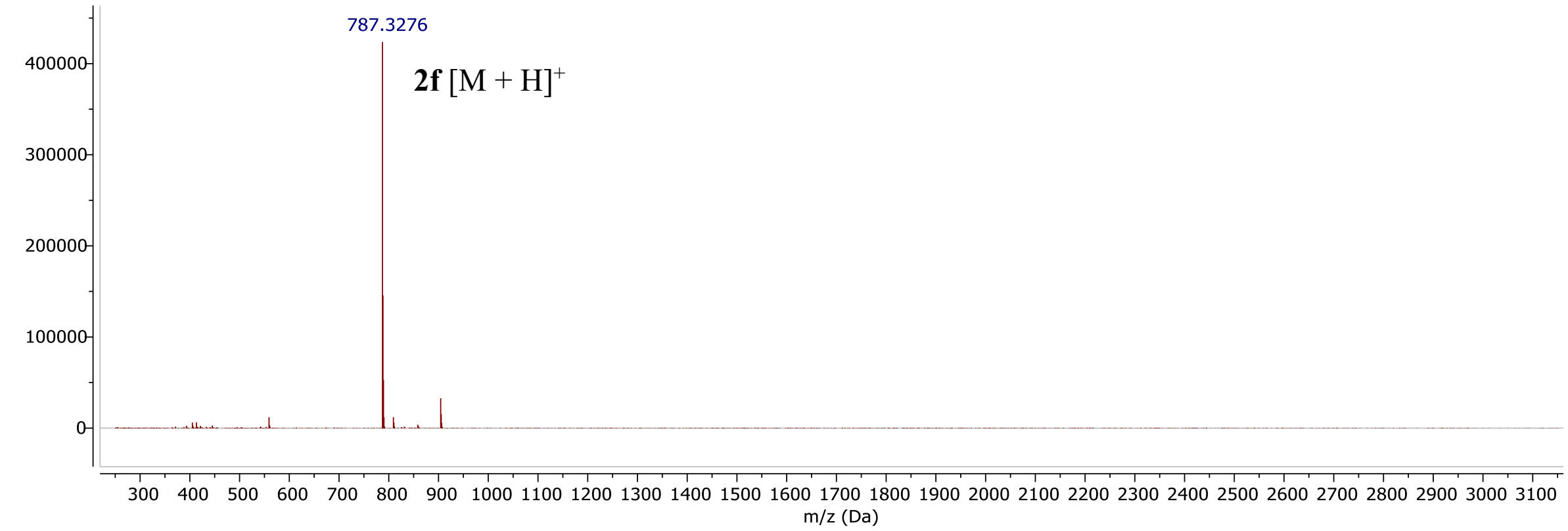

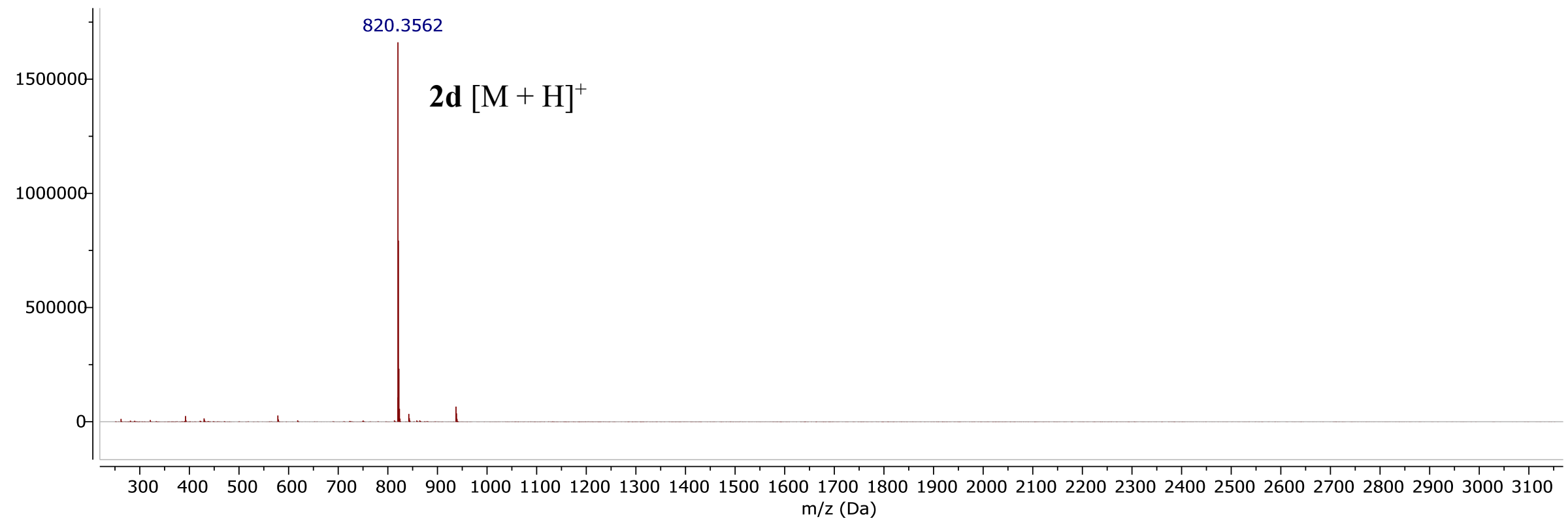

XXI.b.1 Supplementary Figure 13. Phosphite Mediated Orthogonal Cleavage and Modification of Aryl, Ethyl and *tert*-Butyl Containing Disulfides on Fully Unprotected Peptide mixture of **1g**, **1h** and **1j**.

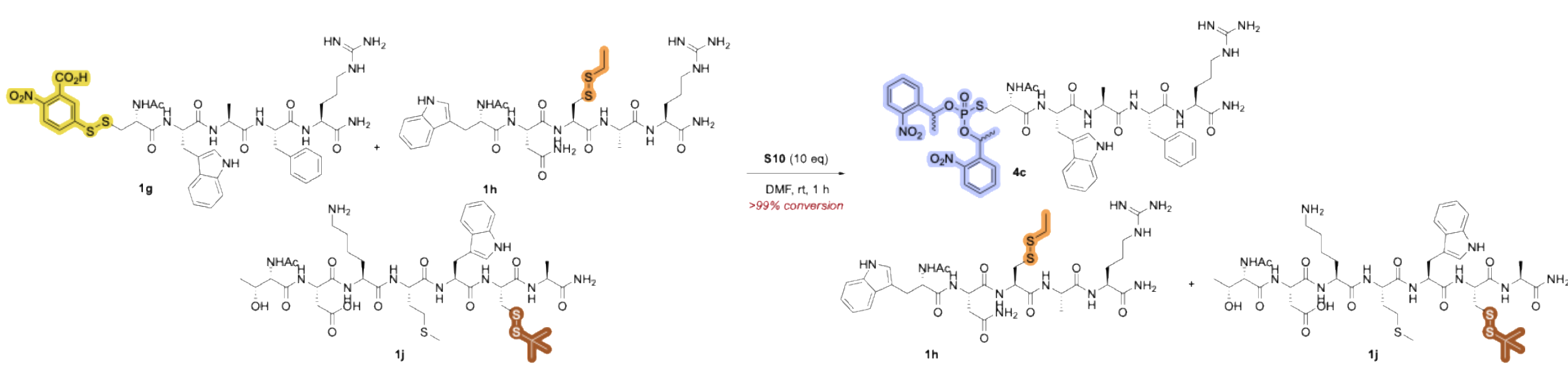

In a 1 dram vial equipped with a magnetic stirrer were added the purified peptide Ac-C(SAr[2])WAFR-$CONH_2$ **1g** (1 mg, 0.0011 mmol, 1 eq), Ac-WNC(SEt)AR-$CONH_2$ **1h** (1 mg, 0.0013 mmol, 1 eq) and Ac-TDKMWC(S^{t}Bu)A-$CONH_2$ **1j** (1 mg, 0.0010 mmol, 1 eq) were dissolved in 400 µL DMF while stirring. In a separate 1 dram vial, tris(1-(2-nitrophenyl)ethyl) phosphite **S10** (16 mg, 0.029 mmol, 10 eq) was taken and dissolved in DMF (100 µL) and the solution was added dropwise to the peptide solution and resulting mixture was allowed to stir 1 h at room temperature. After that, the selective phosphite modified product Ac-C(P(O)(OAr[1])$_2$)WAFR-$CONH_2$ **4c** (pale yellow solid, 3.1 mg, >99% conversion) was determined via analytical HPLC using 0.1% formic acid (FA) in both mobile phases A and B. Following analysis, product was subsequently purified using this same method. Purified peptides **1g**, **1h**, and **1j** have the retention times in the mixture at 11.94, 9.98 and 11.54 respectively.

Peptide Ac-C(P(O)(OAr[1])$_2$)WAFR-$CONH_2$ 4c: **HRMS (ESI)** m/z: $C_{50}H_{62}N_{12}O_{13}PS$ $[M+H]^+$ Calcd for 1101.4012, Found 1101.4019. **Peptide Ac-WNC(SEt)AR-$CONH_2$ 1h: HRMS (ESI)** m/z: $C_{31}H_{48}N_{11}O_7S_2$

[M+H]$^+$ Calcd for 750.3174, Found 750.3156. **Peptide Ac-TDKMWC(S^tBu)A-CONH$_2$ 1j: HRMS (ESI)** m/z: $C_{42}H_{67}N_{10}O_{11}S_3$ [M+H]$^+$ Calcd for 983.4147, Found 983.4130; $C_{42}H_{66}N_{10}NaO_{11}S_3$ [M + Na]$^+$ Calcd for 1005.3967, Found 1005.3954.

Analytical HPLC Method (C18, Water (A)/ACN (B); gradient: 5 to 85% B (0.1% formic acid in ACN) in 20 min, flow rate = 1.0 mL/min, I = 220 nm)**:** retention time of **4c**, **1h** and **1j** are 15.0, 10.16 and 11.7 min respectively.

*Analytical HPLC trace of tris(1-(2-nitrophenyl)ethyl) phosphite **S10** in DMF*

(* = side products from **S10**)

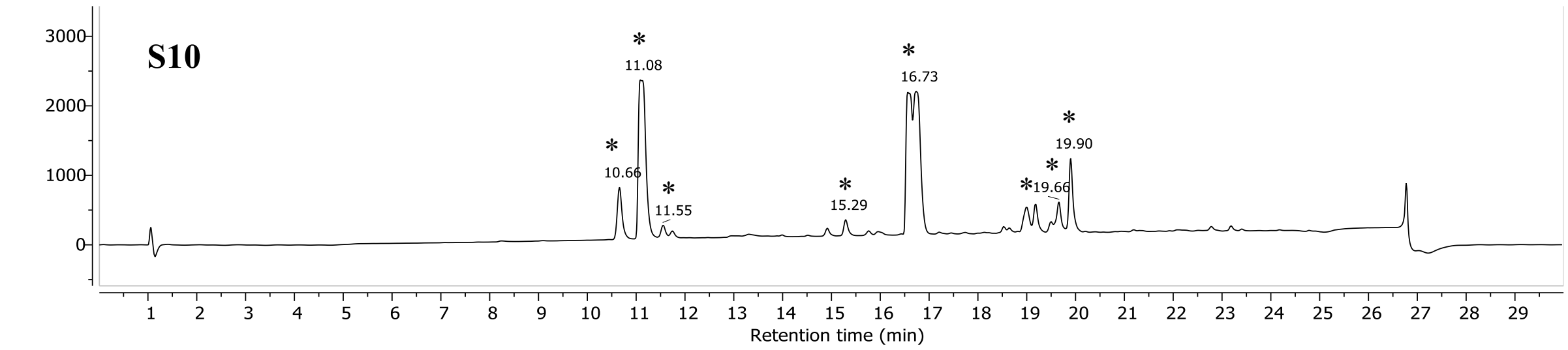

*Analytical HPLC trace of the crude reaction mixture containing Ac-WNC(SEt)AR-CONH$_2$ **1h**, Ac-C(P(O)(OAr^1)$_2$)WAFR-CONH$_2$ **4c** and Ac-TDKMWC(S^tBu)A-CONH$_2$ **1j***

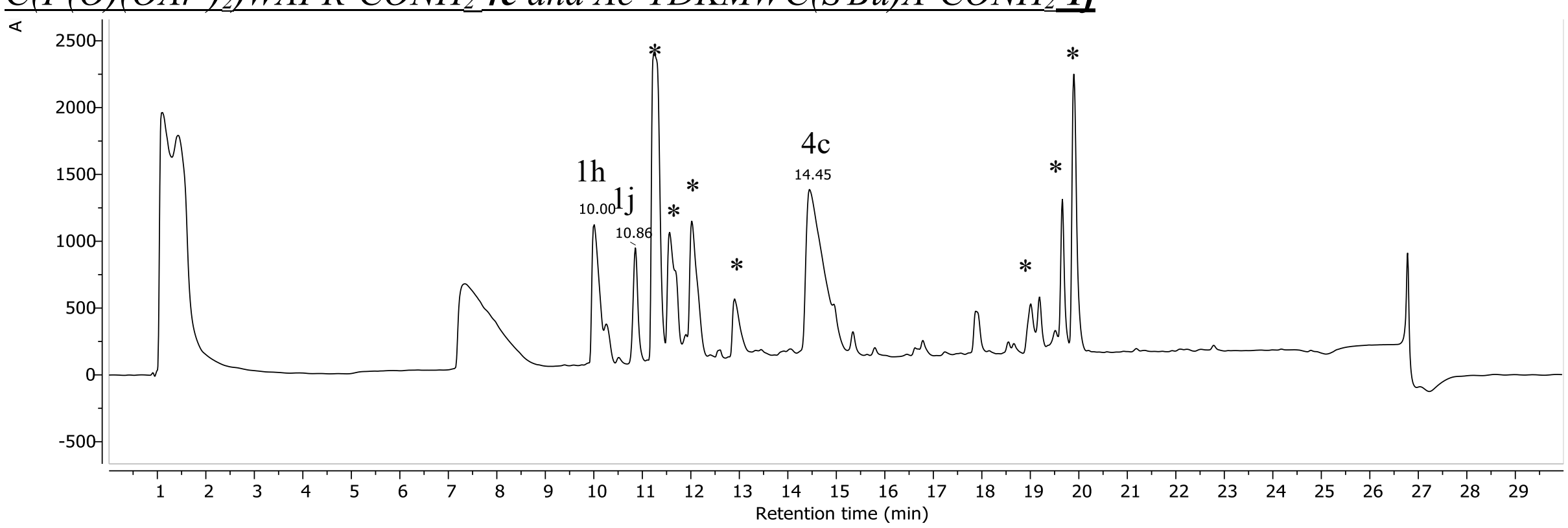

(* = side products from **S10**)

*Analytical HPLC trace of purified peptide mixture containing Ac-WNC(SEt)AR-CONH$_2$ **1h**, Ac-C(P(O)(OAr^1)$_2$)WAFR-CONH$_2$ **4c** and Ac-TDKMWC(S^tBu)A-CONH$_2$ **1j***

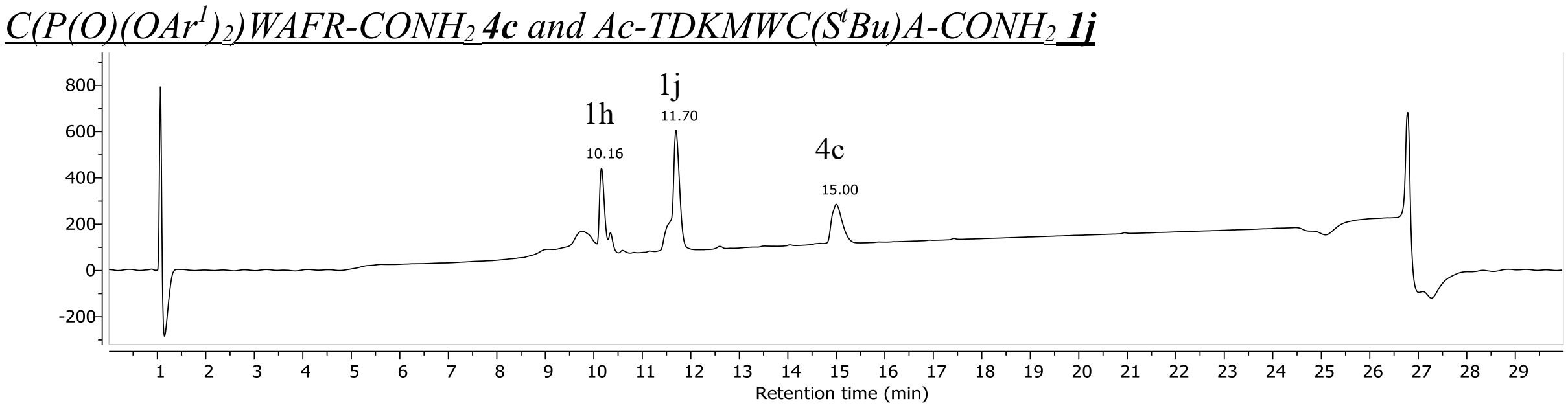

*HRMS spectrum of purified peptide peptide mixture containing Ac-WNC(SEt)AR-CONH$_2$ **1h**, Ac-C(P(O)(OAr^1)$_2$)WAFR-CONH$_2$ **4c** and Ac-TDKMWC(S^{t}Bu)A-CONH$_2$ **1j***

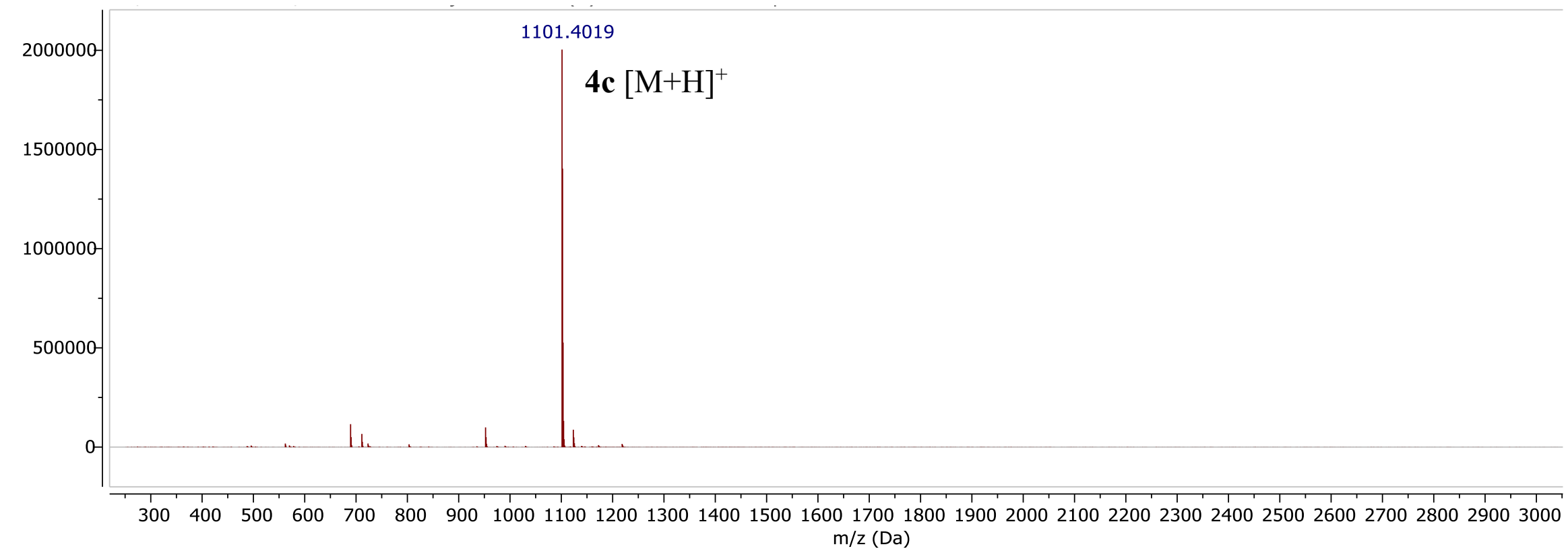

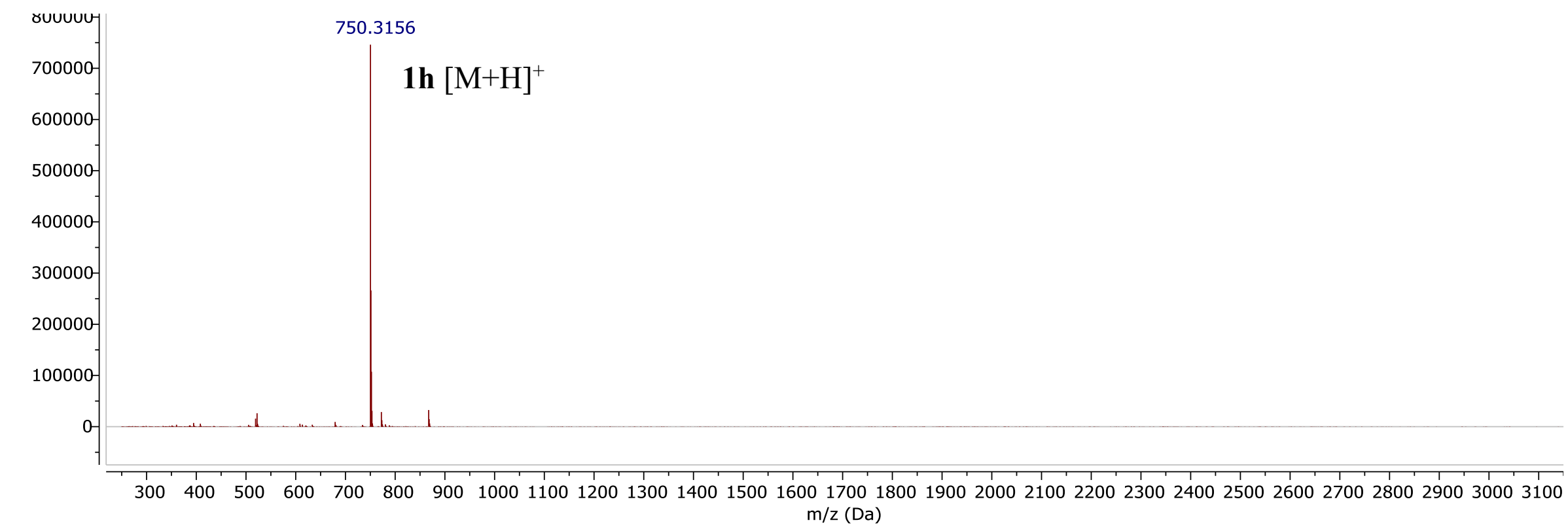

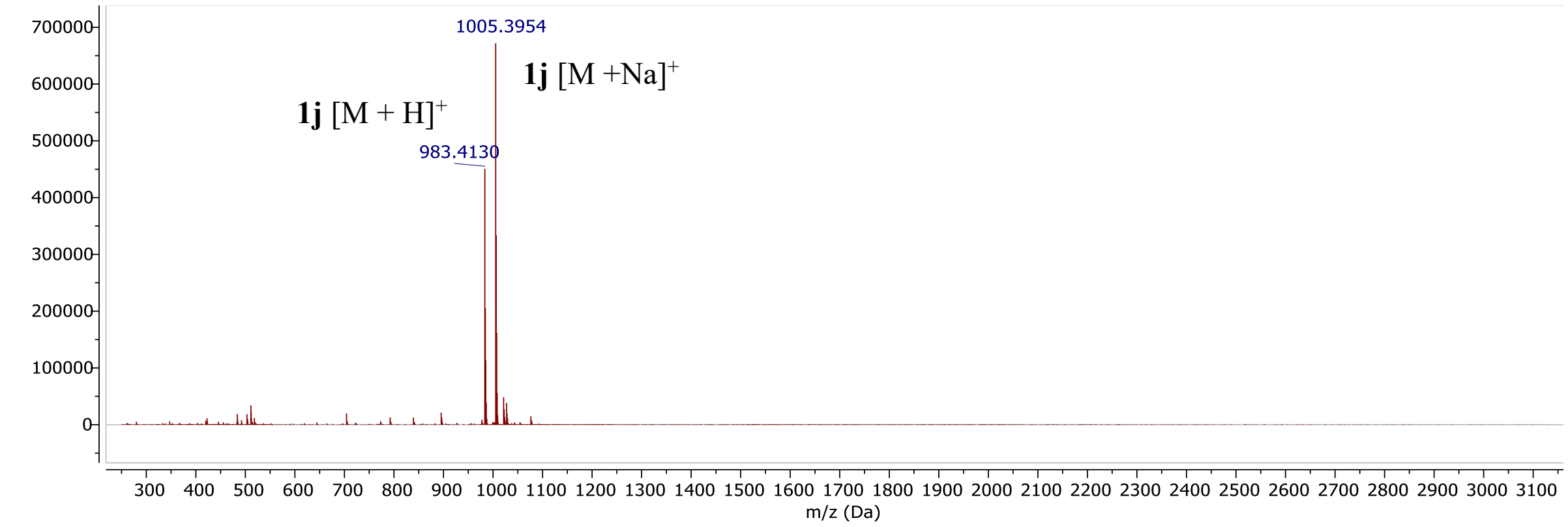

XXI.b.2. Orthogonal Cleavage of Ethyl-Containing Disulfide on **1h** from the Mixture **4c**, **1h** and **1j**.

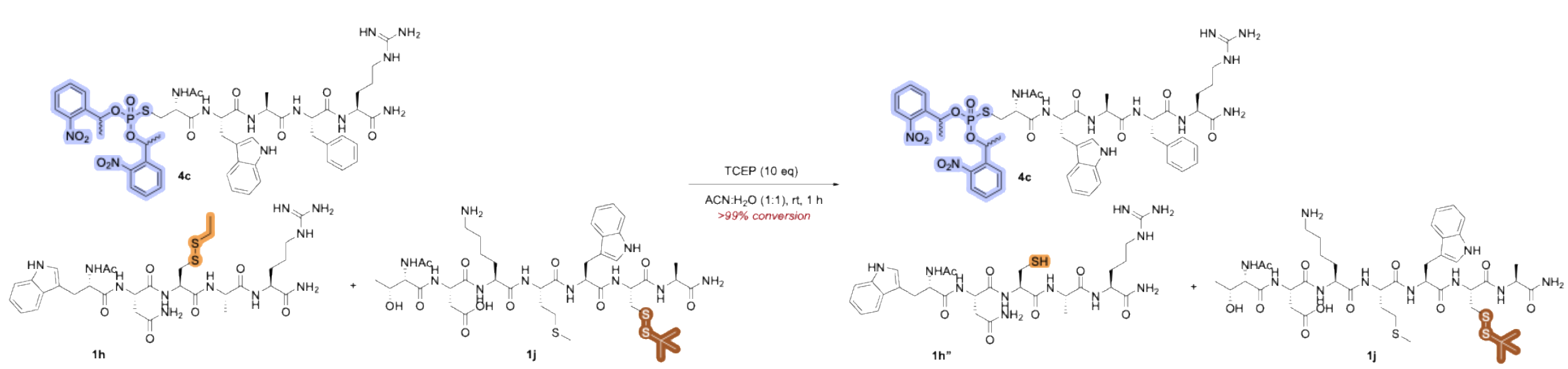

In a 1 dram vial equipped with a magnetic stirrer were added the purified peptide mixture Ac-C(P(O)(OAr^1)$_2$)WAFR-CONH$_2$ **4c**, Ac-WNC(SEt)AR-CONH$_2$ **1h** and Ac-TDKMWC(S^{t}Bu)A-CONH$_2$ **1j**

were dissolved in ACN (300 µL), and H_2O (200 µL) while stirring. In a separate 1 dram vial, TCEP (3.7 mg, 0.013 mmol, 10 eq) was taken and dissolved in H_2O (100 µL) and the solution was added dropwise to the peptide solution and resulting mixture was allowed to stir 1 h at room temperature. After that, the selective reduced thiol product **1h''** (>99% conversion) was determined via analytical HPLC using 0.1% formic acid (FA) in both mobile phases A and B. Following analysis, solvent was evaporated in speedvac and used for the next step without purification.

XXI.b.3. Orthogonal Modification of Free Cys on **1h''** from the Mixture **4c**, **1h''** and **1j**.

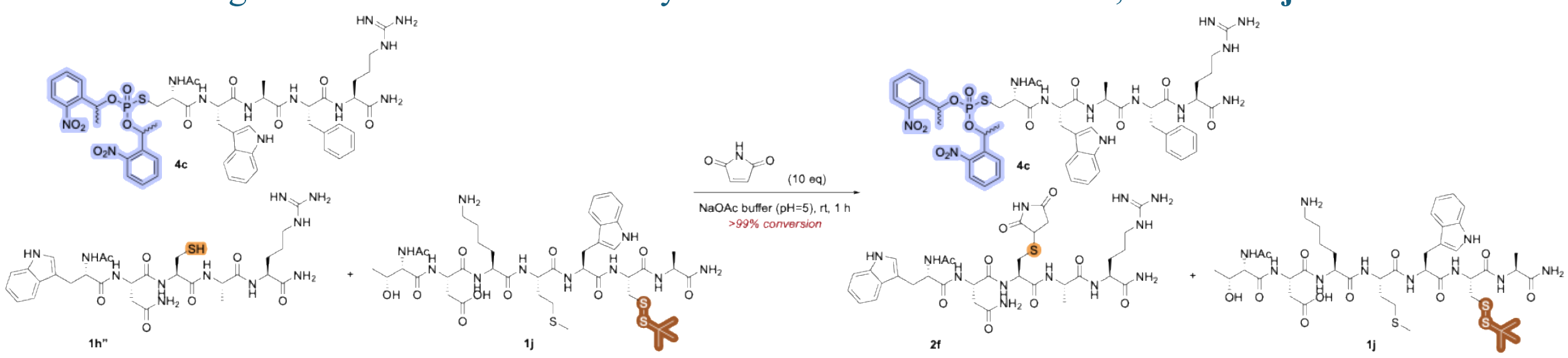

In a 1 dram vial equipped with a magnetic stirrer were added the peptide mixture Ac-C(P(O)(OAr[1])$_2$)WAFR-CONH$_2$ **4c**, Ac-WNCAR-CONH$_2$ **1h''** and Ac-TDKMWC(S^{t}Bu)A-CONH$_2$ **1j** were dissolved in 300 µL of sodium acetate buffer (NaOAc buffer pH = 5) while stirring. In a separate 1 dram vial, maleimide (1.2 mg, 0.012 mmol, 10 eq) was taken and dissolved in NaOAc buffer (100 µL) and the solution was added dropwise to the peptide solution and resulting mixture was allowed to stir 1 h at room temperature. After that, the selective maleimide adduct peptide **2f** (pale yellow solid, 3.2 mg, >99% conversion) was determined via analytical HPLC using 0.1% formic acid (FA) in both mobile phases A and B. Following analysis, products were subsequently purified using this same method.

Peptide Ac-WNC(maleimide)AR-CONH$_2$ 2f: **HRMS (ESI)** m/z: $C_{33}H_{47}N_{12}O_9S$ [M+H]$^+$ Calcd for 787.3304, Found 787.3307. **Peptide Ac-C(P(O)(OAr[1])$_2$)WAFR-CONH$_2$ 4c**: **HRMS (ESI)** m/z: $C_{50}H_{62}N_{12}O_{13}PS$ [M+H]$^+$ Calcd for 1101.4012, Found 1101.4019. **Peptide Ac-TDKMWC(S^{t}Bu)A-CONH$_2$ 1j: HRMS (ESI)** m/z: $C_{42}H_{67}N_{10}O_{11}S_3$ [M+H]$^+$ Calcd for 983.4147, Found 983.4130; $C_{42}H_{66}N_{10}NaO_{11}S_3$ [M+Na]$^+$ Calcd for 1005.3967, Found 1005.3954.

Analytical HPLC Method (C18, Water (A)/ACN (B); gradient: 5 to 85% B (0.1% formic acid in ACN) in 20 min, flow rate = 1.0 mL/min, I = 220 nm)**:** retention time of **4c**, **2f** and **1j** are 15.09, 8.30 and 11.6 min respectively.

*Analytical HPLC trace of the crude reaction mixture containing Ac-WNC(maleimide)AR-CONH$_2$ **2f**, Ac-C(P(O)(OAr[1])$_2$)WAFR-CONH$_2$ **4c** and Ac-TDKMWC(S^{t}Bu)A-CONH$_2$ **1j***

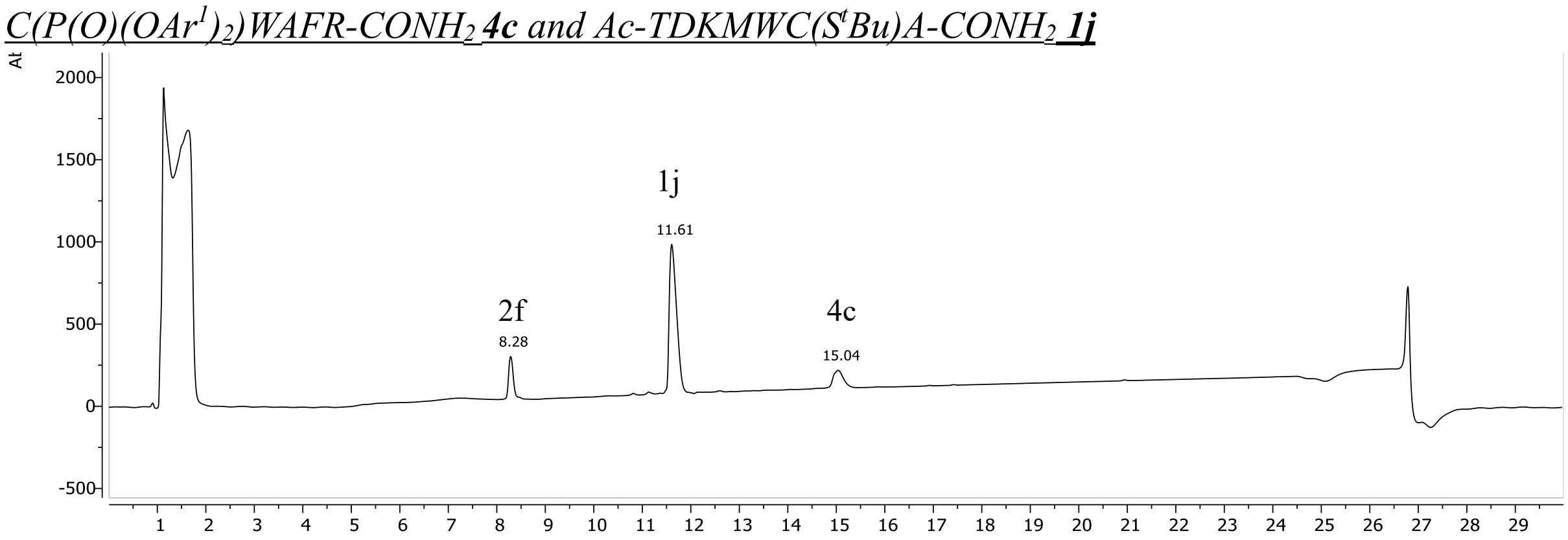

Analytical HPLC trace of purified peptide mixture containing Ac-WNC(maleimide)AR-CONH$_2$ ***2f****, Ac-C(P(O)(OAr^1)$_2$)WAFR-CONH$_2$* ***4c*** *and Ac-TDKMWC(S^{t}Bu)A-CONH$_2$* ***1j***

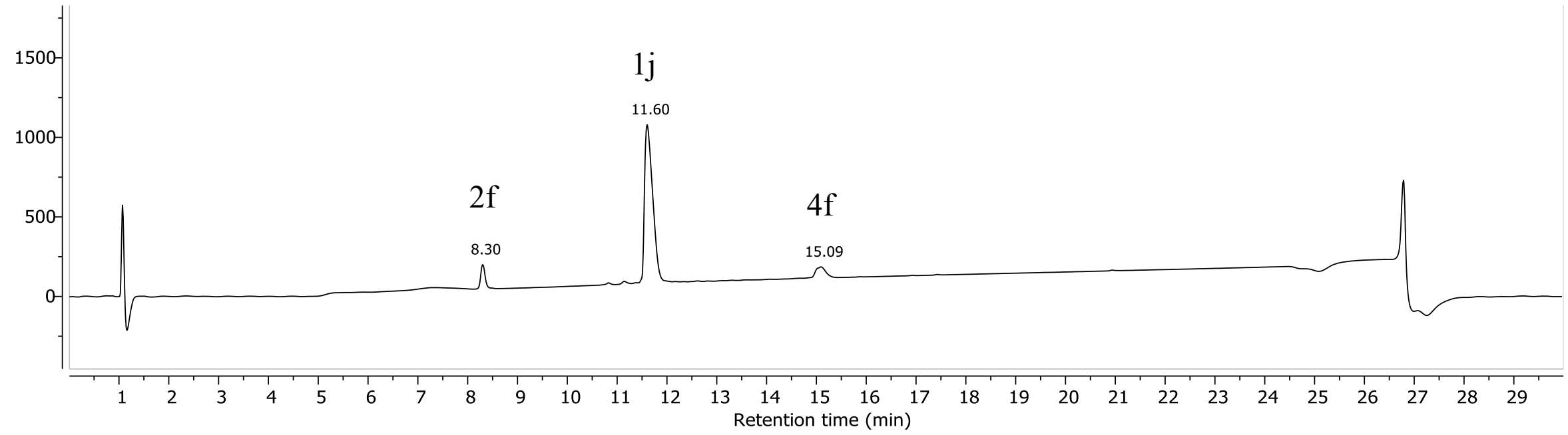

HRMS spectrum of purified peptide peptide mixture containing Ac-WNC(maleimide)AR-CONH$_2$ ***2f****, Ac-C(P(O)(OAr^1)$_2$)WAFR-CONH$_2$* ***4c*** *and Ac-TDKMWC(S^{t}Bu)A-CONH$_2$* ***1j***

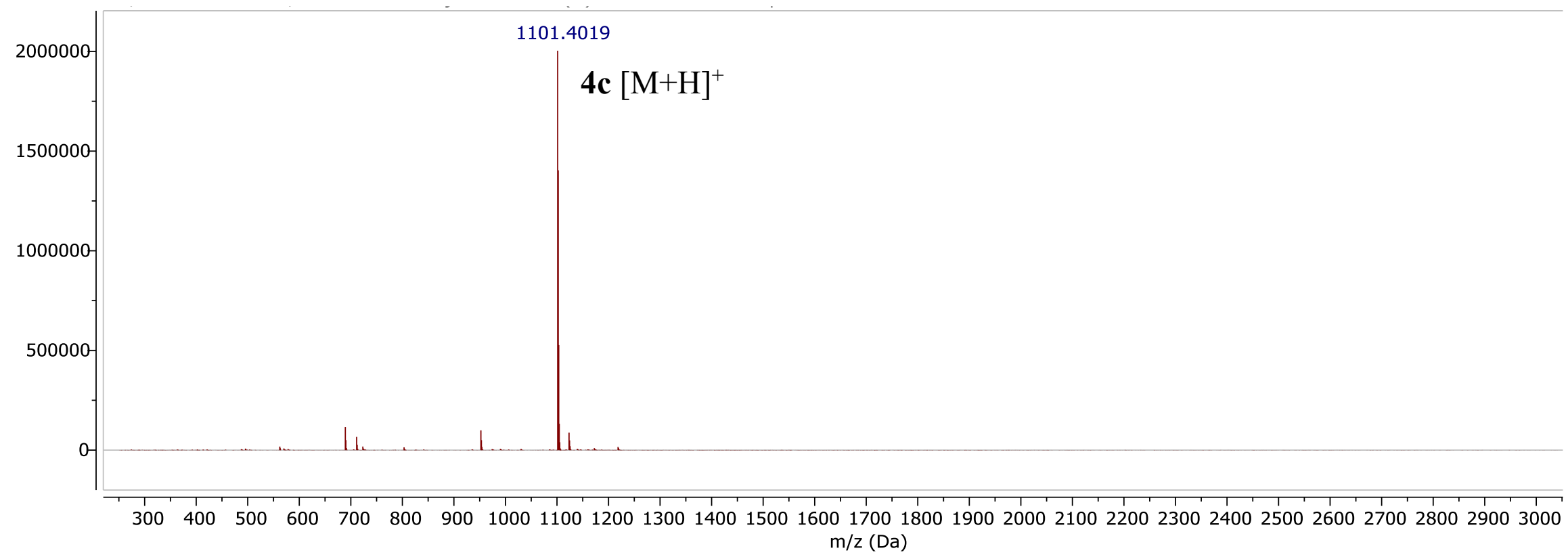

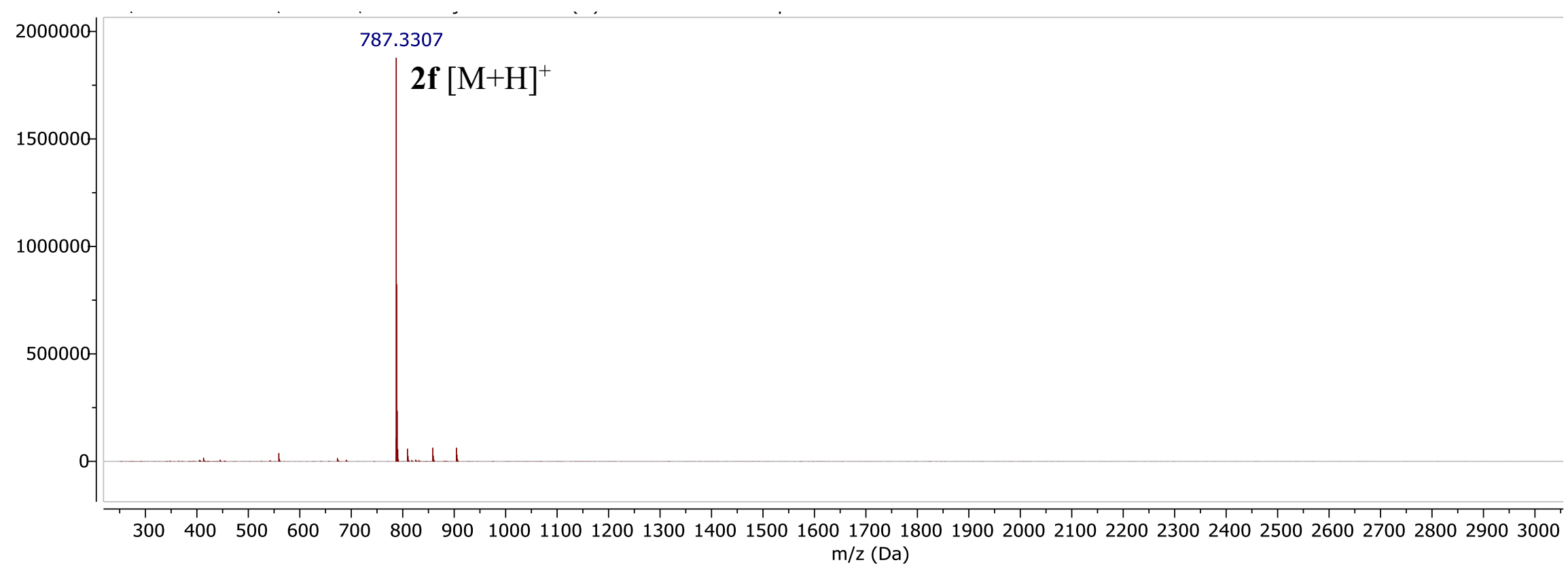

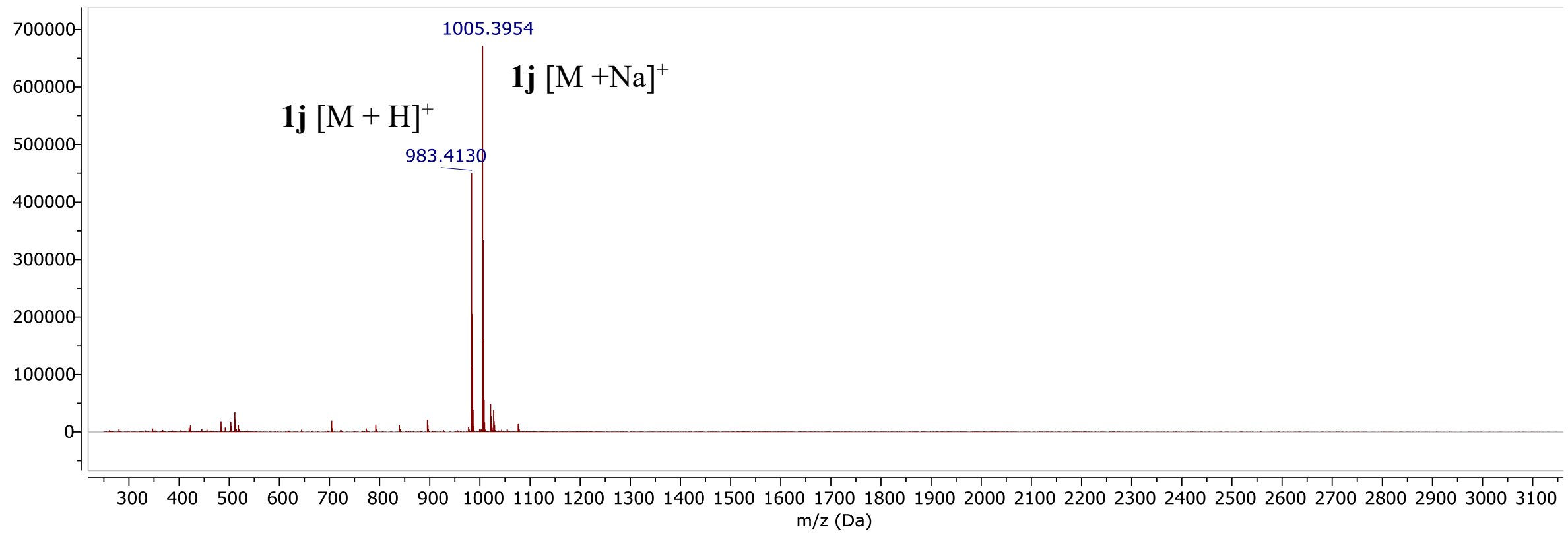

XXI.b.4. Orthogonal Cleavage of *tert*-Butyl Containing Disulfide on **1j** in the Mixture of Peptides **2f**, **1j** and **4c**.

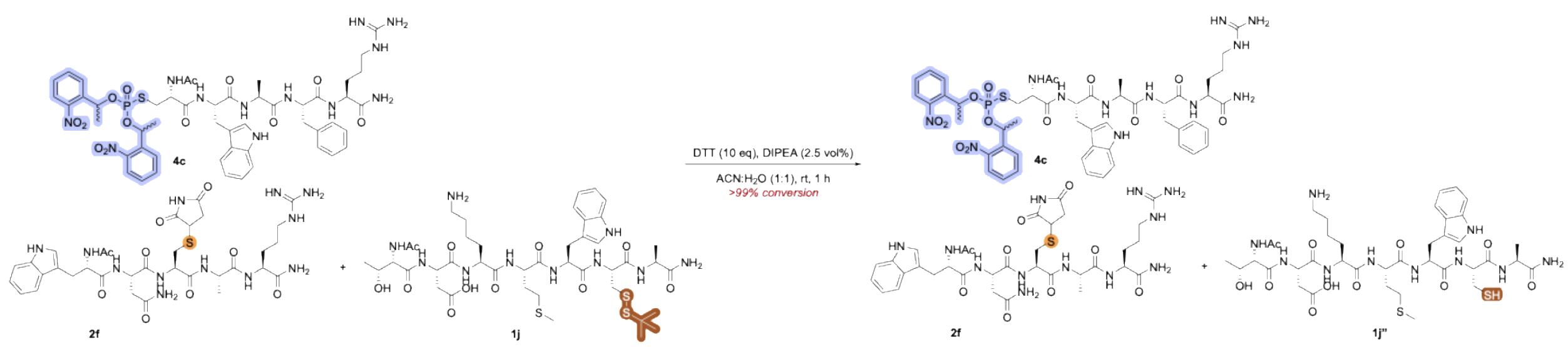

In a 1 dram vial equipped with a magnetic stirrer were added the purified peptide mixture Ac-C(P(O)(OAr[1])$_2$)WAFR-CONH$_2$ **4c**, Ac-WNC(maleimide)AR-CONH$_2$ **2f** and Ac-TDKMWC(S^{t}Bu)A-CONH$_2$ **1j** were dissolved in ACN (500 µL), and H_2O (200 µL) while stirring. In a separate 1 dram vial, dithiothreitol (DTT, 1.6 mg, 0.01 mmol, 10 eq) was taken and dissolved in H_2O (300 µL) and the solution was added dropwise to the peptide solution followed by addition of *N,N*-diisopropylethylamine (DIPEA, 25 µL) at once and resulting mixture was allowed to stir 1 h at room temperature. After that, the selective reduced thiol product **1j"** (>99% conversion) was determined via analytical HPLC using 0.1% formic acid (FA) in both mobile phases A and B. Following analysis, solvent was evaporated in speedvac and used for the next step without purification.

XXI.b.5. Orthogonal Modification of free Cys in Peptide **1j"** in the Mixture of **1j"**, **2f** and **4c**.

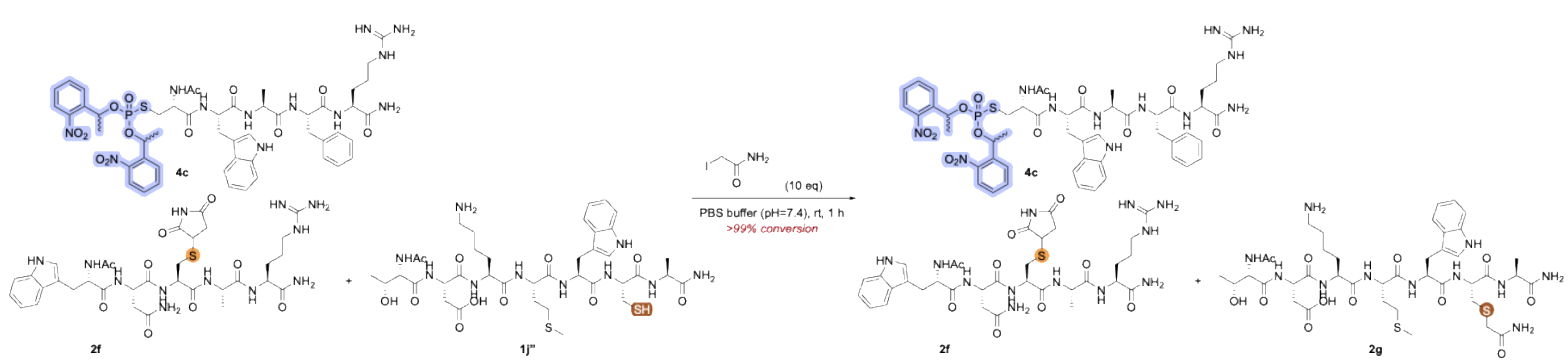

In a 1 dram vial equipped with a magnetic stirrer were added the peptide mixture Ac-C(P(O)(OAr[1])$_2$)WAFR-CONH$_2$ **4c**, Ac-WNC(maleimide)AR-CONH$_2$ **2f** and Ac-TDKMWCA-CONH$_2$ **1j"** were dissolved in PBS buffer (pH=7.4) (300 µL) while stirring. In a separate 1 dram vial, iodoacetamide (2.2 mg, 0.012 mmol, 10 eq) was taken and dissolved in PBS buffer (100 µL) and the solution was added

dropwise to the peptide solution and resulting mixture was allowed to stir 1 h at room temperature. After that, the selective iodoacetamide adduct peptide **2g** (pale yellow solid, 3.2 mg, >99% conversion) was determined via analytical HPLC using 0.1% formic acid (FA) in both mobile phases A and B. Following analysis, product was subsequently purified using this same method.

Peptide Ac-TDKMWC(iodoacetamide)A-CONH₂ 2g: **HRMS (ESI)** m/z: $C_{40}H_{62}N_{11}O_{12}S_2$ $[M+H]^+$ Calcd for 952.4015, Found 952.3991; $[M+Na]^+$ Calcd for 974.3835, Found 974.3813. **Peptide Ac-WNC(maleimide)AR-CONH₂ 2f**: **HRMS (ESI)** m/z: $C_{33}H_{47}N_{12}O_9S$ $[M+H]^+$ Calcd for 787.3304, Found 787.3307. **Peptide Ac-C(P(O)(OAr¹)₂)WAFR-CONH₂ 4c**: **HRMS (ESI)** m/z: $C_{50}H_{62}N_{12}O_{13}PS$ $[M+H]^+$ Calcd for 1101.4012, Found 1101.4019.

Analytical HPLC Method (C18, Water (A)/ACN (B); gradient: 5 to 85% B (0.1% formic acid in ACN) in 20 min, flow rate = 1.0 mL/min, I = 220 nm)**:** retention time of **2g**, **2f** and **4c** are 8.88, 8.29 and 15.04 min respectively.

*Analytical HPLC trace of the crude reaction mixture containing Ac-WNC(maleimide)AR-CONH₂ **2f**, Ac-C(P(O)(OAr¹)₂)WAFR-CONH₂ **4c** and Ac-TDKMWC(iodoacetamide)A-CONH₂ **2g***

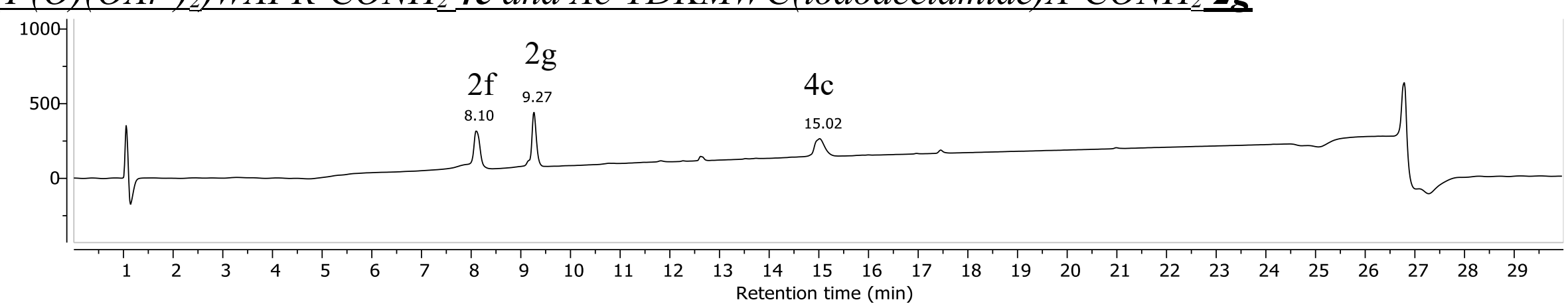

*Analytical HPLC trace of purified peptide mixture containing Ac-WNC(maleimide)AR-CONH₂ **2f**, Ac-C(P(O)(OAr¹)₂)WAFR-CONH₂ **4c** and Ac-TDKMWC(iodoacetamide)A-CONH₂ **2g***

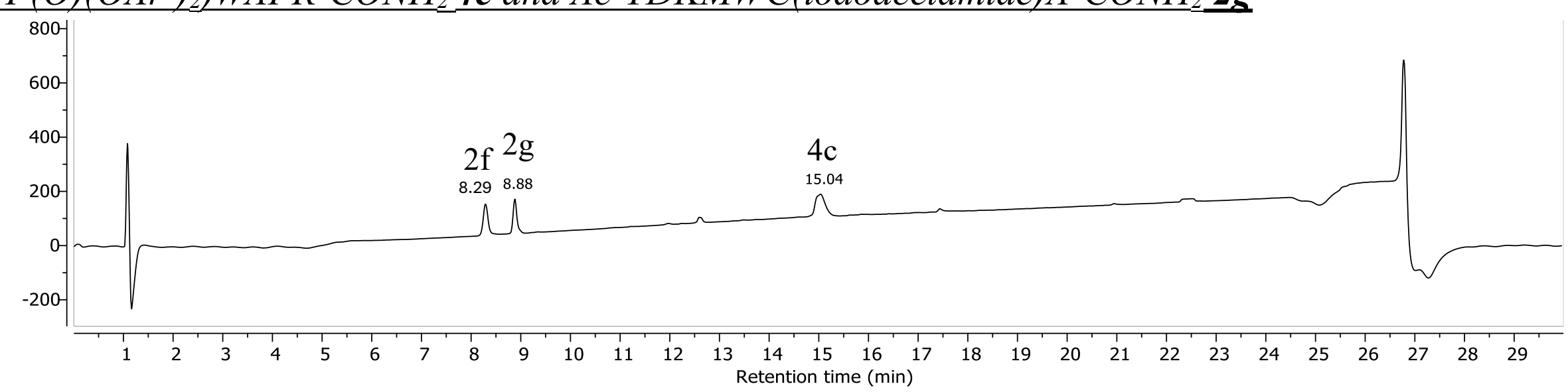

*HRMS spectrum of purified peptide peptide mixture containing Ac-WNC(maleimide)AR-CONH₂ **2f**, Ac-C(P(O)(OAr¹)₂)WAFR-CONH₂ **4c** and Ac-TDKMWC(iodoacetamide)A-CONH₂ **2g***

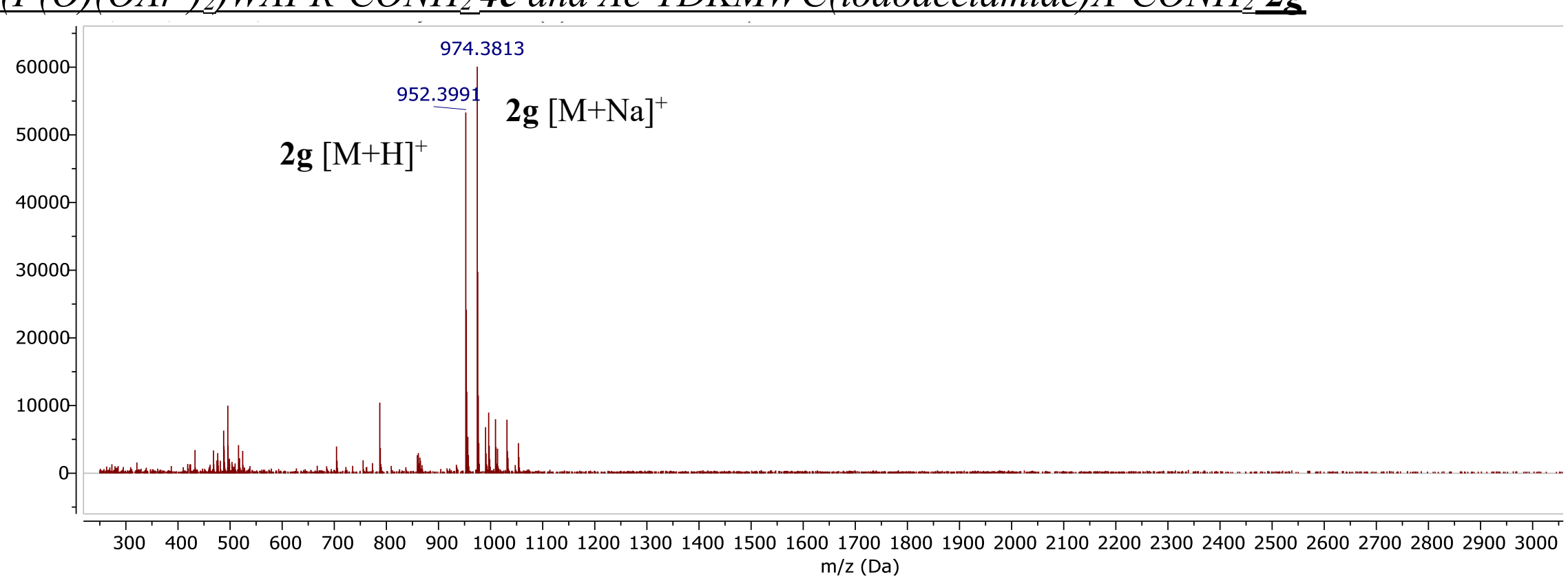

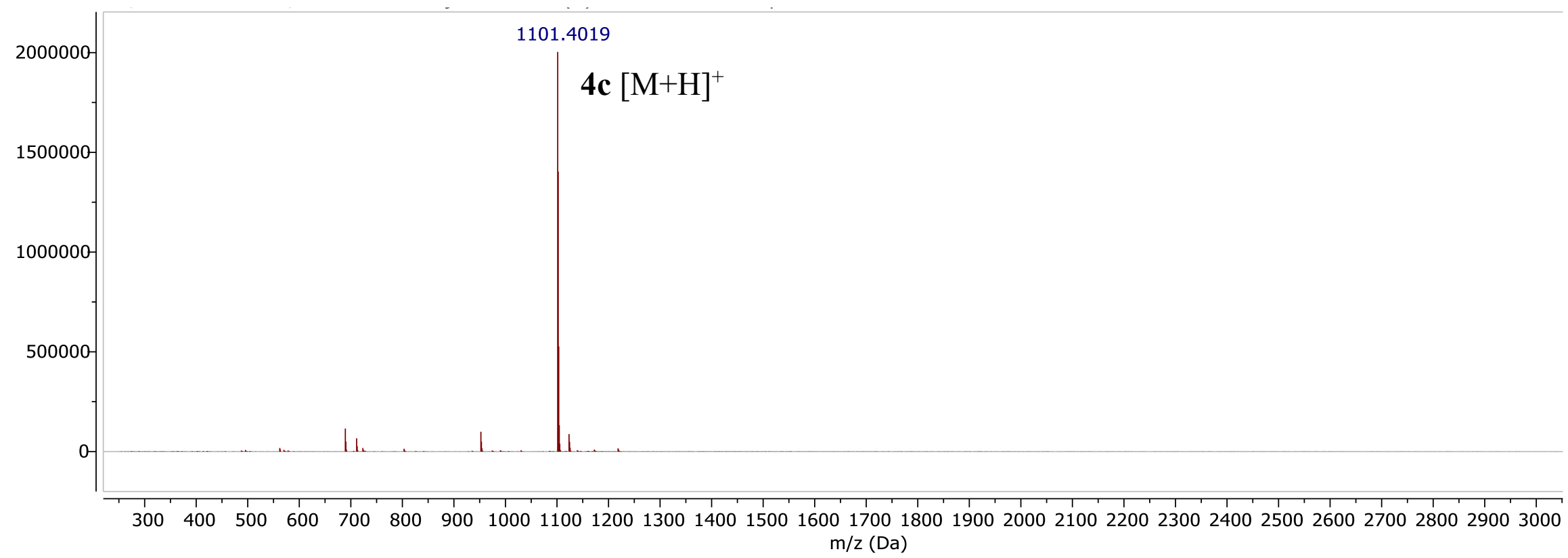

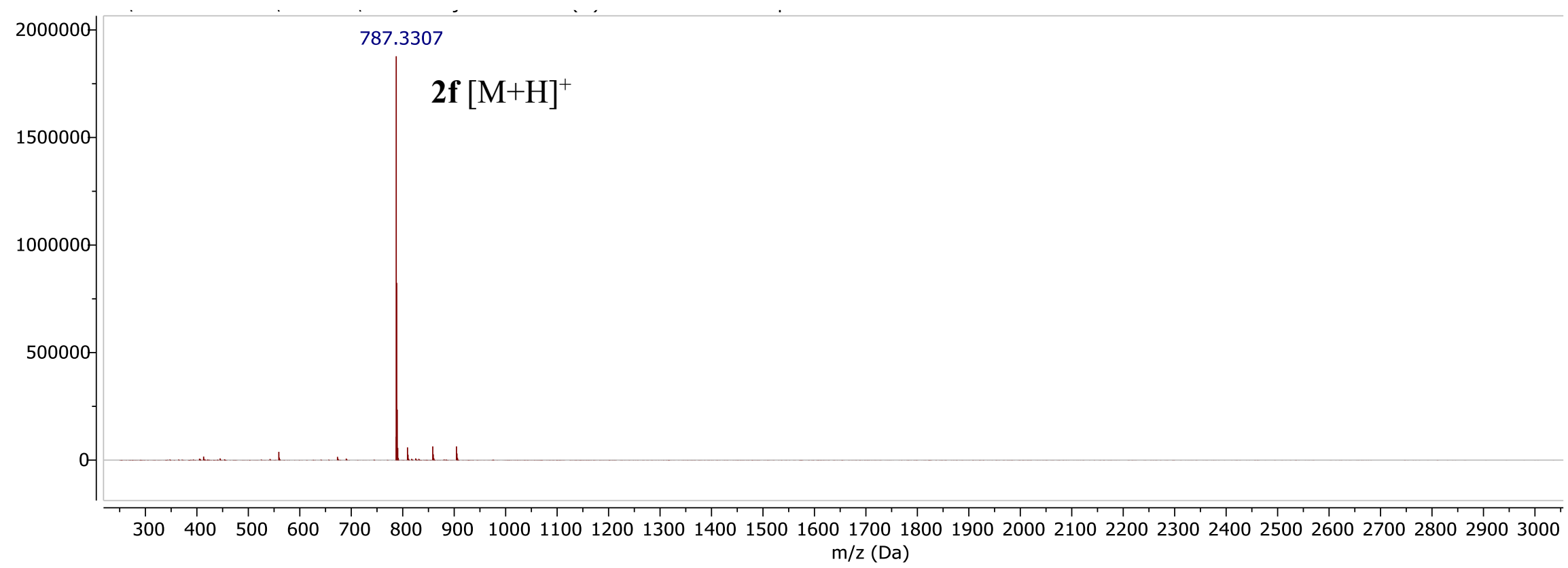

XXII. References

(1) Chalker, J. M.; Gunnoo, S. B.; Boutureira, O.; Gerstberger, S. C.; Fernández-González, M.; Bernardes, G. J. L.; Griffin, L.; Hailu, H.; Schofield, C. J.; Davis, B. G. Methods for Converting Cysteine to Dehydroalanine on Peptides and Proteins. *Chem. Sci.* **2011**, *2*, 1666–1676.
(2) Song, A.; Feng, T.; Shen, X.; Gai, S.; Zhai, Y.; Chen, H. Fluorescence Detection of Glutathione S-Transferases in a Low GSH Level Environment. *Chem. Commun.* **2019**, *55*, 7219-7222.
(3) Yang, K.; Hu, Q.; Li, Q.; Liu J.; Li, Z.-Y.; Li B.; Sun, X. Metal-Free and NBS-Mediated Direct Thiol–Disulfide Exchange Reaction to Access Unsymmetrical Disulfides. *Eur. J. Org. Chem.* **2023**, *26*, e202300394.
(4) Bertran-Vicente, J.; Serwa, R. A.; Schümann, M.; Schmieder, P.; Krause, E.; Hackenberger, C. P. R. Site-Specifically Phosphorylated Lysine Peptides. *J. Am. Chem. Soc.* **2014**, *136*, 13622-13628.

^{1}H NMR of compound **S2** in $CDCl_3$ (400 MHz)

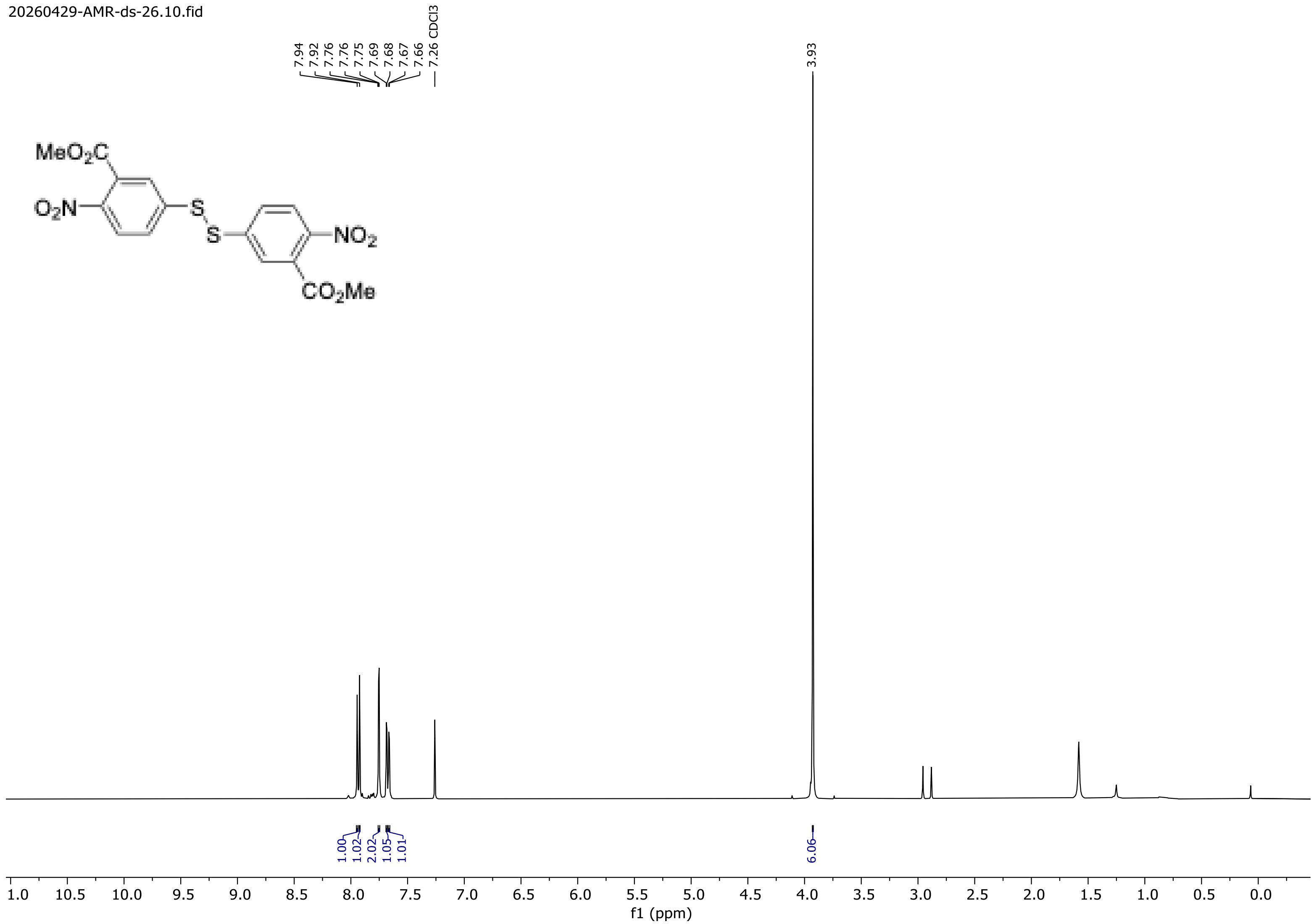

^{1}H NMR of compound **1a** in DMSO-d_6 (400 MHz)

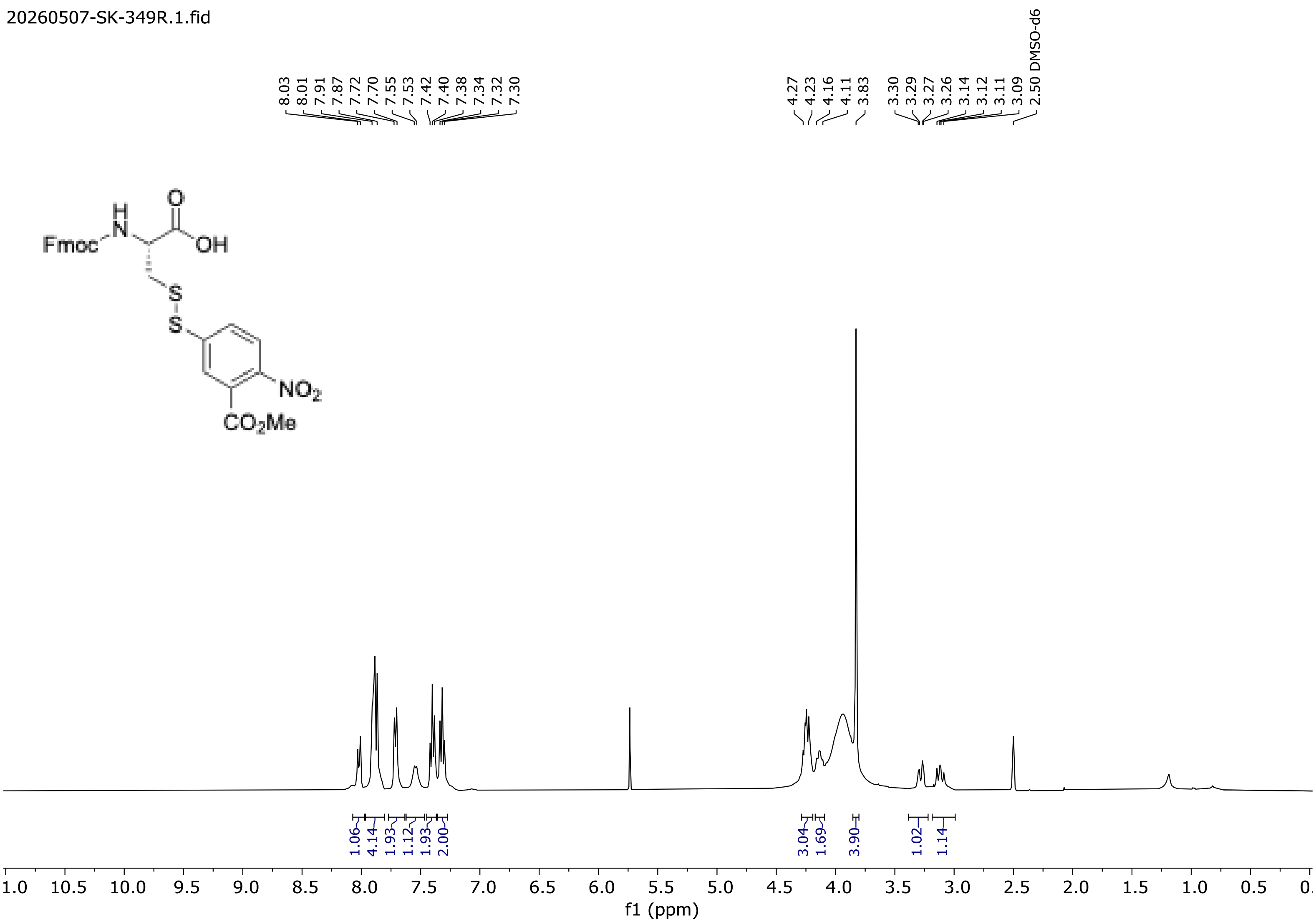

^{13}C NMR of compound **1a** in DMSO-d_6 (400 MHz)

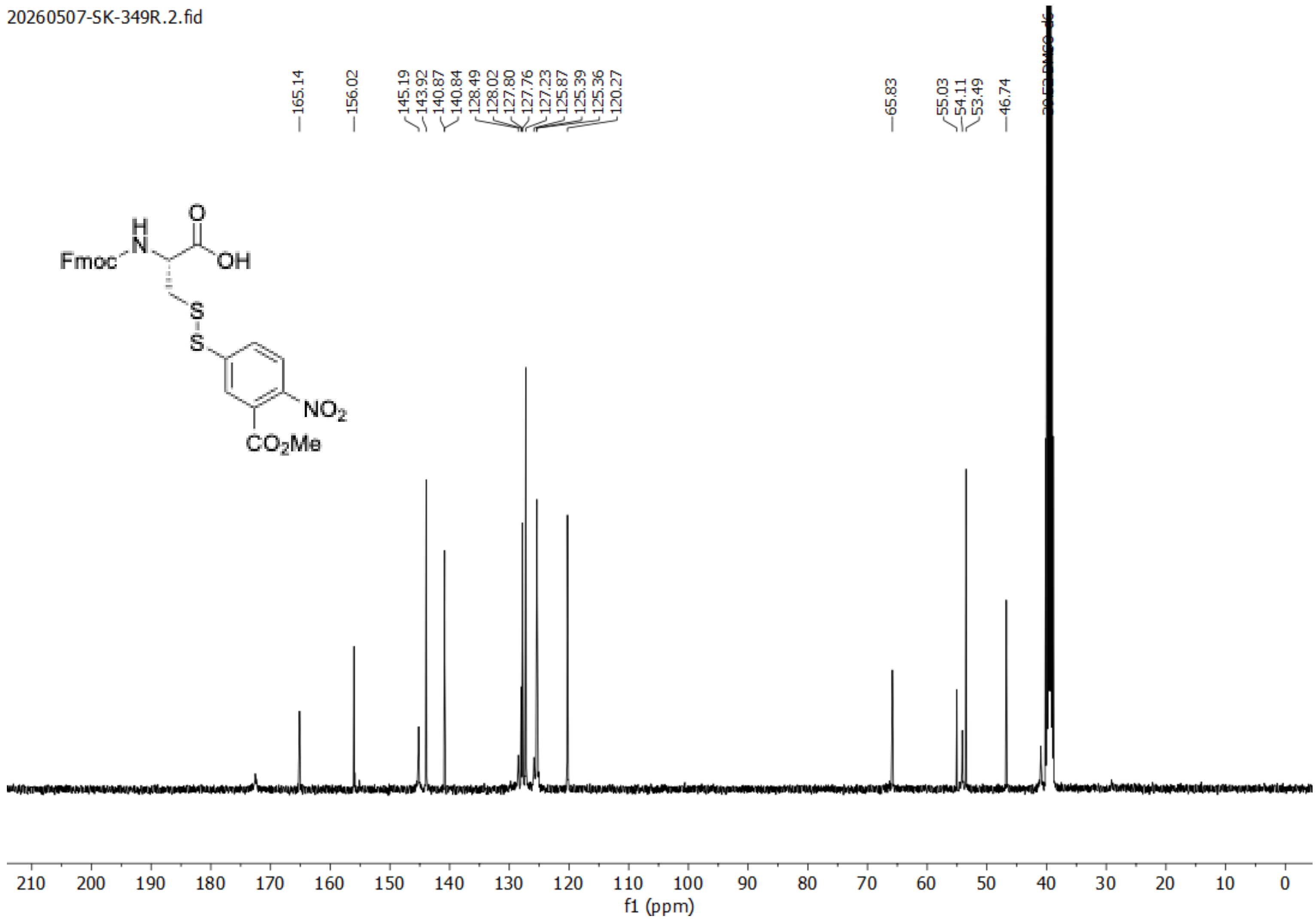

^{1}H NMR of compound **S4** in $CDCl_3$ (400 MHz)

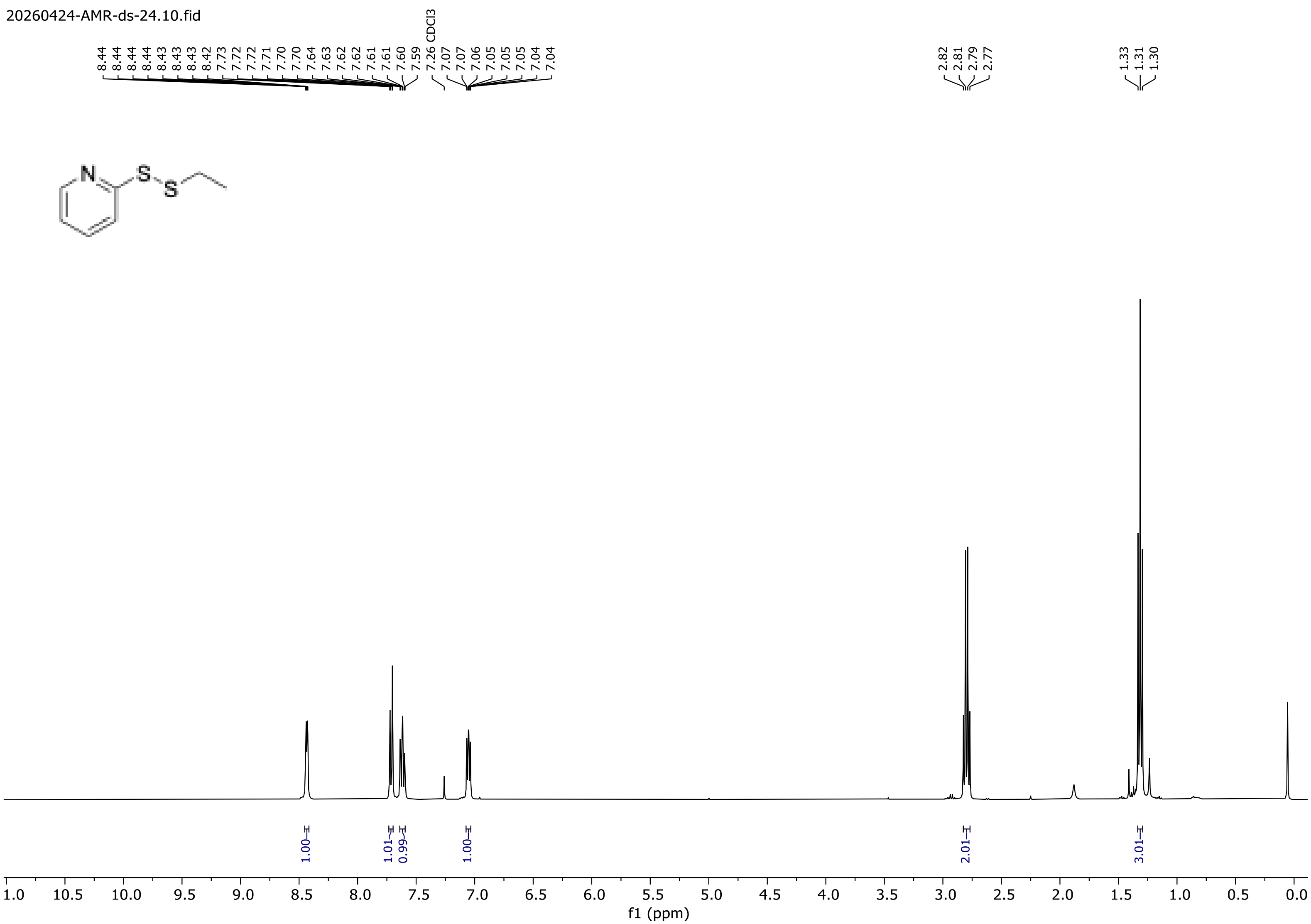

^{1}H NMR of compound **1b** in $CDCl_3$ (400 MHz)

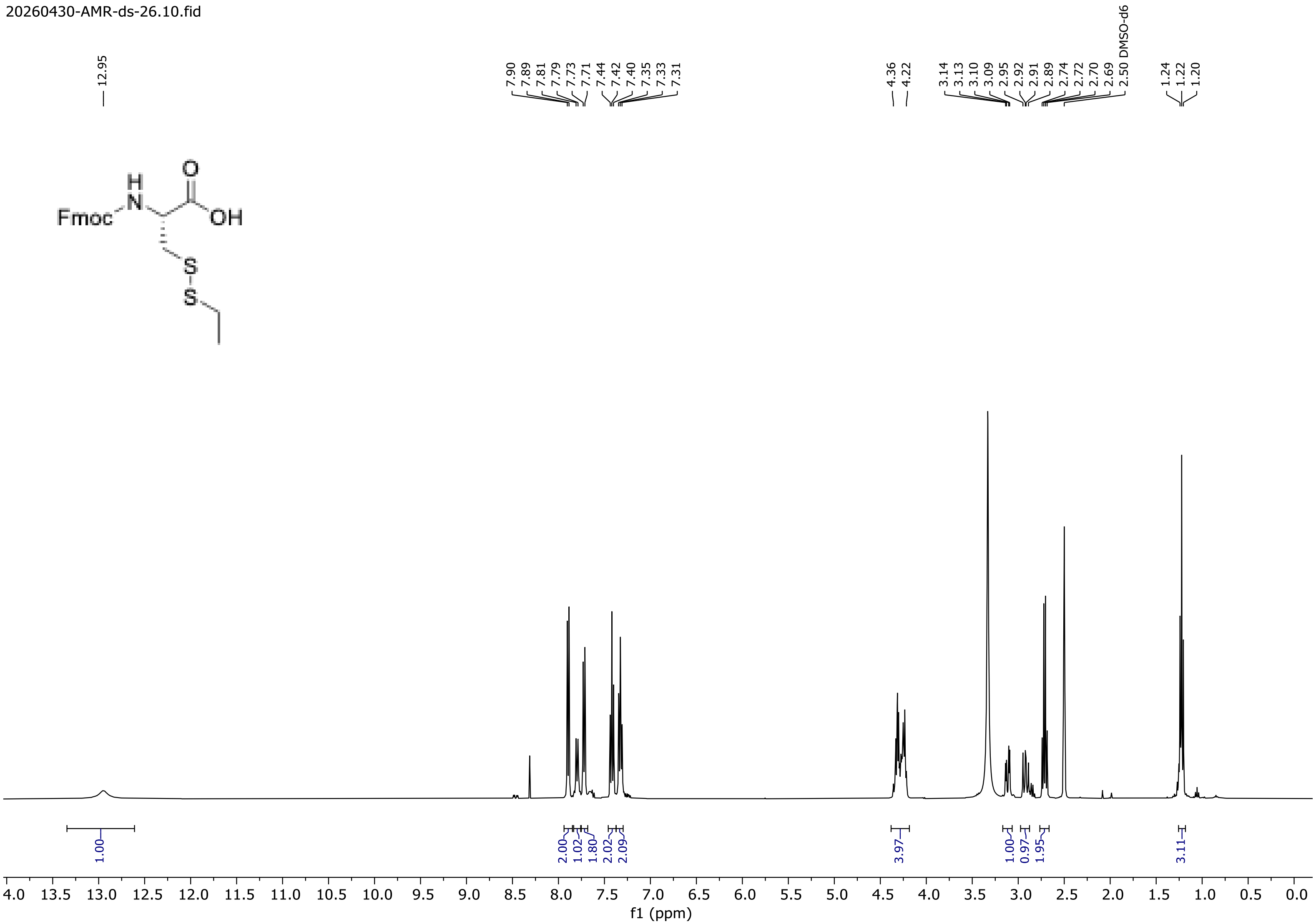

^{13}C NMR of compound **1b** in $CDCl_3$ (400 MHz)

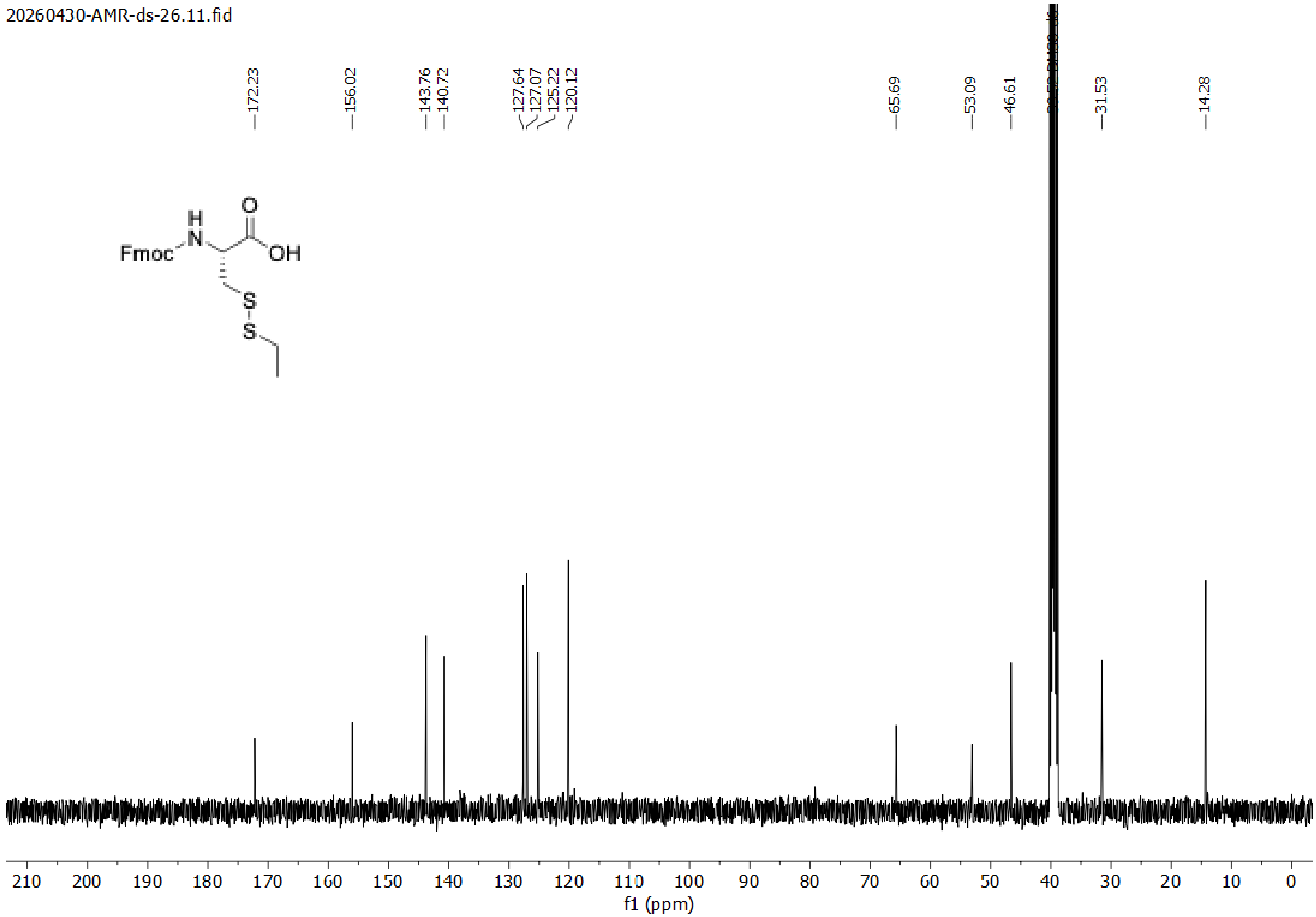

^{1}H NMR of compound **S6** in $CDCl_3$ (400 MHz)

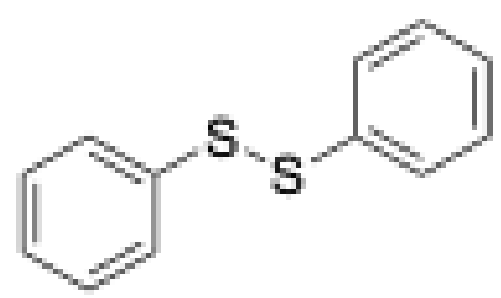

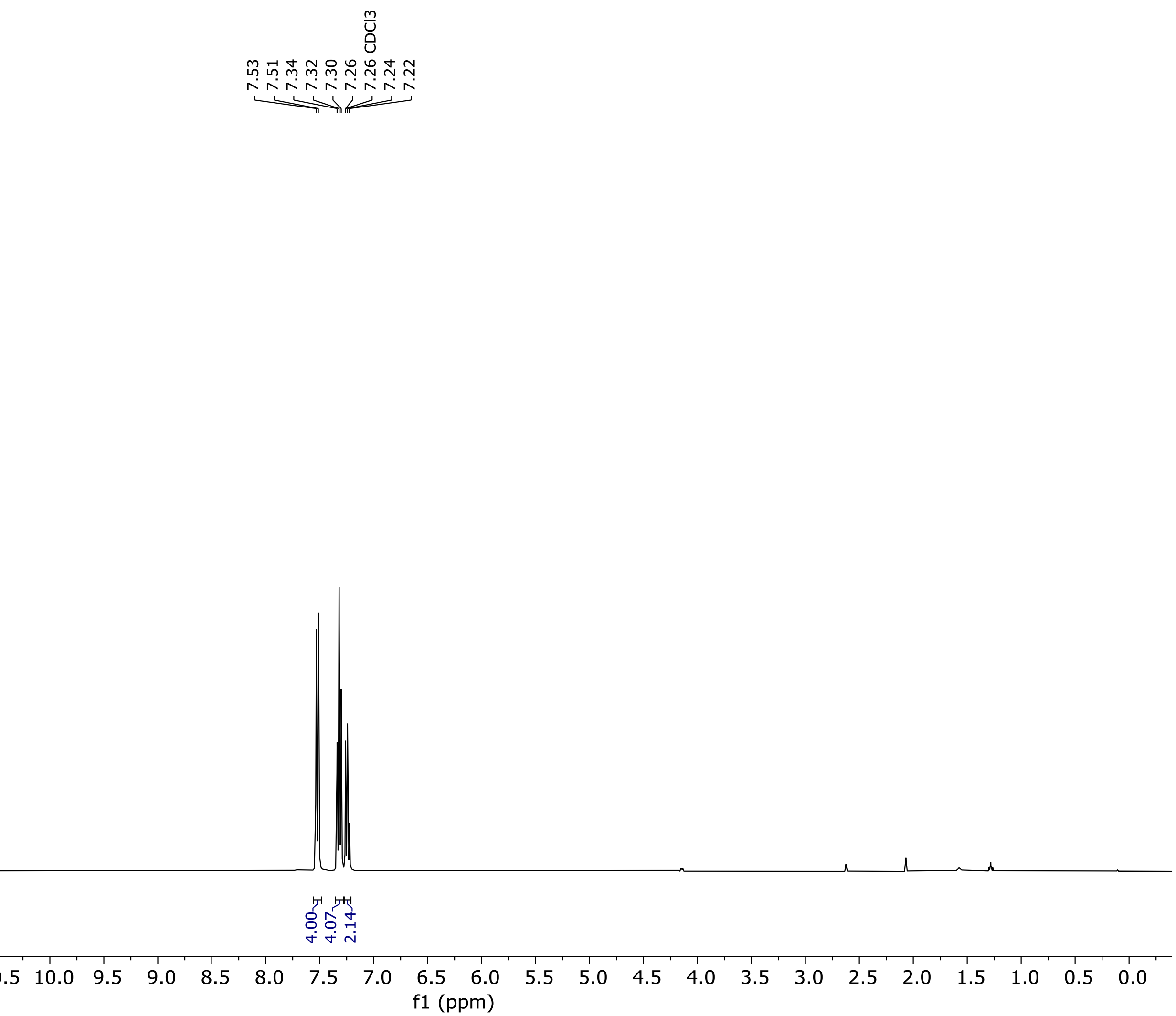

^{1}H NMR of compound **1i** in CDCl3 (400 MHz)

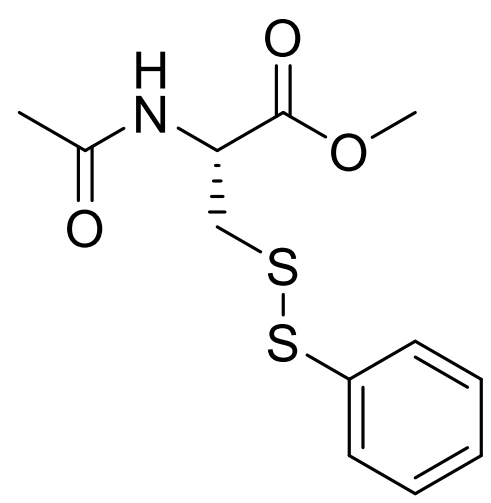

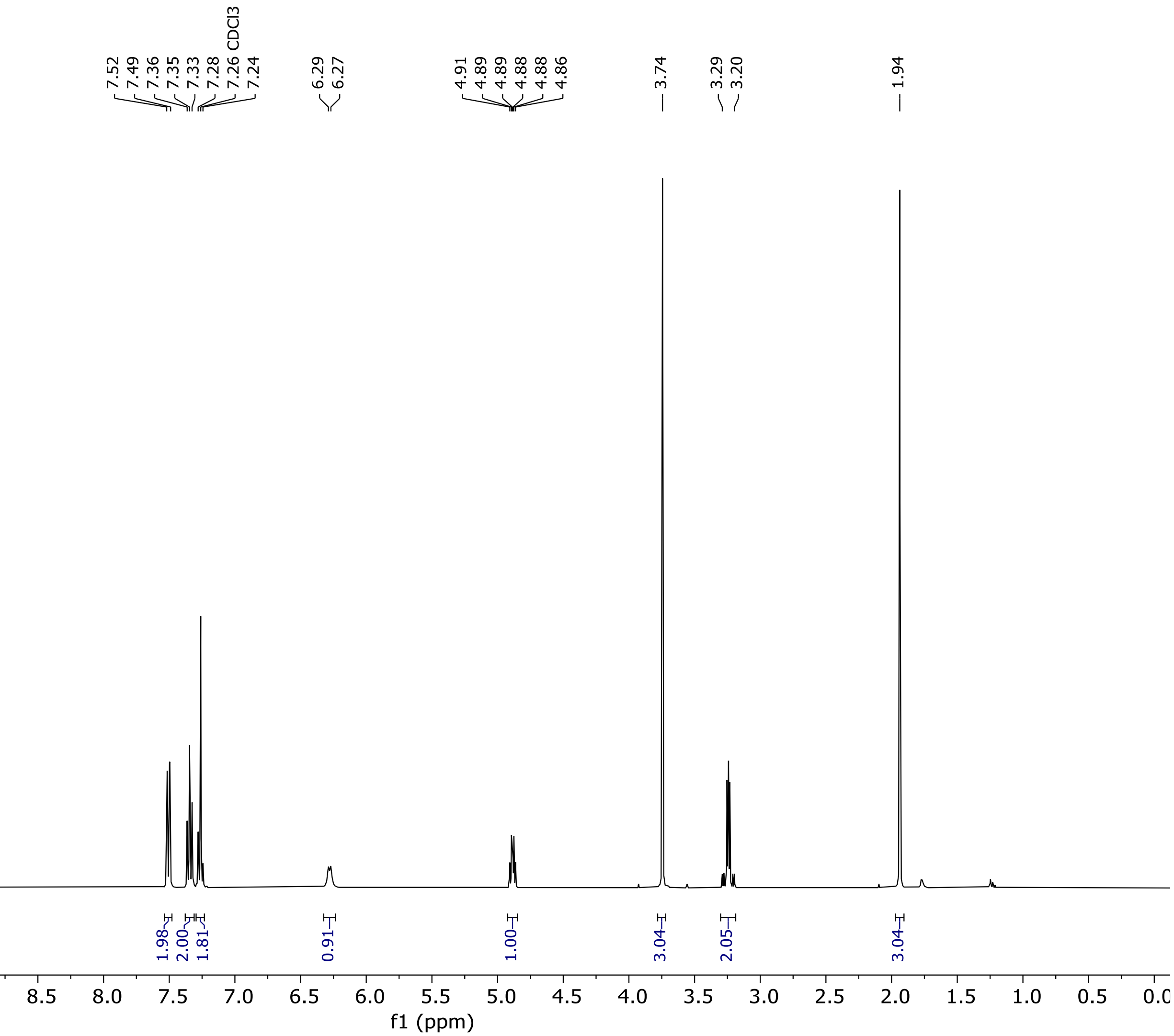

^{13}C NMR of compound **1i** in CDCl3 (400 MHz)

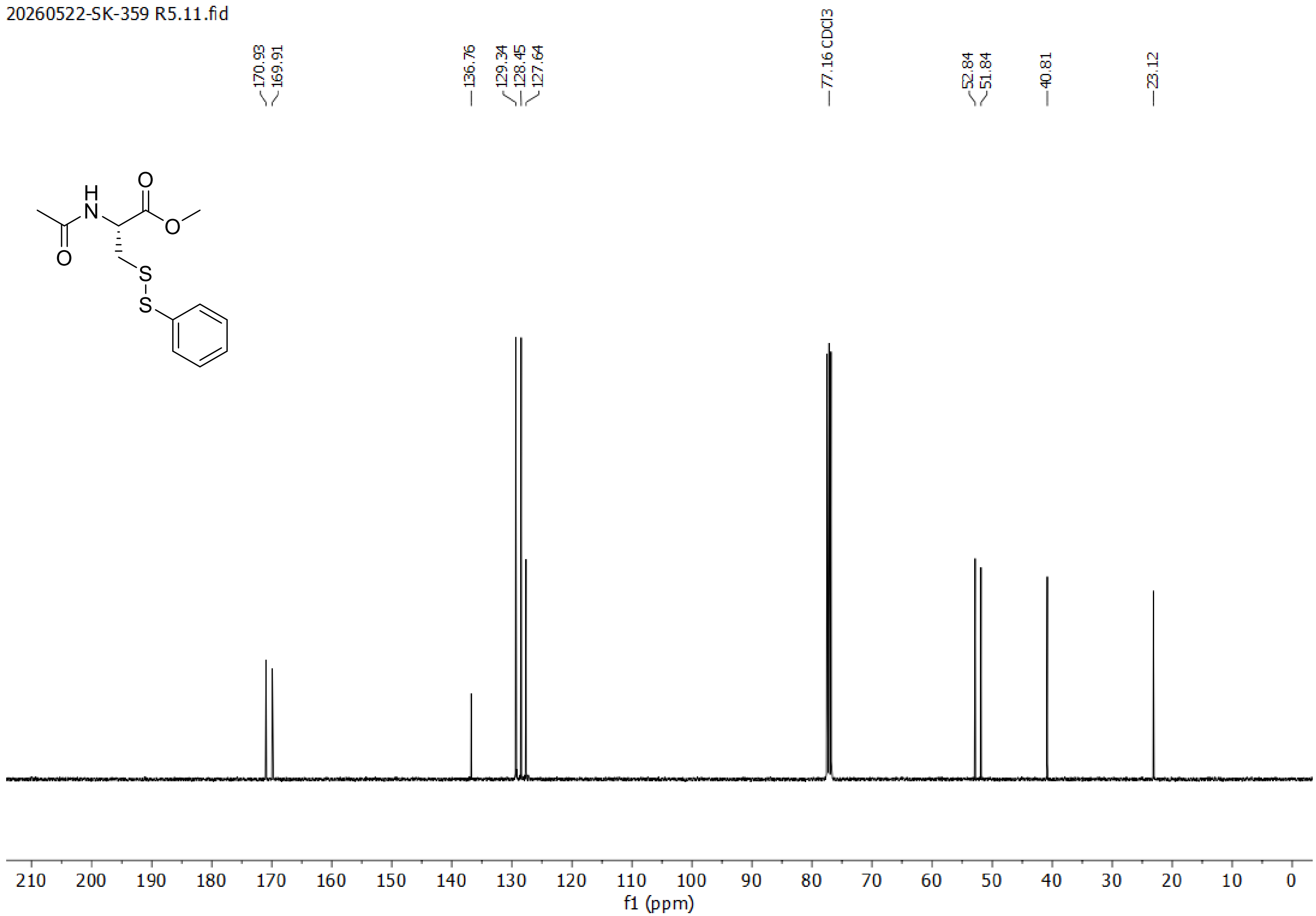

^{1}H NMR of compound **S10** in CD_3CN (400 MHz)

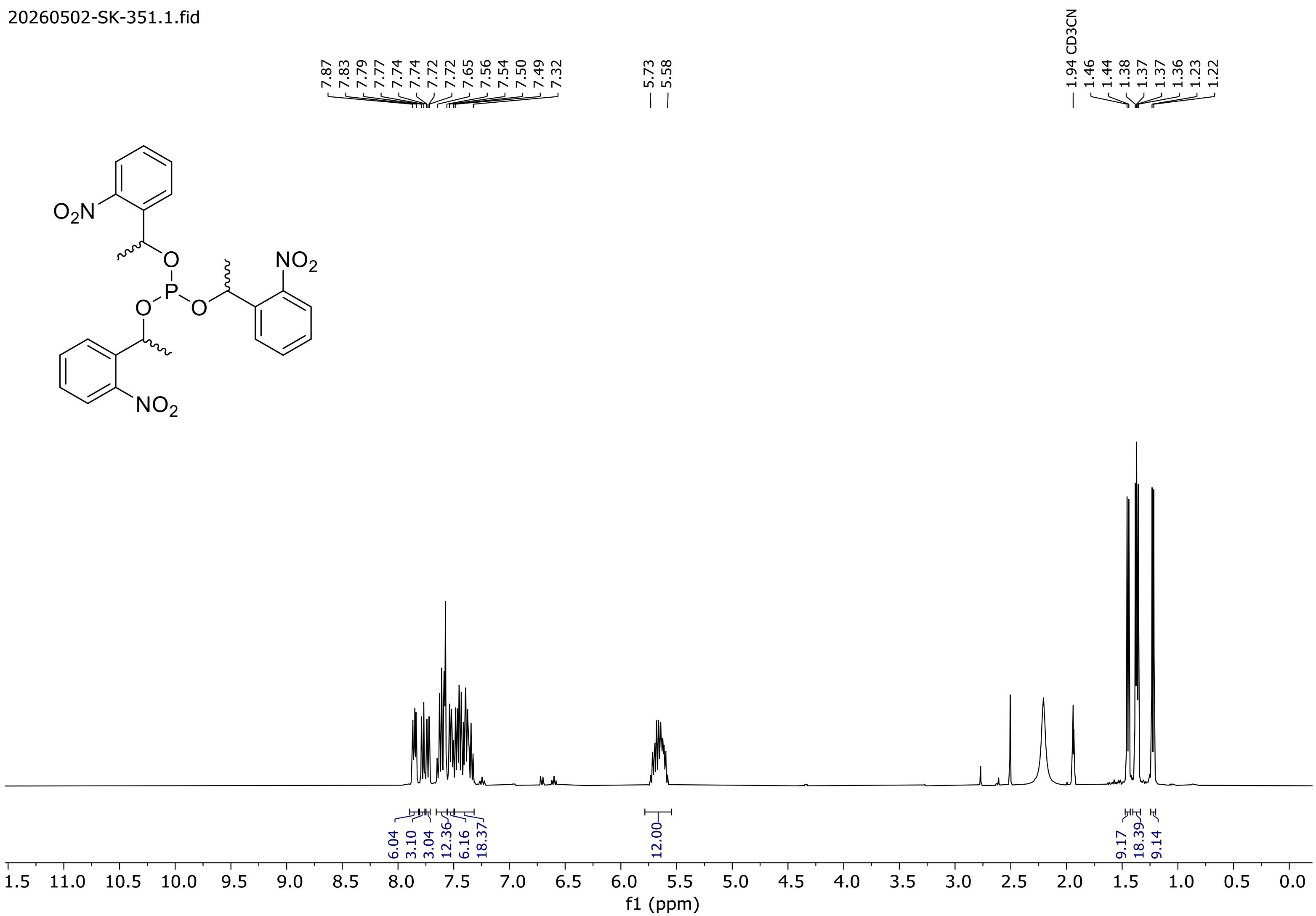

^{13}C NMR of compound **S10** in CD_3CN (400 MHz)

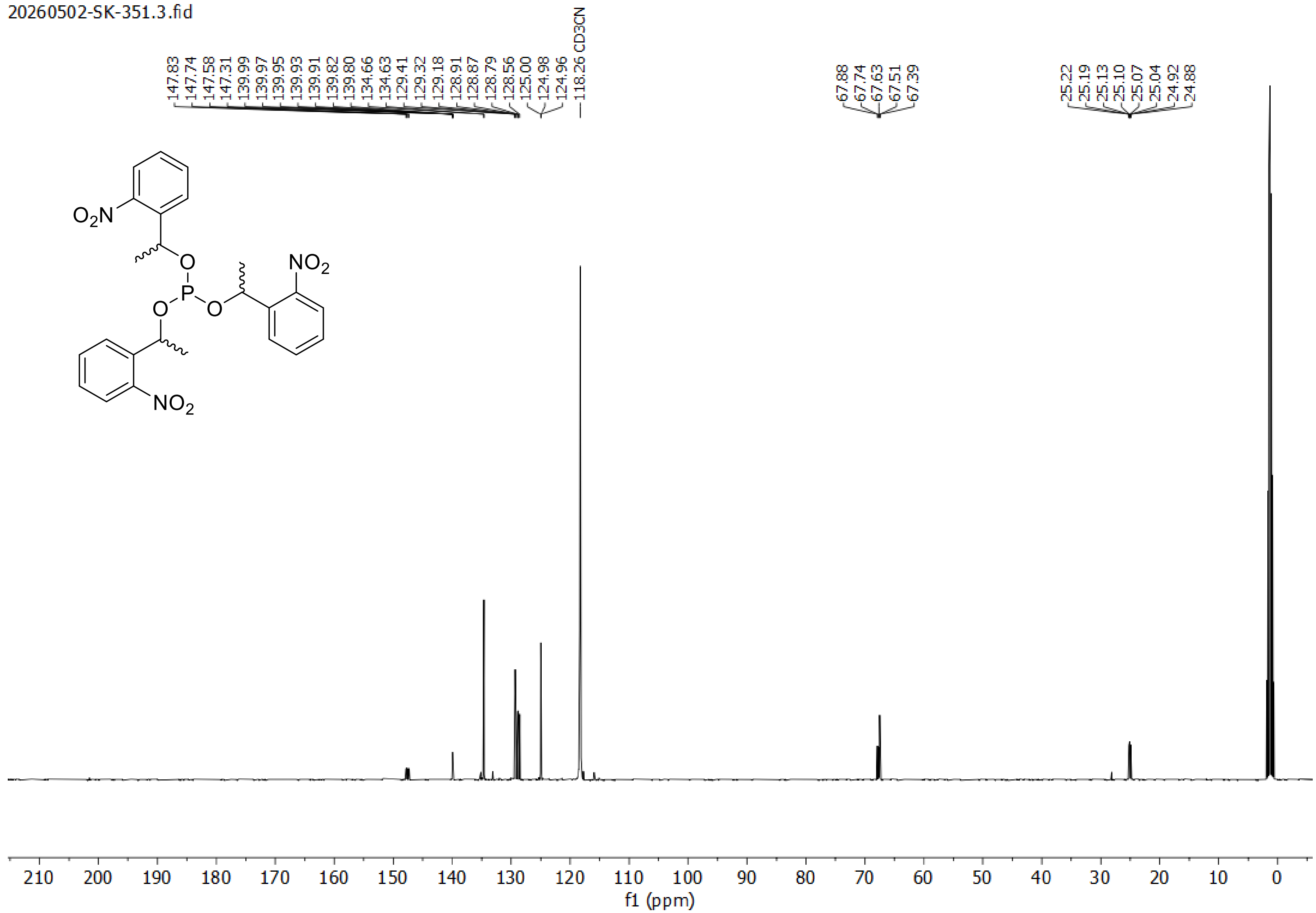

^{31}P NMR of compound **S10** in CD_3CN (400 MHz)

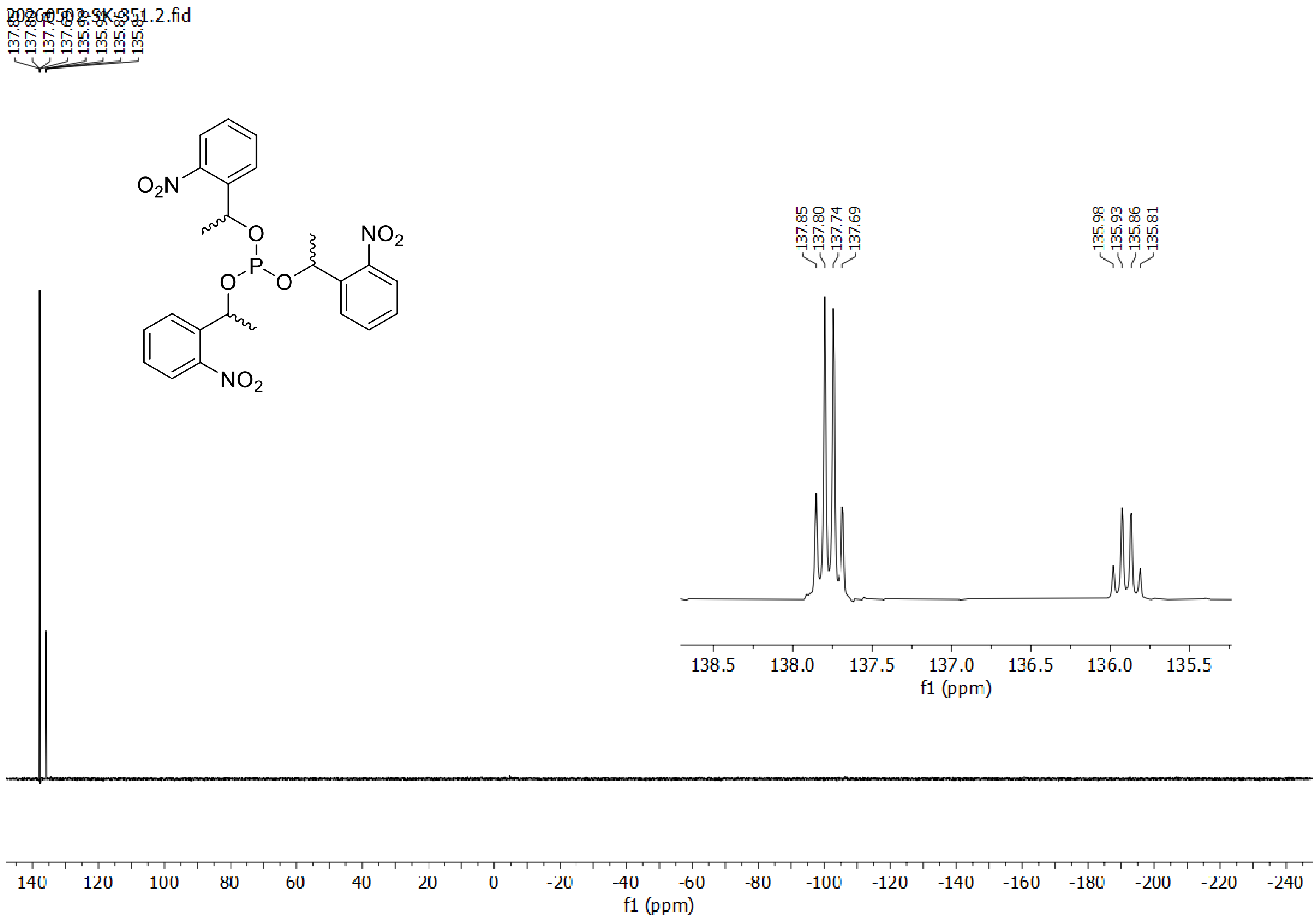

^{1}H NMR of compound **4a** in CD_3CN (600 MHz)

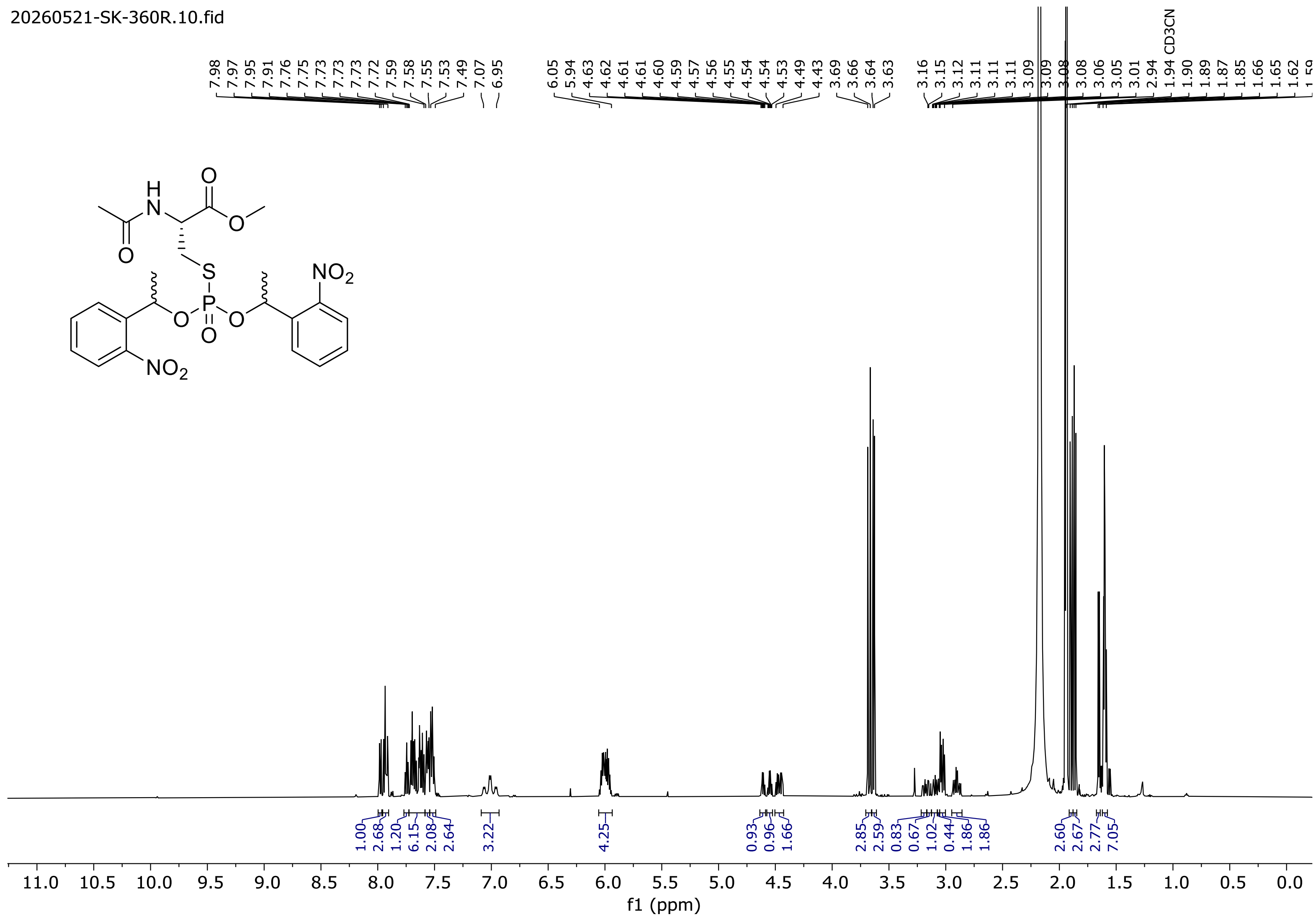

^{13}C NMR of compound **4a** in CD_3CN (600 MHz)

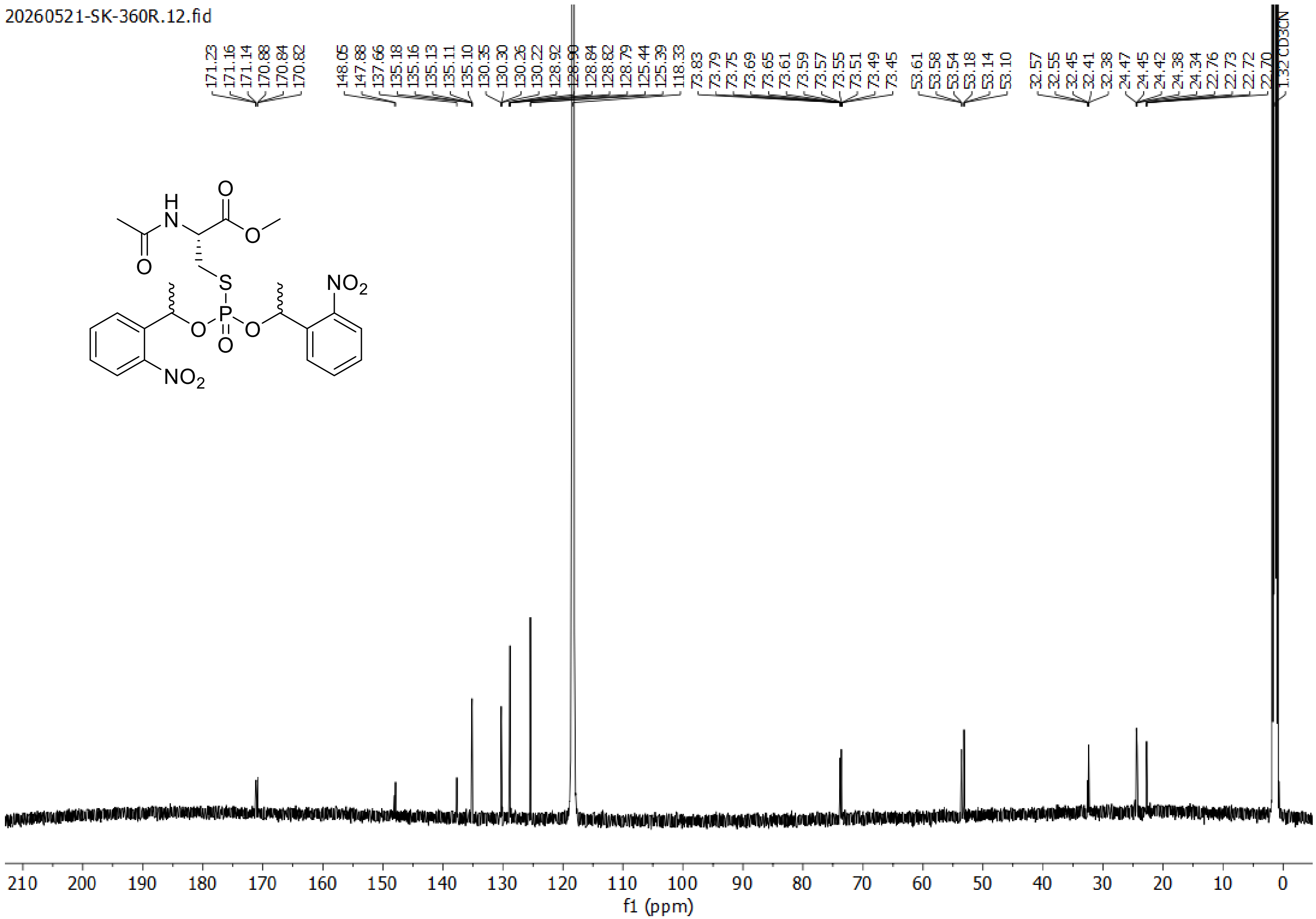

^{31}P NMR of compound **4a** in CD_3CN (600 MHz)

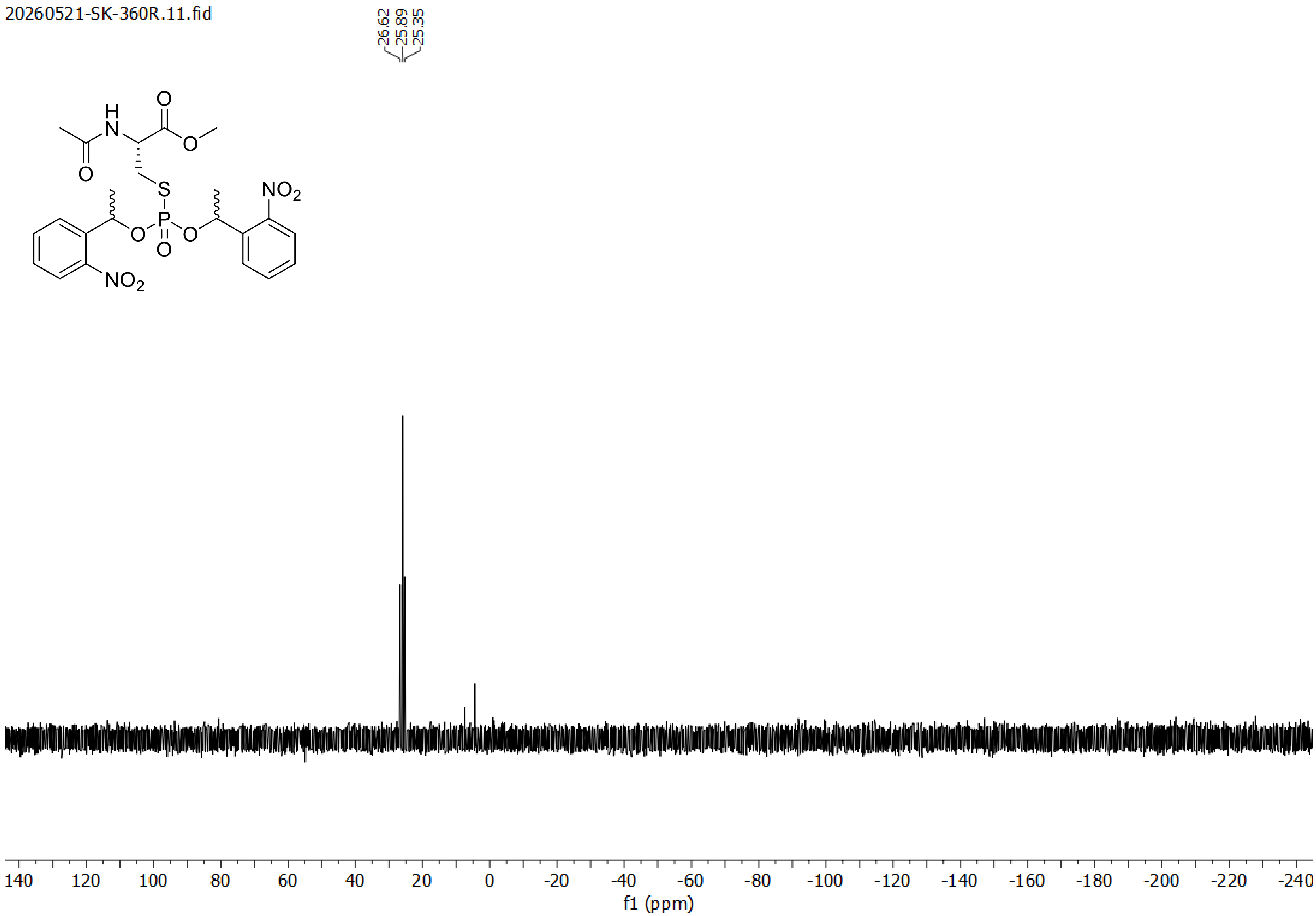

^{1}H NMR of compound **4b** in DMSO-d_6 (400 MHz)

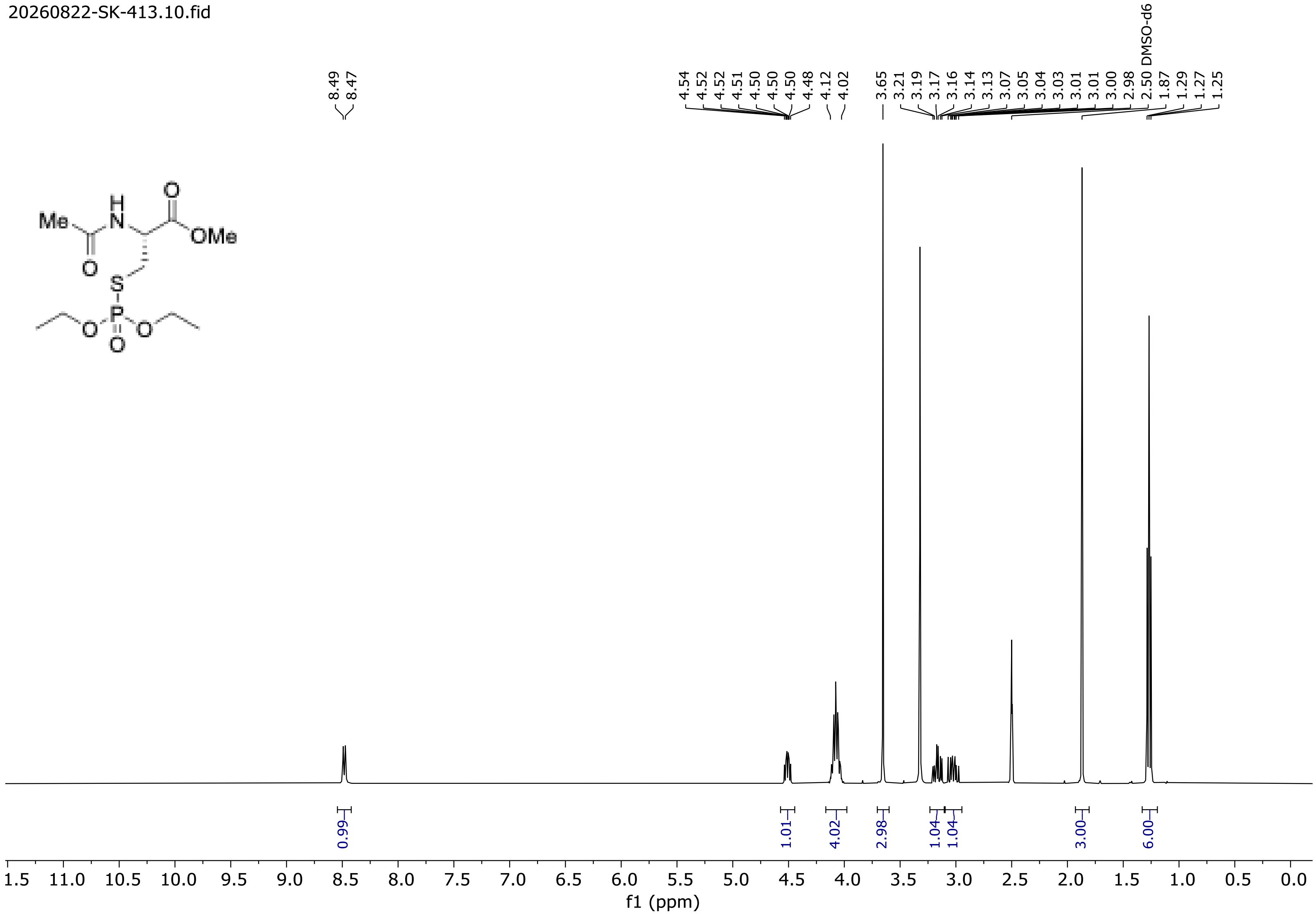

^{13}C NMR of compound **4b** in DMSO-d_6 (400 MHz)

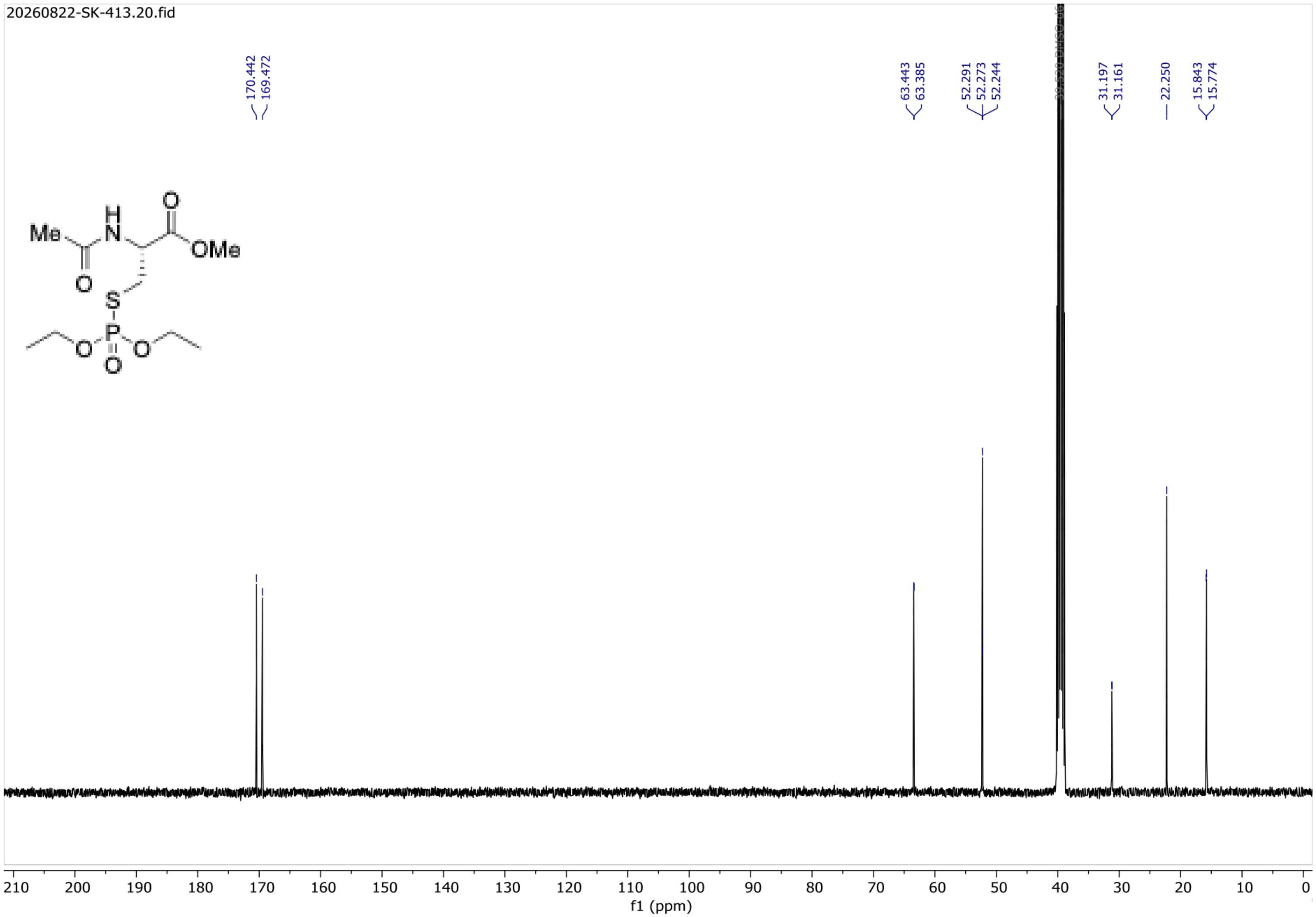

^{31}P NMR of compound **4b** in DMSO-d_6 (400 MHz)

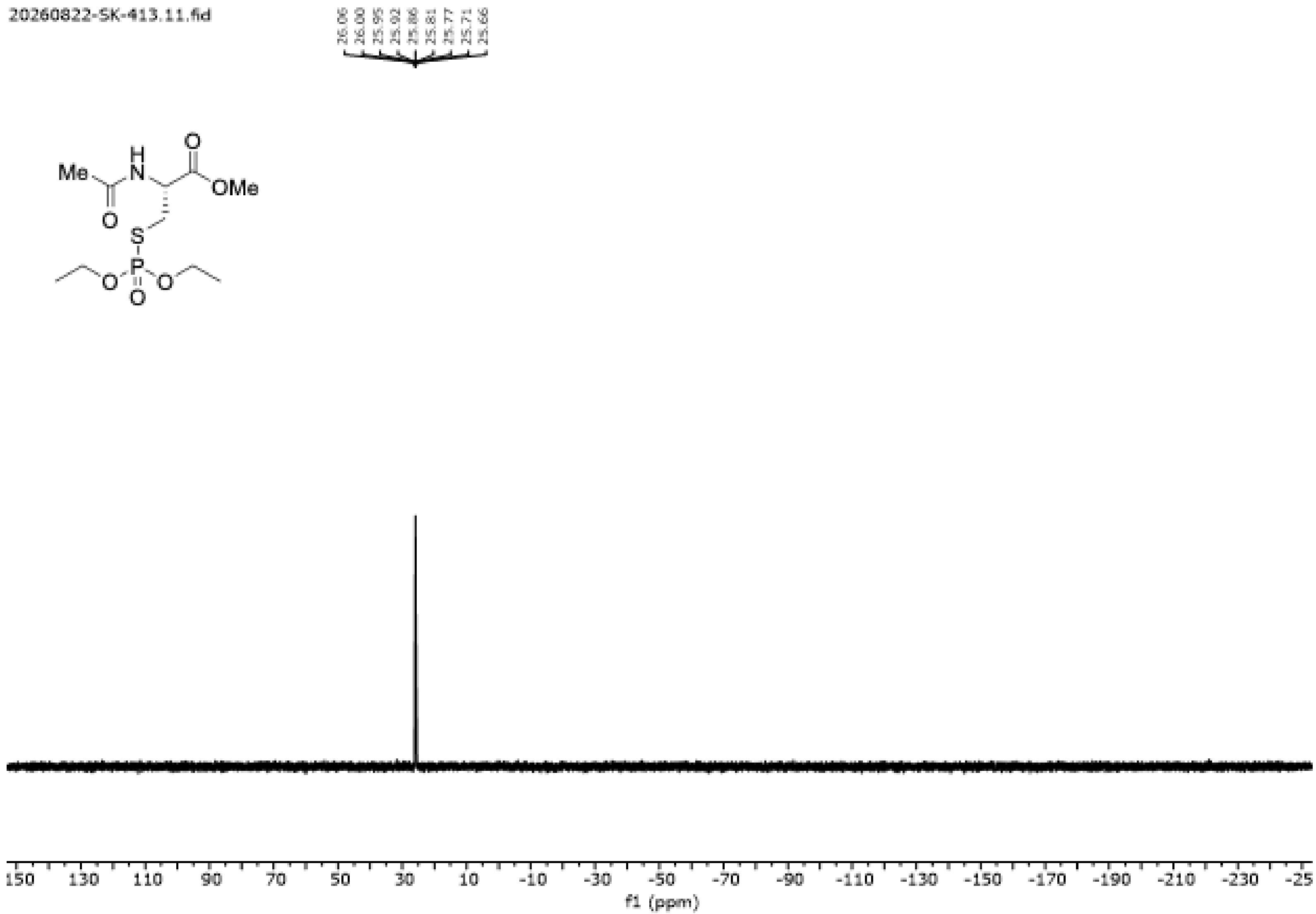

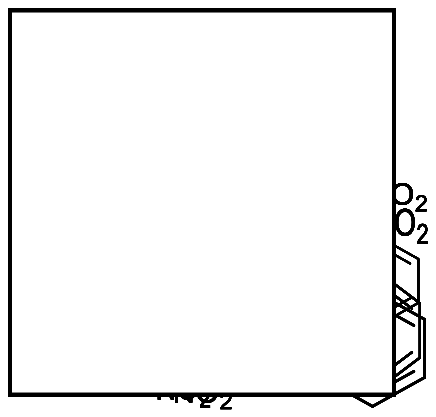

Supplement: Supplementary file 1 [file ol6c03451_si_001.pdf]
